# Enzastaurin inhibits invasion and metastasis in lung cancer by diverse molecules

**DOI:** 10.1038/sj.bjc.6605818

**Published:** 2010-08-24

**Authors:** A Körner, G Mudduluru, C Manegold, H Allgayer

**Affiliations:** 1 Department of Experimental Surgery Mannheim/Molecular Oncology of Solid Tumors (German Cancer Research Center-DKFZ-Heidelberg), Mannheim Medical Faculty, Ruprecht-Karls-University Heidelberg, Mannheim 68167, Germany; 2 Interdisciplinary Thoracic Oncology, Department of Surgery, Medical Faculty Mannheim, University Heidelberg, Mannheim 68167, Germany

**Keywords:** enzastaurin, u-PAR, RASSF1, FHIT, invasion and metastasis

## Abstract

**Background:**

Enzastaurin (Enz) is a serine/threonine kinase inhibitor blocking protein kinase C (PKC)*β*/AKT pathway. However, an ability of this compound to inhibit cancer invasion and metastasis is not yet clearly elucidated.

**Methods:**

The ability of Enz to inhibit invasion and metastasis, and to target molecules was investigated in non-small cell lung cancer (NSCLC) by RT–PCR validated microarray, Matrigel, and *in vivo* chorionallantoic membrane (CAM) assays.

**Results:**

Enzastaurin significantly reduced migration, invasion, and *in vivo* metastasis to lungs and liver (CAM assay) of diverse NSCLC cell lines. Genes promoting cancer progression (*u-PAR*, *VEGFC*, and *HIF1α*) and tumour suppression (*VHL*, *RASSF1*, and *FHIT*) of NSCLC were significantly (*P*<0.05) down- or upregulated after Enz treatment in H460, A549, and H1299 cells, respectively. Luciferase/chromatin immunoprecipitation analysis showed that Enz transcriptionally controls urokinase-type plasminogen activator receptor (u-PAR) expression by promoter inhibition through Sp1, Sp3, and c-Jun(AP-1). Moreover, siRNA knockdown of u-PAR re-sensitised Enz-resistant cells and induced apoptosis, suggesting u-PAR as a marker of Enz resistance.

**Conclusion:**

This study shows that Enz inhibits migration, invasion, and *in vivo* metastasis by targeting u-PAR, besides further targeting progression-related and tumour-suppressor genes in NSCLC. Together with u-PAR being a novel putative marker of Enz response, these data encourage molecularly tailored clinical studies on Enz in NSCLC therapy.

Non-small cell lung cancer (NSCLC) accounts for ∼85% of lung cancer cases and is the leading cause of tumour-related death (Jemal *et al*, 2005; Gridelli *et al*, 2007), progressive stages and metastasis being the most frequent cause for the high NSCLC lethality. Protein kinase C (PKC) isoforms have been shown to be highly expressed in NSCLC as compared to lung epithelial cells (Clark *et al*, 2003). Activation of these isoforms contributes to patient survival, proliferation, and the malignant progression of human cancers including breast carcinoma (Ali *et al*, 2009), B-cell lymphoma (Shipp *et al*, 2002), glioblastoma (da Rocha *et al*, 2002), and colorectal carcinoma (Gokmen-Polar *et al*, 2001). Protein kinase C signalling, which functions through serine/threonine kinase activity, is involved in tumour-induced angiogenesis, tumour growth, differentiation, cytokine secretion, migration, and apoptosis, and is a prominent target for anticancer therapy (Carducci *et al*, 2006). Protein kinase C activation can trigger ERK and PI3K/AKT pathways, promoting cell proliferation and apoptosis (Balendran *et al*, 2000; Goekjian and Jirousek, 2001). Moreover, both PKC and AKT phosphorylate glycogen synthase kinase*β* (GSK3*β*) at Ser9, supporting the notion that these signalling pathways overlap (Fang *et al*, 2002).

Enzastaurin (Enz), an acyclic bisindolylmaleimide, was initially developed as a specific inhibitor of PKC*β* (Faul *et al*, 2003). In addition to this major target, Enz also inhibits other PKC isoforms (*δ*, *α*, *ε*, and *γ*) (Graff *et al*, 2005). Enzastaurin was initially developed for anti-angiogenic cancer therapy (Keyes *et al*, 2002), and recent preclinical studies have shown that Enz exerts proapoptotic, growth inhibitory, and anti-angiogenic properties in a variety of human cancers (Keyes *et al*, 2002; Graff *et al*, 2005; Querfeld *et al*, 2006; Willey *et al*, 2009). Moreover, in clinical studies, Enz was well tolerated and has shown encouraging activity in a variety of tumours (Carducci *et al*, 2006; Watkins *et al*, 2006; Hanauske *et al*, 2009). Enzastaurin was shown to target the PI3K/AKT pathway, to inhibit GSK3*β* and ribosomal protein S6 phosphorylation, to decrease the expression of VEGF (Keyes *et al*, 2002; Graff *et al*, 2005) and also to show a synergism with SUI174 and pemetrexed (Tekle *et al*, 2008; Faoro *et al*, 2009; Giovannetti *et al*, 2010).

Tumour progression is a multistep process including uncontrolled growth, invasion, and metastasis (Douglas and Robert, 2000), which, to a considerable part, is mediated through an abnormal regulation and function of genes. Several studies have analysed the involvement of individual genes (Müller-Tidow *et al*, 2001; Marchetti *et al*, 2002; Werle *et al*, 2004), gene expression differences between cell lines (Chen *et al*, 2001; Gemma *et al*, 2001), and gene expression differences between primary tumours and normal lung tissues in this context (Bhattacharjee *et al*, 2001; Hellmann *et al*, 2001; Beer *et al*, 2002; Heighway *et al*, 2002; Wikman *et al*, 2002; Zochbauer-Muller *et al*, 2002; Borczuk *et al*, 2003; Diederichs *et al*, 2004; Hayes *et al*, 2006). The urokinase-type plasminogen activator (u-PA) and its receptor (u-PAR) are essential for proteolysis in many solid tumours, the main mechanism being the activation of plasminogen-dependent proteolysis and a cascade of protolytic activities, leading to extracellular matrix degradation, invasion, intravasation, and metastasis establishment (Jo *et al*, 2003; Dass *et al*, 2008). Extensive clinical studies have shown that the expression of u-PAR, u-PA, and its specific inhibitor PAI-1 is associated with tumour recurrence and poor survival of diverse cancer types (Werle *et al*, 2004; Beyer *et al*, 2006). In addition to the u-PAR, an overexpression of further molecules such as hypoxia-inducible factor 1*α* (HIF1*α*), a transcription factor, and vascular endothelial growth factor C (VEGFC), a major inducer of angiogenesis, has been specifically shown to correlate with poor prognosis, tumour progression, and metastasis of NSCLC (Zhang *et al*, 2007; Wang *et al*, 2009). Genes *u-PAR*, *HIF1α*, and *VEGFC* are transcriptionally activated by, for example, MAPK- and/or PI3K/AKT-related pathways (Jo *et al*, 2003; Tsai *et al*, 2003; Phillips *et al*, 2005; Skurk *et al*, 2005). Tumour-suppressor genes *VHL*, *RASSF1*, and *FHIT* are downregulated in NSCLC (Zochbauer-Muller *et al*, 2002; Hayes *et al*, 2006), which are implicated in the ubiquitin ligase system (Karhausen *et al*, 2005), cell-cycle regulation (Dammann *et al*, 2000), and apoptosis (Ji *et al*, 1999), respectively.

Although Enz has been reported to reduce tumour growth and to enhance apoptosis, a potential ability of this compound to inhibit invasion and metastasis as the most important characteristics of malignant tumours has never been studied. Therefore, this study was conducted to (1) determine the effects of Enz on migration, invasion, and *in vivo* metastasis; and (2) determine and implicate first molecular targets mediating this ability of Enz in NSCLC. Furthermore, in a systematic profiling approach we sought to give general insights into important target molecules of Enz in NSCLC.

Our study reveals that PKC inhibition by Enz reduces NSCLC proliferation, migration, invasion, and *in vivo* metastasis. Moreover, we show that *u-PAR*, *VEGFC*, and *HIF1α*, among other targets, are being downregulated, and the tumour-suppressor genes *FHIT*, *RASSF1*, and *VHL* are upregulated upon Enz treatment. We further describe u-PAR as an essential target being transcriptionally regulated by Enz through Sp1, Sp3, and c-Jun(AP-1) binding to the promoter, and as a putative novel marker of Enz sensitivity.

## Materials and methods

### Materials, cell lines, and reagents

Enzastaurin (in DMSO) was a generous gift from Eli Lilly (Indianapolis, IN, USA). All cell lines (H460, LXF289, A427, H1395, A549, and H1299) were obtained from the American Type Culture Collection (ATCC, Rockville, MD, USA) and cultured in ATCC-recommended medium (10% FBS, 37°C, 5% CO2). Fertilised special pathogen-free eggs were from Charles River (Hilden, Germany). Antibodies used for supershift/chromatin immunoprecipitation analysis (ChIP) experiments were from Santa Cruz Biotechnology (Santa Cruz, CA, USA): p-c-Jun (sc-822x), JunD (sc-74x), JunB (sc-46x), c-Fos (sc-52x), FosB (sc-7203x), Fra-1 (sc-22794x), Fra-2 (sc-604x), Sp1 (sc-59x), Sp3 (sc-644x), and unspecific IgG (sc-2338). Western blot antibodies against phospho-PKC(PAN) 9371, phospho-p44/42(Thr202/Tyr204) 4370, p44/42 9102, phospho-GSK3*β*(Ser9) 9336, and phospho-Akt(Ser473) 4058 were from Cell Signaling, Danvers, MA, USA, and Akt1 (sc-1618) and *β*-Actin (sc-1616R) were from Santa Cruz Biotechnology.

### Proliferation assay

Cells (3 × 10^3^) per well were seeded in a 96-well plate in a total volume of 100 *μ*l medium with 10% FBS. After 24 h, cells were treated either with DMSO or Enz at concentrations between 0 and 15 *μ*M. After 72 h, the growth inhibitory effect was evaluated using 20 *μ*l per well of CellTiter96 AqueousOneSolution (Promega, Madison, WI, USA) according to the manufacturer's instructions.

### Wound-healing assay

Cells (3 × 10^5^) per well were seeded in a 24-well plate in a total volume of 500 *μ*l medium (10% FBS). After 24 h, the medium was changed to serum-free and a wound created in the monolayer by using a pipette tip. Cells were washed twice (PBS), and incubated with fresh medium supplemented with DMSO or Enz. In case of EGF stimulation, EGF (100 nM) was added 4 h after initial DMSO or Enz treatment. Digital pictures were taken at 0 and 48 h. To quantify migration, we calculated the diameter of the wound as the average of three different locations.

### Matrigel invasion assay

Cells (1 × 10^5^), starved in serum-free medium (24 h), were plated on transwell chambers precoated with 10 *μ*g Matrigel. DMSO or Enz was applied to bottom and top chambers. In case of EGF, cells were pretreated (4 h) with DMSO or Enz, followed by EGF (100 nM). 10% FBS medium in the bottom chamber was used as a chemoattractant. After 24 h, noninvading cells were removed with cotton swabs. Invaded cells were trypsinised and counted using the ATP luminescence-based motility-invasion assay as described (Leupold *et al*, 2007).

### cDNA labelling, array hybridisation, and data analysis

Total RNA was isolated as described (Mudduluru and Allgayer, 2008), and probe labelling and array hybridisation on Illumina Human Sentrix-8 BeadChip arrays (Illumina, San Diego, CA, USA) were conducted according to Illumina's recommended sample labelling procedure based on the modified Eberwine protocol (Eberwine *et al*, 1992). Scanning was carried out using a Beadstation array scanner, adjusted to a scaling factor of 1 and PMT settings at 430. Data extraction was carried out for all beads individually, and outliers were removed when median absolute deviation was >2.5. All remaining data points were used for the calculation of the mean average signal for a given probe, and standard deviation for each probe was calculated. Data analysis was carried out by normalisation of the signals using the quantile normalisation algorithm without background subtraction, and differentially regulated genes defined by calculating the standard deviation differences of a given probe in a one-by-one comparison of samples.

### Luciferase reporter assay

Cells were transiently transfected with 500 ng of −398/+51 u-PAR WT/Sp1- or AP1-mutant plasmid, or with pGL3 basic (Allgayer *et al*, 1999), and 50 ng of Renilla-luciferase plasmid as a transfection control using Effectene (Qiagen, Hilden, Germany). After 24 h, cells were treated with DMSO or Enz for another 24 h, and reporter assays performed using the Dual-Luciferase reporter assay system (Promega).

### Real-time PCR

Total RNA isolation and cDNA synthesis was performed as described (Mudduluru and Allgayer, 2008). The u-PAR (Hs00182181; Applied Biosystems, Carlsbad, CA, USA) mRNA was quantified using TaqMan Universal PCR Master Mix and *β*-Actin (Human ACTB 4333762F; Applied Biosystems) as housekeeping gene. Quantification of HIF-1*α*, VEGFC, VHL, RASSF1, FHIT, Sp1, Sp3, and c-Jun mRNA was performed using SYBR Green-PCR-Master-Mix (Applied Biosystems). Relative expression of these genes was calculated by the 2^−ΔΔCT^ method. Primer sequences are provided in Supplementary Table 1.

### ELISA and western blotting

ELISA and western blot analysis were performed as described (Leupold *et al*, 2007; Mudduluru and Allgayer, 2008). In brief, for ELISA, cells were either treated with DMSO or with Enz for 24 h. For western blot analysis, cells were serum starved for 24 h and treated with DMSO or Enz for another 24 h. Cells were stimulated with EGF (100 nM) for 15 min. Cells were washed twice with ice-cold PBS and lysed with lysis buffer (Biosource, Camarillo, CA, USA). Protein concentration was determined by BCA (Pierce, Rockford, IL, USA). The u-PAR protein was assayed using the Imubind-u-PAR-ELISA kit (American Diagnostica, Hauppauge, NY, USA).

### Transfection of si-uPAR

si-u-PAR(ID289377; Ambion, Austin, TX, USA) was used to knockdown the expression of u-PAR. A nonspecific si-RNA (scrambled) served as a negative control. Sh-RNA vectors of sh-u-PAR (RHS4430-99290516), and non-silencing control (RHS4346) were purchased from Open Biosystems (Huntsville, AL, USA). A549 and H1299 cells were transiently transfected using RNAiFect or Effectene, respectively (Qiagen). At 24 h after transfection, cells were used for MTT and Matrigel assays as described.

### Apoptosis assays

Cells (1 × 10^6^ per ml) were treated with Enz (as the respective IC_50_ values) and harvested after 48 h either with or without transfections. Apoptosis induction by Enz was measured by the FITC Annexin V Apoptosis Detection Kit I (BD Pharmingen, San Diego, CA, USA) as described (Querfeld *et al*, 2006). According to the manufacturer's instructions, cells were incubated with 5 *μ*l per test FITC-conjugated annexin V in the presence of 5 *μ*l per test PI, and further screened by flow cytometry (BD Biosciences, San Diego, CA, USA). Annexin V-positive PI-positive cells correspond to apoptotic cells and are represented as a percent of apoptotic cells against DMSO-treated cells.

### Electrophoretic mobility shift and ChIP assay

Gel shifts were performed as described (Mudduluru and Allgayer, 2008), using 5 *μ*g of nuclear extract of DMSO- or Enz-treated (IC_50_, 24 h) A549 cells. Chromatin immunoprecipitation was performed according to the manufacturer's protocol (Upstate, Lake Placid, NY, USA) as described (Leupold *et al*, 2007; Mudduluru and Allgayer, 2008), with 2 *μ*g specific (Sp1, Sp3, and p-c-Jun) and nonspecific IgG overnight, using aliquots of precleared lysates. DNA was purified and eluted with 100 *μ*l elution buffer (Qiagen reaction purification kit). PCR was performed according to Leupold *et al* (2007), for region −190/−171 (u-PAR-AP1). Amplification of region −152/−135 (u-PAR-Sp1/Sp3/AP-2*α*-like) was performed by SYBR Green Master Mix, with the specific primers: For 5′-AGGCAATCTGGGGACAGAG-3′ and Rev 5′-GGACTCCTCCCAGACGTTTT-3′.

### Chorionallantoic membrane assay

The chorionallantoic membrane (CAM) assay was performed as described (Leupold *et al*, 2007), with few modifications. Briefly, 2 × 10^6^ cells were inoculated on the CAM of 10-day-old chicken embryos. On days 12 and 14, chicken embryos were treated intravenously with different concentrations of Enz (2 or 4 *μ*M) or DMSO, respectively. Untreated embryos served as a control. On day 16, the embryos were killed. Metastasis was determined by harvesting lungs and liver, and processing the tissue for genomic human DNA by quantitative *alu-*PCR.

### Statistical analysis

Statistical significance of differences between Enz- *vs* DMSO-treated samples were calculated with the Student's *t*-test (*P*⩽0.05 was considered as statistically significant, *P*⩽0.1 as a trend).

## Results

### Enzastaurin inhibits the growth of human NSCLC cells

**Figure 1:**
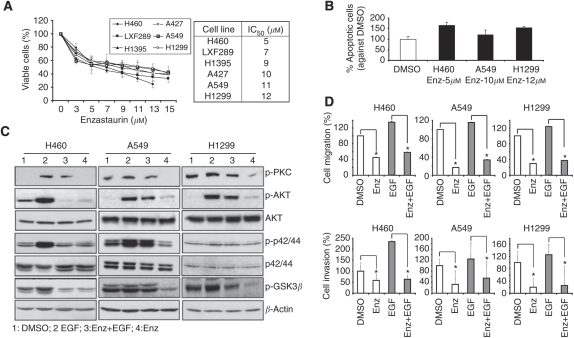
Enzastaurin inhibits NSCLC proliferation, migration, and invasion. (**A**) Cell proliferation of NSCLC after Enz treatment. Data are expressed as the percentage of control (treated with DMSO) cells. Points, mean±s.d. of three experiments. Resulting IC_50_ values for Enz for all NSCLC cell lines investigated are represented in the box. (**B**) Apoptosis analysis of H460, A549, H1299 after Enz treatment. After 48 h of Enz treatment, cells were screened by flow cytometry and percent of apoptotic cells was calculated against to DMSO-treated cells. (**C**) Western blot analysis of cells treated with EGF, Enz, or with the combination of both agents. Specific antibodies against the indicated molecules were used, together with anti-beta-Actin as a loading control. (**C** and **D**) Percentage of migrating cells (would-healing assay) and invading cells (Matrigel assay). Cells were treated as described in the Materials and Methods section. DMSO-treated samples served as a control (100%), and other treated samples were calculated and plotted as a percentage of this value. ^*^P<0.05.

Six human NSCLC cell lines were tested for their sensitivity to Enz regarding proliferation (Figure 1A). Large cell-type H460 and adenocarcinoma LXF289 cells showed the highest sensitivity. A moderate response was observed in H1395 (adenocarcinoma) and A427 (squamous carcinoma) cells, whereas A549 (squamous carcinoma) and H1299 (adenocarcinoma) cells showed comparatively more resistance to Enz. Mean IC_50_ values of the cells are presented in the box in Figure 1A. Similarly, viable cells were counted after Enz treatment, confirming the IC_50_ concentrations determined by MTT assays (data not shown). Next, to determine if the calculated IC_50_ concentrations of Enz were able to induce apoptosis and inhibit the known target molecules of Enz, apoptotic assays and western blot analysis were performed. H460, A549, and H1299 cells were treated for 48 h with their respective IC_50_ concentrations. Enzastaurin (IC_50_ concentrations) induced apoptosis of H460, A549, and H1299 cells compared with the DMSO-treated samples was shown in Figure 1B. Because EGFR-induced signalling represents a major pathway driving NSCLC progression, we in addition sought to investigate the ability of Enz to inhibit essential target molecules following EGF stimulation. H460, A549, and H1299 cells were pretreated either with DMSO, or with Enz, for 24 h, and stimulated, or unstimulated, with EGF (100 nmol l^−1^) for 15 min before harvesting the cells. As results, we found that Enz treatment can inhibit the phosphorylation of PKC, AKT, p42/44, and GSK3*β* both in the presence and in the absence of EGF (Figure 1C, Supplementary Figure 1).

### Enzastaurin affects cell migration and invasion

To determine whether Enz affects the migration and invasion of NSCLC cells *in vitro*, we evaluated the migratory (wound-healing assay) and invasive (Transwell *in vitro* invasion assay) capacity of H460, A549, and H1299 cells, after treatment. Enzastaurin- or Enz/EGF-treated cells displayed a significantly reduced migration and invasion as compared to DMSO- and EGF-stimulated control cells; respectively (*P*<0.02; Figure 1D), showing that Enz inhibits EGF-induced cell migration and invasion in addition to spontaneous invasion in NSCLC. This prompted further experiments to study changes in gene expression upon Enz treatment, to delineate major target molecules mediating Enz-inhibited invasion.

### Analysis of gene expression changes after Enz treatment

**Figure 2:**
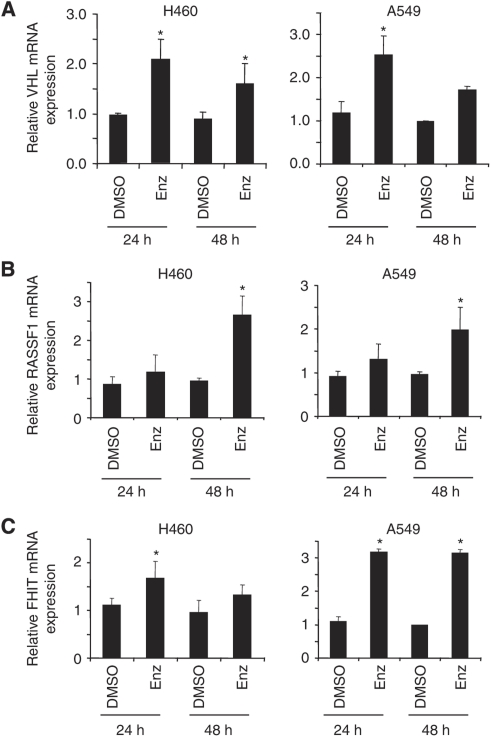
Enzastaurin induces the expression of tumour-suppressor genes. Real-time PCR quantification of (**A**) *VHL*, (**B**) *RASSF1*, and (**C**) *FHIT* mRNA in H460 and A549 cells after 24–48 h of DMSO or Enz (IC_50_) treatment. Relative gene expression was normalised against *β*-Actin mRNA. ^*^P<0.05.

**Figure 3:**
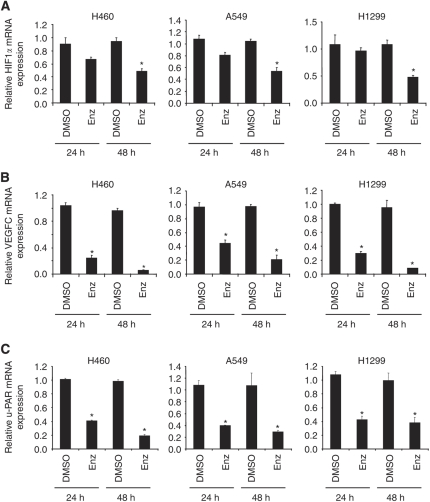
Effect of enzastaurin on the expression of pro-invasive genes. Real-time PCR quantification of (**A**) *HIF1α*, (**B**) *VEGFC*, and (**C**) *u-PAR* mRNA in H460, A549, and H1299 cells after 24–48 h of DMSO or Enz (IC_50_) treatment. Relative gene expression was normalised against *β*-Actin mRNA. ^*^P<0.05.

To analyse changes in gene expression after Enz treatment, we applied Illumina Human Sentrix-8 BeadChip assays. A549 cells were either treated with DMSO, or with Enz, for 24 or 48 h at the same concentration as used for inhibiting invasion. Total RNA was isolated, labelled, and hybridised to the arrays. After 24 h, 1255 genes (616 up, 639 down), and after 48 h, 2711 genes (1477 up, 1234 down) were significantly (⩾1.5-fold) deregulated after Enz treatment (Supplementary Tables 2 and 3), respectively. Comparing our microarray results with data present in the literature, we found that a considerable number of genes that have been described to be either up- or downregulated specifically in NSCLC in previous publications were significantly altered upon Enz treatment (Table 1). These genes include genes regulating cell proliferation, structure, adhesion, migration, invasion, or angiogenesis, or are cell-cycle regulators. We found that all of these genes as described in Table 1 were inversely regulated upon Enz treatment in A549 cells as compared to their gene expression status reported in previous publications on NSCLC for the untreated situation. Interestingly, three tumour suppressors (*FHIT*, *RASSF1*, and *VHL*), which have been reported to be downregulated in NSCLC (Wikman *et al*, 2002; Hayes *et al*, 2006), were upregulated upon Enz treatment, which was validated by RT–PCR (Figure 2A–C) at the mRNA level. Also, *HIF1α*, *VEGFC*, and *u-PAR* (Chen *et al*, 2001; Werle *et al*, 2004; Hayes *et al*, 2006), genes that are known to be key regulators of various aspects of carcinogenesis, invasion, and metastasis, were significantly downregulated after Enz treatment. The expression of these genes was validated in NSCLC cell lines through real-time PCR, which confirmed their cell line-independent regulation upon Enz treatment (Figure 3A–C). Taken together, Enz negatively regulates essential genes that are reportedly relevant for NSCLC, induces the expression of three NSCLC-related tumour-suppressors, and inhibits three essential genes of invasion and metastasis at the mRNA level.

### Enzastaurin inhibits u-PAR gene expression in NSCLC cells

**Figure 4:**
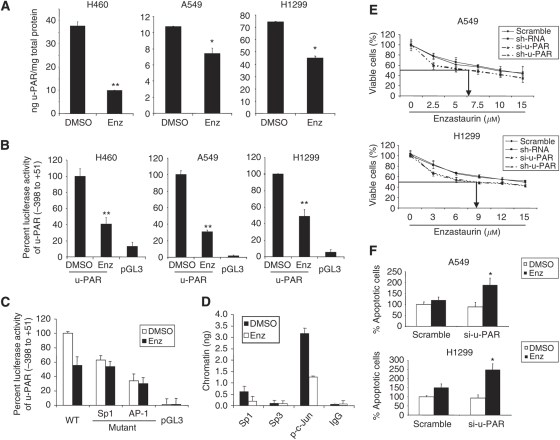
Enzastaurin inhibits u-PAR promoter activity and u-PAR protein expression. (**A**) Total amounts of u-PAR protein as evaluated by ELISA as described in the Materials and Methods section. Each value represents the average of triplicate measurements. (**B**) Luciferase activity assay for H460, A549, and H1299 cells. Data are presented as the mean±s.d. of three independent experiments performed in quadruplicate. ^*^P<0.05. (**C**) Luciferase activity assay of A549 cells transfected with wild-type (wt) u-PAR promoter, and Sp/AP1 binding site mutated reporter constructs. Values represent the mean of three independent experiments performed in triplicate; bars, s.d. (**D**) Real-time PCR quantification of u-PAR promoter binding after chromatin immunoprecipitation (ChIP) with Sp1, Sp3, p-c-Jun, or IgG antibodies in the presence, or absence, of Enz (24 h). (**E** and **F**) Cell viability (MTT) and apoptosis assay. At 24 h after transfection with si-u-PAR/sh-u-PAR, cells were treated with Enz for 48 h and the results compared to DMSO-treated controls for MTT assay, represented as percentage and for apoptosis cell were screened by flow cytometry and percent of apoptotic cells were calculated against to DMSO-treated cells.

The u-PAR has been reported to be one of the major metastasis-related genes being overexpressed in many cancer types, and also in NSCLC (Beer *et al*, 2002; Werle *et al*, 2004). Previous reports including our own have shown that an overexpression of this receptor is associated with a high invasive and metastatic capacity of NSCLC, and also EGF-induced, NSCLC cells, among other cancer types (Nikolova *et al*, 2009). Therefore, we decided to specifically analyse a putative function of the u-PAR in Enz-regulated invasion, and molecular mediators addressed by Enz to regulate u-PAR in NSCLC. ELISA showed a significant reduction of u-PAR protein after Enz treatment in H460 (*P*=0.0021), A549 (*P*=0.02), and H1299 (*P*=0.0022) cells as compared to the DMSO-treated samples (Figure 4A), in addition to the downregulation of u-PAR mRNA observed in the same cell lines in the previous section. It has been well reported that u-PAR gene expression in malignant cells is largely because of the transcriptional regulation of the gene (Allgayer *et al*, 1999; Laufs *et al*, 2006; Leupold *et al*, 2007). Specifically, it has been reported that the u-PAR promoter, to a major part, is being regulated by transcription factors of the AP-1 and Sp family (Allgayer *et al*, 1999; Laufs *et al*, 2006; Leupold *et al*, 2007). Correspondingly, we found that Enz treatment in A549 and H1299 cells significantly downregulated the expression of Sp1-, Sp3-, and c-Jun-transcription factors (*P*<0.05; Supplementary Figure 2), which have been reported previously to be major regulators of u-PAR promoter activity. In luciferase assays of H460, A549, and H1299 cells transfected with a reporter plasmid driven by the −398/+51 upstream region of the u-PAR gene, we found a significant reduction in u-PAR wild-type promoter activity after Enz treatment (*P*<0.008) (Figure 4B). Moreover, site-directed mutagenesis abolishing the binding of either Sp- or AP-1-family members to their respective binding sites within the u-PAR promoter abolished the ability of Enz to induce a further reduction of promoter activity of the respective construct (Figure 4C). This was paralleled by a specific reduction of the binding of either Sp1, Sp3, or AP-1-family members to their specific *cis* elements within the u-PAR promoter in EMSA analysis (Supplementary Figure 3b and c), which could also be observed for the situation of EGF-induced A549 cells (Supplementary Figure 3a). Finally, to show that Enz is able to regulate transcription factor binding to the natural u-PAR promoter, we performed ChIP analysis for the endogenous u-PAR upstream region. Chromatin immunoprecipitation revealed significantly less binding of especially Sp1, and phospho-c-Jun, to the endogenous u-PAR promoter after Enz treatment (Figure 4D, Supplementary Figure 3d). Taken together, data suggest that Enz inhibits the expression of the u-PAR, at least to a relevant part by suppressing u-PAR promoter activity, by downregulation of the binding of especially Sp1, and c-Jun out of the AP-1 family.

### u-PAR knockdown re-sensitises NSCLC cells and induces apoptosis to Enz

A427, A459, and H1299 are relatively resistant to Enz treatment as compared to other NSCLC cell lines. This observation supported the notion that endogenous u-PAR gene expression might be associated with resistance to Enz. Therefore, we conducted specific knockdown experiments with an si-RNA/sh-RNA against endogenous u-PAR, the efficient knockdown of endogenous u-PAR being confirmed with u-PAR-ELISA (Supplementary Figure 4a and b). We observed that A549 and H1299 cells treated with si-u-PAR/sh-u-PAR were significantly re-sensitised to Enz (A549/IC_50_⩽7.0 *μ*M; H1299/IC_50_⩽9.0 *μ*M) when compared to scrambled control-transfected cells (A549/IC_50_=10 *μ*M; H1299/IC_50_=12 *μ*M) (Figure 4E). Similarly, viable cells were counted after Enz treatment either with scrambled/sh-RNA or with si-u-PAR/sh-u-PAR knockdown, which confirmed the IC_50_ concentrations determined by MTT assay (data not shown). Furthermore, u-PAR knockdown cells significantly induced apoptosis when compared with control cells after 48 h of Enz treatment (Figure 4F), and also showed a further decrease in u-PAR protein levels after Enz treatment (Supplementary Figure 4a and b). These data suggest that u-PAR might be one molecular parameter indicating Enz resistance of NSCLC cells.

### Enzastaurin reduces distant metastasis of NSCLC cells *in vivo*

**Figure 5:**
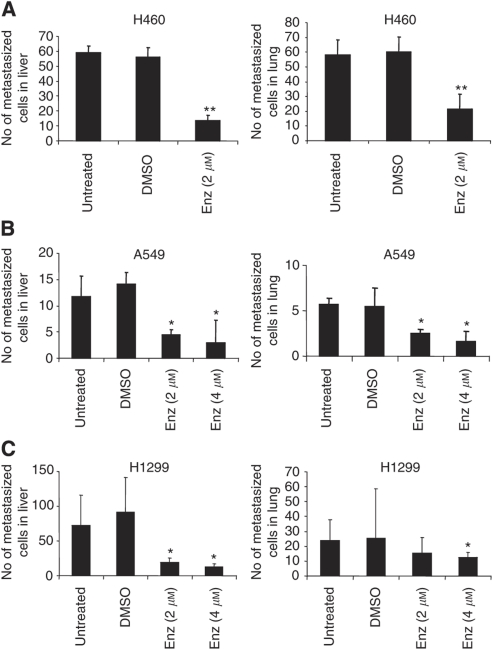
Enzastaurin inhibits NSCLC metastasis *in vivo*. (**A**–**C**) Number of metastasising cells in the livers and lungs of chicken embryos treated intravenously with different concentrations of Enz (2 or 4 *μ*M) or DMSO, respectively. Human Alu sequences were amplified and quantified by real-time PCR. ^**^P<0.001; ^*^P⩽0.05.

Our previous results suggested that Enz reduces migration and invasion *in vitro*. To strengthen these observations, we used the chicken embryo metastasis assay (CAM) (Leupold *et al*, 2007) to check the ability of Enz to inhibit the formation of distant metastasis *in vivo*. Towards this end, H460, A549, and H1299 cells were inoculated on the upper CAM of 10-day-old chicken embryos. On day 12 and 14, the embryos were treated intravenously with 2 or 4 *μ*M of Enz, or equal concentrations of DMSO, respectively. On day 16, the embryos were killed, lungs and livers harvested for DNA, and the number of cells metastasised into livers and lungs were measured with a TaqMan-based PCR amplifying human alu-sequences on the chicken background (Leupold *et al*, 2007). As a result, we found that Enz was able to reduce the formation of distant metastasis significantly in H460 (liver *P*=0.001, lungs *P*=0.003), A549 (liver *P*=0.037, lungs *P*⩽0.01), and H1299 (liver *P*=0.02, lungs *P*=0.05) cells when compared with their respective DMSO controls (Figure 5A–C). These data suggest that Enz, besides migration and invasion, inhibits *in vivo* metastasis of three NSCLC cell lines.

## Discussion

This is the study to show that Enz inhibits migration, invasion, and *in vivo* metastasis in NSCLC, and to explore first mechanisms contributing to Enz-induced inhibition of migration, invasion, and metastasis-related processes in NSCLC. We show that Enz transcriptionally inhibits u-PAR gene expression, downregulates the tumour progression-related molecules HIF1*α* and VEGFC, and also upregulates the tumour-suppressor genes *VHL*, *RASSF1*, and *FHIT*. Furthermore, our findings suggest that an si-u-PAR/sh-u-PAR knockdown sensitises resistant NSCLC cells to Enz and induces apoptosis. These findings suggest that Enz treatment in NSCLC patients might contribute to enhancing their survival by inhibiting genes of tumour progression and by inducing tumour-suppressor genes.

Enzastaurin was initially developed as a specific inhibitor of PKC*β*, also inhibiting the other PKC isoforms (*δ*, *α*, *ε*, and *γ*), PI3K/AKT, GSK3*β*, and S6K (Faul *et al*, 2003; Graff *et al*, 2005; Tekle *et al*, 2008; Faoro *et al*, 2009; Willey *et al*, 2009; Giovannetti *et al*, 2010). In agreement with previous studies, our initial observations in six NSCLC cell lines have shown that Enz inhibits cell growth and activation of PKC, p-42/44, AKT, and GSK3 *β* (Faul *et al*, 2003; Graff *et al*, 2005). Furthermore, we in addition show that it inhibits the EGF-stimulated activation of these kinases. In addition to initial findings in cultured hepatocellular carcinoma, we now show that Enz inhibits the *in vitro* migration and invasion of NSCLC cells (Guo *et al*, 2009), and that EGF-induced invasion is countered by Enz, which is a major pathway in NSCLC progression (Nikolova *et al*, 2009). Correspondingly, PKC isoforms have been shown to be activated in NSCLC cells, and to correlate with tumour progression in different cancers (Shipp *et al*, 2002; Clark *et al*, 2003; Ali *et al*, 2009; Gokmen-Polar *et al*, 2001). As further fundamental pathways, the MAPK/ERK and PI3K/AKT signalling axis have been reported to be activated upon PKC activation, which leads to cell proliferation and anti-apoptosis (Balendran *et al*, 2000; Goekjian and Jirousek, 2001). By inhibiting the activation PKC/other Ser/Thr kinases such as AKT, p-42/44, and GSK3*β* in NSCLC cells, in our gene expression array we now found that Enz indeed significantly downregulates a number of genes that are involved in different cellular functions such as cell proliferation, anti-apoptosis, but also cell adhesion, migration, invasion, and angiogenesis, which are known to be upregulated in the NSCLC (Bhattacharjee *et al*, 2001; Chen *et al*, 2001; Hellmann *et al*, 2001; Beer *et al*, 2002; Wikman *et al*, 2002; Borczuk *et al*, 2003; Diederichs *et al*, 2004; Werle *et al*, 2004; Hayes *et al*, 2006). Moreover, remarkably, it significantly upregulates the tumour-suppressor genes (*FHIT*, *RASSF1*, and *VHL*) that are known to be downregulated in NSCLC (Zochbauer-Muller *et al*, 2002; Hayes *et al*, 2006). It has been reported that the expression of *RASSF1* and *FHIT* is inhibited in NSCLC tumours, their downregulation interestingly also having been shown to be associated with promoter hypermethylation (Zochbauer-Muller *et al*, 2002). These tumour-suppressor genes have been implicated as either a pivotal gatekeeper of cell-cycle progression (Dammann *et al*, 2000) or a molecule able to reverse the malignant phenotype and to induce tumour cell apoptosis (Ji *et al*, 1999), respectively.

Hypoxia-inducible factor 1*α*, one of the essential targets of Enz validated in this study, is getting stabilised in hypoxic conditions, inactivates tumour-suppressors (p53, PTEN), activates several oncogenic pathways (Src, HER/2, H-ras) (Zhang *et al*, 2007), and transactivates VEGF through hypoxia response elements, this being central to the initiation of pro-angiogenic signalling and neovascular formation (Forsythe *et al*, 1996). Intracellular HIF1*α* concentrations are tightly regulated by the von Hippel–Lindau tumour-suppressor protein, a product of the third tumour-suppressor gene upregulated by Enz in our study, and a component of the E3 ubiquitin ligase system (Karhausen *et al*, 2005). It is an interesting observation of this study that VHL is getting upregulated, and its targeted molecule HIF1*α*, in addition to VEGF that is transactivated by HIF1*α*, is getting downregulated upon Enz treatment. Correspondingly, previous reports have suggested these genes to be inversely regulated in NSCLC (Forsythe *et al*, 1996; Wang *et al*, 2009). In addition, u-PA and u-PAR, which are upregulated in NSCLC (Werle *et al*, 2004; Beyer *et al*, 2006), are getting downregulated upon Enz treatment in our study. Interestingly, all three of these tumour progression-related genes, *u-PAR*, *HIF1α*, and *VEGFC*, are either activated by the downstream axis of, or transcriptionally regulated at least in part, by the MAPK/ERK and/or PI3K/AKT pathways (Jo *et al*, 2003; Tsai *et al*, 2003; Phillips *et al*, 2005; Skurk *et al*, 2005), which are inhibited by Enz treatment (Faul *et al*, 2003; Graff *et al*, 2005).

The u-PAR is an essential molecule in the context of proteolysis, migration, invasion, and metastasis (Jo *et al*, 2003; Dass *et al*, 2008), and its function and transcriptional regulation are well studied (Laufs *et al*, 2006). Interestingly, in our study, Enz inhibited u-PAR promoter activity by inhibiting the gene expression and binding of Sp1, Sp3, and c-Jun(AP-1) to the u-PAR-promoter *in vitro* and *in vivo* (ChIP). In the context of suppressing u-PAR gene expression through Enz, it was an interesting initial observation in our study that the knockdown of u-PAR might be able to resensitise relatively resistant NSCLC cells and induces apoptosis after Enz treatment (Querfeld *et al*, 2006; Lee *et al*, 2008; Morgillo *et al*, 2008). This finding emphasises the general importance of defining critical molecular markers that are associated with a potential resistance towards novel targeted therapy compounds, because otherwise it might lead to the treatment of patients that, by their molecular condition, are unable to respond to the novel drug. In fact, this might be one of the explanations as to why initial clinical trials with Enz that were not associated with parallel translational molecular studies at the resected tumour tissue of treated patients, led to initial contradictory results (Casey *et al*, 2009; Richards *et al*, 2009; Kreisl *et al*, 2010; Wick *et al*, 2010). However, with our results we might give an additional encouragement for the initiation of more tailored-therapy clinical studies, in which putatively essential molecular markers associated with response or resistance are being measured before the decision for a certain combination of therapies in the individual patient.

In our paper, we show that Enz inhibits *in vivo* distant metastasis to liver and lungs in the CAM assay. Apart from its pro-apoptotic, growth inhibitory, and anti-angiogenic properties (Keyes *et al*, 2002; Graff *et al*, 2005; Querfeld *et al*, 2006; Willey *et al*, 2009), this could be, at least in part, due to downregulating tumour progression and invasion-related genes (*u-PAR*, *HIF1α*, and *VEGFC*) and upregulating NSCLC tumour-related suppressor genes (*FHIT*, *RASSF1*, and *VHL*) by Enz. We consider it unlikely that a main mechanism of metastasis inhibition by Enz can be explained by anti-angiogenesis, because we observed that DMSO- or Enz-dipped plugs did not show any significant difference in their effect on the formation of embryonic vasculature of the upper CAM (data not shown). However, certainly, an anti-angiogenic component of Enz in the context of anti-metastatic action in our model cannot be ruled out completely.

Taken together, this is the first comprehensive analysis to investigate differentially regulated genes in NSCLC cells upon Enz treatment, and to implicate novel Enz targets in the context of migration, invasion, and distant metastasis in NSCLC. Our results suggest that Enz has a major role in downregulating the u-PAR through Sp1/Sp3 and c-Jun(AP-1), besides HIF1*α* and VEGFC that are implicated in tumour growth and metastasis, in parallel to upregulating tumour-suppressors relevant for NSCLC. Furthermore, the knockdown of u-PAR re-sensitises NSCLC cells to Enz treatment. These findings give important information for ongoing clinical trials with Enz in NSCLC patients, and further studies investigating resected tumour tissue or blood samples of Enz treated patients for, for example, u-PAR or serum-soluble u-PAR (Henic *et al*, 2008), as a potentially clinically relevant marker of therapy resistance are clearly encouraged.

## Figures and Tables

**Table 1:** Enz inversely regulates relevant genes reported for NSCLC

**Gene symbol**	**Accession no.**	**Fold change (Enz 24/48 h *vs* DMSO)**	**Reference**
*Cell cycle, proliferation, migration, and angiogenesis*			
*CCND1*	NM_053056	−5.1	Borczuk *et al*, 2003
*S100P*	NM_005980	−2.3	Beer *et al*, 2002
*MCM 2*	NM_004526	−2.1	Wikman *et al*, 2002
*CEACAM1*	NM_0010249	−4.2	Bhattacharjee *et al*, 2001
*PRSS3*	NM_002771	−2.4	Diederichs *et al*, 2004
*PLAU*	NM_002658	−2.2	Chen *et al*, 2001
*PLAUR*	NM_1005376	−1.8	Werle *et al*, 2004; Hayes *et al*, 2006
*ERBB3*	NM_001982	−7.4	Beer *et al*, 2002
*FGFR3*	NM_000142	−4.7	Hellmann *et al*, 2001
*HIF1α*	NM_001530	−2.4	Hayes *et al*, 2006
*IGFBP3*	NM_0010133	−13	Beer *et al*, 2002; Hellmann *et al*, 2001; Wikman *et al*, 2002
*GADD45A*	NM_001924	+9.6	Hellmann *et al*, 2001
			
*Cell adhesion, cell structure, and signal transduction*			
*KRT7*	NM_005556	−1.7	Beer *et al*, 2002
*KRT8*	NM_002264	−2.6	Wikman *et al*, 2002
*KRT18*	NM_000224	−2.1	Wikman *et al*, 2002
*MUC1*	NM_0010180	−4.1	Borczuk *et al*, 2003
*VEGFC*	NM_005429	−2.4	Borczuk *et al*, 2003
*FGG*	NM_000509	−3.5	Diederichs *et al*, 2004
*SFTPD*	NM_003019	−2.4	Borczuk *et al*, 2003
*ITGB4BP*	NM_181466	+2.2	Hellmann *et al*, 2001
*PLEC1*	NM_201384	+2.4	Hellmann *et al*, 2001
			
*Tumor suppressor*			
*FHIT*	NM_002012	+5.0	Zochbauer-Muller *et al*, 2002
*RASSF1*	NM_007182	+2.0	Zochbauer-Muller *et al*, 2002
*VHL*	NM_000551	+1.7	Hayes *et al*, 2006
			
*Other genes*			
*TOP 2A*	NM_001067	−3.8	Hellmann *et al*, 2001; Wikman *et al*, 2002
*SLC1A5*	NM_005628	+2.1	Beer *et al*, 2002
*ABCC3*	NM_020037	−5.0	Beer *et al*, 2002
