# Supplementary material for: Enzastaurin inhibits invasion and metastasis in lung cancer by diverse molecules
Source: Br J Cancer. 2010 Aug 24;103(6):802–11. doi: 10.1038/sj.bjc.6605818 (PMC2966618; doi:10.1038/sj.bjc.6605818)
Supplement: Supplementary Figure 1 [file 6605818x1.ppt]

## Slide 1
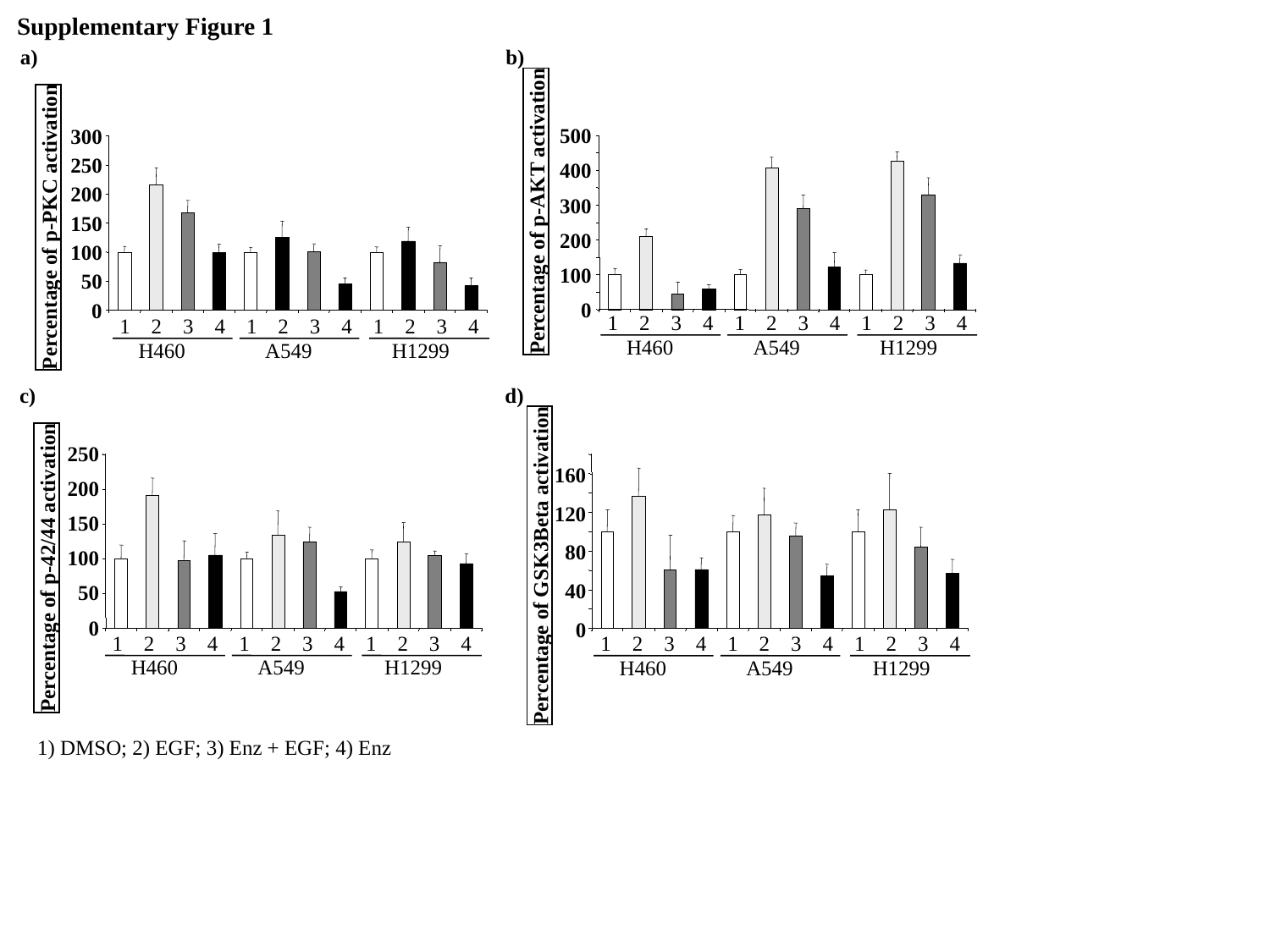

Supplementary Figure 1
a)
b)
500
300
250
400
200
300
Percentage of p-AKT activation
150
Percentage of p-PKC activation
200
100
100
50
0
0
1 2 3 4 1 2 3 4 1 2 3 4
1 2 3 4 1 2 3 4 1 2 3 4
H460
A549
H1299
H460
A549
H1299
c)
d)
250
160
200
120
150
80
100
Percentage of GSK3Beta activation
Percentage of p-42/44 activation
40
50
0
0
1 2 3 4 1 2 3 4 1 2 3 4
1 2 3 4 1 2 3 4 1 2 3 4
H460
A549
H1299
H460
A549
H1299
1) DMSO; 2) EGF; 3) Enz + EGF; 4) Enz
