# Supplementary material for: Enzastaurin inhibits invasion and metastasis in lung cancer by diverse molecules
Source: Br J Cancer. 2010 Aug 24;103(6):802–11. doi: 10.1038/sj.bjc.6605818 (PMC2966618; doi:10.1038/sj.bjc.6605818)
Supplement: Supplementary Figure 2 [file 6605818x2.ppt]

## Slide 1
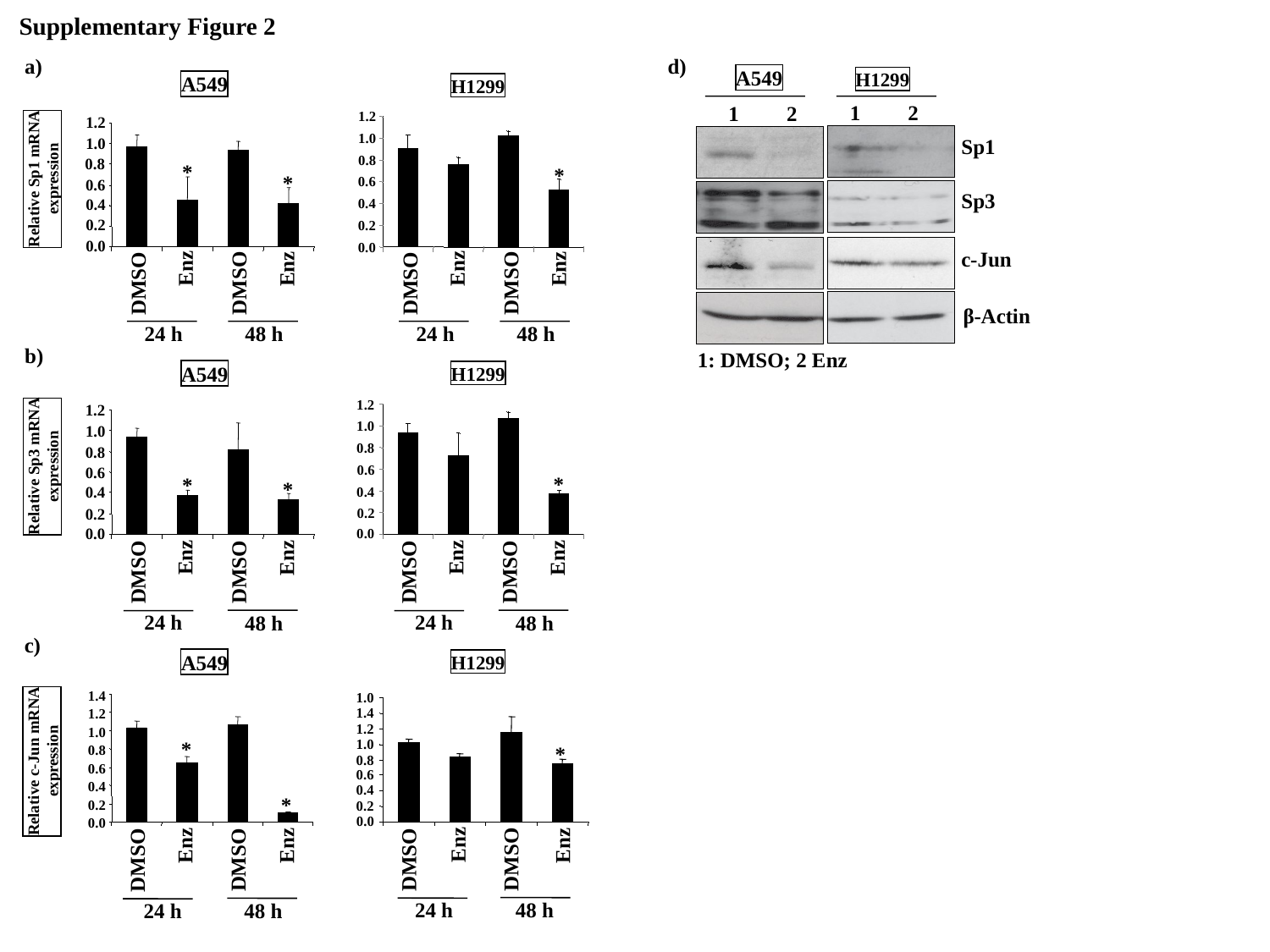

Supplementary Figure 2
a)
d)
A549
H1299
A549
H1299
 1 2
 1 2
1.2
1.2
Sp1
1.0
1.0
0.8
*
0.8
*
Relative Sp1 mRNA
expression
*
0.6
0.6
Sp3
0.4
0.4
0.2
0.2
0.0
0.0
c-Jun
Enz
Enz
Enz
Enz
DMSO
DMSO
DMSO
DMSO
β-Actin
24 h
24 h
48 h
48 h
b)
 1: DMSO; 2 Enz
A549
H1299
1.2
1.2
1.0
1.0
0.8
0.8
Relative Sp3 mRNA
expression
0.6
0.6
*
*
*
0.4
0.4
0.2
0.2
0.0
0.0
Enz
Enz
Enz
Enz
DMSO
DMSO
DMSO
DMSO
24 h
24 h
48 h
48 h
c)
A549
H1299
1.4
1.2
1.0
0.8
0.6
0.4
0.2
0.0
Enz
Enz
DMSO
DMSO
24 h
48 h
1.0
1.4
1.2
1.0
0.8
0.6
0.4
0.2
0.0
Enz
Enz
DMSO
DMSO
24 h
48 h
*
*
Relative c-Jun mRNA
 expression
*
