# Supplementary material for: Enzastaurin inhibits invasion and metastasis in lung cancer by diverse molecules
Source: Br J Cancer. 2010 Aug 24;103(6):802–11. doi: 10.1038/sj.bjc.6605818 (PMC2966618; doi:10.1038/sj.bjc.6605818)
Supplement: Supplementary Figure 3 [file 6605818x3.ppt]

## Slide 1
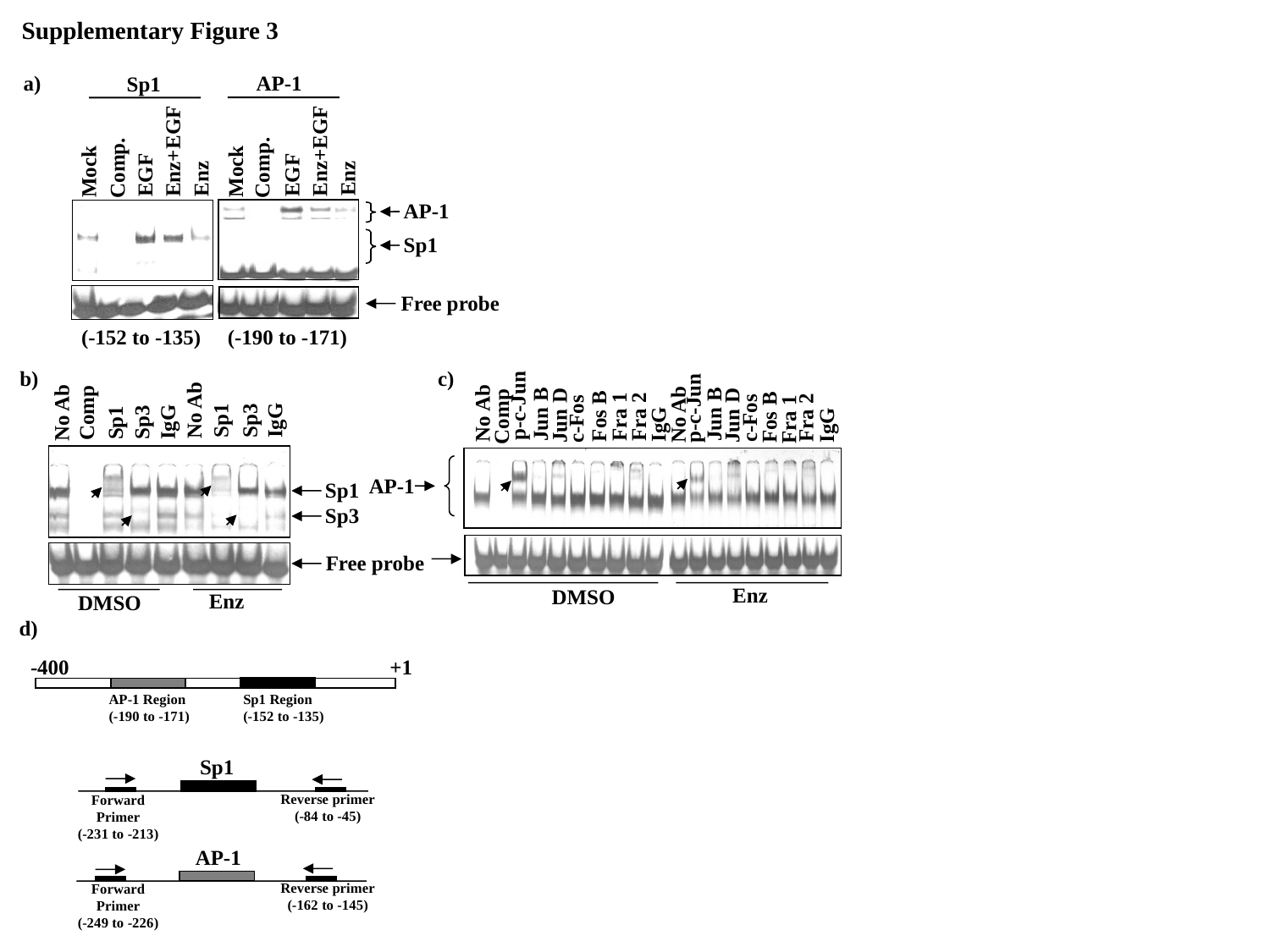

Supplementary Figure 3
a)
AP-1
Sp1
Enz+EGF
Enz+EGF
Enz
Enz
EGF
EGF
Comp.
Comp.
Mock
Mock
AP-1
Sp1
Free probe
(-190 to -171)
(-152 to -135)
c)
b)
Comp
No Ab
No Ab
Fos B
p-c-Jun
c-Fos
Jun D
Jun D
c-Fos
Fos B
p-c-Jun
 Fra 2
 Fra 1
 IgG
 Fra 2
Sp1
Sp3
IgG
Jun B
Jun B
 IgG
No Ab
 Fra 1
Sp3
IgG
No Ab
Sp1
Comp
AP-1
Sp1
Sp3
Free probe
Enz
DMSO
Enz
DMSO
d)
-400
+1
AP-1 Region
(-190 to -171)
Sp1 Region
(-152 to -135)
Sp1
Reverse primer
(-84 to -45)
Forward Primer
(-231 to -213)
AP-1
Reverse primer
(-162 to -145)
Forward Primer
(-249 to -226)
