# Supplementary material for: Enzastaurin inhibits invasion and metastasis in lung cancer by diverse molecules
Source: Br J Cancer. 2010 Aug 24;103(6):802–11. doi: 10.1038/sj.bjc.6605818 (PMC2966618; doi:10.1038/sj.bjc.6605818)
Supplement: Supplementary Figure 4 [file 6605818x4.ppt]

## Slide 1
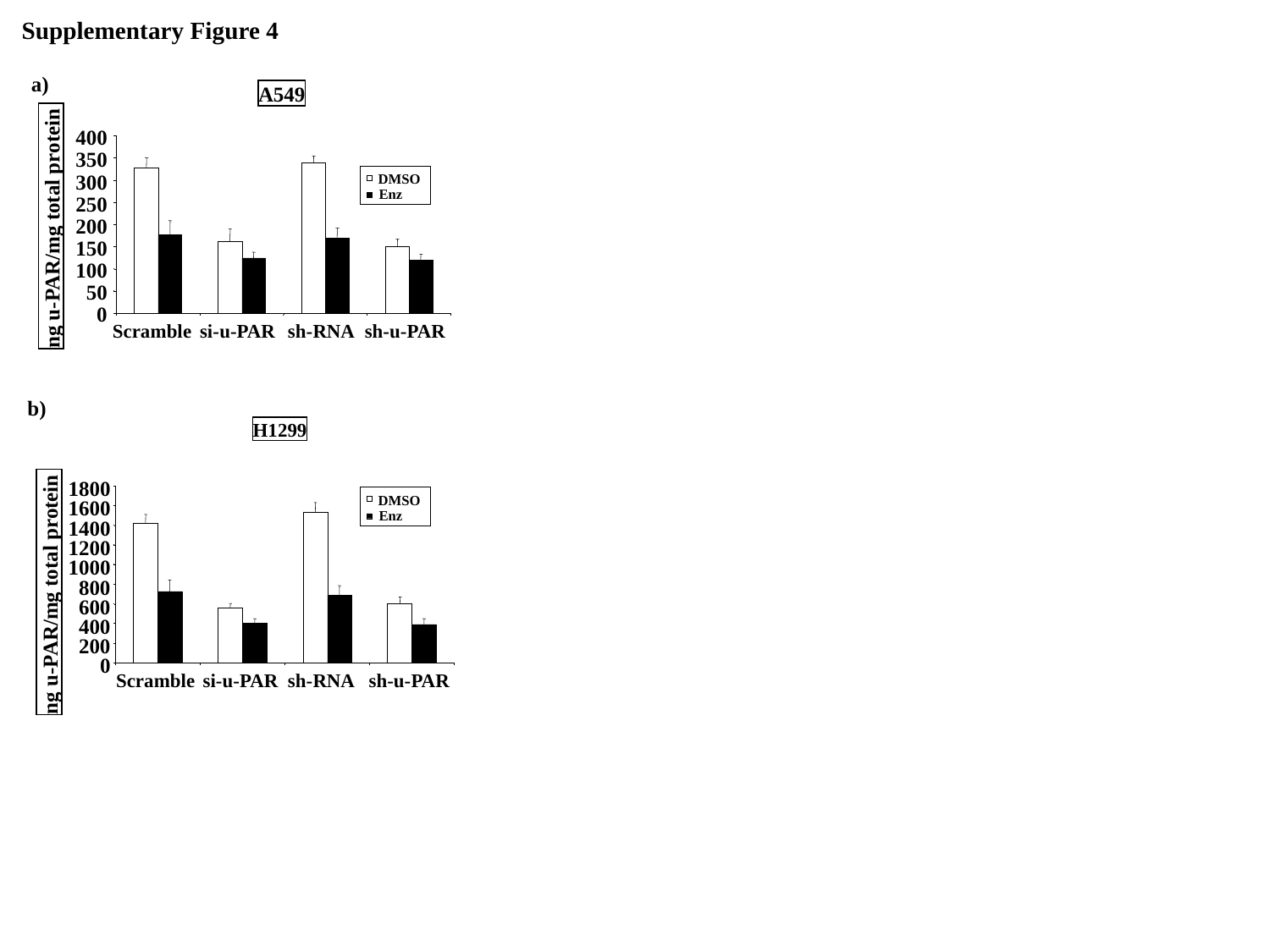

Supplementary Figure 4
a)
A549
400
350
DMSO
Enz
300
250
200
ng u-PAR/mg total protein
150
100
50
0
Scramble
si-u-PAR
sh-RNA
sh-u-PAR
b)
H1299
1800
DMSO
Enz
1600
1400
1200
1000
800
ng u-PAR/mg total protein
600
400
200
0
Scramble
si-u-PAR
sh-RNA
sh-u-PAR
