# Supplementary material for: Enzastaurin inhibits invasion and metastasis in lung cancer by diverse molecules
Source: Br J Cancer. 2010 Aug 24;103(6):802–11. doi: 10.1038/sj.bjc.6605818 (PMC2966618; doi:10.1038/sj.bjc.6605818)
Supplement: Supplementary Table 1 [file 6605818x5.doc]

**Supplementary Table 1**

# Oligos used for SYBR Green Taq man assay

| **S.No** | **Gene Name** | **Sequence (5’ --- 3’)** |
| --- | --- | --- |
| 1 | HIF1α For | CCAGCAGACTCAAATACAAGAACC |
| 2 | HIF1α Rev | TGTATGTGGGTAGGAGATGGAGAT |
| 3 | VEGFC For | ACCAAACAAGGAGCTGGATG |
| 4 | VEGFC Rev | ATTTCTGGGGCAGGTTCTTT |
| 5 | VHL_For | TCTCAATGTTGACGGACAGC |
| 6 | VHL_Ror | ACATTTGGGTGGGTCTTCCAG |
| 7 | RASSF1_For | AAGTTCACCTGCCACTACCG |
| 8 | RASSF1_Rev | CGGTCCTTGTTCAAGCTCAT |
| 9 | FHIT_For | TGGCCAACATCTCATCAAG |
| 10 | FHIT_Rev | ACAAATCGGCCACTTCATC |
| 11 | Sp1 For | CTGGTCATACTGTGGGAAACGC |
| 12 | Sp1 Rev | TGTTGGCAAGACGGGCAATG |
| 13 | Sp3 For | GGAAAAAGACTTCGGAGGGTAGC |
| 14 | Sp3 Rev | GCAAGGTGGTCACTTCTCATAAAGC |
| 15 | c-Jun For | ACGCAAACCTCAGCAACTTC |
| 16 | c-Jun Rev | CACTGTCTGAGGCTCCTCCT |
| 17 | β-Actin For | TCCCTGGAGAAGAGCTACG |
| 18 | β-Actin Rev | GTAGTTTCGTGGATGCCACA |
