# Supplementary material for: Enzastaurin inhibits invasion and metastasis in lung cancer by diverse molecules
Source: Br J Cancer. 2010 Aug 24;103(6):802–11. doi: 10.1038/sj.bjc.6605818 (PMC2966618; doi:10.1038/sj.bjc.6605818)
Supplement: Supplementary Table 2 [file 6605818x6.doc]

**Supplementary Table 2**

**Significantly (> 1.5 fold) deregulated genes after 24 hours of Enz-treatment**

| **S. No** | **Symbol** | **Fold Change** | **Genbank** | **Probe_ID (ILL)** |
| --- | --- | --- | --- | --- |
| 1 | **TM4SF20** | **-19,7** | NM_024795 | ILMN_1775830 |
| 2 | **BNIP3L** | **-8,7** | NM_004331 | ILMN_1718961 |
| 3 | **FGB** | **-6,7** | NM_005141 | ILMN_1678049 |
| 4 | **JUP** | **-6,2** | NM_021991 | ILMN_1733811 |
| 5 | **RAP1GA1** | **-6,2** | NM_002885 | ILMN_1776519 |
| 6 | **RNF19** | **-6,0** | NM_015435 | ILMN_1673875 |
| 7 | **NUSAP1** | **-5,8** | NM_016359 | ILMN_1726720 |
| 8 | **FGA** | **-5,7** | NM_021871 | ILMN_1656487 |
| 9 | **ANGPTL4** | **-5,7** | NM_139314 | ILMN_1707727 |
| 10 | **TF** | **-5,6** | NM_001063 | ILMN_1768425 |
| 11 | **KLHDC2** | **-5,5** | NM_014315 | ILMN_1741204 |
| 12 | **CD55** | **-5,4** | NM_000574 | ILMN_1800540 |
| 13 | **CDH19** | **-5,2** | NM_021153 | ILMN_1787825 |
| 14 | **TFPI** | **-5,2** | NM_001032281 | ILMN_1662619 |
| 15 | **ERRFI1** | **-5,2** | NM_018948 | ILMN_1665510 |
| 16 | **ULK1** | **-5,0** | NM_003565 | ILMN_1735052 |
| 17 | **CP** | **-5,0** | NM_000096 | ILMN_1739608 |
| 18 | **ELF3** | **-4,9** | NM_004433 | ILMN_1769201 |
| 19 | **GABARAPL1** | **-4,9** | NM_031412 | ILMN_1667846 |
| 20 | **HBP1** | **-4,8** | NM_012257 | ILMN_1685415 |
| 21 | **MTMR11** | **-4,7** | NM_181873 | ILMN_1769299 |
| 22 | **SCNN1A** | **-4,7** | NM_001038 | ILMN_1713995 |
| 23 | **HLA-DMB** | **-4,6** | NM_002118 | ILMN_1761733 |
| 24 | **HSPA2** | **-4,6** | NM_021979 | ILMN_1766499 |
| 25 | **LOC201895** | **-4,5** | NM_174921 | ILMN_1713892 |
| 26 | **B4GALT4** | **-4,5** | NM_003778 | ILMN_1661500 |
| 27 | **LGR4** | **-4,4** | NM_018490 | ILMN_1686895 |
| 28 | **CNTN1** | **-4,4** | NM_001843 | ILMN_1728853 |
| 29 | **BTG1** | **-4,3** | NM_001731 | ILMN_1775743 |
| 30 | **CCND3** | **-4,3** | NM_001760 | ILMN_1668721 |
| 31 | **FSTL1** | **-4,2** | NM_007085 | ILMN_1715426 |
| 32 | **CEACAM6** | **-4,0** | NM_002483 | ILMN_1712522 |
| 33 | **PTPNS1** | **-4,0** | NM_080792 | ILMN_1758146 |
| 34 | **RARB** | **-3,9** | NM_000965 | ILMN_1699723 |
| 35 | **TM4SF4** | **-3,9** | NM_004617 | ILMN_1792404 |
| 36 | **SGK** | **-3,9** | NM_005627 | ILMN_1702487 |
| 37 | **TOP2A** | **-3,8** | NM_001067 | ILMN_1686097 |
| 38 | **FGFR3** | **-3,8** | NM_000142 | ILMN_1723123 |
| 39 | **NDRG1** | **-3,8** | NM_006096 | ILMN_1809931 |
| 40 | **DHRS3** | **-3,8** | NM_004753 | ILMN_1752478 |
| 41 | **LRP11** | **-3,7** | NM_032832 | ILMN_1676197 |
| 42 | **FYB** | **-3,7** | NM_199335 | ILMN_1796537 |
| 43 | **RICTOR** | **-3,7** | NM_152756 | ILMN_1727171 |
| 44 | **DKFZP686A01247** | **-3,7** | NM_014988 | ILMN_1664138 |
| 45 | **SPSB3** | **-3,6** | NM_080861 | ILMN_1682864 |
| 46 | **CDC2L6** | **-3,6** | NM_015076 | ILMN_1676891 |
| 47 | **GALNT4** | **-3,6** | NM_003774 | ILMN_1739297 |
| 48 | **ALS2CR13** | **-3,5** | NM_173511 | ILMN_1739942 |
| 49 | **TM4SF1** | **-3,5** | NM_014220 | ILMN_1770338 |
| 50 | **DAPK1** | **-3,5** | NM_004938 | ILMN_1708340 |
| 51 | **AGR2** | **-3,5** | NM_006408 | ILMN_1814151 |
| 52 | **SLC29A4** | **-3,4** | NM_153247 | ILMN_1801377 |
| 53 | **UGDH** | **-3,4** | NM_003359 | ILMN_1729563 |
| 54 | **ARL6IP** | **-3,4** | NM_015161 | ILMN_1708416 |
| 55 | **ANLN** | **-3,4** | NM_018685 | ILMN_1739645 |
| 56 | **IQWD1** | **-3,3** | NM_018442 | ILMN_1670000 |
| 57 | **AKAP12** | **-3,3** | NM_005100 | ILMN_1684836 |
| 58 | **GAS2** | **-3,3** | NM_177553 | ILMN_1804569 |
| 59 | **ST3GAL5** | **-3,3** | NM_003896 | ILMN_1713496 |
| 60 | **CSPG2** | **-3,3** | NM_004385 | ILMN_1687301 |
| 61 | **FAM100B** | **-3,3** | NM_182565 | ILMN_1782778 |
| 62 | **GNG12** | **-3,2** | NM_018841 | ILMN_1673380 |
| 63 | **ANXA13** | **-3,2** | NM_001003954 | ILMN_1799243 |
| 64 | **GADD45B** | **-3,2** | NM_015675 | ILMN_1718977 |
| 65 | **DOK4** | **-3,2** | NM_018110 | ILMN_1774261 |
| 66 | **RRM2** | **-3,2** | NM_001034 | ILMN_1678669 |
| 67 | **DUSP1** | **-3,1** | NM_004417 | ILMN_1781285 |
| 68 | **TOPBP1** | **-3,1** | NM_007027 | ILMN_1684929 |
| 69 | **ERBB3** | **-3,1** | NM_001982 | ILMN_1751346 |
| 70 | **SLC39A10** | **-3,1** | NM_020342 | ILMN_1656129 |
| 71 | **PERP** | **-3,1** | NM_022121 | ILMN_1726161 |
| 72 | **ECT2** | **-3,1** | NM_018098 | ILMN_1719307 |
| 73 | **HOXA5** | **-3,1** | NM_019102 | ILMN_1753613 |
| 74 | **ARHGEF16** | **-3,1** | NM_014448 | ILMN_1669928 |
| 75 | **ITGAV** | **-3,1** | NM_002210 | ILMN_1706592 |
| 76 | **PPFIBP2** | **-3,1** | NM_003621 | ILMN_1675656 |
| 77 | **OPN3** | **-3,0** | NM_001030012 | ILMN_1716988 |
| 78 | **LANCL1** | **-3,0** | NM_006055 | ILMN_1703697 |
| 79 | **DPYSL2** | **-3,0** | NM_001386 | ILMN_1672503 |
| 80 | **PALM** | **-3,0** | NM_002579 | ILMN_1812031 |
| 81 | **RHBDF1** | **-3,0** | NM_022450 | ILMN_1808404 |
| 82 | **PTRF** | **-3,0** | NM_012232 | ILMN_1757552 |
| 83 | **ITGB1** | **-3,0** | NM_133376 | ILMN_1714820 |
| 84 | **VAV3** | **-3,0** | NM_006113 | ILMN_1657679 |
| 85 | **HSPA1A** | **-3,0** | NM_005345 | ILMN_1789074 |
| 86 | **PLS3** | **-3,0** | NM_005032 | ILMN_1785265 |
| 87 | **NPY1R** | **-3,0** | NM_000909 | ILMN_1799878 |
| 88 | **CXCL16** | **-3,0** | NM_022059 | ILMN_1728478 |
| 89 | **SLC24A6** | **-3,0** | NM_024959 | ILMN_1701655 |
| 90 | **CDC42EP4** | **-2,9** | NM_012121 | ILMN_1745223 |
| 91 | **IGFBP3** | **-2,9** | NM_001013398 | ILMN_1746085 |
| 92 | **SEPHS2** | **-2,9** | NM_012248 | ILMN_1687824 |
| 93 | **ACSM3** | **-2,9** | NM_005622 | ILMN_1771084 |
| 94 | **RB1CC1** | **-2,9** | NM_014781 | ILMN_1736796 |
| 95 | **ALDH3A2** | **-2,9** | NM_001031806 | ILMN_1794825 |
| 96 | **UHRF1** | **-2,9** | NM_013282 | ILMN_1786065 |
| 97 | **ARPC5** | **-2,9** | NM_005717 | ILMN_1768394 |
| 98 | **C1orf116** | **-2,9** | NM_023938 | ILMN_1706483 |
| 99 | **JAK1** | **-2,9** | NM_002227 | ILMN_1793384 |
| 100 | **DEK** | **-2,8** | NM_003472 | ILMN_1663061 |
| 101 | **EPS8** | **-2,8** | NM_004447 | ILMN_1733282 |
| 102 | **STAG2** | **-2,8** | NM_006603 | ILMN_1782609 |
| 103 | **BBX** | **-2,8** | NM_020235 | ILMN_1745415 |
| 104 | **RBL2** | **-2,8** | NM_005611 | ILMN_1756999 |
| 105 | **STAT1** | **-2,8** | NM_007315 | ILMN_1777325 |
| 106 | **ARRB1** | **-2,8** | NM_004041 | ILMN_1735218 |
| 107 | **ADD3** | **-2,8** | NM_016824 | ILMN_1814526 |
| 108 | **MAN2A1** | **-2,8** | NM_002372 | ILMN_1809402 |
| 109 | **TRPM4** | **-2,8** | NM_017636 | ILMN_1679401 |
| 110 | **TIMP2** | **-2,8** | NM_003255 | ILMN_1670054 |
| 111 | **TOP2B** | **-2,8** | NM_001068 | ILMN_1777663 |
| 112 | **CRIM1** | **-2,8** | NM_016441 | ILMN_1809793 |
| 113 | **GYG2** | **-2,8** | NM_003918 | ILMN_1815225 |
| 114 | **LPP** | **-2,8** | NM_005578 | ILMN_1651254 |
| 115 | **UNC93B1** | **-2,8** | NM_030930 | ILMN_1654812 |
| 116 | **RALB** | **-2,8** | NM_002881 | ILMN_1676358 |
| 117 | **TACC2** | **-2,8** | NM_006997 | ILMN_1754407 |
| 118 | **ATP2A2** | **-2,8** | NM_001681 | ILMN_1655884 |
| 119 | **C5** | **-2,8** | NM_001735 | ILMN_1746819 |
| 120 | **RNASE4** | **-2,8** | NM_194430 | ILMN_1696974 |
| 121 | **GALM** | **-2,8** | NM_138801 | ILMN_1671482 |
| 122 | **SCAMP1** | **-2,8** | NM_052822 | ILMN_1728907 |
| 123 | **ZC3HAV1** | **-2,8** | NM_020119 | ILMN_1667068 |
| 124 | **CD2AP** | **-2,8** | NM_012120 | ILMN_1730433 |
| 125 | **GATS** | **-2,8** | NM_178831 | ILMN_1699631 |
| 126 | **FLOT2** | **-2,8** | NM_004475 | ILMN_1726222 |
| 127 | **ANG** | **-2,7** | NM_001145 | ILMN_1760727 |
| 128 | **MYO1A** | **-2,7** | NM_005379 | ILMN_1684031 |
| 129 | **SSH3** | **-2,7** | NM_017857 | ILMN_1755234 |
| 130 | **MAP3K8** | **-2,7** | NM_005204 | ILMN_1741159 |
| 131 | **RECQL** | **-2,7** | NM_002907 | ILMN_1680850 |
| 132 | **NDFIP2** | **-2,7** | NM_019080 | ILMN_1677396 |
| 133 | **GCH1** | **-2,7** | NM_001024024 | ILMN_1812759 |
| 134 | **GOLT1B** | **-2,7** | NM_016072 | ILMN_1767837 |
| 135 | **GAL3ST1** | **-2,7** | NM_004861 | ILMN_1729905 |
| 136 | **PCMTD1** | **-2,7** | NM_052937 | ILMN_1737426 |
| 137 | **CAPN1** | **-2,7** | NM_005186 | ILMN_1705261 |
| 138 | **CD38** | **-2,7** | NM_001775 | ILMN_1754039 |
| 139 | **FAM13A1** | **-2,7** | NM_014883 | ILMN_1752510 |
| 140 | **FBLN1** | **-2,7** | NM_006486 | ILMN_1672536 |
| 141 | **INPPL1** | **-2,7** | NM_001567 | ILMN_1728426 |
| 142 | **TRIM31** | **-2,7** | NM_007028 | ILMN_1748685 |
| 143 | **TMPO** | **-2,7** | NM_003276 | ILMN_1768816 |
| 144 | **GABRB3** | **-2,7** | NM_000814 | ILMN_1709681 |
| 145 | **MYO10** | **-2,6** | NM_012334 | ILMN_1703576 |
| 146 | **PAPSS2** | **-2,6** | NM_001015880 | ILMN_1668675 |
| 147 | **PANX2** | **-2,6** | NM_052839 | ILMN_1694810 |
| 148 | **BASP1** | **-2,6** | NM_006317 | ILMN_1651826 |
| 149 | **BCL6** | **-2,6** | NM_001706 | ILMN_1737314 |
| 150 | **NCOA7** | **-2,6** | NM_181782 | ILMN_1687768 |
| 151 | **KIAA0528** | **-2,6** | NM_014802 | ILMN_1682572 |
| 152 | **CENTD3** | **-2,6** | NM_022481 | ILMN_1812618 |
| 153 | **CORO2A** | **-2,6** | NM_052820 | ILMN_1813746 |
| 154 | **CA12** | **-2,6** | NM_001218 | ILMN_1720998 |
| 155 | **RAI17** | **-2,6** | NM_020338 | ILMN_1771627 |
| 156 | **FOXA1** | **-2,6** | NM_004496 | ILMN_1766650 |
| 157 | **SDS** | **-2,6** | NM_006843 | ILMN_1811114 |
| 158 | **IGFBP1** | **-2,6** | NM_000596 | ILMN_1728445 |
| 159 | **DARS2** | **-2,6** | NM_018122 | ILMN_1676191 |
| 160 | **C5orf15** | **-2,6** | NM_020199 | ILMN_1695917 |
| 161 | **ITM2B** | **-2,6** | NM_021999 | ILMN_1713733 |
| 162 | **TOB1** | **-2,6** | NM_005749 | ILMN_1672004 |
| 163 | **LAMA5** | **-2,6** | NM_005560 | ILMN_1773567 |
| 164 | **STK38** | **-2,6** | NM_007271 | ILMN_1799153 |
| 165 | **CYP2S1** | **-2,6** | NM_030622 | ILMN_1705403 |
| 166 | **ACVR1** | **-2,6** | NM_001105 | ILMN_1760490 |
| 167 | **BCHE** | **-2,6** | NM_000055 | ILMN_1685641 |
| 168 | **PGD** | **-2,6** | NM_002631 | ILMN_1794165 |
| 169 | **FAM65A** | **-2,5** | NM_024519 | ILMN_1680037 |
| 170 | **WDR51B** | **-2,5** | NM_172240 | ILMN_1667201 |
| 171 | **PTBP1** | **-2,5** | NM_175847 | ILMN_1655154 |
| 172 | **ALDH2** | **-2,5** | NM_000690 | ILMN_1793859 |
| 173 | **SLC25A24** | **-2,5** | NM_013386 | ILMN_1752639 |
| 174 | **VDP** | **-2,5** | NM_003715 | ILMN_1692121 |
| 175 | **ZA20D2** | **-2,5** | NM_006007 | ILMN_1795228 |
| 176 | **COPB** | **-2,5** | NM_016451 | ILMN_1699112 |
| 177 | **TXNRD1** | **-2,5** | NM_003330 | ILMN_1717056 |
| 178 | **SNX4** | **-2,5** | NM_003794 | ILMN_1738736 |
| 179 | **RAD21** | **-2,5** | NM_006265 | ILMN_1748578 |
| 180 | **RDX** | **-2,5** | NM_002906 | ILMN_1708611 |
| 181 | **FLRT3** | **-2,5** | NM_013281 | ILMN_1805665 |
| 182 | **EPB41L1** | **-2,5** | NM_012156 | ILMN_1719475 |
| 183 | **NPY5R** | **-2,5** | NM_006174 | ILMN_1718198 |
| 184 | **SUV420H2** | **-2,5** | NM_032701 | ILMN_1812208 |
| 185 | **ABCC3** | **-2,5** | NM_020037 | ILMN_1677814 |
| 186 | **CNAP1** | **-2,5** | NM_014865 | ILMN_1775008 |
| 187 | **SLC36A4** | **-2,5** | NM_152313 | ILMN_1802348 |
| 188 | **AXL** | **-2,5** | NM_001699 | ILMN_1701877 |
| 189 | **C1orf115** | **-2,5** | NM_024709 | ILMN_1674817 |
| 190 | **CAV1** | **-2,5** | NM_001753 | ILMN_1687583 |
| 191 | **USP9X** | **-2,5** | NM_004652 | ILMN_1656165 |
| 192 | **CTDSP2** | **-2,5** | NM_005730 | ILMN_1692962 |
| 193 | **VGLL4** | **-2,5** | NM_014667 | ILMN_1768480 |
| 194 | **C19orf21** | **-2,5** | NM_173481 | ILMN_1683905 |
| 195 | **FLJ40629** | **-2,5** | NM_152515 | ILMN_1751776 |
| 196 | **PROS1** | **-2,5** | NM_000313 | ILMN_1671928 |
| 197 | **RTKN** | **-2,5** | NM_001015055 | ILMN_1680591 |
| 198 | **STAT4** | **-2,5** | NM_003151 | ILMN_1785202 |
| 199 | **ACF** | **-2,5** | NM_138933 | ILMN_1806310 |
| 200 | **LEPREL1** | **-2,5** | NM_018192 | ILMN_1657373 |
| 201 | **PTP4A1** | **-2,5** | NM_003463 | ILMN_1760575 |
| 202 | **BUB1** | **-2,5** | NM_004336 | ILMN_1736090 |
| 203 | **CDC2** | **-2,5** | NM_033379 | ILMN_1747911 |
| 204 | **YPEL5** | **-2,5** | NM_016061 | ILMN_1711069 |
| 205 | **MYADM** | **-2,5** | NM_001020820 | ILMN_1658528 |
| 206 | **RCC2** | **-2,5** | NM_018715 | ILMN_1720124 |
| 207 | **PJA2** | **-2,5** | NM_014819 | ILMN_1688702 |
| 208 | **FOS** | **-2,4** | NM_005252 | ILMN_1669523 |
| 209 | **ASPM** | **-2,4** | NM_018136 | ILMN_1815184 |
| 210 | **PDZK1** | **-2,4** | NM_002614 | ILMN_1694535 |
| 211 | **FER1L3** | **-2,4** | NM_013451 | ILMN_1810289 |
| 212 | **BTBD11** | **-2,4** | NM_152322 | ILMN_1705066 |
| 213 | **SPAG5** | **-2,4** | NM_006461 | ILMN_1768291 |
| 214 | **ZNF395** | **-2,4** | NM_018660 | ILMN_1772876 |
| 215 | **SMC4L1** | **-2,4** | NM_001002799 | ILMN_1728556 |
| 216 | **TEGT** | **-2,4** | NM_003217 | ILMN_1693311 |
| 217 | **KCTD3** | **-2,4** | NM_016121 | ILMN_1800220 |
| 218 | **BIN1** | **-2,4** | NM_139348 | ILMN_1674160 |
| 219 | **RHOU** | **-2,4** | NM_021205 | ILMN_1748992 |
| 220 | **TMOD3** | **-2,4** | NM_014547 | ILMN_1809484 |
| 221 | **PAPOLA** | **-2,4** | NM_032632 | ILMN_1798354 |
| 222 | **CRAT** | **-2,4** | NM_000755 | ILMN_1728671 |
| 223 | **TMCO3** | **-2,4** | NM_017905 | ILMN_1654748 |
| 224 | **DUSP5** | **-2,4** | NM_004419 | ILMN_1656501 |
| 225 | **ZBED1** | **-2,4** | NM_004729 | ILMN_1694466 |
| 226 | **DFNA5** | **-2,4** | NM_004403 | ILMN_1670145 |
| 227 | **CFH** | **-2,4** | NM_001014975 | ILMN_1810910 |
| 228 | **CYP24A1** | **-2,4** | NM_000782 | ILMN_1685663 |
| 229 | **CGN** | **-2,4** | NM_020770 | ILMN_1746801 |
| 230 | **DCP2** | **-2,4** | NM_152624 | ILMN_1669905 |
| 231 | **RPS6KA3** | **-2,4** | NM_004586 | ILMN_1806294 |
| 232 | **PPP4R1** | **-2,4** | NM_005134 | ILMN_1724544 |
| 233 | **CHMP2B** | **-2,4** | NM_014043 | ILMN_1683698 |
| 234 | **MAPK3** | **-2,4** | NM_002746 | ILMN_1812747 |
| 235 | **C14orf106** | **-2,4** | NM_018353 | ILMN_1666208 |
| 236 | **AHCYL1** | **-2,4** | NM_006621 | ILMN_1770412 |
| 237 | **SCAP2** | **-2,4** | NM_003930 | ILMN_1657129 |
| 238 | **DHRS8** | **-2,4** | NM_016245 | ILMN_1722076 |
| 239 | **MAP4K2** | **-2,4** | NM_004579 | ILMN_1723625 |
| 240 | **PDXK** | **-2,4** | NM_003681 | ILMN_1672504 |
| 241 | **KLHL5** | **-2,4** | NM_001007075 | ILMN_1706687 |
| 242 | **SHMT1** | **-2,4** | NM_148918 | ILMN_1811933 |
| 243 | **STIL** | **-2,4** | NM_003035 | ILMN_1807232 |
| 244 | **LYPLA1** | **-2,4** | NM_006330 | ILMN_1666713 |
| 245 | **PREI3** | **-2,4** | NM_199482 | ILMN_1733356 |
| 246 | **HIF1A** | **-2,4** | NM_001530 | ILMN_1763260 |
| 247 | **UGP2** | **-2,3** | NM_006759 | ILMN_1671969 |
| 248 | **ARHGAP27** | **-2,3** | NM_199282 | ILMN_1734652 |
| 249 | **CTNNB1** | **-2,3** | NM_001904 | ILMN_1757350 |
| 250 | **CD46** | **-2,3** | NM_153826 | ILMN_1815689 |
| 251 | **SIDT2** | **-2,3** | NM_015996 | ILMN_1791912 |
| 252 | **NUCB1** | **-2,3** | NM_006184 | ILMN_1722634 |
| 253 | **TNS3** | **-2,3** | NM_022748 | ILMN_1667893 |
| 254 | **CYP26B1** | **-2,3** | NM_019885 | ILMN_1812297 |
| 255 | **HERC5** | **-2,3** | NM_016323 | ILMN_1729749 |
| 256 | **PLOD2** | **-2,3** | NM_182943 | ILMN_1799139 |
| 257 | **TMEM123** | **-2,3** | NM_052932 | ILMN_1724139 |
| 258 | **KLHL23** | **-2,3** | NM_144711 | ILMN_1763379 |
| 259 | **SH3KBP1** | **-2,3** | NM_001024666 | ILMN_1808501 |
| 260 | **SEPX1** | **-2,3** | NM_016332 | ILMN_1719661 |
| 261 | **CENPF** | **-2,3** | NM_016343 | ILMN_1664516 |
| 262 | **CLDN1** | **-2,3** | NM_021101 | ILMN_1724686 |
| 263 | **GPRC5A** | **-2,3** | NM_003979 | ILMN_1682599 |
| 264 | **ZNF281** | **-2,3** | NM_012482 | ILMN_1802758 |
| 265 | **SLC7A5** | **-2,3** | NM_003486 | ILMN_1720373 |
| 266 | **ST6GAL1** | **-2,3** | NM_173217 | ILMN_1756501 |
| 267 | **SEC23A** | **-2,3** | NM_006364 | ILMN_1790311 |
| 268 | **FLJ40432** | **-2,3** | NM_152523 | ILMN_1810069 |
| 269 | **CDKN1B** | **-2,3** | NM_004064 | ILMN_1722811 |
| 270 | **PHIP** | **-2,3** | NM_017934 | ILMN_1788689 |
| 271 | **PRKAR1A** | **-2,3** | NM_002734 | ILMN_1738632 |
| 272 | **PDIA4** | **-2,3** | NM_004911 | ILMN_1772118 |
| 273 | **EPHA3** | **-2,3** | NM_005233 | ILMN_1775931 |
| 274 | **MCM5** | **-2,3** | NM_006739 | ILMN_1815169 |
| 275 | **SCP2** | **-2,3** | NM_002979 | ILMN_1694776 |
| 276 | **TTK** | **-2,3** | NM_003318 | ILMN_1788166 |
| 277 | **RIC8B** | **-2,3** | NM_018157 | ILMN_1663532 |
| 278 | **SOX9** | **-2,3** | NM_000346 | ILMN_1705803 |
| 279 | **GOLPH3L** | **-2,3** | NM_018178 | ILMN_1655570 |
| 280 | **DHX9** | **-2,3** | NM_001357 | ILMN_1656016 |
| 281 | **PQLC3** | **-2,3** | NM_152391 | ILMN_1814213 |
| 282 | **FAM107B** | **-2,3** | NM_031453 | ILMN_1758672 |
| 283 | **ROCK2** | **-2,3** | NM_004850 | ILMN_1659099 |
| 284 | **ALDH4A1** | **-2,3** | NM_170726 | ILMN_1656368 |
| 285 | **STT3B** | **-2,3** | NM_178862 | ILMN_1716372 |
| 286 | **DTL** | **-2,2** | NM_016448 | ILMN_1745251 |
| 287 | **GLUD1** | **-2,2** | NM_005271 | ILMN_1713756 |
| 288 | **KIFAP3** | **-2,2** | NM_014970 | ILMN_1697884 |
| 289 | **RBM23** | **-2,2** | NM_018107 | ILMN_1780756 |
| 290 | **C6orf55** | **-2,2** | NM_016485 | ILMN_1690233 |
| 291 | **TSPAN8** | **-2,2** | NM_004616 | ILMN_1683263 |
| 292 | **SDPR** | **-2,2** | NM_004657 | ILMN_1715991 |
| 293 | **CAT** | **-2,2** | NM_001752 | ILMN_1805905 |
| 294 | **PCDH9** | **-2,2** | NM_020403 | ILMN_1752294 |
| 295 | **TSPAN17** | **-2,2** | NM_012171 | ILMN_1777881 |
| 296 | **RCN2** | **-2,2** | NM_002902 | ILMN_1662129 |
| 297 | **HMGB3** | **-2,2** | NM_005342 | ILMN_1733519 |
| 298 | **EXO1** | **-2,2** | NM_003686 | ILMN_1673721 |
| 299 | **ITGB5** | **-2,2** | NM_002213 | ILMN_1796755 |
| 300 | **MGC24665** | **-2,2** | NM_152308 | ILMN_1790537 |
| 301 | **SUOX** | **-2,2** | NM_001032386 | ILMN_1803745 |
| 302 | **DHX15** | **-2,2** | NM_001358 | ILMN_1754839 |
| 303 | **LASP1** | **-2,2** | NM_006148 | ILMN_1665909 |
| 304 | **MUC1** | **-2,2** | NM_001018021 | ILMN_1756992 |
| 305 | **IGSF11** | **-2,2** | NM_001015887 | ILMN_1753502 |
| 306 | **C8orf1** | **-2,2** | NM_004337 | ILMN_1735472 |
| 307 | **MAGED1** | **-2,2** | NM_001005333 | ILMN_1775522 |
| 308 | **SMAD4** | **-2,2** | NM_005359 | ILMN_1741477 |
| 309 | **FRAT2** | **-2,2** | NM_012083 | ILMN_1788213 |
| 310 | **FLJ13236** | **-2,2** | NM_024902 | ILMN_1749636 |
| 311 | **LBR** | **-2,2** | NM_002296 | ILMN_1724240 |
| 312 | **37500** | **-2,2** | NM_001008491 | ILMN_1740252 |
| 313 | **ATXN2** | **-2,2** | NM_002973 | ILMN_1743829 |
| 314 | **NTS** | **-2,2** | NM_006183 | ILMN_1764690 |
| 315 | **KIF23** | **-2,2** | NM_004856 | ILMN_1811472 |
| 316 | **ACTN4** | **-2,2** | NM_004924 | ILMN_1725534 |
| 317 | **PLOD1** | **-2,2** | NM_000302 | ILMN_1684391 |
| 318 | **PLEKHC1** | **-2,2** | NM_006832 | ILMN_1695290 |
| 319 | **PRKCD** | **-2,2** | NM_212539 | ILMN_1801105 |
| 320 | **ZA20D3** | **-2,2** | NM_019006 | ILMN_1778803 |
| 321 | **KYNU** | **-2,2** | NM_003937 | ILMN_1746517 |
| 322 | **GBE1** | **-2,2** | NM_000158 | ILMN_1789702 |
| 323 | **ADAM9** | **-2,2** | NM_003816 | ILMN_1727524 |
| 324 | **COL5A2** | **-2,2** | NM_000393 | ILMN_1729117 |
| 325 | **PCYOX1** | **-2,2** | NM_016297 | ILMN_1679725 |
| 326 | **GPX2** | **-2,2** | NM_002083 | ILMN_1662776 |
| 327 | **G3BP2** | **-2,2** | NM_012297 | ILMN_1720422 |
| 328 | **MYH10** | **-2,2** | NM_005964 | ILMN_1815154 |
| 329 | **KRT8** | **-2,2** | NM_002273 | ILMN_1753584 |
| 330 | **IARS2** | **-2,2** | NM_018060 | ILMN_1671207 |
| 331 | **ATP1B1** | **-2,2** | NM_001677 | ILMN_1736862 |
| 332 | **PLAU** | **-2,2** | NM_002658 | ILMN_1656057 |
| 333 | **EFNA1** | **-2,2** | NM_004428 | ILMN_1775903 |
| 334 | **RASA1** | **-2,1** | NM_002890 | ILMN_1725312 |
| 335 | **YY1** | **-2,1** | NM_003403 | ILMN_1770892 |
| 336 | **PDE4D** | **-2,1** | NM_006203 | ILMN_1791483 |
| 337 | **SCAP** | **-2,1** | NM_012235 | ILMN_1677534 |
| 338 | **PSAP** | **-2,1** | NM_002778 | ILMN_1749109 |
| 339 | **KIAA1370** | **-2,1** | NM_019600 | ILMN_1700733 |
| 340 | **NET1** | **-2,1** | NM_005863 | ILMN_1758311 |
| 341 | **MMD** | **-2,1** | NM_012329 | ILMN_1733937 |
| 342 | **CALD1** | **-2,1** | NM_004342 | ILMN_1730487 |
| 343 | **GLG1** | **-2,1** | NM_012201 | ILMN_1772261 |
| 344 | **GPIAP1** | **-2,1** | NM_203364 | ILMN_1754145 |
| 345 | **CRIPT** | **-2,1** | NM_014171 | ILMN_1813256 |
| 346 | **ABCD3** | **-2,1** | NM_002858 | ILMN_1674032 |
| 347 | **PTPLAD1** | **-2,1** | NM_016395 | ILMN_1658746 |
| 348 | **DSG2** | **-2,1** | NM_001943 | ILMN_1704196 |
| 349 | **ITSN1** | **-2,1** | NM_001001132 | ILMN_1718769 |
| 350 | **DYNC1I2** | **-2,1** | NM_001378 | ILMN_1773847 |
| 351 | **CEBPZ** | **-2,1** | NM_005760 | ILMN_1744147 |
| 352 | **IDH1** | **-2,1** | NM_005896 | ILMN_1696432 |
| 353 | **SLC23A1** | **-2,1** | NM_152685 | ILMN_1760950 |
| 354 | **PAPSS1** | **-2,1** | NM_005443 | ILMN_1781819 |
| 355 | **ALDH5A1** | **-2,1** | NM_001080 | ILMN_1715859 |
| 356 | **WDR1** | **-2,1** | NM_017491 | ILMN_1780036 |
| 357 | **F2** | **-2,1** | NM_000506 | ILMN_1671753 |
| 358 | **LAD1** | **-2,1** | NM_005558 | ILMN_1782389 |
| 359 | **CAV2** | **-2,1** | NM_001233 | ILMN_1658835 |
| 360 | **WDR48** | **-2,1** | NM_020839 | ILMN_1762103 |
| 361 | **C20orf46** | **-2,1** | NM_018354 | ILMN_1751228 |
| 362 | **XRCC5** | **-2,1** | NM_021141 | ILMN_1754919 |
| 363 | **SCARA3** | **-2,1** | NM_182826 | ILMN_1746376 |
| 364 | **SFRS6** | **-2,1** | NM_006275 | ILMN_1697469 |
| 365 | **MCM2** | **-2,1** | NM_004526 | ILMN_1681503 |
| 366 | **STK6** | **-2,1** | NM_198434 | ILMN_1680955 |
| 367 | **NFIB** | **-2,1** | NM_005596 | ILMN_1778991 |
| 368 | **WFS1** | **-2,1** | NM_006005 | ILMN_1759023 |
| 369 | **SMAD3** | **-2,1** | NM_005902 | ILMN_1682738 |
| 370 | **CPOX** | **-2,1** | NM_000097 | ILMN_1726852 |
| 371 | **C5orf21** | **-2,1** | NM_032042 | ILMN_1654542 |
| 372 | **ACTR2** | **-2,1** | NM_001005386 | ILMN_1697774 |
| 373 | **SC4MOL** | **-2,1** | NM_006745 | ILMN_1689842 |
| 374 | **NT5C2** | **-2,1** | NM_012229 | ILMN_1682165 |
| 375 | **FGG** | **-2,1** | NM_000509 | ILMN_1804335 |
| 376 | **PACSIN2** | **-2,1** | NM_007229 | ILMN_1702396 |
| 377 | **TPCN2** | **-2,1** | NM_139075 | ILMN_1726873 |
| 378 | **WDR40A** | **-2,1** | NM_015397 | ILMN_1786328 |
| 379 | **MAP7** | **-2,1** | NM_003980 | ILMN_1712719 |
| 380 | **TAX1BP1** | **-2,1** | NM_006024 | ILMN_1793118 |
| 381 | **VCL** | **-2,1** | NM_003373 | ILMN_1795429 |
| 382 | **GSDMDC1** | **-2,1** | NM_024736 | ILMN_1665428 |
| 383 | **CPD** | **-2,1** | NM_001304 | ILMN_1703074 |
| 384 | **IL13RA1** | **-2,1** | NM_001560 | ILMN_1768505 |
| 385 | **FLJ21908** | **-2,1** | NM_024604 | ILMN_1766916 |
| 386 | **IGSF3** | **-2,1** | NM_001542 | ILMN_1713014 |
| 387 | **HMMR** | **-2,1** | NM_012485 | ILMN_1781942 |
| 388 | **ISG20** | **-2,1** | NM_002201 | ILMN_1659913 |
| 389 | **DNM1L** | **-2,1** | NM_012062 | ILMN_1778690 |
| 390 | **EPDR1** | **-2,1** | NM_017549 | ILMN_1675797 |
| 391 | **FKSG30** | **-2,1** | NM_001017421 | ILMN_1814998 |
| 392 | **RHPN2** | **-2,1** | NM_033103 | ILMN_1753143 |
| 393 | **HSP90B1** | **-2,1** | NM_003299 | ILMN_1686328 |
| 394 | **FLJ10081** | **-2,1** | NM_017991 | ILMN_1663571 |
| 395 | **CAP2** | **-2,1** | NM_006366 | ILMN_1691237 |
| 396 | **MBD4** | **-2,1** | NM_003925 | ILMN_1708764 |
| 397 | **COCH** | **-2,1** | NM_004086 | ILMN_1711514 |
| 398 | **PARP4** | **-2,1** | NM_006437 | ILMN_1776464 |
| 399 | **STX5A** | **-2,1** | NM_003164 | ILMN_1749345 |
| 400 | **SMARCA3** | **-2,1** | NM_003071 | ILMN_1673820 |
| 401 | **KIF20A** | **-2,1** | NM_005733 | ILMN_1695658 |
| 402 | **HRB** | **-2,1** | NM_004504 | ILMN_1792497 |
| 403 | **VIL2** | **-2,1** | NM_003379 | ILMN_1795937 |
| 404 | **SLC2A3** | **-2,1** | NM_006931 | ILMN_1775708 |
| 405 | **LTB4R** | **-2,1** | NM_181657 | ILMN_1723079 |
| 406 | **KIF11** | **-2,1** | NM_004523 | ILMN_1794539 |
| 407 | **ATP9B** | **-2,0** | NM_198531 | ILMN_1658684 |
| 408 | **ADD1** | **-2,0** | NM_001119 | ILMN_1759252 |
| 409 | **EHBP1** | **-2,0** | NM_015252 | ILMN_1803348 |
| 410 | **KIAA1600** | **-2,0** | NM_020940 | ILMN_1752927 |
| 411 | **MID1** | **-2,0** | NM_000381 | ILMN_1761858 |
| 412 | **RAB5A** | **-2,0** | NM_004162 | ILMN_1808875 |
| 413 | **TRAM1** | **-2,0** | NM_014294 | ILMN_1737146 |
| 414 | **ALDH9A1** | **-2,0** | NM_000696 | ILMN_1761804 |
| 415 | **KIF5B** | **-2,0** | NM_004521 | ILMN_1788160 |
| 416 | **40057** | **-2,0** | NM_006640 | ILMN_1769118 |
| 417 | **PRKD2** | **-2,0** | NM_016457 | ILMN_1656953 |
| 418 | **C7orf27** | **-2,0** | NM_152743 | ILMN_1804498 |
| 419 | **ELF1** | **-2,0** | NM_172373 | ILMN_1664010 |
| 420 | **SDSL** | **-2,0** | NM_138432 | ILMN_1750674 |
| 421 | **FOXO3A** | **-2,0** | NM_001455 | ILMN_1681703 |
| 422 | **BTBD2** | **-2,0** | NM_017797 | ILMN_1701660 |
| 423 | **M-RIP** | **-2,0** | NM_201274 | ILMN_1774547 |
| 424 | **QARS** | **-2,0** | NM_005051 | ILMN_1763080 |
| 425 | **REEP5** | **-2,0** | NM_005669 | ILMN_1758941 |
| 426 | **KLHDC3** | **-2,0** | NM_057161 | ILMN_1730940 |
| 427 | **IREB2** | **-2,0** | NM_004136 | ILMN_1726554 |
| 428 | **CALM1** | **-2,0** | NM_006888 | ILMN_1778242 |
| 429 | **NCKAP1** | **-2,0** | NM_013436 | ILMN_1747392 |
| 430 | **SLITL2** | **-2,0** | NM_138440 | ILMN_1667295 |
| 431 | **SNX27** | **-2,0** | NM_030918 | ILMN_1714401 |
| 432 | **CTNNA1** | **-2,0** | NM_001903 | ILMN_1804854 |
| 433 | **EIF4G2** | **-2,0** | NM_001418 | ILMN_1761519 |
| 434 | **MCM7** | **-2,0** | NM_005916 | ILMN_1704702 |
| 435 | **KRT18** | **-2,0** | NM_000224 | ILMN_1803408 |
| 436 | **PECR** | **-2,0** | NM_018441 | ILMN_1814120 |
| 437 | **ADM** | **-2,0** | NM_001124 | ILMN_1708934 |
| 438 | **FLJ23191** | **-2,0** | NM_024574 | ILMN_1689176 |
| 439 | **PIAS3** | **-2,0** | NM_006099 | ILMN_1675497 |
| 440 | **LKAP** | **-2,0** | NM_014647 | ILMN_1793371 |
| 441 | **POLA2** | **-2,0** | NM_002689 | ILMN_1696713 |
| 442 | **CDH17** | **-2,0** | NM_004063 | ILMN_1814015 |
| 443 | **YWHAZ** | **-2,0** | NM_003406 | ILMN_1801928 |
| 444 | **CDCA2** | **-2,0** | NM_152562 | ILMN_1660654 |
| 445 | **GCNT2** | **-2,0** | NM_001491 | ILMN_1680390 |
| 446 | **RPA1** | **-2,0** | NM_002945 | ILMN_1795719 |
| 447 | **HPS6** | **-2,0** | NM_024747 | ILMN_1718537 |
| 448 | **ACO1** | **-2,0** | NM_002197 | ILMN_1750800 |
| 449 | **SLC9A3R1** | **-2,0** | NM_004252 | ILMN_1680925 |
| 450 | **P4HA1** | **-2,0** | NM_000917 | ILMN_1693334 |
| 451 | **KIAA0174** | **-2,0** | NM_014761 | ILMN_1740351 |
| 452 | **E2F2** | **-2,0** | NM_004091 | ILMN_1777233 |
| 453 | **GABRE** | **-2,0** | NM_021990 | ILMN_1784706 |
| 454 | **C1orf112** | **-2,0** | NM_018186 | ILMN_1727540 |
| 455 | **DIP13B** | **-2,0** | NM_018171 | ILMN_1765076 |
| 456 | **RNPEP** | **-2,0** | NM_020216 | ILMN_1738347 |
| 457 | **PBK** | **-2,0** | NM_018492 | ILMN_1673673 |
| 458 | **DDB1** | **-2,0** | NM_001923 | ILMN_1775937 |
| 459 | **ATAD2** | **-2,0** | NM_014109 | ILMN_1763064 |
| 460 | **SNX10** | **-2,0** | NM_013322 | ILMN_1786257 |
| 461 | **TMED7** | **-2,0** | NM_181836 | ILMN_1672405 |
| 462 | **C1orf106** | **-2,0** | NM_018265 | ILMN_1713952 |
| 463 | **PAFAH1B1** | **-2,0** | NM_000430 | ILMN_1722276 |
| 464 | **MVP** | **-2,0** | NM_005115 | ILMN_1803277 |
| 465 | **SFXN1** | **-2,0** | NM_022754 | ILMN_1713680 |
| 466 | **ZNF217** | **-2,0** | NM_006526 | ILMN_1789841 |
| 467 | **SAFB** | **-2,0** | NM_002967 | ILMN_1722059 |
| 468 | **FBXO8** | **-2,0** | NM_012180 | ILMN_1672843 |
| 469 | **SNAP23** | **-2,0** | NM_003825 | ILMN_1687519 |
| 470 | **MKRN1** | **-2,0** | NM_013446 | ILMN_1671583 |
| 471 | **RAB1A** | **-2,0** | NM_004161 | ILMN_1663843 |
| 472 | **CHD9** | **-2,0** | NM_025134 | ILMN_1762972 |
| 473 | **STK3** | **-2,0** | NM_006281 | ILMN_1666453 |
| 474 | **P15RS** | **-2,0** | NM_018170 | ILMN_1764207 |
| 475 | **UBXD2** | **-2,0** | NM_014607 | ILMN_1781097 |
| 476 | **SNX5** | **-2,0** | NM_014426 | ILMN_1714554 |
| 477 | **C17orf70** | **-2,0** | NM_025161 | ILMN_1711823 |
| 478 | **COL4A1** | **-2,0** | NM_001845 | ILMN_1653028 |
| 479 | **NUP107** | **-2,0** | NM_020401 | ILMN_1815924 |
| 480 | **SMAD6** | **-1,9** | NM_005585 | ILMN_1767068 |
| 481 | **LRRC45** | **-1,9** | NM_144999 | ILMN_1754325 |
| 482 | **LACTB** | **-1,9** | NM_032857 | ILMN_1703335 |
| 483 | **PHF21A** | **-1,9** | NM_016621 | ILMN_1699496 |
| 484 | **GOLGA5** | **-1,9** | NM_005113 | ILMN_1773741 |
| 485 | **SCARB1** | **-1,9** | NM_005505 | ILMN_1668387 |
| 486 | **DBN1** | **-1,9** | NM_080881 | ILMN_1769926 |
| 487 | **NCSTN** | **-1,9** | NM_015331 | ILMN_1735180 |
| 488 | **GRAMD1A** | **-1,9** | NM_020895 | ILMN_1737157 |
| 489 | **PGM1** | **-1,9** | NM_002633 | ILMN_1800659 |
| 490 | **MXD4** | **-1,9** | NM_006454 | ILMN_1756541 |
| 491 | **CCNA2** | **-1,9** | NM_001237 | ILMN_1786125 |
| 492 | **EML4** | **-1,9** | NM_019063 | ILMN_1718297 |
| 493 | **C22orf13** | **-1,9** | NM_031444 | ILMN_1764410 |
| 494 | **MELK** | **-1,9** | NM_014791 | ILMN_1731184 |
| 495 | **GMCL1** | **-1,9** | NM_178439 | ILMN_1670532 |
| 496 | **AP3B1** | **-1,9** | NM_003664 | ILMN_1768867 |
| 497 | **OTUD5** | **-1,9** | NM_017602 | ILMN_1726470 |
| 498 | **PRMT7** | **-1,9** | NM_019023 | ILMN_1699994 |
| 499 | **C2orf30** | **-1,9** | NM_015701 | ILMN_1724376 |
| 500 | **C21orf55** | **-1,9** | NM_017833 | ILMN_1814204 |
| 501 | **ANXA1** | **-1,9** | NM_000700 | ILMN_1681783 |
| 502 | **NSF** | **-1,9** | NM_006178 | ILMN_1680353 |
| 503 | **ADAM15** | **-1,9** | NM_207195 | ILMN_1756920 |
| 504 | **GLO1** | **-1,9** | NM_006708 | ILMN_1702177 |
| 505 | **CPNE3** | **-1,9** | NM_003909 | ILMN_1773576 |
| 506 | **RCD-8** | **-1,9** | NM_014329 | ILMN_1665212 |
| 507 | **FBXO5** | **-1,9** | NM_012177 | ILMN_1710676 |
| 508 | **SPG7** | **-1,9** | NM_003119 | ILMN_1754529 |
| 509 | **NAP1L4** | **-1,9** | NM_005969 | ILMN_1804327 |
| 510 | **TMED5** | **-1,9** | NM_016040 | ILMN_1803279 |
| 511 | **MARCKS** | **-1,9** | NM_002356 | ILMN_1807042 |
| 512 | **HK1** | **-1,9** | NM_033500 | ILMN_1727672 |
| 513 | **CLCN7** | **-1,9** | NM_001287 | ILMN_1694731 |
| 514 | **BRD2** | **-1,9** | NM_005104 | ILMN_1758918 |
| 515 | **FBXO21** | **-1,9** | NM_015002 | ILMN_1745887 |
| 516 | **TMEM59** | **-1,9** | NM_004872 | ILMN_1792508 |
| 517 | **ARID3A** | **-1,9** | NM_005224 | ILMN_1670130 |
| 518 | **TKT** | **-1,9** | NM_001064 | ILMN_1736597 |
| 519 | **SRRM1** | **-1,9** | NM_005839 | ILMN_1697670 |
| 520 | **PAIP2** | **-1,9** | NM_001033112 | ILMN_1784753 |
| 521 | **RSPRY1** | **-1,9** | NM_133368 | ILMN_1763694 |
| 522 | **CALM3** | **-1,9** | NM_005184 | ILMN_1666385 |
| 523 | **TMEM41B** | **-1,9** | NM_015012 | ILMN_1678004 |
| 524 | **ARHGAP17** | **-1,9** | NM_018054 | ILMN_1718610 |
| 525 | **EFHD2** | **-1,9** | NM_024329 | ILMN_1761463 |
| 526 | **DEFB1** | **-1,9** | NM_005218 | ILMN_1686573 |
| 527 | **SFRS2** | **-1,9** | NM_003016 | ILMN_1696407 |
| 528 | **PHLDB1** | **-1,9** | NM_015157 | ILMN_1666819 |
| 529 | **MATR3** | **-1,9** | NM_018834 | ILMN_1815107 |
| 530 | **CCNI** | **-1,9** | NM_006835 | ILMN_1691942 |
| 531 | **PARP1** | **-1,9** | NM_001618 | ILMN_1686871 |
| 532 | **C9orf88** | **-1,9** | NM_022833 | ILMN_1661755 |
| 533 | **SLC44A1** | **-1,9** | NM_080546 | ILMN_1700695 |
| 534 | **CSE1L** | **-1,9** | NM_001316 | ILMN_1665797 |
| 535 | **ZNF265** | **-1,9** | NM_005455 | ILMN_1703015 |
| 536 | **RXRB** | **-1,9** | NM_021976 | ILMN_1774074 |
| 537 | **GABPA** | **-1,9** | NM_002040 | ILMN_1750029 |
| 538 | **OLFML2A** | **-1,9** | NM_182487 | ILMN_1761425 |
| 539 | **NBN** | **-1,8** | NM_001024688 | ILMN_1734833 |
| 540 | **SDC1** | **-1,8** | NM_002997 | ILMN_1815308 |
| 541 | **GEMIN4** | **-1,8** | NM_015721 | ILMN_1770206 |
| 542 | **SYTL4** | **-1,8** | NM_080737 | ILMN_1719599 |
| 543 | **SLC7A7** | **-1,8** | NM_003982 | ILMN_1810275 |
| 544 | **RAB10** | **-1,8** | NM_016131 | ILMN_1793433 |
| 545 | **CPN1** | **-1,8** | NM_001308 | ILMN_1808674 |
| 546 | **CCT6A** | **-1,8** | NM_001762 | ILMN_1719611 |
| 547 | **MFGE8** | **-1,8** | NM_005928 | ILMN_1756071 |
| 548 | **SPHK2** | **-1,8** | NM_020126 | ILMN_1729281 |
| 549 | **SUZ12** | **-1,8** | NM_015355 | ILMN_1797813 |
| 550 | **STOM** | **-1,8** | NM_004099 | ILMN_1766657 |
| 551 | **FLJ20160** | **-1,8** | NM_017694 | ILMN_1710209 |
| 552 | **SPP1** | **-1,8** | NM_000582 | ILMN_1651354 |
| 553 | **ABL1** | **-1,8** | NM_007313 | ILMN_1713732 |
| 554 | **DDX5** | **-1,8** | NM_004396 | ILMN_1805344 |
| 555 | **PPARG** | **-1,8** | NM_138712 | ILMN_1800225 |
| 556 | **PLAUR** | **-1,8** | NM_1005376 | ILMN_1691508 |
| 557 | **RAD54B** | **-1,8** | NM_012415 | ILMN_1722127 |
| 558 | **SF3B1** | **-1,8** | NM_012433 | ILMN_1813603 |
| 559 | **C2orf29** | **-1,8** | NM_017546 | ILMN_1708906 |
| 560 | **CCM2** | **-1,8** | NM_031443 | ILMN_1784352 |
| 561 | **ROD1** | **-1,8** | NM_005156 | ILMN_1768197 |
| 562 | **MAT2B** | **-1,8** | NM_013283 | ILMN_1688437 |
| 563 | **HSPA1B** | **-1,8** | NM_005346 | ILMN_1660436 |
| 564 | **GPT2** | **-1,8** | NM_133443 | ILMN_1750527 |
| 565 | **KBTBD2** | **-1,8** | NM_015483 | ILMN_1784540 |
| 566 | **CTSD** | **-1,8** | NM_001909 | ILMN_1714546 |
| 567 | **SNX6** | **-1,8** | NM_152233 | ILMN_1807873 |
| 568 | **NCOA3** | **-1,8** | NM_006534 | ILMN_1708805 |
| 569 | **BCLAF1** | **-1,8** | NM_014739 | ILMN_1775192 |
| 570 | **MBTPS1** | **-1,8** | NM_201268 | ILMN_1651719 |
| 571 | **USP3** | **-1,8** | NM_006537 | ILMN_1725862 |
| 572 | **TNFRSF1A** | **-1,8** | NM_001065 | ILMN_1685005 |
| 573 | **RRM1** | **-1,8** | NM_001033 | ILMN_1771593 |
| 574 | **PGRMC2** | **-1,8** | NM_006320 | ILMN_1711682 |
| 575 | **GCLM** | **-1,8** | NM_002061 | ILMN_1788547 |
| 576 | **DEDD2** | **-1,8** | NM_133328 | ILMN_1768031 |
| 577 | **RIPK2** | **-1,8** | NM_003821 | ILMN_1758939 |
| 578 | **ZBTB33** | **-1,8** | NM_006777 | ILMN_1673138 |
| 579 | **GOT2** | **-1,8** | NM_002080 | ILMN_1801348 |
| 580 | **KDELC2** | **-1,8** | NM_153705 | ILMN_1651557 |
| 581 | **MGEA5** | **-1,8** | NM_012215 | ILMN_1686750 |
| 582 | **CPS1** | **-1,8** | NM_001875 | ILMN_1792748 |
| 583 | **BCDIN3** | **-1,8** | NM_019606 | ILMN_1739616 |
| 584 | **DAP** | **-1,8** | NM_004394 | ILMN_1715637 |
| 585 | **TSC22D4** | **-1,8** | NM_030935 | ILMN_1706609 |
| 586 | **CAPZA2** | **-1,8** | NM_006136 | ILMN_1768870 |
| 587 | **PPP2R1A** | **-1,8** | NM_014225 | ILMN_1810467 |
| 588 | **PGK1** | **-1,8** | NM_000291 | ILMN_1755749 |
| 589 | **EIF2AK1** | **-1,8** | NM_014413 | ILMN_1700815 |
| 590 | **C16orf58** | **-1,7** | NM_022744 | ILMN_1685289 |
| 591 | **HNRPM** | **-1,7** | NM_031203 | ILMN_1745385 |
| 592 | **HIBADH** | **-1,7** | NM_152740 | ILMN_1804150 |
| 593 | **OSBP** | **-1,7** | NM_002556 | ILMN_1706376 |
| 594 | **CUL1** | **-1,7** | NM_003592 | ILMN_1749629 |
| 595 | **NRD1** | **-1,7** | NM_002525 | ILMN_1800897 |
| 596 | **JOSD1** | **-1,7** | NM_014876 | ILMN_1798536 |
| 597 | **RHOQ** | **-1,7** | NM_012249 | ILMN_1810559 |
| 598 | **KIAA1838** | **-1,7** | NM_032448 | ILMN_1679641 |
| 599 | **ADAR** | **-1,7** | NM_015840 | ILMN_1776777 |
| 600 | **TGM2** | **-1,7** | NM_004613 | ILMN_1705750 |
| 601 | **ABHD10** | **-1,7** | NM_018394 | ILMN_1770031 |
| 602 | **LMNA** | **-1,7** | NM_005572 | ILMN_1696749 |
| 603 | **ARFGAP3** | **-1,7** | NM_014570 | ILMN_1731287 |
| 604 | **TACC1** | **-1,7** | NM_006283 | ILMN_1770084 |
| 605 | **SLC37A4** | **-1,7** | NM_001467 | ILMN_1678678 |
| 606 | **FAM113B** | **-1,7** | NM_138371 | ILMN_1712431 |
| 607 | **NCOA1** | **-1,7** | NM_147223 | ILMN_1669033 |
| 608 | **KNS2** | **-1,7** | NM_005552 | ILMN_1654653 |
| 609 | **ZNF161** | **-1,7** | NM_007146 | ILMN_1705310 |
| 610 | **MLPH** | **-1,7** | NM_024101 | ILMN_1795342 |
| 611 | **FN3KRP** | **-1,7** | NM_024619 | ILMN_1652333 |
| 612 | **COG2** | **-1,7** | NM_007357 | ILMN_1776993 |
| 613 | **EPRS** | **-1,7** | NM_004446 | ILMN_1783695 |
| 614 | **ASCC1** | **-1,7** | NM_015947 | ILMN_1685097 |
| 615 | **STARD7** | **-1,7** | NM_020151 | ILMN_1774415 |
| 616 | **EPAS1** | **-1,7** | NM_001430 | ILMN_1760034 |
| 617 | **DNMT1** | **-1,7** | NM_001379 | ILMN_1760201 |
| 618 | **C19orf2** | **-1,7** | NM_134447 | ILMN_1798728 |
| 619 | **TRFP** | **-1,7** | NM_004275 | ILMN_1777526 |
| 620 | **NULP1** | **-1,7** | NM_014972 | ILMN_1814971 |
| 621 | **FLJ20105** | **-1,7** | NM_017669 | ILMN_1790992 |
| 622 | **GPI** | **-1,7** | NM_000175 | ILMN_1654465 |
| 623 | **PAICS** | **-1,7** | NM_006452 | ILMN_1773760 |
| 624 | **BUB3** | **-1,7** | NM_004725 | ILMN_1693145 |
| 625 | **PRC1** | **-1,6** | NM_003981 | ILMN_1728934 |
| 626 | **UBQLN4** | **-1,6** | NM_020131 | ILMN_1789349 |
| 627 | **NCOA4** | **-1,6** | NM_005437 | ILMN_1773906 |
| 628 | **FAM20B** | **-1,6** | NM_014864 | ILMN_1811373 |
| 629 | **C9orf10** | **-1,6** | NM_014612 | ILMN_1721089 |
| 630 | **LACTB2** | **-1,6** | NM_016027 | ILMN_1660635 |
| 631 | **SF3B2** | **-1,6** | NM_006842 | ILMN_1737807 |
| 632 | **PGRMC1** | **-1,6** | NM_006667 | ILMN_1684771 |
| 633 | **ACLY** | **-1,6** | NM_198830 | ILMN_1749014 |
| 634 | **MAPRE1** | **-1,6** | NM_012325 | ILMN_1777721 |
| 635 | **TYK2** | **-1,6** | NM_003331 | ILMN_1676955 |
| 636 | **CAP1** | **-1,6** | NM_006367 | ILMN_1797604 |
| 637 | **KIAA1542** | **-1,6** | NM_020901 | ILMN_1715032 |
| 638 | **PARP6** | **-1,6** | NM_020213 | ILMN_1703701 |
| 639 | **GNS** | **-1,5** | NM_002076 | ILMN_1744517 |
| 640 | **LOC93343** | **1,6** | NM_138401 | ILMN_1664587 |
| 641 | **RPS8** | **1,6** | NM_001012 | ILMN_1656203 |
| 642 | **RPS24** | **1,6** | NM_001026 | ILMN_1783489 |
| 643 | **RPS4X** | **1,7** | NM_001007 | ILMN_1810577 |
| 644 | **FDXR** | **1,7** | NM_024417 | ILMN_1799319 |
| 645 | **SLC35B1** | **1,7** | NM_005827 | ILMN_1727840 |
| 646 | **PARK7** | **1,7** | NM_007262 | ILMN_1744713 |
| 647 | **GNPDA1** | **1,7** | NM_005471 | ILMN_1784709 |
| 648 | **ADCK1** | **1,7** | NM_020421 | ILMN_1698777 |
| 649 | **NDUFA1** | **1,7** | NM_004541 | ILMN_1784286 |
| 650 | **CYB5R1** | **1,7** | NM_016243 | ILMN_1729237 |
| 651 | **C17orf45** | **1,8** | NM_152350 | ILMN_1719224 |
| 652 | **RPS4Y1** | **1,8** | NM_001008 | ILMN_1783142 |
| 653 | **C1QBP** | **1,8** | NM_001212 | ILMN_1668996 |
| 654 | **FBL** | **1,8** | NM_001436 | ILMN_1719205 |
| 655 | **RPL36AL** | **1,8** | NM_001001 | ILMN_1691599 |
| 656 | **PIN1** | **1,8** | NM_006221 | ILMN_1776375 |
| 657 | **WBP5** | **1,8** | NM_001006614 | ILMN_1679838 |
| 658 | **XTP3TPA** | **1,8** | NM_024096 | ILMN_1763129 |
| 659 | **ATP5G2** | **1,8** | NM_005176 | ILMN_1703046 |
| 660 | **MRPS22** | **1,8** | NM_020191 | ILMN_1655377 |
| 661 | **NAT5** | **1,8** | NM_181528 | ILMN_1689097 |
| 662 | **C3orf31** | **1,8** | NM_138807 | ILMN_1793724 |
| 663 | **MED28** | **1,8** | NM_025205 | ILMN_1749821 |
| 664 | **LOC374395** | **1,8** | NM_199337 | ILMN_1733757 |
| 665 | **PA2G4** | **1,8** | NM_006191 | ILMN_1728984 |
| 666 | **HSCARG** | **1,8** | NM_020677 | ILMN_1709814 |
| 667 | **TMEM60** | **1,8** | NM_032936 | ILMN_1752213 |
| 668 | **ASNS** | **1,8** | NM_133436 | ILMN_1796417 |
| 669 | **THEM2** | **1,8** | NM_018473 | ILMN_1797336 |
| 670 | **EBP** | **1,8** | NM_006579 | ILMN_1658065 |
| 671 | **RPS14** | **1,8** | NM_001025071 | ILMN_1666635 |
| 672 | **CD2BP2** | **1,8** | NM_006110 | ILMN_1807264 |
| 673 | **RRAS** | **1,9** | NM_006270 | ILMN_1780825 |
| 674 | **NDUFA4** | **1,9** | NM_002489 | ILMN_1751258 |
| 675 | **MRPL43** | **1,9** | NM_032112 | ILMN_1681131 |
| 676 | **POLR2E** | **1,9** | NM_002695 | ILMN_1746679 |
| 677 | **S100A4** | **1,9** | NM_002961 | ILMN_1684306 |
| 678 | **SFXN4** | **1,9** | NM_178867 | ILMN_1658437 |
| 679 | **HIST2H2AC** | **1,9** | NM_003517 | ILMN_1768973 |
| 680 | **FXYD5** | **1,9** | NM_014164 | ILMN_1704286 |
| 681 | **CSNK1E** | **1,9** | NM_152221 | ILMN_1724363 |
| 682 | **COX5A** | **1,9** | NM_004255 | ILMN_1704477 |
| 683 | **C15orf24** | **1,9** | NM_020154 | ILMN_1704633 |
| 684 | **ATP5O** | **1,9** | NM_001697 | ILMN_1734782 |
| 685 | **CGI-69** | **1,9** | NM_016016 | ILMN_1721723 |
| 686 | **TMEM126B** | **1,9** | NM_018480 | ILMN_1766851 |
| 687 | **F12** | **1,9** | NM_000505 | ILMN_1671766 |
| 688 | **DBI** | **1,9** | NM_020548 | ILMN_1715120 |
| 689 | **NUDT3** | **1,9** | NM_006703 | ILMN_1724907 |
| 690 | **DJ122O8.2** | **1,9** | NM_020466 | ILMN_1703132 |
| 691 | **PYCR2** | **1,9** | NM_013328 | ILMN_1808568 |
| 692 | **SELS** | **1,9** | NM_203472 | ILMN_1803744 |
| 693 | **NUDC** | **1,9** | NM_006600 | ILMN_1774079 |
| 694 | **COMMD3** | **1,9** | NM_012071 | ILMN_1690392 |
| 695 | **UPF2** | **1,9** | NM_015542 | ILMN_1739283 |
| 696 | **TUBB2B** | **1,9** | NM_178012 | ILMN_1680874 |
| 697 | **PSMB3** | **1,9** | NM_002795 | ILMN_1748651 |
| 698 | **C12orf10** | **1,9** | NM_021640 | ILMN_1777765 |
| 699 | **COX6B1** | **1,9** | NM_001863 | ILMN_1781094 |
| 700 | **S100A6** | **1,9** | NM_014624 | ILMN_1713636 |
| 701 | **LGALS1** | **1,9** | NM_002305 | ILMN_1723978 |
| 702 | **JTV1** | **1,9** | NM_006303 | ILMN_1673252 |
| 703 | **RPL35A** | **1,9** | NM_000996 | ILMN_1756360 |
| 704 | **EBNA1BP2** | **2,0** | NM_006824 | ILMN_1768127 |
| 705 | **CIB1** | **2,0** | NM_006384 | ILMN_1656899 |
| 706 | **CPLX2** | **2,0** | NM_006650 | ILMN_1669382 |
| 707 | **CCDC72** | **2,0** | NM_015933 | ILMN_1707783 |
| 708 | **YIF1A** | **2,0** | NM_020470 | ILMN_1712975 |
| 709 | **F8A1** | **2,0** | NM_012151 | ILMN_1790249 |
| 710 | **NDUFB2** | **2,0** | NM_004546 | ILMN_1714495 |
| 711 | **STOML2** | **2,0** | NM_013442 | ILMN_1663002 |
| 712 | **AUP1** | **2,0** | NM_012103 | ILMN_1651378 |
| 713 | **MRPS30** | **2,0** | NM_016640 | ILMN_1726743 |
| 714 | **C2orf26** | **2,0** | NM_023016 | ILMN_1724040 |
| 715 | **UQCRB** | **2,0** | NM_006294 | ILMN_1759453 |
| 716 | **COX7A2** | **2,0** | NM_001865 | ILMN_1701293 |
| 717 | **SERF1B** | **2,0** | NM_022978 | ILMN_1655011 |
| 718 | **MGC13170** | **2,0** | NM_199249 | ILMN_1759184 |
| 719 | **NDUFA8** | **2,0** | NM_014222 | ILMN_1759729 |
| 720 | **NME2** | **2,0** | NM_002512 | ILMN_1662905 |
| 721 | **NUDT1** | **2,0** | NM_198954 | ILMN_1735692 |
| 722 | **RHOG** | **2,0** | NM_001665 | ILMN_1739792 |
| 723 | **C9orf74** | **2,0** | NM_030914 | ILMN_1774196 |
| 724 | **LOC283377** | **2,0** | NM_207344 | ILMN_1729868 |
| 725 | **HARS2** | **2,0** | NM_080820 | ILMN_1774432 |
| 726 | **SEC61G** | **2,0** | NM_001012456 | ILMN_1787026 |
| 727 | **EIF2B2** | **2,0** | NM_014239 | ILMN_1713380 |
| 728 | **C20orf52** | **2,0** | NM_080748 | ILMN_1664429 |
| 729 | **ATG3** | **2,0** | NM_022488 | ILMN_1769566 |
| 730 | **MIF** | **2,0** | NM_002415 | ILMN_1716169 |
| 731 | **TBCA** | **2,0** | NM_004607 | ILMN_1726239 |
| 732 | **SHFM1** | **2,0** | NM_006304 | ILMN_1794505 |
| 733 | **MGC4172** | **2,0** | NM_024308 | ILMN_1756701 |
| 734 | **AP1S1** | **2,0** | NM_001283 | ILMN_1662927 |
| 735 | **RPL26** | **2,0** | NM_000987 | ILMN_1731546 |
| 736 | **PRR6** | **2,0** | NM_181716 | ILMN_1729142 |
| 737 | **DAD1** | **2,0** | NM_001344 | ILMN_1731619 |
| 738 | **UBA52** | **2,0** | NM_003333 | ILMN_1782977 |
| 739 | **SIVA** | **2,0** | NM_006427 | ILMN_1679841 |
| 740 | **HSPC148** | **2,1** | NM_016403 | ILMN_1713482 |
| 741 | **ID3** | **2,1** | NM_002167 | ILMN_1732296 |
| 742 | **NDUFB8** | **2,1** | NM_005004 | ILMN_1661170 |
| 743 | **SPAG7** | **2,1** | NM_004890 | ILMN_1684446 |
| 744 | **C6orf79** | **2,1** | NM_022102 | ILMN_1761961 |
| 745 | **PRRT3** | **2,1** | NM_207351 | ILMN_1784985 |
| 746 | **EIF3S4** | **2,1** | NM_003755 | ILMN_1689446 |
| 747 | **NDUFB3** | **2,1** | NM_002491 | ILMN_1718428 |
| 748 | **SF3B14** | **2,1** | NM_016047 | ILMN_1703720 |
| 749 | **NUTF2** | **2,1** | NM_005796 | ILMN_1655046 |
| 750 | **TOMM22** | **2,1** | NM_020243 | ILMN_1714623 |
| 751 | **UBIAD1** | **2,1** | NM_013319 | ILMN_1651872 |
| 752 | **IMP3** | **2,1** | NM_018285 | ILMN_1733696 |
| 753 | **MRPL52** | **2,1** | NM_180981 | ILMN_1758578 |
| 754 | **ATPIF1** | **2,1** | NM_016311 | ILMN_1727332 |
| 755 | **COMMD6** | **2,1** | NM_203495 | ILMN_1777378 |
| 756 | **RPL18A** | **2,1** | NM_000980 | ILMN_1652748 |
| 757 | **TXNL5** | **2,1** | NM_032731 | ILMN_1659437 |
| 758 | **MRPS18A** | **2,1** | NM_018135 | ILMN_1730391 |
| 759 | **AKR1A1** | **2,1** | NM_006066 | ILMN_1728047 |
| 760 | **MRPL24** | **2,1** | NM_024540 | ILMN_1695576 |
| 761 | **MRPL32** | **2,1** | NM_031903 | ILMN_1749432 |
| 762 | **FBXO22** | **2,1** | NM_012170 | ILMN_1786469 |
| 763 | **MRPL18** | **2,1** | NM_014161 | ILMN_1804479 |
| 764 | **C16orf33** | **2,1** | NM_024571 | ILMN_1801118 |
| 765 | **HEBP1** | **2,1** | NM_015987 | ILMN_1802557 |
| 766 | **COX7C** | **2,1** | NM_001867 | ILMN_1798189 |
| 767 | **C14orf156** | **2,1** | NM_031210 | ILMN_1661945 |
| 768 | **VKORC1** | **2,1** | NM_024006 | ILMN_1739946 |
| 769 | **C11orf10** | **2,1** | NM_014206 | ILMN_1786759 |
| 770 | **CCDC23** | **2,1** | NM_199342 | ILMN_1801283 |
| 771 | **TMEM14C** | **2,1** | NM_016462 | ILMN_1657857 |
| 772 | **DYNLRB1** | **2,1** | NM_014183 | ILMN_1658053 |
| 773 | **PDCD2** | **2,1** | NM_002598 | ILMN_1686401 |
| 774 | **ZNF524** | **2,1** | NM_153219 | ILMN_1810147 |
| 775 | **ZMYND19** | **2,1** | NM_138462 | ILMN_1757627 |
| 776 | **EIF4E2** | **2,1** | NM_004846 | ILMN_1738326 |
| 777 | **DYNLT1** | **2,1** | NM_006519 | ILMN_1678766 |
| 778 | **LSM5** | **2,1** | NM_012322 | ILMN_1737947 |
| 779 | **TMEM111** | **2,1** | NM_018447 | ILMN_1812325 |
| 780 | **EXOSC7** | **2,1** | NM_015004 | ILMN_1726354 |
| 781 | **GALR2** | **2,1** | NM_003857 | ILMN_1733847 |
| 782 | **C20orf35** | **2,1** | NM_018478 | ILMN_1730612 |
| 783 | **NFKBIB** | **2,1** | NM_002503 | ILMN_1690473 |
| 784 | **GRHPR** | **2,1** | NM_012203 | ILMN_1664798 |
| 785 | **SNRPD2** | **2,1** | NM_004597 | ILMN_1679088 |
| 786 | **DCI** | **2,1** | NM_001919 | ILMN_1803485 |
| 787 | **MTCP1** | **2,1** | NM_001018025 | ILMN_1814230 |
| 788 | **CES2** | **2,2** | NM_198061 | ILMN_1696675 |
| 789 | **HINT1** | **2,2** | NM_005340 | ILMN_1807710 |
| 790 | **SAC3D1** | **2,2** | NM_013299 | ILMN_1776674 |
| 791 | **PFN1** | **2,2** | NM_005022 | ILMN_1763012 |
| 792 | **ITGB4BP** | **2,2** | NM_181466 | ILMN_1787410 |
| 793 | **STRA13** | **2,2** | NM_144998 | ILMN_1769634 |
| 794 | **FAM58A** | **2,2** | NM_152274 | ILMN_1770623 |
| 795 | **TIMM23** | **2,2** | NM_006327 | ILMN_1773493 |
| 796 | **TMEM42** | **2,2** | NM_144638 | ILMN_1760245 |
| 797 | **SCO2** | **2,2** | NM_005138 | ILMN_1811522 |
| 798 | **C8orf40** | **2,2** | NM_138436 | ILMN_1677385 |
| 799 | **C7orf36** | **2,2** | NM_020192 | ILMN_1793508 |
| 800 | **GNG11** | **2,2** | NM_004126 | ILMN_1782419 |
| 801 | **MRPS23** | **2,2** | NM_016070 | ILMN_1687359 |
| 802 | **HIST1H2BD** | **2,2** | NM_138720 | ILMN_1758623 |
| 803 | **GAMT** | **2,2** | NM_000156 | ILMN_1756469 |
| 804 | **BMP4** | **2,2** | NM_001202 | ILMN_1740900 |
| 805 | **COX6A1** | **2,2** | NM_004373 | ILMN_1783636 |
| 806 | **FAM44B** | **2,2** | NM_138369 | ILMN_1716730 |
| 807 | **ACTA2** | **2,2** | NM_001613 | ILMN_1671703 |
| 808 | **NDUFS3** | **2,2** | NM_004551 | ILMN_1756355 |
| 809 | **MGC61571** | **2,2** | NM_182523 | ILMN_1707634 |
| 810 | **EXOSC3** | **2,2** | NM_016042 | ILMN_1734194 |
| 811 | **RPL24** | **2,2** | NM_000986 | ILMN_1656662 |
| 812 | **MGC3731** | **2,2** | NM_024313 | ILMN_1759991 |
| 813 | **PPIAL4** | **2,2** | NM_178230 | ILMN_1776260 |
| 814 | **STAMBPL1** | **2,2** | NM_020799 | ILMN_1682799 |
| 815 | **PEMT** | **2,2** | NM_148173 | ILMN_1745806 |
| 816 | **NDUFA2** | **2,2** | NM_002488 | ILMN_1767123 |
| 817 | **ATP5J** | **2,2** | NM_001003703 | ILMN_1661574 |
| 818 | **C12orf57** | **2,2** | NM_138425 | ILMN_1812191 |
| 819 | **C18orf56** | **2,2** | NM_001012716 | ILMN_1796074 |
| 820 | **DHX33** | **2,2** | NM_020162 | ILMN_1730059 |
| 821 | **SERTAD1** | **2,2** | NM_013376 | ILMN_1794017 |
| 822 | **SLC25A4** | **2,2** | NM_001151 | ILMN_1681670 |
| 823 | **CCDC59** | **2,2** | NM_014167 | ILMN_1662318 |
| 824 | **LSM2** | **2,2** | NM_021177 | ILMN_1791544 |
| 825 | **ATP5H** | **2,2** | NM_005177 | ILMN_1703047 |
| 826 | **RNF113A** | **2,2** | NM_006978 | ILMN_1786388 |
| 827 | **LOC201164** | **2,2** | NM_178836 | ILMN_1731518 |
| 828 | **NMB** | **2,2** | NM_021077 | ILMN_1683940 |
| 829 | **ENDOG** | **2,2** | NM_004435 | ILMN_1722309 |
| 830 | **RPL6** | **2,2** | NM_001024662 | ILMN_1717490 |
| 831 | **SNRPF** | **2,2** | NM_003095 | ILMN_1678966 |
| 832 | **MTHFS** | **2,2** | NM_006441 | ILMN_1772302 |
| 833 | **FKBP2** | **2,2** | NM_004470 | ILMN_1807563 |
| 834 | **SNFT** | **2,3** | NM_018664 | ILMN_1763207 |
| 835 | **C21orf59** | **2,3** | NM_017835 | ILMN_1776147 |
| 836 | **MRPS26** | **2,3** | NM_030811 | ILMN_1676026 |
| 837 | **MRPS33** | **2,3** | NM_016071 | ILMN_1741264 |
| 838 | **C6orf136** | **2,3** | NM_145029 | ILMN_1813236 |
| 839 | **MRPL33** | **2,3** | NM_004891 | ILMN_1731599 |
| 840 | **ZMAT5** | **2,3** | NM_019103 | ILMN_1745421 |
| 841 | **NXT1** | **2,3** | NM_013248 | ILMN_1760280 |
| 842 | **STC2** | **2,3** | NM_003714 | ILMN_1691884 |
| 843 | **TOMM7** | **2,3** | NM_019059 | ILMN_1674069 |
| 844 | **POLE4** | **2,3** | NM_019896 | ILMN_1660063 |
| 845 | **NEIL2** | **2,3** | NM_145043 | ILMN_1715680 |
| 846 | **ZFPM1** | **2,3** | NM_153813 | ILMN_1651438 |
| 847 | **AP2S1** | **2,3** | NM_004069 | ILMN_1662426 |
| 848 | **PHLDA2** | **2,3** | NM_003311 | ILMN_1671557 |
| 849 | **COX8A** | **2,3** | NM_004074 | ILMN_1809495 |
| 850 | **GUK1** | **2,3** | NM_000858 | ILMN_1758398 |
| 851 | **FLAD1** | **2,3** | NM_201398 | ILMN_1663667 |
| 852 | **MRPL11** | **2,3** | NM_016050 | ILMN_1690371 |
| 853 | **RRAGC** | **2,3** | NM_022157 | ILMN_1760121 |
| 854 | **GTF2H5** | **2,3** | NM_207118 | ILMN_1739497 |
| 855 | **CLPP** | **2,3** | NM_006012 | ILMN_1725705 |
| 856 | **ID2** | **2,3** | NM_002166 | ILMN_1793990 |
| 857 | **IMMP1L** | **2,3** | NM_144981 | ILMN_1749612 |
| 858 | **MID1IP1** | **2,3** | NM_021242 | ILMN_1668960 |
| 859 | **SAMD4A** | **2,3** | NM_015589 | ILMN_1690466 |
| 860 | **RPP25** | **2,3** | NM_017793 | ILMN_1695271 |
| 861 | **C6orf66** | **2,3** | NM_014165 | ILMN_1659524 |
| 862 | **PSMA7** | **2,3** | NM_002792 | ILMN_1754544 |
| 863 | **SDF2L1** | **2,3** | NM_022044 | ILMN_1749213 |
| 864 | **C17orf49** | **2,3** | NM_174893 | ILMN_1763688 |
| 865 | **COL7A1** | **2,3** | NM_000094 | ILMN_1751161 |
| 866 | **CCNB1IP1** | **2,3** | NM_021178 | ILMN_1752394 |
| 867 | **SRP19** | **2,3** | NM_003135 | ILMN_1728385 |
| 868 | **FAM98C** | **2,3** | NM_174905 | ILMN_1760609 |
| 869 | **GRINA** | **2,3** | NM_000837 | ILMN_1796490 |
| 870 | **UAP1L1** | **2,3** | NM_207309 | ILMN_1653712 |
| 871 | **MGC13096** | **2,3** | NM_032346 | ILMN_1706149 |
| 872 | **C18orf21** | **2,3** | NM_031446 | ILMN_1805998 |
| 873 | **FLJ90652** | **2,3** | NM_173618 | ILMN_1724406 |
| 874 | **TRAPPC4** | **2,3** | NM_016146 | ILMN_1814650 |
| 875 | **ATP5L** | **2,3** | NM_006476 | ILMN_1812638 |
| 876 | **LSMD1** | **2,3** | NM_032356 | ILMN_1733960 |
| 877 | **NDUFS5** | **2,3** | NM_004552 | ILMN_1776104 |
| 878 | **PHPT1** | **2,3** | NM_014172 | ILMN_1676611 |
| 879 | **POLR1D** | **2,3** | NM_015972 | ILMN_1767422 |
| 880 | **DYRK4** | **2,3** | NM_003845 | ILMN_1777991 |
| 881 | **C12orf47** | **2,3** | NM_016534 | ILMN_1798957 |
| 882 | **PTD008** | **2,3** | NM_016145 | ILMN_1757914 |
| 883 | **RPL39L** | **2,3** | NM_052969 | ILMN_1712413 |
| 884 | **HSPC196** | **2,4** | NM_016464 | ILMN_1664761 |
| 885 | **CST3** | **2,4** | NM_000099 | ILMN_1800354 |
| 886 | **QP-C** | **2,4** | NM_014402 | ILMN_1666471 |
| 887 | **NDUFB11** | **2,4** | NM_019056 | ILMN_1749709 |
| 888 | **MRPS11** | **2,4** | NM_176805 | ILMN_1722905 |
| 889 | **SLC2A8** | **2,4** | NM_014580 | ILMN_1724609 |
| 890 | **NDUFS6** | **2,4** | NM_004553 | ILMN_1706765 |
| 891 | **RPL10A** | **2,4** | NM_007104 | ILMN_1808041 |
| 892 | **FAM86A** | **2,4** | NM_201400 | ILMN_1780298 |
| 893 | **H2AFJ** | **2,4** | NM_177925 | ILMN_1708728 |
| 894 | **MGC4825** | **2,4** | NM_024122 | ILMN_1721623 |
| 895 | **EXOSC5** | **2,4** | NM_020158 | ILMN_1659725 |
| 896 | **C1orf57** | **2,4** | NM_032324 | ILMN_1657446 |
| 897 | **BTF3** | **2,4** | NM_001207 | ILMN_1676221 |
| 898 | **EEF1B2** | **2,4** | NM_001959 | ILMN_1701930 |
| 899 | **RBAF600** | **2,4** | NM_020765 | ILMN_1699226 |
| 900 | **MESP1** | **2,4** | NM_018670 | ILMN_1806603 |
| 901 | **PHLDA3** | **2,4** | NM_012396 | ILMN_1659106 |
| 902 | **NOLA2** | **2,4** | NM_017838 | ILMN_1718672 |
| 903 | **RABEPK** | **2,4** | NM_005833 | ILMN_1741957 |
| 904 | **PAK1IP1** | **2,4** | NM_017906 | ILMN_1726064 |
| 905 | **CKLF** | **2,4** | NM_016326 | ILMN_1712389 |
| 906 | **HSPC152** | **2,4** | NM_016404 | ILMN_1690802 |
| 907 | **MGC2803** | **2,4** | NM_024038 | ILMN_1691795 |
| 908 | **HCFC1R1** | **2,4** | NM_001002017 | ILMN_1757877 |
| 909 | **CLTB** | **2,4** | NM_001834 | ILMN_1674609 |
| 910 | **UXT** | **2,4** | NM_004182 | ILMN_1693687 |
| 911 | **ATP5J2** | **2,4** | NM_001003714 | ILMN_1750143 |
| 912 | **CSNK2B** | **2,4** | NM_001320 | ILMN_1800461 |
| 913 | **BXDC1** | **2,4** | NM_032194 | ILMN_1664167 |
| 914 | **DLNB14** | **2,4** | NM_198489 | ILMN_1709227 |
| 915 | **ITGAE** | **2,4** | NM_002208 | ILMN_1683927 |
| 916 | **HRAS** | **2,4** | NM_176795 | ILMN_1773751 |
| 917 | **RPL37A** | **2,4** | NM_000998 | ILMN_1711222 |
| 918 | **C10orf58** | **2,4** | NM_032333 | ILMN_1754538 |
| 919 | **SCAND1** | **2,4** | NM_033630 | ILMN_1794230 |
| 920 | **SCNM1** | **2,4** | NM_024041 | ILMN_1746598 |
| 921 | **38231** | **2,4** | NM_080417 | ILMN_1776157 |
| 922 | **BZRP** | **2,4** | NM_000714 | ILMN_1754663 |
| 923 | **EBPL** | **2,4** | NM_032565 | ILMN_1805922 |
| 924 | **FKBP11** | **2,4** | NM_016594 | ILMN_1787345 |
| 925 | **CNFN** | **2,4** | NM_032488 | ILMN_1803838 |
| 926 | **MGC22793** | **2,4** | NM_145030 | ILMN_1738514 |
| 927 | **C9orf23** | **2,4** | NM_148178 | ILMN_1683175 |
| 928 | **SEPW1** | **2,4** | NM_003009 | ILMN_1769226 |
| 929 | **POLR2G** | **2,4** | NM_002696 | ILMN_1748438 |
| 930 | **MRPL20** | **2,4** | NM_017971 | ILMN_1693352 |
| 931 | **ATP6V0B** | **2,4** | NM_004047 | ILMN_1721391 |
| 932 | **IGFL2** | **2,4** | NM_001002915 | ILMN_1745238 |
| 933 | **MDP-1** | **2,4** | NM_138476 | ILMN_1662263 |
| 934 | **SP5** | **2,4** | NM_001003845 | ILMN_1775956 |
| 935 | **RP9** | **2,4** | NM_203288 | ILMN_1687922 |
| 936 | **PPIB** | **2,4** | NM_000942 | ILMN_1711745 |
| 937 | **NUDT22** | **2,5** | NM_032344 | ILMN_1743582 |
| 938 | **TCEB1** | **2,5** | NM_005648 | ILMN_1704873 |
| 939 | **TNFSF7** | **2,5** | NM_001252 | ILMN_1760247 |
| 940 | **RAD51C** | **2,5** | NM_058216 | ILMN_1760635 |
| 941 | **UROD** | **2,5** | NM_000374 | ILMN_1740742 |
| 942 | **NME1-NME2** | **2,5** | NM_001018136 | ILMN_1693430 |
| 943 | **MGC5509** | **2,5** | NM_024093 | ILMN_1701131 |
| 944 | **ITGB1BP1** | **2,5** | NM_004763 | ILMN_1690099 |
| 945 | **PMVK** | **2,5** | NM_006556 | ILMN_1715896 |
| 946 | **ELOF1** | **2,5** | NM_032377 | ILMN_1723185 |
| 947 | **C6orf49** | **2,5** | NM_013397 | ILMN_1695868 |
| 948 | **TRA16** | **2,5** | NM_176880 | ILMN_1784512 |
| 949 | **NEDD8** | **2,5** | NM_006156 | ILMN_1785711 |
| 950 | **C19orf10** | **2,5** | NM_019107 | ILMN_1787844 |
| 951 | **BLOC1S1** | **2,5** | NM_001487 | ILMN_1767549 |
| 952 | **BCL2L12** | **2,5** | NM_052842 | ILMN_1752953 |
| 953 | **RPL17** | **2,5** | NM_000985 | ILMN_1701689 |
| 954 | **ZCSL2** | **2,5** | NM_206831 | ILMN_1679912 |
| 955 | **RGS10** | **2,5** | NM_002925 | ILMN_1733538 |
| 956 | **C7orf30** | **2,5** | NM_138446 | ILMN_1739798 |
| 957 | **C21orf70** | **2,5** | NM_058190 | ILMN_1751301 |
| 958 | **MGC13379** | **2,5** | NM_016499 | ILMN_1732577 |
| 959 | **MT** | **2,5** | NM_173467 | ILMN_1745513 |
| 960 | **AARSD1** | **2,5** | NM_025267 | ILMN_1700461 |
| 961 | **DSCR2** | **2,5** | NM_003720 | ILMN_1779264 |
| 962 | **ANAPC11** | **2,5** | NM_001002247 | ILMN_1722102 |
| 963 | **NUDT8** | **2,5** | NM_181843 | ILMN_1768712 |
| 964 | **FLCN** | **2,5** | NM_144997 | ILMN_1720555 |
| 965 | **APOC1** | **2,5** | NM_001645 | ILMN_1789007 |
| 966 | **RPS18** | **2,5** | NM_022551 | ILMN_1753534 |
| 967 | **ERCC1** | **2,5** | NM_001983 | ILMN_1797172 |
| 968 | **TRIAP1** | **2,5** | NM_016399 | ILMN_1774083 |
| 969 | **TIMM17B** | **2,5** | NM_005834 | ILMN_1813260 |
| 970 | **TMEM99** | **2,5** | NM_145274 | ILMN_1758173 |
| 971 | **GAD1** | **2,5** | NM_013445 | ILMN_1660973 |
| 972 | **ACN9** | **2,5** | NM_020186 | ILMN_1771348 |
| 973 | **EIF3S12** | **2,5** | NM_013234 | ILMN_1694057 |
| 974 | **INHBE** | **2,5** | NM_031479 | ILMN_1811767 |
| 975 | **CTXN1** | **2,5** | NM_206833 | ILMN_1759766 |
| 976 | **FIS1** | **2,5** | NM_016068 | ILMN_1658351 |
| 977 | **WDR74** | **2,5** | NM_018093 | ILMN_1789775 |
| 978 | **TCEB2** | **2,5** | NM_007108 | ILMN_1733927 |
| 979 | **DDT** | **2,5** | NM_001355 | ILMN_1690982 |
| 980 | **C1orf33** | **2,5** | NM_016183 | ILMN_1689800 |
| 981 | **CSTB** | **2,5** | NM_000100 | ILMN_1761797 |
| 982 | **D15Wsu75e** | **2,5** | NM_015704 | ILMN_1737580 |
| 983 | **TNPO2** | **2,5** | NM_013433 | ILMN_1656066 |
| 984 | **MGC13114** | **2,5** | NM_032366 | ILMN_1774990 |
| 985 | **C11orf17** | **2,6** | NM_020642 | ILMN_1788356 |
| 986 | **COX17** | **2,6** | NM_005694 | ILMN_1770885 |
| 987 | **LOC129531** | **2,6** | NM_138798 | ILMN_1677133 |
| 988 | **RPL35** | **2,6** | NM_007209 | ILMN_1775243 |
| 989 | **MGC17839** | **2,6** | NM_174926 | ILMN_1815346 |
| 990 | **POP5** | **2,6** | NM_015918 | ILMN_1687864 |
| 991 | **PDLIM7** | **2,6** | NM_213636 | ILMN_1690125 |
| 992 | **NDUFA12** | **2,6** | NM_018838 | ILMN_1803632 |
| 993 | **TMEM126A** | **2,6** | NM_032273 | ILMN_1679542 |
| 994 | **CALML4** | **2,6** | NM_001031733 | ILMN_1652389 |
| 995 | **C6orf125** | **2,6** | NM_032340 | ILMN_1790461 |
| 996 | **ZFAND2A** | **2,6** | NM_182491 | ILMN_1694671 |
| 997 | **BOLA2** | **2,6** | NM_001031827 | ILMN_1810680 |
| 998 | **PTRH1** | **2,6** | NM_001002913 | ILMN_1685240 |
| 999 | **MRPS34** | **2,6** | NM_023936 | ILMN_1780127 |
| 1000 | **SSBP1** | **2,6** | NM_003143 | ILMN_1809478 |
| 1001 | **C7orf21** | **2,6** | NM_031434 | ILMN_1666050 |
| 1002 | **ANKRD39** | **2,6** | NM_016466 | ILMN_1710979 |
| 1003 | **PTMS** | **2,6** | NM_002824 | ILMN_1721046 |
| 1004 | **HES6** | **2,6** | NM_018645 | ILMN_1694268 |
| 1005 | **ATP6V0C** | **2,6** | NM_001694 | ILMN_1773849 |
| 1006 | **SEC11L3** | **2,6** | NM_033280 | ILMN_1701681 |
| 1007 | **GLRX2** | **2,6** | NM_197962 | ILMN_1734903 |
| 1008 | **SPCS1** | **2,6** | NM_014041 | ILMN_1665280 |
| 1009 | **RPL9** | **2,6** | NM_001024921 | ILMN_1729033 |
| 1010 | **DUSP23** | **2,6** | NM_017823 | ILMN_1659462 |
| 1011 | **GPS2** | **2,6** | NM_004489 | ILMN_1815158 |
| 1012 | **MEA1** | **2,6** | NM_014623 | ILMN_1727073 |
| 1013 | **C12orf5** | **2,6** | NM_020375 | ILMN_1791792 |
| 1014 | **C1orf128** | **2,6** | NM_020362 | ILMN_1784207 |
| 1015 | **PXMP2** | **2,6** | NM_018663 | ILMN_1799015 |
| 1016 | **FAU** | **2,6** | NM_001997 | ILMN_1664614 |
| 1017 | **TRAPPC1** | **2,6** | NM_021210 | ILMN_1716913 |
| 1018 | **G10** | **2,6** | NM_003910 | ILMN_1710697 |
| 1019 | **TRG20** | **2,6** | NM_032286 | ILMN_1707631 |
| 1020 | **RNU3IP2** | **2,6** | NM_004704 | ILMN_1795758 |
| 1021 | **RPP21** | **2,6** | NM_024839 | ILMN_1717681 |
| 1022 | **SEC61B** | **2,6** | NM_006808 | ILMN_1736389 |
| 1023 | **RP11-529I10.4** | **2,7** | NM_015448 | ILMN_1744584 |
| 1024 | **NOLA1** | **2,7** | NM_032993 | ILMN_1681925 |
| 1025 | **NDUFA13** | **2,7** | NM_015965 | ILMN_1767139 |
| 1026 | **FLJ20512** | **2,7** | NM_017854 | ILMN_1704024 |
| 1027 | **C1orf50** | **2,7** | NM_024097 | ILMN_1801941 |
| 1028 | **TLCD1** | **2,7** | NM_138463 | ILMN_1784655 |
| 1029 | **ARPC5L** | **2,7** | NM_030978 | ILMN_1800844 |
| 1030 | **C18orf37** | **2,7** | NM_194281 | ILMN_1730294 |
| 1031 | **FLJ20186** | **2,7** | NM_017702 | ILMN_1656185 |
| 1032 | **ZNHIT1** | **2,7** | NM_006349 | ILMN_1741491 |
| 1033 | **CXorf33** | **2,7** | NM_198450 | ILMN_1777483 |
| 1034 | **SRM** | **2,7** | NM_003132 | ILMN_1661337 |
| 1035 | **RPL26L1** | **2,7** | NM_016093 | ILMN_1776586 |
| 1036 | **PLP2** | **2,7** | NM_002668 | ILMN_1738767 |
| 1037 | **MRPL46** | **2,7** | NM_022163 | ILMN_1722838 |
| 1038 | **PEX16** | **2,7** | NM_004813 | ILMN_1745655 |
| 1039 | **RPL8** | **2,7** | NM_033301 | ILMN_1811433 |
| 1040 | **UBL5** | **2,7** | NM_024292 | ILMN_1691379 |
| 1041 | **MAPBPIP** | **2,7** | NM_014017 | ILMN_1756352 |
| 1042 | **HSD17B8** | **2,7** | NM_014234 | ILMN_1715324 |
| 1043 | **POLR2H** | **2,7** | NM_006232 | ILMN_1689445 |
| 1044 | **C12orf24** | **2,7** | NM_013300 | ILMN_1753781 |
| 1045 | **KRTCAP2** | **2,7** | NM_173852 | ILMN_1658802 |
| 1046 | **MICA** | **2,7** | NM_000247 | ILMN_1655675 |
| 1047 | **POLR1C** | **2,7** | NM_004875 | ILMN_1767219 |
| 1048 | **MLC1SA** | **2,7** | NM_002475 | ILMN_1713450 |
| 1049 | **AK2** | **2,7** | NM_001625 | ILMN_1670542 |
| 1050 | **LOC348262** | **2,8** | NM_207368 | ILMN_1733799 |
| 1051 | **HAX1** | **2,8** | NM_001018837 | ILMN_1750658 |
| 1052 | **NPC2** | **2,8** | NM_006432 | ILMN_1716678 |
| 1053 | **GPX1** | **2,8** | NM_201397 | ILMN_1749662 |
| 1054 | **CLTA** | **2,8** | NM_007096 | ILMN_1695420 |
| 1055 | **MORN2** | **2,8** | NM_194270 | ILMN_1756826 |
| 1056 | **PYCR1** | **2,8** | NM_153824 | ILMN_1796013 |
| 1057 | **FKBP1A** | **2,8** | NM_054014 | ILMN_1702237 |
| 1058 | **GLIPR1** | **2,8** | NM_006851 | ILMN_1769245 |
| 1059 | **TUBB3** | **2,8** | NM_006086 | ILMN_1791726 |
| 1060 | **HSPC171** | **2,8** | NM_014187 | ILMN_1700419 |
| 1061 | **UCRC** | **2,8** | NM_001003684 | ILMN_1781986 |
| 1062 | **FAM96B** | **2,8** | NM_016062 | ILMN_1779813 |
| 1063 | **BOLA3** | **2,8** | NM_212552 | ILMN_1786658 |
| 1064 | **ICT1** | **2,8** | NM_001545 | ILMN_1734508 |
| 1065 | **RPL36** | **2,8** | NM_033643 | ILMN_1685088 |
| 1066 | **WBSCR22** | **2,8** | NM_017528 | ILMN_1697348 |
| 1067 | **HDDC3** | **2,8** | NM_198527 | ILMN_1781638 |
| 1068 | **NHP2L1** | **2,8** | NM_001003796 | ILMN_1697614 |
| 1069 | **MRPL41** | **2,8** | NM_032477 | ILMN_1705464 |
| 1070 | **MRPL23** | **2,8** | NM_021134 | ILMN_1806123 |
| 1071 | **NIFUN** | **2,8** | NM_014301 | ILMN_1735432 |
| 1072 | **CLDN2** | **2,8** | NM_020384 | ILMN_1795190 |
| 1073 | **FLJ20625** | **2,8** | NM_017907 | ILMN_1815878 |
| 1074 | **TMEM4** | **2,8** | NM_014255 | ILMN_1723586 |
| 1075 | **MRPL22** | **2,8** | NM_014180 | ILMN_1663220 |
| 1076 | **PTPLA** | **2,8** | NM_014241 | ILMN_1725791 |
| 1077 | **DPM3** | **2,8** | NM_018973 | ILMN_1673323 |
| 1078 | **EEF1D** | **2,8** | NM_032378 | ILMN_1782543 |
| 1079 | **DKFZp686O24166** | **2,8** | NM_001009913 | ILMN_1669972 |
| 1080 | **BTG2** | **2,8** | NM_006763 | ILMN_1770085 |
| 1081 | **ARL4** | **2,8** | NM_005738 | ILMN_1775405 |
| 1082 | **C16orf24** | **2,8** | NM_023933 | ILMN_1773780 |
| 1083 | **STEAP1** | **2,8** | NM_012449 | ILMN_1663575 |
| 1084 | **C21orf57** | **2,8** | NM_058181 | ILMN_1795836 |
| 1085 | **NOLA3** | **2,8** | NM_018648 | ILMN_1815479 |
| 1086 | **SNRPB2** | **2,8** | NM_003092 | ILMN_1771620 |
| 1087 | **CHMP2A** | **2,9** | NM_198426 | ILMN_1762932 |
| 1088 | **ZD52F10** | **2,9** | NM_033317 | ILMN_1778319 |
| 1089 | **COMMD1** | **2,9** | NM_152516 | ILMN_1761242 |
| 1090 | **DLL3** | **2,9** | NM_016941 | ILMN_1736096 |
| 1091 | **C9orf142** | **2,9** | NM_183241 | ILMN_1761138 |
| 1092 | **NDUFA3** | **2,9** | NM_004542 | ILMN_1784641 |
| 1093 | **NUDT14** | **2,9** | NM_177533 | ILMN_1669788 |
| 1094 | **SH2D2A** | **2,9** | NM_003975 | ILMN_1766319 |
| 1095 | **PTRH2** | **2,9** | NM_016077 | ILMN_1811594 |
| 1096 | **FJX1** | **2,9** | NM_014344 | ILMN_1746465 |
| 1097 | **ZNF581** | **2,9** | NM_016535 | ILMN_1679093 |
| 1098 | **TMEM93** | **2,9** | NM_001014764 | ILMN_1758674 |
| 1099 | **MRPL14** | **2,9** | NM_032111 | ILMN_1727004 |
| 1100 | **BLOC1S2** | **2,9** | NM_001001342 | ILMN_1679782 |
| 1101 | **DCXR** | **2,9** | NM_016286 | ILMN_1681437 |
| 1102 | **PHLDA1** | **2,9** | NM_007350 | ILMN_1687978 |
| 1103 | **RIP** | **2,9** | NM_001033002 | ILMN_1770339 |
| 1104 | **PUSL1** | **2,9** | NM_153339 | ILMN_1780315 |
| 1105 | **C19orf24** | **2,9** | NM_017914 | ILMN_1678052 |
| 1106 | **RPS19BP1** | **2,9** | NM_194326 | ILMN_1702059 |
| 1107 | **GEMIN6** | **2,9** | NM_024775 | ILMN_1707484 |
| 1108 | **ALKBH2** | **2,9** | NM_001001655 | ILMN_1690252 |
| 1109 | **NDUFB9** | **2,9** | NM_005005 | ILMN_1666326 |
| 1110 | **H2BFS** | **2,9** | NM_017445 | ILMN_1813314 |
| 1111 | **PDCD5** | **2,9** | NM_004708 | ILMN_1668425 |
| 1112 | **NDUFB6** | **2,9** | NM_002493 | ILMN_1763147 |
| 1113 | **PRR7** | **2,9** | NM_030567 | ILMN_1677509 |
| 1114 | **EDF1** | **3,0** | NM_003792 | ILMN_1696544 |
| 1115 | **MRPL53** | **3,0** | NM_053050 | ILMN_1813682 |
| 1116 | **IMP4** | **3,0** | NM_033416 | ILMN_1661347 |
| 1117 | **NDUFB7** | **3,0** | NM_004146 | ILMN_1813604 |
| 1118 | **MRPS21** | **3,0** | NM_018997 | ILMN_1660292 |
| 1119 | **NDUFS7** | **3,0** | NM_024407 | ILMN_1669966 |
| 1120 | **RRAGD** | **3,0** | NM_021244 | ILMN_1699772 |
| 1121 | **KRT10** | **3,0** | NM_000421 | ILMN_1716093 |
| 1122 | **CMTM8** | **3,0** | NM_178868 | ILMN_1710124 |
| 1123 | **MRPL55** | **3,0** | NM_181464 | ILMN_1813817 |
| 1124 | **SAT2** | **3,0** | NM_133491 | ILMN_1746883 |
| 1125 | **PFDN6** | **3,0** | NM_014260 | ILMN_1661490 |
| 1126 | **NDUFC1** | **3,0** | NM_002494 | ILMN_1733603 |
| 1127 | **FOSL1** | **3,0** | NM_005438 | ILMN_1771841 |
| 1128 | **ATP6V1F** | **3,0** | NM_004231 | ILMN_1678308 |
| 1129 | **MRPS15** | **3,0** | NM_031280 | ILMN_1680703 |
| 1130 | **MRPL54** | **3,0** | NM_172251 | ILMN_1658486 |
| 1131 | **AURKAIP1** | **3,1** | NM_017900 | ILMN_1700793 |
| 1132 | **NDUFA7** | **3,1** | NM_005001 | ILMN_1675239 |
| 1133 | **HSPC268** | **3,1** | NM_197964 | ILMN_1779751 |
| 1134 | **ATP6V0E** | **3,1** | NM_003945 | ILMN_1715635 |
| 1135 | **HIGD2A** | **3,1** | NM_138820 | ILMN_1774334 |
| 1136 | **NIFIE14** | **3,1** | NM_032635 | ILMN_1803624 |
| 1137 | **MRPS24** | **3,1** | NM_032014 | ILMN_1802553 |
| 1138 | **C6orf108** | **3,1** | NM_199184 | ILMN_1769343 |
| 1139 | **RANGNRF** | **3,1** | NM_016492 | ILMN_1745760 |
| 1140 | **POLR2L** | **3,1** | NM_021128 | ILMN_1670037 |
| 1141 | **UCHL3** | **3,1** | NM_006002 | ILMN_1660111 |
| 1142 | **BAX** | **3,1** | NM_004324 | ILMN_1683300 |
| 1143 | **CHCHD5** | **3,1** | NM_032309 | ILMN_1797530 |
| 1144 | **HADH2** | **3,1** | NM_004493 | ILMN_1758275 |
| 1145 | **CHCHD8** | **3,1** | NM_016565 | ILMN_1732750 |
| 1146 | **MGC15416** | **3,1** | NM_138418 | ILMN_1730523 |
| 1147 | **TIMP1** | **3,1** | NM_003254 | ILMN_1711566 |
| 1148 | **SSR4** | **3,1** | NM_006280 | ILMN_1680403 |
| 1149 | **ATP5I** | **3,1** | NM_007100 | ILMN_1803509 |
| 1150 | **MGC2408** | **3,2** | NM_032331 | ILMN_1762883 |
| 1151 | **EMG1** | **3,2** | NM_006331 | ILMN_1797074 |
| 1152 | **CKS2** | **3,2** | NM_001827 | ILMN_1756326 |
| 1153 | **MT2A** | **3,2** | NM_005953 | ILMN_1690957 |
| 1154 | **HSPC023** | **3,2** | NM_014047 | ILMN_1671374 |
| 1155 | **PCBD1** | **3,2** | NM_001001939 | ILMN_1708813 |
| 1156 | **RPS7** | **3,2** | NM_001011 | ILMN_1750722 |
| 1157 | **MAGOH** | **3,2** | NM_002370 | ILMN_1814396 |
| 1158 | **FAM86B1** | **3,2** | NM_032916 | ILMN_1655051 |
| 1159 | **LSM7** | **3,2** | NM_016199 | ILMN_1678165 |
| 1160 | **SOD2** | **3,2** | NM_001024465 | ILMN_1775672 |
| 1161 | **RPS21** | **3,3** | NM_001024 | ILMN_1800573 |
| 1162 | **IL32** | **3,3** | NM_001012632 | ILMN_1778010 |
| 1163 | **MRPL21** | **3,3** | NM_181513 | ILMN_1744835 |
| 1164 | **EXOSC4** | **3,3** | NM_019037 | ILMN_1745271 |
| 1165 | **LSM10** | **3,3** | NM_032881 | ILMN_1751803 |
| 1166 | **IER3** | **3,3** | NM_052815 | ILMN_1682717 |
| 1167 | **MRPL12** | **3,3** | NM_002949 | ILMN_1699603 |
| 1168 | **NDUFA11** | **3,3** | NM_175614 | ILMN_1682299 |
| 1169 | **MRPL40** | **3,3** | NM_003776 | ILMN_1687403 |
| 1170 | **SF3B5** | **3,3** | NM_031287 | ILMN_1689389 |
| 1171 | **IFI30** | **3,3** | NM_006332 | ILMN_1682846 |
| 1172 | **MRPL2** | **3,3** | NM_015950 | ILMN_1763264 |
| 1173 | **RPL14** | **3,3** | NM_003973 | ILMN_1726460 |
| 1174 | **NSMCE1** | **3,3** | NM_145080 | ILMN_1697962 |
| 1175 | **DXS9879E** | **3,3** | NM_006014 | ILMN_1708151 |
| 1176 | **TIMM10** | **3,3** | NM_012456 | ILMN_1765332 |
| 1177 | **MT1A** | **3,3** | NM_005946 | ILMN_1691156 |
| 1178 | **PCGF1** | **3,3** | NM_032673 | ILMN_1757956 |
| 1179 | **LOC283951** | **3,3** | NM_001010878 | ILMN_1759325 |
| 1180 | **POLR2F** | **3,3** | NM_021974 | ILMN_1745885 |
| 1181 | **MGC71993** | **3,3** | NM_001004333 | ILMN_1715698 |
| 1182 | **ZNF342** | **3,3** | NM_145288 | ILMN_1693242 |
| 1183 | **HSPC176** | **3,3** | NM_016209 | ILMN_1747058 |
| 1184 | **DHRS9** | **3,4** | NM_005771 | ILMN_1733998 |
| 1185 | **NME1** | **3,4** | NM_198175 | ILMN_1713875 |
| 1186 | **NKD1** | **3,4** | NM_033119 | ILMN_1692674 |
| 1187 | **MRPS18C** | **3,4** | NM_016067 | ILMN_1658416 |
| 1188 | **SERF2** | **3,4** | NM_001018108 | ILMN_1789136 |
| 1189 | **TAF10** | **3,4** | NM_006284 | ILMN_1721093 |
| 1190 | **LOC389541** | **3,4** | NM_001008395 | ILMN_1779735 |
| 1191 | **SLC27A5** | **3,4** | NM_012254 | ILMN_1725366 |
| 1192 | **QIL1** | **3,4** | NM_205767 | ILMN_1765684 |
| 1193 | **POLR3K** | **3,4** | NM_016310 | ILMN_1801664 |
| 1194 | **EGR1** | **3,4** | NM_001964 | ILMN_1762899 |
| 1195 | **RPL13** | **3,4** | NM_033251 | ILMN_1709039 |
| 1196 | **MRPS17** | **3,5** | NM_015969 | ILMN_1804851 |
| 1197 | **XAGE1** | **3,5** | NM_020411 | ILMN_1691494 |
| 1198 | **TIMM9** | **3,5** | NM_012460 | ILMN_1653709 |
| 1199 | **S100A11** | **3,5** | NM_005620 | ILMN_1750101 |
| 1200 | **TM4SF19** | **3,5** | NM_138461 | ILMN_1808325 |
| 1201 | **C14orf2** | **3,5** | NM_004894 | ILMN_1652722 |
| 1202 | **CUTA** | **3,5** | NM_001014433 | ILMN_1712390 |
| 1203 | **RPL34** | **3,5** | NM_033625 | ILMN_1774823 |
| 1204 | **ORF1-FL49** | **3,5** | NM_032412 | ILMN_1761566 |
| 1205 | **HINT2** | **3,5** | NM_032593 | ILMN_1697820 |
| 1206 | **mimitin** | **3,5** | NM_174889 | ILMN_1682857 |
| 1207 | **CDKN1A** | **3,6** | NM_078467 | ILMN_1787212 |
| 1208 | **GADD45A** | **3,6** | NM_001924 | ILMN_1694075 |
| 1209 | **RPS15** | **3,6** | NM_001018 | ILMN_1691807 |
| 1210 | **ARD1A** | **3,6** | NM_003491 | ILMN_1721977 |
| 1211 | **POLR2I** | **3,7** | NM_006233 | ILMN_1720542 |
| 1212 | **MRPL36** | **3,7** | NM_032479 | ILMN_1800197 |
| 1213 | **ATOX1** | **3,7** | NM_004045 | ILMN_1670609 |
| 1214 | **MRPS12** | **3,7** | NM_033363 | ILMN_1714515 |
| 1215 | **ATP5D** | **3,7** | NM_001001975 | ILMN_1653599 |
| 1216 | **LOC205251** | **3,7** | NM_174925 | ILMN_1693685 |
| 1217 | **OCIAD2** | **3,7** | NM_152398 | ILMN_1772286 |
| 1218 | **ATP5G1** | **3,8** | NM_005175 | ILMN_1712430 |
| 1219 | **MRPL27** | **3,8** | NM_016504 | ILMN_1811327 |
| 1220 | **SLC39A4** | **3,8** | NM_017767 | ILMN_1706386 |
| 1221 | **NEU1** | **3,8** | NM_000434 | ILMN_1763144 |
| 1222 | **MPV17** | **3,9** | NM_002437 | ILMN_1691090 |
| 1223 | **PRDX5** | **3,9** | NM_012094 | ILMN_1815024 |
| 1224 | **UPP1** | **3,9** | NM_181597 | ILMN_1798256 |
| 1225 | **C17orf79** | **4,0** | NM_018405 | ILMN_1752947 |
| 1226 | **APRT** | **4,0** | NM_001030018 | ILMN_1726410 |
| 1227 | **ZNRD1** | **4,1** | NM_170783 | ILMN_1692486 |
| 1228 | **HSPC111** | **4,1** | NM_016391 | ILMN_1704055 |
| 1229 | **DRAP1** | **4,1** | NM_006442 | ILMN_1733048 |
| 1230 | **MRPL34** | **4,1** | NM_023937 | ILMN_1783681 |
| 1231 | **Magmas** | **4,1** | NM_016069 | ILMN_1763884 |
| 1232 | **HBQ1** | **4,2** | NM_005331 | ILMN_1696183 |
| 1233 | **C12orf45** | **4,2** | NM_152318 | ILMN_1728435 |
| 1234 | **C11orf48** | **4,3** | NM_024099 | ILMN_1739345 |
| 1235 | **RPS29** | **4,3** | NM_001030001 | ILMN_1719476 |
| 1236 | **LOC352909** | **4,4** | NM_001031802 | ILMN_1772074 |
| 1237 | **GDF15** | **4,4** | NM_004864 | ILMN_1763658 |
| 1238 | **C22orf16** | **4,6** | NM_213720 | ILMN_1740170 |
| 1239 | **PSMB10** | **4,8** | NM_002801 | ILMN_1683026 |
| 1240 | **FLJ25801** | **4,8** | NM_173553 | ILMN_1666893 |
| 1241 | **RBP1** | **4,8** | NM_002899 | ILMN_1656837 |
| 1242 | **TNFRSF12A** | **4,9** | NM_016639 | ILMN_1689004 |
| 1243 | **EMP3** | **5,2** | NM_001425 | ILMN_1765446 |
| 1244 | **MT1X** | **5,2** | NM_005952 | ILMN_1775170 |
| 1245 | **SFN** | **5,3** | NM_006142 | ILMN_1806607 |
| 1246 | **TIGA1** | **5,3** | NM_053000 | ILMN_1654609 |
| 1247 | **ZNF593** | **5,6** | NM_015871 | ILMN_1703441 |
| 1248 | **VGF** | **5,7** | NM_003378 | ILMN_1757497 |
| 1249 | **C6orf1** | **6,0** | NM_001008703 | ILMN_1744196 |
| 1250 | **BRI3** | **6,3** | NM_015379 | ILMN_1781580 |
| 1251 | **DDIT3** | **6,8** | NM_004083 | ILMN_1676984 |
| 1252 | **AXIN2** | **6,8** | NM_004655 | ILMN_1724480 |
| 1253 | **IL8** | **7,0** | NM_000584 | ILMN_1666733 |
| 1254 | **CST1** | **7,0** | NM_001898 | ILMN_1753449 |
| 1255 | **CALCB** | **11,8** | NM_000728 | ILMN_1694427 |
