# Supplementary material for: Enzastaurin inhibits invasion and metastasis in lung cancer by diverse molecules
Source: Br J Cancer. 2010 Aug 24;103(6):802–11. doi: 10.1038/sj.bjc.6605818 (PMC2966618; doi:10.1038/sj.bjc.6605818)
Supplement: Supplementary Table 3 [file 6605818x7.doc]

**Supplementary Table 3**

**Significantly (> 1.5 fold) deregulated genes after 48 hours of Enz-treatment**

| **S.No** | **Symbol** | **Fold Change** | **Genbank** | **Probe_ID (ILL)** |
| --- | --- | --- | --- | --- |
| 1 | **TM4SF20** | **-28,7** | NM_024795 | ILMN_1775830 |
| 2 | **GALNT4** | **-20,9** | NM_003774 | ILMN_1739297 |
| 3 | **HLA-DMB** | **-19,9** | NM_002118 | ILMN_1761733 |
| 4 | **FGB** | **-18,8** | NM_005141 | ILMN_1678049 |
| 5 | **MXD4** | **-17,6** | NM_006454 | ILMN_1756541 |
| 6 | **PDXK** | **-16,5** | NM_003681 | ILMN_1672504 |
| 7 | **TXNIP** | **-15,5** | NM_006472 | ILMN_1697448 |
| 8 | **FGA** | **-15,2** | NM_021871 | ILMN_1656487 |
| 9 | **RAP1GA1** | **-14,3** | NM_002885 | ILMN_1776519 |
| 10 | **FLRT3** | **-13,4** | NM_013281 | ILMN_1805665 |
| 11 | **IGFBP3** | **-13,0** | NM_001013398 | ILMN_1746085 |
| 12 | **CP** | **-12,8** | NM_000096 | ILMN_1739608 |
| 13 | **CYP24A1** | **-12,3** | NM_000782 | ILMN_1685663 |
| 14 | **TF** | **-12,2** | NM_001063 | ILMN_1768425 |
| 15 | **SLC38A2** | **-12,0** | NM_018976 | ILMN_1651799 |
| 16 | **BNIP3L** | **-12,0** | NM_004331 | ILMN_1718961 |
| 17 | **CALM1** | **-11,9** | NM_006888 | ILMN_1778242 |
| 18 | **PAFAH1B1** | **-11,8** | NM_000430 | ILMN_1722276 |
| 19 | **TSPAN8** | **-11,7** | NM_004616 | ILMN_1683263 |
| 20 | **TFPI** | **-11,7** | NM_001032281 | ILMN_1662619 |
| 21 | **IGFBP1** | **-11,6** | NM_000596 | ILMN_1728445 |
| 22 | **SCNN1A** | **-11,6** | NM_001038 | ILMN_1713995 |
| 23 | **PTPNS1** | **-10,8** | NM_080792 | ILMN_1758146 |
| 24 | **LGR4** | **-10,1** | NM_018490 | ILMN_1686895 |
| 25 | **TNS3** | **-9,7** | NM_022748 | ILMN_1667893 |
| 26 | **SLC7A5** | **-9,7** | NM_003486 | ILMN_1720373 |
| 27 | **TM4SF4** | **-9,6** | NM_004617 | ILMN_1792404 |
| 28 | **ALS2CR13** | **-9,6** | NM_173511 | ILMN_1739942 |
| 29 | **HBP1** | **-9,6** | NM_012257 | ILMN_1685415 |
| 30 | **LRP11** | **-9,3** | NM_032832 | ILMN_1676197 |
| 31 | **IQWD1** | **-9,2** | NM_018442 | ILMN_1670000 |
| 32 | **CEACAM6** | **-9,0** | NM_002483 | ILMN_1712522 |
| 33 | **SMAD6** | **-9,0** | NM_005585 | ILMN_1767068 |
| 34 | **FOXQ1** | **-9,0** | NM_033260 | ILMN_1669046 |
| 35 | **RNASE4** | **-8,7** | NM_194430 | ILMN_1696974 |
| 36 | **TMEM2** | **-8,6** | NM_013390 | ILMN_1784661 |
| 37 | **RNF103** | **-8,4** | NM_005667 | ILMN_1752677 |
| 38 | **ANG** | **-8,4** | NM_001145 | ILMN_1760727 |
| 39 | **TACC2** | **-8,1** | NM_006997 | ILMN_1754407 |
| 40 | **GPT2** | **-8,0** | NM_133443 | ILMN_1750527 |
| 41 | **PDE4D** | **-8,0** | NM_006203 | ILMN_1791483 |
| 42 | **INSIG1** | **-8,0** | NM_005542 | ILMN_1793474 |
| 43 | **C20orf177** | **-7,9** | NM_022106 | ILMN_1718712 |
| 44 | **RNF19** | **-7,9** | NM_015435 | ILMN_1673875 |
| 45 | **ULK1** | **-7,9** | NM_003565 | ILMN_1735052 |
| 46 | **VAV3** | **-7,8** | NM_006113 | ILMN_1657679 |
| 47 | **LDLR** | **-7,6** | NM_000527 | ILMN_1651611 |
| 48 | **TM4SF1** | **-7,6** | NM_014220 | ILMN_1770338 |
| 49 | **ERBB3** | **-7,4** | NM_001982 | ILMN_1751346 |
| 50 | **FGL1** | **-7,3** | NM_201553 | ILMN_1682828 |
| 51 | **GALNAC4S-6ST** | **-7,0** | NM_015892 | ILMN_1670926 |
| 52 | **FOXA1** | **-6,9** | NM_004496 | ILMN_1766650 |
| 53 | **PGRMC2** | **-6,7** | NM_006320 | ILMN_1711682 |
| 54 | **C9orf152** | **-6,6** | NM_001012993 | ILMN_1781745 |
| 55 | **JUP** | **-6,6** | NM_021991 | ILMN_1733811 |
| 56 | **NCOA7** | **-6,5** | NM_181782 | ILMN_1687768 |
| 57 | **PAPSS2** | **-6,5** | NM_001015880 | ILMN_1668675 |
| 58 | **C1orf116** | **-6,4** | NM_023938 | ILMN_1706483 |
| 59 | **SPSB3** | **-6,3** | NM_080861 | ILMN_1682864 |
| 60 | **AGR2** | **-6,3** | NM_006408 | ILMN_1814151 |
| 61 | **MB** | **-6,2** | NM_005368 | ILMN_1666109 |
| 62 | **SERPINA6** | **-6,1** | NM_001756 | ILMN_1682021 |
| 63 | **LPP** | **-6,1** | NM_005578 | ILMN_1651254 |
| 64 | **ELF3** | **-6,0** | NM_004433 | ILMN_1769201 |
| 65 | **STS-1** | **-6,0** | NM_032873 | ILMN_1653856 |
| 66 | **ZBED1** | **-5,9** | NM_004729 | ILMN_1694466 |
| 67 | **ZNF161** | **-5,9** | NM_007146 | ILMN_1705310 |
| 68 | **SGK** | **-5,9** | NM_005627 | ILMN_1702487 |
| 69 | **HPS6** | **-5,8** | NM_024747 | ILMN_1718537 |
| 70 | **FAM113B** | **-5,8** | NM_138371 | ILMN_1712431 |
| 71 | **RASSF7** | **-5,8** | NM_003475 | ILMN_1733110 |
| 72 | **CA12** | **-5,8** | NM_001218 | ILMN_1720998 |
| 73 | **VPS54** | **-5,8** | NM_016516 | ILMN_1761086 |
| 74 | **IXL** | **-5,8** | NM_017592 | ILMN_1728360 |
| 75 | **FAM100B** | **-5,8** | NM_182565 | ILMN_1782778 |
| 76 | **ANXA4** | **-5,7** | NM_001153 | ILMN_1711408 |
| 77 | **ALDH3A2** | **-5,7** | NM_001031806 | ILMN_1794825 |
| 78 | **HIG2** | **-5,7** | NM_013332 | ILMN_1659990 |
| 79 | **GAL3ST1** | **-5,6** | NM_004861 | ILMN_1729905 |
| 80 | **RICTOR** | **-5,6** | NM_152756 | ILMN_1727171 |
| 81 | **GATS** | **-5,5** | NM_178831 | ILMN_1699631 |
| 82 | **DAPK1** | **-5,5** | NM_004938 | ILMN_1708340 |
| 83 | **KIAA1370** | **-5,5** | NM_019600 | ILMN_1700733 |
| 84 | **BTG1** | **-5,5** | NM_001731 | ILMN_1775743 |
| 85 | **ST6GAL1** | **-5,5** | NM_173217 | ILMN_1756501 |
| 86 | **ASF1A** | **-5,5** | NM_014034 | ILMN_1658285 |
| 87 | **PPARGC1A** | **-5,4** | NM_013261 | ILMN_1750062 |
| 88 | **LOC58486** | **-5,4** | NM_021211 | ILMN_1664424 |
| 89 | **LOC51315** | **-5,4** | NM_016618 | ILMN_1745620 |
| 90 | **STARD7** | **-5,4** | NM_020151 | ILMN_1774415 |
| 91 | **MGC16385** | **-5,3** | NM_145039 | ILMN_1692072 |
| 92 | **NARF** | **-5,3** | NM_031968 | ILMN_1726884 |
| 93 | **TMCO3** | **-5,3** | NM_017905 | ILMN_1654748 |
| 94 | **CDH19** | **-5,3** | NM_021153 | ILMN_1787825 |
| 95 | **CYP26B1** | **-5,3** | NM_019885 | ILMN_1812297 |
| 96 | **SEPX1** | **-5,3** | NM_016332 | ILMN_1719661 |
| 97 | **DUSP4** | **-5,3** | NM_057158 | ILMN_1734387 |
| 98 | **ZA20D2** | **-5,2** | NM_006007 | ILMN_1795228 |
| 99 | **RHOV** | **-5,2** | NM_133639 | ILMN_1698484 |
| 100 | **DOK4** | **-5,2** | NM_018110 | ILMN_1774261 |
| 101 | **HSPA2** | **-5,2** | NM_021979 | ILMN_1766499 |
| 102 | **SLC16A4** | **-5,1** | NM_004696 | ILMN_1804673 |
| 103 | **FKSG44** | **-5,1** | NM_031904 | ILMN_1781047 |
| 104 | **CCND1** | **-5,1** | NM_053056 | ILMN_1688480 |
| 105 | **CDC42EP4** | **-5,1** | NM_012121 | ILMN_1745223 |
| 106 | **PTP4A1** | **-5,1** | NM_003463 | ILMN_1760575 |
| 107 | **EDN1** | **-5,1** | NM_001955 | ILMN_1682775 |
| 108 | **FRAT2** | **-5,0** | NM_012083 | ILMN_1788213 |
| 109 | **FZD2** | **-5,0** | NM_001466 | ILMN_1653711 |
| 110 | **REEP5** | **-5,0** | NM_005669 | ILMN_1758941 |
| 111 | **HERPUD1** | **-5,0** | NM_001010990 | ILMN_1700346 |
| 112 | **SREBF1** | **-4,9** | NM_004176 | ILMN_1663035 |
| 113 | **HOXA5** | **-4,9** | NM_019102 | ILMN_1753613 |
| 114 | **ITSN1** | **-4,9** | NM_001001132 | ILMN_1718769 |
| 115 | **ASPM** | **-4,9** | NM_018136 | ILMN_1815184 |
| 116 | **DHRS3** | **-4,9** | NM_004753 | ILMN_1752478 |
| 117 | **FLJ12505** | **-4,9** | NM_024749 | ILMN_1692698 |
| 118 | **RHOQ** | **-4,9** | NM_012249 | ILMN_1810559 |
| 119 | **ANGPTL4** | **-4,9** | NM_139314 | ILMN_1707727 |
| 120 | **KIAA0999** | **-4,9** | NM_025164 | ILMN_1732343 |
| 121 | **C14orf108** | **-4,9** | NM_018229 | ILMN_1741331 |
| 122 | **NRIP1** | **-4,9** | NM_003489 | ILMN_1718629 |
| 123 | **PDK4** | **-4,8** | NM_002612 | ILMN_1684982 |
| 124 | **USP9X** | **-4,8** | NM_004652 | ILMN_1656165 |
| 125 | **C2orf30** | **-4,8** | NM_015701 | ILMN_1724376 |
| 126 | **HMGB3** | **-4,8** | NM_005342 | ILMN_1733519 |
| 127 | **PUM2** | **-4,8** | NM_015317 | ILMN_1722345 |
| 128 | **FAM107B** | **-4,8** | NM_031453 | ILMN_1758672 |
| 129 | **GABRB3** | **-4,8** | NM_000814 | ILMN_1709681 |
| 130 | **CYP4F3** | **-4,8** | NM_000896 | ILMN_1736190 |
| 131 | **FBXO21** | **-4,8** | NM_015002 | ILMN_1745887 |
| 132 | **IGSF3** | **-4,8** | NM_001542 | ILMN_1713014 |
| 133 | **KLF2** | **-4,8** | NM_016270 | ILMN_1735930 |
| 134 | **TRIAD3** | **-4,7** | NM_019011 | ILMN_1729980 |
| 135 | **ZNF212** | **-4,7** | NM_012256 | ILMN_1684964 |
| 136 | **ANK3** | **-4,7** | NM_001149 | ILMN_1657016 |
| 137 | **IREB2** | **-4,7** | NM_004136 | ILMN_1726554 |
| 138 | **MGC5242** | **-4,7** | NM_024033 | ILMN_1740903 |
| 139 | **C5orf21** | **-4,7** | NM_032042 | ILMN_1654542 |
| 140 | **FGFR3** | **-4,7** | NM_000142 | ILMN_1723123 |
| 141 | **PER2** | **-4,7** | NM_022817 | ILMN_1738095 |
| 142 | **CCND3** | **-4,7** | NM_001760 | ILMN_1668721 |
| 143 | **PDPR** | **-4,7** | NM_017990 | ILMN_1667034 |
| 144 | **IRS2** | **-4,7** | NM_003749 | ILMN_1763809 |
| 145 | **MANSC1** | **-4,7** | NM_018050 | ILMN_1652490 |
| 146 | **UBE4B** | **-4,7** | NM_006048 | ILMN_1675674 |
| 147 | **C17orf59** | **-4,7** | NM_017622 | ILMN_1789643 |
| 148 | **MGC24039** | **-4,7** | NM_144973 | ILMN_1791593 |
| 149 | **RAI17** | **-4,7** | NM_020338 | ILMN_1771627 |
| 150 | **SFRS6** | **-4,7** | NM_006275 | ILMN_1697469 |
| 151 | **GPX2** | **-4,6** | NM_002083 | ILMN_1662776 |
| 152 | **PTRF** | **-4,6** | NM_012232 | ILMN_1757552 |
| 153 | **CAV2** | **-4,6** | NM_001233 | ILMN_1658835 |
| 154 | **SEPHS2** | **-4,6** | NM_012248 | ILMN_1687824 |
| 155 | **EEF2K** | **-4,6** | NM_013302 | ILMN_1789171 |
| 156 | **C8orf55** | **-4,6** | NM_016647 | ILMN_1777740 |
| 157 | **FNDC3B** | **-4,5** | NM_022763 | ILMN_1753554 |
| 158 | **UBE2J1** | **-4,5** | NM_016021 | ILMN_1713759 |
| 159 | **CXXC5** | **-4,5** | NM_016463 | ILMN_1745256 |
| 160 | **ANXA13** | **-4,5** | NM_001003954 | ILMN_1737612 |
| 161 | **GLCE** | **-4,5** | NM_015554 | ILMN_1714349 |
| 162 | **CTDSP2** | **-4,5** | NM_005730 | ILMN_1692962 |
| 163 | **CDC2L6** | **-4,5** | NM_015076 | ILMN_1676891 |
| 164 | **NBLA04196** | **-4,5** | NM_022900 | ILMN_1668699 |
| 165 | **NET1** | **-4,4** | NM_005863 | ILMN_1758311 |
| 166 | **NTS** | **-4,4** | NM_006183 | ILMN_1764690 |
| 167 | **CD55** | **-4,4** | NM_000574 | ILMN_1800540 |
| 168 | **DDX5** | **-4,4** | NM_004396 | ILMN_1805344 |
| 169 | **GPR37** | **-4,4** | NM_005302 | ILMN_1668271 |
| 170 | **FAM84B** | **-4,4** | NM_174911 | ILMN_1670807 |
| 171 | **RHOU** | **-4,4** | NM_021205 | ILMN_1748992 |
| 172 | **RTKN** | **-4,4** | NM_001015055 | ILMN_1680591 |
| 173 | **PAPPA** | **-4,3** | NM_002581 | ILMN_1721770 |
| 174 | **BCL6** | **-4,3** | NM_001706 | ILMN_1737314 |
| 175 | **ZCCHC14** | **-4,3** | NM_015144 | ILMN_1743456 |
| 176 | **SPHK2** | **-4,3** | NM_020126 | ILMN_1729281 |
| 177 | **KIAA1279** | **-4,3** | NM_015634 | ILMN_1745813 |
| 178 | **CNTN1** | **-4,3** | NM_001843 | ILMN_1728853 |
| 179 | **HNRPH3** | **-4,3** | NM_012207 | ILMN_1654920 |
| 180 | **ALDH5A1** | **-4,3** | NM_001080 | ILMN_1715859 |
| 181 | **UGDH** | **-4,3** | NM_003359 | ILMN_1729563 |
| 182 | **PLSCR4** | **-4,3** | NM_020353 | ILMN_1757338 |
| 183 | **DKK1** | **-4,3** | NM_012242 | ILMN_1773337 |
| 184 | **RTN4RL2** | **-4,2** | NM_178570 | ILMN_1725098 |
| 185 | **LOC400566** | **-4,2** | NM_001013672 | ILMN_1707137 |
| 186 | **CTDSP1** | **-4,2** | NM_021198 | ILMN_1728163 |
| 187 | **CEACAM1** | **-4,2** | NM_001024912 | ILMN_1716815 |
| 188 | **KLHDC2** | **-4,2** | NM_014315 | ILMN_1741204 |
| 189 | **CXCL16** | **-4,2** | NM_022059 | ILMN_1728478 |
| 190 | **C21orf25** | **-4,2** | NM_199050 | ILMN_1652512 |
| 191 | **OSTbeta** | **-4,2** | NM_178859 | ILMN_1750312 |
| 192 | **MUC1** | **-4,1** | NM_001018021 | ILMN_1756992 |
| 193 | **RASSF2** | **-4,1** | NM_170773 | ILMN_1682212 |
| 194 | **TNFRSF1A** | **-4,1** | NM_001065 | ILMN_1685005 |
| 195 | **KLHDC3** | **-4,1** | NM_057161 | ILMN_1730940 |
| 196 | **JMJD1C** | **-4,1** | NM_004241 | ILMN_1764970 |
| 197 | **SUOX** | **-4,1** | NM_001032386 | ILMN_1803745 |
| 198 | **ASXL2** | **-4,1** | NM_018263 | ILMN_1698968 |
| 199 | **OVGP1** | **-4,1** | NM_002557 | ILMN_1734542 |
| 200 | **EMILIN2** | **-4,1** | NM_032048 | ILMN_1697268 |
| 201 | **FLJ13236** | **-4,0** | NM_024902 | ILMN_1749636 |
| 202 | **INPP5A** | **-4,0** | NM_005539 | ILMN_1735224 |
| 203 | **C20orf11** | **-4,0** | NM_017896 | ILMN_1720188 |
| 204 | **ARMC7** | **-4,0** | NM_024585 | ILMN_1797298 |
| 205 | **BMP4** | **-4,0** | NM_001202 | ILMN_1740900 |
| 206 | **LOC201895** | **-4,0** | NM_174921 | ILMN_1713892 |
| 207 | **C6orf62** | **-4,0** | NM_030939 | ILMN_1653797 |
| 208 | **C14orf106** | **-4,0** | NM_018353 | ILMN_1666208 |
| 209 | **GSDMDC1** | **-4,0** | NM_024736 | ILMN_1665428 |
| 210 | **ERRFI1** | **-4,0** | NM_018948 | ILMN_1665510 |
| 211 | **TTC15** | **-4,0** | NM_016030 | ILMN_1693317 |
| 212 | **PPFIBP2** | **-4,0** | NM_003621 | ILMN_1675656 |
| 213 | **CFH** | **-4,0** | NM_001014975 | ILMN_1810910 |
| 214 | **LARGE** | **-4,0** | NM_004737 | ILMN_1662038 |
| 215 | **KIAA1600** | **-4,0** | NM_020940 | ILMN_1752927 |
| 216 | **KYNU** | **-4,0** | NM_003937 | ILMN_1746517 |
| 217 | **DDX3X** | **-4,0** | NM_001356 | ILMN_1794392 |
| 218 | **KLHL23** | **-4,0** | NM_144711 | ILMN_1763379 |
| 219 | **TGFBR2** | **-4,0** | NM_001024847 | ILMN_1726245 |
| 220 | **DDEF2** | **-4,0** | NM_003887 | ILMN_1757237 |
| 221 | **FOS** | **-3,9** | NM_005252 | ILMN_1669523 |
| 222 | **CD46** | **-3,9** | NM_153826 | ILMN_1815689 |
| 223 | **FOXC1** | **-3,9** | NM_001453 | ILMN_1743864 |
| 224 | **C1GALT1C1** | **-3,9** | NM_001011551 | ILMN_1751234 |
| 225 | **PANX2** | **-3,9** | NM_052839 | ILMN_1694810 |
| 226 | **ARHGEF16** | **-3,9** | NM_014448 | ILMN_1669928 |
| 227 | **CENPB** | **-3,9** | NM_001810 | ILMN_1664028 |
| 228 | **KIAA0355** | **-3,9** | NM_014686 | ILMN_1659845 |
| 229 | **PERP** | **-3,9** | NM_022121 | ILMN_1726161 |
| 230 | **PNMA2** | **-3,9** | NM_007257 | ILMN_1790778 |
| 231 | **SLC35A5** | **-3,9** | NM_017945 | ILMN_1709817 |
| 232 | **PLEKHA6** | **-3,9** | NM_014935 | ILMN_1797557 |
| 233 | **C6orf69** | **-3,9** | NM_173562 | ILMN_1803476 |
| 234 | **TRAK2** | **-3,9** | NM_015049 | ILMN_1781691 |
| 235 | **TRAM1** | **-3,9** | NM_014294 | ILMN_1737146 |
| 236 | **SFRS2** | **-3,9** | NM_003016 | ILMN_1696407 |
| 237 | **FAM79A** | **-3,9** | NM_182752 | ILMN_1727479 |
| 238 | **SHANK2** | **-3,9** | NM_012309 | ILMN_1814790 |
| 239 | **LACTB2** | **-3,8** | NM_016027 | ILMN_1660635 |
| 240 | **GYG2** | **-3,8** | NM_003918 | ILMN_1815225 |
| 241 | **LANCL1** | **-3,8** | NM_006055 | ILMN_1703697 |
| 242 | **ADSSL1** | **-3,8** | NM_199165 | ILMN_1720235 |
| 243 | **BCKDHA** | **-3,8** | NM_000709 | ILMN_1731442 |
| 244 | **OAT** | **-3,8** | NM_000274 | ILMN_1654441 |
| 245 | **DNAJB6** | **-3,8** | NM_058246 | ILMN_1793770 |
| 246 | **CEBPA** | **-3,8** | NM_004364 | ILMN_1715715 |
| 247 | **TMEM37** | **-3,8** | NM_183240 | ILMN_1670672 |
| 248 | **HYLS1** | **-3,8** | NM_145014 | ILMN_1747078 |
| 249 | **NPY1R** | **-3,8** | NM_000909 | ILMN_1799878 |
| 250 | **IGF2BP3** | **-3,8** | NM_006547 | ILMN_1807423 |
| 251 | **STC2** | **-3,8** | NM_003714 | ILMN_1691884 |
| 252 | **NT5E** | **-3,8** | NM_002526 | ILMN_1697220 |
| 253 | **ZNF395** | **-3,8** | NM_018660 | ILMN_1772876 |
| 254 | **PDE7B** | **-3,8** | NM_018945 | ILMN_1806533 |
| 255 | **CRIM1** | **-3,7** | NM_016441 | ILMN_1809793 |
| 256 | **CYBRD1** | **-3,7** | NM_024843 | ILMN_1712305 |
| 257 | **SDCBP2** | **-3,7** | NM_080489 | ILMN_1705107 |
| 258 | **KIAA0196** | **-3,7** | NM_014846 | ILMN_1728676 |
| 259 | **CD2AP** | **-3,7** | NM_012120 | ILMN_1730433 |
| 260 | **FLJ33814** | **-3,7** | NM_173510 | ILMN_1809889 |
| 261 | **DDIT4L** | **-3,7** | NM_145244 | ILMN_1696537 |
| 262 | **TOP2B** | **-3,7** | NM_001068 | ILMN_1777663 |
| 263 | **MAEA** | **-3,7** | NM_001017405 | ILMN_1747771 |
| 264 | **RHPN2** | **-3,7** | NM_033103 | ILMN_1753143 |
| 265 | **RIPK1** | **-3,7** | NM_003804 | ILMN_1657937 |
| 266 | **RBM12** | **-3,7** | NM_006047 | ILMN_1797698 |
| 267 | **ABR** | **-3,7** | NM_021962 | ILMN_1672878 |
| 268 | **FSTL1** | **-3,7** | NM_007085 | ILMN_1715426 |
| 269 | **PRSS23** | **-3,7** | NM_007173 | ILMN_1797776 |
| 270 | **NDRG1** | **-3,7** | NM_006096 | ILMN_1809931 |
| 271 | **ALCAM** | **-3,7** | NM_001627 | ILMN_1670870 |
| 272 | **HSPA1B** | **-3,7** | NM_005346 | ILMN_1660436 |
| 273 | **KIAA0240** | **-3,7** | NM_015349 | ILMN_1696127 |
| 274 | **CLDN7** | **-3,7** | NM_001307 | ILMN_1723564 |
| 275 | **TIGD5** | **-3,7** | NM_032862 | ILMN_1692816 |
| 276 | **CUL4A** | **-3,7** | NM_003589 | ILMN_1695792 |
| 277 | **SLC39A5** | **-3,7** | NM_173596 | ILMN_1794951 |
| 278 | **SH3PXD2A** | **-3,6** | NM_014631 | ILMN_1743103 |
| 279 | **SFRS3** | **-3,6** | NM_003017 | ILMN_1723212 |
| 280 | **SPRED1** | **-3,6** | NM_152594 | ILMN_1804277 |
| 281 | **OSBPL2** | **-3,6** | NM_144498 | ILMN_1656482 |
| 282 | **CHST13** | **-3,6** | NM_152889 | ILMN_1734707 |
| 283 | **SMARCAD1** | **-3,6** | NM_020159 | ILMN_1741976 |
| 284 | **PAFAH2** | **-3,6** | NM_000437 | ILMN_1682919 |
| 285 | **LXN** | **-3,6** | NM_020169 | ILMN_1723962 |
| 286 | **PCMTD2** | **-3,6** | NM_018257 | ILMN_1767848 |
| 287 | **F7** | **-3,6** | NM_000131 | ILMN_1740559 |
| 288 | **SLC39A10** | **-3,6** | NM_020342 | ILMN_1656129 |
| 289 | **CABC1** | **-3,6** | NM_020247 | ILMN_1731064 |
| 290 | **CREB3L2** | **-3,6** | NM_194071 | ILMN_1751097 |
| 291 | **SIX5** | **-3,6** | NM_175875 | ILMN_1793672 |
| 292 | **EHBP1** | **-3,6** | NM_015252 | ILMN_1803348 |
| 293 | **NCOA3** | **-3,6** | NM_006534 | ILMN_1708805 |
| 294 | **MGC10992** | **-3,6** | NM_033212 | ILMN_1796762 |
| 295 | **BTBD11** | **-3,6** | NM_152322 | ILMN_1705066 |
| 296 | **ZNF689** | **-3,5** | NM_138447 | ILMN_1759008 |
| 297 | **CLCN7** | **-3,5** | NM_001287 | ILMN_1694731 |
| 298 | **CLDN15** | **-3,5** | NM_014343 | ILMN_1682226 |
| 299 | **HNRPA0** | **-3,5** | NM_006805 | ILMN_1753279 |
| 300 | **DPYSL2** | **-3,5** | NM_001386 | ILMN_1672503 |
| 301 | **SYT17** | **-3,5** | NM_016524 | ILMN_1657760 |
| 302 | **RDH10** | **-3,5** | NM_172037 | ILMN_1714335 |
| 303 | **PROS1** | **-3,5** | NM_000313 | ILMN_1671928 |
| 304 | **ETV6** | **-3,5** | NM_001987 | ILMN_1789596 |
| 305 | **TST** | **-3,5** | NM_003312 | ILMN_1691572 |
| 306 | **AHCTF1** | **-3,5** | NM_015446 | ILMN_1809139 |
| 307 | **CTDSPL** | **-3,5** | NM_005808 | ILMN_1683444 |
| 308 | **GNE** | **-3,5** | NM_005476 | ILMN_1664172 |
| 309 | **PLEKHA5** | **-3,5** | NM_019012 | ILMN_1775974 |
| 310 | **POLR3H** | **-3,5** | NM_138338 | ILMN_1786024 |
| 311 | **ROCK2** | **-3,5** | NM_004850 | ILMN_1659099 |
| 312 | **ICK** | **-3,5** | NM_014920 | ILMN_1709882 |
| 313 | **FLJ43339** | **-3,5** | NM_207380 | ILMN_1775330 |
| 314 | **C5orf15** | **-3,5** | NM_020199 | ILMN_1695917 |
| 315 | **ELF2** | **-3,5** | NM_006874 | ILMN_1691559 |
| 316 | **DKFZP686A01247** | **-3,5** | NM_014988 | ILMN_1664138 |
| 317 | **DDX17** | **-3,5** | NM_030881 | ILMN_1764285 |
| 318 | **FBXO32** | **-3,5** | NM_148177 | ILMN_1703955 |
| 319 | **EPS8** | **-3,5** | NM_004447 | ILMN_1733282 |
| 320 | **CAMK2N1** | **-3,5** | NM_018584 | ILMN_1794863 |
| 321 | **SDS** | **-3,5** | NM_006843 | ILMN_1811114 |
| 322 | **SLC38A1** | **-3,5** | NM_030674 | ILMN_1769911 |
| 323 | **ANKFY1** | **-3,5** | NM_016376 | ILMN_1794470 |
| 324 | **INHBB** | **-3,5** | NM_002193 | ILMN_1685714 |
| 325 | **RSPO3** | **-3,5** | NM_032784 | ILMN_1681983 |
| 326 | **MBP** | **-3,5** | NM_001025100 | ILMN_1672660 |
| 327 | **ALDH3B1** | **-3,4** | NM_001030010 | ILMN_1728662 |
| 328 | **PRKCA** | **-3,4** | NM_002737 | ILMN_1771800 |
| 329 | **MAGED1** | **-3,4** | NM_001005333 | ILMN_1775522 |
| 330 | **NT5C2** | **-3,4** | NM_012229 | ILMN_1682165 |
| 331 | **BCDIN3** | **-3,4** | NM_019606 | ILMN_1739616 |
| 332 | **TXNRD1** | **-3,4** | NM_003330 | ILMN_1717056 |
| 333 | **RAB35** | **-3,4** | NM_006861 | ILMN_1812571 |
| 334 | **FLJ20160** | **-3,4** | NM_017694 | ILMN_1710209 |
| 335 | **EXOC6** | **-3,4** | NM_001013848 | ILMN_1651628 |
| 336 | **GSPT2** | **-3,4** | NM_018094 | ILMN_1730101 |
| 337 | **HDAC1** | **-3,4** | NM_004964 | ILMN_1727458 |
| 338 | **RND1** | **-3,4** | NM_014470 | ILMN_1651838 |
| 339 | **TCFL5** | **-3,4** | NM_006602 | ILMN_1814247 |
| 340 | **SRC** | **-3,4** | NM_005417 | ILMN_1729987 |
| 341 | **WWP1** | **-3,4** | NM_007013 | ILMN_1692092 |
| 342 | **CNTNAP2** | **-3,4** | NM_014141 | ILMN_1690223 |
| 343 | **APOA1** | **-3,4** | NM_000039 | ILMN_1690884 |
| 344 | **NUSAP1** | **-3,4** | NM_016359 | ILMN_1726720 |
| 345 | **EFNA1** | **-3,4** | NM_004428 | ILMN_1775903 |
| 346 | **TCF2** | **-3,4** | NM_000458 | ILMN_1778337 |
| 347 | **ATAD4** | **-3,4** | NM_024320 | ILMN_1748970 |
| 348 | **RBM23** | **-3,4** | NM_018107 | ILMN_1780756 |
| 349 | **B3GALT6** | **-3,4** | NM_080605 | ILMN_1739749 |
| 350 | **FADD** | **-3,4** | NM_003824 | ILMN_1758658 |
| 351 | **EIF4EBP2** | **-3,4** | NM_004096 | ILMN_1762728 |
| 352 | **FAM83A** | **-3,4** | NM_032899 | ILMN_1796479 |
| 353 | **TFB2M** | **-3,4** | NM_022366 | ILMN_1779998 |
| 354 | **RB1CC1** | **-3,4** | NM_014781 | ILMN_1736796 |
| 355 | **F2** | **-3,4** | NM_000506 | ILMN_1671753 |
| 356 | **BTBD7** | **-3,4** | NM_001002860 | ILMN_1687743 |
| 357 | **C19orf12** | **-3,4** | NM_031448 | ILMN_1664920 |
| 358 | **SUV420H2** | **-3,3** | NM_032701 | ILMN_1812208 |
| 359 | **C1RL** | **-3,3** | NM_016546 | ILMN_1733288 |
| 360 | **ITGAV** | **-3,3** | NM_002210 | ILMN_1706592 |
| 361 | **PIGM** | **-3,3** | NM_145167 | ILMN_1799860 |
| 362 | **B4GALT4** | **-3,3** | NM_003778 | ILMN_1661500 |
| 363 | **C1orf59** | **-3,3** | NM_144584 | ILMN_1682428 |
| 364 | **ATP6V0A1** | **-3,3** | NM_005177 | ILMN_1752579 |
| 365 | **RARB** | **-3,3** | NM_000965 | ILMN_1671151 |
| 366 | **PILRB** | **-3,3** | NM_175047 | ILMN_1768754 |
| 367 | **MYO1A** | **-3,3** | NM_005379 | ILMN_1684031 |
| 368 | **AKAP12** | **-3,3** | NM_005100 | ILMN_1684836 |
| 369 | **LMBRD1** | **-3,3** | NM_018368 | ILMN_1652128 |
| 370 | **IFNAR1** | **-3,3** | NM_000629 | ILMN_1752923 |
| 371 | **C14orf147** | **-3,3** | NM_138288 | ILMN_1699676 |
| 372 | **SLC23A1** | **-3,3** | NM_152685 | ILMN_1760950 |
| 373 | **PHF20L1** | **-3,3** | NM_016018 | ILMN_1732985 |
| 374 | **PLEKHA9** | **-3,3** | NM_015899 | ILMN_1706610 |
| 375 | **RBM4B** | **-3,3** | NM_031492 | ILMN_1743104 |
| 376 | **SOX9** | **-3,3** | NM_000346 | ILMN_1705803 |
| 377 | **PDHB** | **-3,3** | NM_000925 | ILMN_1739274 |
| 378 | **TMEM30A** | **-3,3** | NM_018247 | ILMN_1802337 |
| 379 | **CCDC71** | **-3,3** | NM_022903 | ILMN_1768433 |
| 380 | **ITGB5** | **-3,3** | NM_002213 | ILMN_1796755 |
| 381 | **FAM13A1** | **-3,3** | NM_014883 | ILMN_1752510 |
| 382 | **LOC144233** | **-3,3** | NM_181708 | ILMN_1796113 |
| 383 | **TESC** | **-3,3** | NM_017899 | ILMN_1750181 |
| 384 | **TOB1** | **-3,3** | NM_005749 | ILMN_1672004 |
| 385 | **GNA11** | **-3,2** | NM_002067 | ILMN_1739781 |
| 386 | **NCOA5** | **-3,2** | NM_020967 | ILMN_1770035 |
| 387 | **CPD** | **-3,2** | NM_001304 | ILMN_1703074 |
| 388 | **UBL3** | **-3,2** | NM_007106 | ILMN_1810729 |
| 389 | **C21orf55** | **-3,2** | NM_017833 | ILMN_1814204 |
| 390 | **GALM** | **-3,2** | NM_138801 | ILMN_1671482 |
| 391 | **C5** | **-3,2** | NM_001735 | ILMN_1746819 |
| 392 | **GOLT1B** | **-3,2** | NM_016072 | ILMN_1767837 |
| 393 | **TIMP2** | **-3,2** | NM_003255 | ILMN_1670054 |
| 394 | **RNF170** | **-3,2** | NM_030954 | ILMN_1709265 |
| 395 | **EPHA3** | **-3,2** | NM_005233 | ILMN_1775931 |
| 396 | **STK38** | **-3,2** | NM_007271 | ILMN_1799153 |
| 397 | **CAPN5** | **-3,2** | NM_004055 | ILMN_1737089 |
| 398 | **TMEM59** | **-3,2** | NM_004872 | ILMN_1792508 |
| 399 | **ARID2** | **-3,2** | NM_152641 | ILMN_1795247 |
| 400 | **MVP** | **-3,2** | NM_005115 | ILMN_1803277 |
| 401 | **PALM** | **-3,2** | NM_002579 | ILMN_1812031 |
| 402 | **NIF3L1** | **-3,2** | NM_021824 | ILMN_1777066 |
| 403 | **CCPG1** | **-3,2** | NM_004748 | ILMN_1794190 |
| 404 | **WDR36** | **-3,2** | NM_139281 | ILMN_1727184 |
| 405 | **ACSL3** | **-3,2** | NM_004457 | ILMN_1666096 |
| 406 | **SHRM** | **-3,2** | NM_020859 | ILMN_1791392 |
| 407 | **GMFB** | **-3,2** | NM_004124 | ILMN_1752915 |
| 408 | **PUM1** | **-3,2** | NM_014676 | ILMN_1783424 |
| 409 | **FAM20C** | **-3,2** | NM_020223 | ILMN_1712684 |
| 410 | **C1orf71** | **-3,2** | NM_152609 | ILMN_1670263 |
| 411 | **ACOT11** | **-3,2** | NM_147161 | ILMN_1739594 |
| 412 | **EDG2** | **-3,2** | NM_001401 | ILMN_1701441 |
| 413 | **SLC30A7** | **-3,2** | NM_133496 | ILMN_1789999 |
| 414 | **LOC400657** | **-3,1** | NM_001008234 | ILMN_1725528 |
| 415 | **SLC29A4** | **-3,1** | NM_153247 | ILMN_1801377 |
| 416 | **SLC16A5** | **-3,1** | NM_004695 | ILMN_1755649 |
| 417 | **LCMT2** | **-3,1** | NM_014793 | ILMN_1667577 |
| 418 | **ZNF281** | **-3,1** | NM_012482 | ILMN_1802758 |
| 419 | **TMED10** | **-3,1** | NM_006827 | ILMN_1736585 |
| 420 | **INSL4** | **-3,1** | NM_002195 | ILMN_1720540 |
| 421 | **LEMD3** | **-3,1** | NM_014319 | ILMN_1727361 |
| 422 | **NETO2** | **-3,1** | NM_018092 | ILMN_1760849 |
| 423 | **RPS6KB1** | **-3,1** | NM_003161 | ILMN_1704557 |
| 424 | **MRFAP1L1** | **-3,1** | NM_152301 | ILMN_1689774 |
| 425 | **PLCL2** | **-3,1** | NM_015184 | ILMN_1737025 |
| 426 | **PELI2** | **-3,1** | NM_021255 | ILMN_1780132 |
| 427 | **MAT2A** | **-3,1** | NM_005911 | ILMN_1737298 |
| 428 | **KIAA0350** | **-3,1** | NM_015226 | ILMN_1744851 |
| 429 | **ITM2B** | **-3,1** | NM_021999 | ILMN_1713733 |
| 430 | **AASDHPPT** | **-3,1** | NM_015423 | ILMN_1698189 |
| 431 | **STAT4** | **-3,1** | NM_003151 | ILMN_1785202 |
| 432 | **PCYOX1** | **-3,1** | NM_016297 | ILMN_1679725 |
| 433 | **ARHGAP21** | **-3,1** | NM_020824 | ILMN_1811592 |
| 434 | **MARCKS** | **-3,1** | NM_002356 | ILMN_1807042 |
| 435 | **LOC203547** | **-3,1** | NM_001017980 | ILMN_1682694 |
| 436 | **TOR1A** | **-3,1** | NM_000113 | ILMN_1805812 |
| 437 | **ACVR1** | **-3,1** | NM_001105 | ILMN_1760490 |
| 438 | **TMEM47** | **-3,1** | NM_031442 | ILMN_1700274 |
| 439 | **DHFRL1** | **-3,1** | NM_176815 | ILMN_1762426 |
| 440 | **PON2** | **-3,1** | NM_000305 | ILMN_1714158 |
| 441 | **SPG7** | **-3,1** | NM_003119 | ILMN_1754529 |
| 442 | **CORO2A** | **-3,1** | NM_052820 | ILMN_1813746 |
| 443 | **AP1G1** | **-3,0** | NM_001030007 | ILMN_1652569 |
| 444 | **TUBB2C** | **-3,0** | NM_006088 | ILMN_1780769 |
| 445 | **AEBP2** | **-3,0** | NM_153207 | ILMN_1803376 |
| 446 | **VPS36** | **-3,0** | NM_016075 | ILMN_1802519 |
| 447 | **SRP46** | **-3,0** | NM_032102 | ILMN_1772702 |
| 448 | **SLPI** | **-3,0** | NM_003064 | ILMN_1669650 |
| 449 | **MGC35212** | **-3,0** | NM_152764 | ILMN_1754241 |
| 450 | **ANKRD17** | **-3,0** | NM_198889 | ILMN_1712019 |
| 451 | **PCNA** | **-3,0** | NM_182649 | ILMN_1694177 |
| 452 | **SYNJ2BP** | **-3,0** | NM_018373 | ILMN_1697793 |
| 453 | **CDKN2C** | **-3,0** | NM_078626 | ILMN_1656415 |
| 454 | **C11orf54** | **-3,0** | NM_014039 | ILMN_1664738 |
| 455 | **HOXC6** | **-3,0** | NM_153693 | ILMN_1794492 |
| 456 | **LSM14A** | **-3,0** | NM_015578 | ILMN_1654628 |
| 457 | **RCN2** | **-3,0** | NM_002902 | ILMN_1662129 |
| 458 | **ACF** | **-3,0** | NM_138933 | ILMN_1806310 |
| 459 | **CSPG2** | **-3,0** | NM_004385 | ILMN_1687301 |
| 460 | **ARID5B** | **-3,0** | NM_032199 | ILMN_1721626 |
| 461 | **CHMP2B** | **-3,0** | NM_014043 | ILMN_1683698 |
| 462 | **SLC5A11** | **-3,0** | NM_052944 | ILMN_1715003 |
| 463 | **LBR** | **-3,0** | NM_002296 | ILMN_1724240 |
| 464 | **ARSD** | **-3,0** | NM_001669 | ILMN_1684873 |
| 465 | **ACSM3** | **-3,0** | NM_202000 | ILMN_1685952 |
| 466 | **LAMA5** | **-3,0** | NM_005560 | ILMN_1773567 |
| 467 | **PTER** | **-3,0** | NM_030664 | ILMN_1795336 |
| 468 | **GOLGA3** | **-3,0** | NM_005895 | ILMN_1733511 |
| 469 | **FLOT2** | **-3,0** | NM_004475 | ILMN_1726222 |
| 470 | **VIL2** | **-3,0** | NM_003379 | ILMN_1795937 |
| 471 | **C9orf5** | **-3,0** | NM_032012 | ILMN_1788384 |
| 472 | **UBE2Q1** | **-3,0** | NM_017582 | ILMN_1811751 |
| 473 | **PTPRK** | **-3,0** | NM_002844 | ILMN_1810962 |
| 474 | **DNAJC12** | **-3,0** | NM_021800 | ILMN_1803073 |
| 475 | **DDX23** | **-3,0** | NM_004818 | ILMN_1784218 |
| 476 | **KIAA0859** | **-3,0** | NM_015935 | ILMN_1790575 |
| 477 | **LOC91461** | **-3,0** | NM_138370 | ILMN_1734445 |
| 478 | **SMAD4** | **-3,0** | NM_005359 | ILMN_1741477 |
| 479 | **ZNF217** | **-3,0** | NM_006526 | ILMN_1789841 |
| 480 | **VRK3** | **-3,0** | NM_001025778 | ILMN_1771697 |
| 481 | **MIS12** | **-3,0** | NM_024039 | ILMN_1718069 |
| 482 | **TMEM66** | **-3,0** | NM_016127 | ILMN_1780141 |
| 483 | **MYLIP** | **-3,0** | NM_013262 | ILMN_1656111 |
| 484 | **PFKFB4** | **-3,0** | NM_004567 | ILMN_1653292 |
| 485 | **CHD9** | **-3,0** | NM_025134 | ILMN_1762972 |
| 486 | **DHRS8** | **-3,0** | NM_016245 | ILMN_1722076 |
| 487 | **ISGF3G** | **-3,0** | NM_006084 | ILMN_1745471 |
| 488 | **GADD45B** | **-3,0** | NM_015675 | ILMN_1718977 |
| 489 | **MGC24665** | **-3,0** | NM_152308 | ILMN_1790537 |
| 490 | **HDHD1A** | **-3,0** | NM_012080 | ILMN_1710136 |
| 491 | **LASP1** | **-3,0** | NM_006148 | ILMN_1665909 |
| 492 | **ZCCHC6** | **-3,0** | NM_024617 | ILMN_1739548 |
| 493 | **C14orf58** | **-3,0** | NM_017791 | ILMN_1696043 |
| 494 | **RAB40B** | **-2,9** | NM_006822 | ILMN_1685820 |
| 495 | **PDCD6IP** | **-2,9** | NM_013374 | ILMN_1693259 |
| 496 | **CART1** | **-2,9** | NM_006982 | ILMN_1724540 |
| 497 | **CDK6** | **-2,9** | NM_001259 | ILMN_1802615 |
| 498 | **TEGT** | **-2,9** | NM_003217 | ILMN_1693311 |
| 499 | **NR1H3** | **-2,9** | NM_005693 | ILMN_1814022 |
| 500 | **ASGR1** | **-2,9** | NM_001671 | ILMN_1769013 |
| 501 | **C19orf21** | **-2,9** | NM_173481 | ILMN_1683905 |
| 502 | **ATP6V1C1** | **-2,9** | NM_001695 | ILMN_1659801 |
| 503 | **TJP3** | **-2,9** | NM_014428 | ILMN_1659610 |
| 504 | **GOLPH3L** | **-2,9** | NM_018178 | ILMN_1655570 |
| 505 | **SASH1** | **-2,9** | NM_015278 | ILMN_1712673 |
| 506 | **UBXD8** | **-2,9** | NM_014613 | ILMN_1670472 |
| 507 | **LACTB** | **-2,9** | NM_032857 | ILMN_1703335 |
| 508 | **C9orf42** | **-2,9** | NM_138333 | ILMN_1723353 |
| 509 | **YPEL5** | **-2,9** | NM_016061 | ILMN_1711069 |
| 510 | **WDR68** | **-2,9** | NM_005828 | ILMN_1682264 |
| 511 | **TBC1D8** | **-2,9** | NM_007063 | ILMN_1735495 |
| 512 | **FOXD2** | **-2,9** | NM_004474 | ILMN_1789400 |
| 513 | **TAF4** | **-2,9** | NM_003185 | ILMN_1737535 |
| 514 | **PRKCBP1** | **-2,9** | NM_012408 | ILMN_1652407 |
| 515 | **ARRB1** | **-2,9** | NM_004041 | ILMN_1735218 |
| 516 | **DDX46** | **-2,9** | NM_014829 | ILMN_1727001 |
| 517 | **CALD1** | **-2,9** | NM_004342 | ILMN_1730487 |
| 518 | **KIAA0143** | **-2,9** | NM_015137 | ILMN_1664776 |
| 519 | **EPB41L1** | **-2,9** | NM_012156 | ILMN_1719475 |
| 520 | **RDX** | **-2,9** | NM_002906 | ILMN_1708611 |
| 521 | **HECTD1** | **-2,9** | NM_015382 | ILMN_1670097 |
| 522 | **WDR35** | **-2,9** | NM_001006657 | ILMN_1789879 |
| 523 | **GALGT** | **-2,9** | NM_001478 | ILMN_1805725 |
| 524 | **ADD1** | **-2,9** | NM_001119 | ILMN_1759252 |
| 525 | **SORCS2** | **-2,9** | NM_020777 | ILMN_1695935 |
| 526 | **GABARAPL1** | **-2,9** | NM_031412 | ILMN_1667846 |
| 527 | **NUP35** | **-2,9** | NM_138285 | ILMN_1815723 |
| 528 | **SMEK2** | **-2,9** | NM_020463 | ILMN_1661650 |
| 529 | **OPN3** | **-2,9** | NM_014322 | ILMN_1670844 |
| 530 | **CUL1** | **-2,9** | NM_003592 | ILMN_1749629 |
| 531 | **TBL1X** | **-2,9** | NM_005647 | ILMN_1744795 |
| 532 | **MUC13** | **-2,9** | NM_033049 | ILMN_1651568 |
| 533 | **ELF1** | **-2,9** | NM_172373 | ILMN_1664010 |
| 534 | **SMC4L1** | **-2,9** | NM_001002799 | ILMN_1728556 |
| 535 | **MGEA5** | **-2,9** | NM_012215 | ILMN_1686750 |
| 536 | **DEGS1** | **-2,9** | NM_003676 | ILMN_1780058 |
| 537 | **HDAC8** | **-2,9** | NM_018486 | ILMN_1651544 |
| 538 | **EFHD2** | **-2,8** | NM_024329 | ILMN_1761463 |
| 539 | **IRF2BP2** | **-2,8** | NM_182972 | ILMN_1671005 |
| 540 | **MAP3K8** | **-2,8** | NM_005204 | ILMN_1741159 |
| 541 | **ZMYND11** | **-2,8** | NM_006624 | ILMN_1658149 |
| 542 | **CYB5-M** | **-2,8** | NM_030579 | ILMN_1684321 |
| 543 | **SUZ12** | **-2,8** | NM_015355 | ILMN_1797813 |
| 544 | **SPRED2** | **-2,8** | NM_181784 | ILMN_1791232 |
| 545 | **STK6** | **-2,8** | NM_198434 | ILMN_1680955 |
| 546 | **ANKRD5** | **-2,8** | NM_022096 | ILMN_1756437 |
| 547 | **USP13** | **-2,8** | NM_003940 | ILMN_1708059 |
| 548 | **ISG20** | **-2,8** | NM_002201 | ILMN_1659913 |
| 549 | **EML4** | **-2,8** | NM_019063 | ILMN_1718297 |
| 550 | **MLPH** | **-2,8** | NM_024101 | ILMN_1795342 |
| 551 | **UNC50** | **-2,8** | NM_014044 | ILMN_1766981 |
| 552 | **SLC35E3** | **-2,8** | NM_018656 | ILMN_1749521 |
| 553 | **FLJ22471** | **-2,8** | NM_025140 | ILMN_1731107 |
| 554 | **ATP9B** | **-2,8** | NM_198531 | ILMN_1658684 |
| 555 | **ZNF434** | **-2,8** | NM_017810 | ILMN_1684591 |
| 556 | **BID** | **-2,8** | NM_001196 | ILMN_1763386 |
| 557 | **NUCB1** | **-2,8** | NM_006184 | ILMN_1722634 |
| 558 | **LHPP** | **-2,8** | NM_022126 | ILMN_1752199 |
| 559 | **LIN28B** | **-2,8** | NM_001004317 | ILMN_1748697 |
| 560 | **MEF2D** | **-2,8** | NM_005920 | ILMN_1763228 |
| 561 | **ZFYVE26** | **-2,8** | NM_015346 | ILMN_1798061 |
| 562 | **RAB11FIP3** | **-2,8** | NM_014700 | ILMN_1708328 |
| 563 | **MYO1E** | **-2,8** | NM_004998 | ILMN_1773342 |
| 564 | **HMGCS1** | **-2,8** | NM_002130 | ILMN_1797728 |
| 565 | **WDR40A** | **-2,8** | NM_015397 | ILMN_1786328 |
| 566 | **PGD** | **-2,8** | NM_002631 | ILMN_1794165 |
| 567 | **C9orf64** | **-2,8** | NM_032307 | ILMN_1777318 |
| 568 | **SLC7A9** | **-2,8** | NM_014270 | ILMN_1735445 |
| 569 | **BIRC6** | **-2,8** | NM_016252 | ILMN_1801605 |
| 570 | **LOC400451** | **-2,8** | NM_207446 | ILMN_1652797 |
| 571 | **FLJ11000** | **-2,8** | NM_018295 | ILMN_1736863 |
| 572 | **HNRPM** | **-2,8** | NM_031203 | ILMN_1745385 |
| 573 | **EPS15** | **-2,8** | NM_001981 | ILMN_1665357 |
| 574 | **C8orf1** | **-2,8** | NM_004337 | ILMN_1735472 |
| 575 | **PTPN12** | **-2,8** | NM_002835 | ILMN_1695509 |
| 576 | **PCDH9** | **-2,8** | NM_020403 | ILMN_1752294 |
| 577 | **NPY5R** | **-2,8** | NM_006174 | ILMN_1718198 |
| 578 | **LOC116143** | **-2,8** | NM_138458 | ILMN_1662065 |
| 579 | **CCM2** | **-2,8** | NM_031443 | ILMN_1784352 |
| 580 | **TMOD3** | **-2,8** | NM_014547 | ILMN_1809484 |
| 581 | **EPDR1** | **-2,8** | NM_017549 | ILMN_1675797 |
| 582 | **KIAA1729** | **-2,8** | NM_053042 | ILMN_1682449 |
| 583 | **KIAA0895** | **-2,8** | NM_015314 | ILMN_1690484 |
| 584 | **RTN4IP1** | **-2,8** | NM_032730 | ILMN_1758827 |
| 585 | **BAZ2B** | **-2,8** | NM_013450 | ILMN_1720850 |
| 586 | **SPPL2A** | **-2,8** | NM_032802 | ILMN_1734229 |
| 587 | **SEC23IP** | **-2,8** | NM_007190 | ILMN_1690690 |
| 588 | **KIAA0182** | **-2,8** | NM_014615 | ILMN_1807767 |
| 589 | **BCAR3** | **-2,8** | NM_003567 | ILMN_1763638 |
| 590 | **LRRC8D** | **-2,8** | NM_018103 | ILMN_1782878 |
| 591 | **ANXA8** | **-2,8** | NM_001630 | ILMN_1778087 |
| 592 | **RFWD2** | **-2,7** | NM_022457 | ILMN_1661002 |
| 593 | **C8orf13** | **-2,7** | NM_053279 | ILMN_1687213 |
| 594 | **ARL6IP6** | **-2,7** | NM_152522 | ILMN_1797964 |
| 595 | **RARA** | **-2,7** | NM_001024809 | ILMN_1659206 |
| 596 | **RFP** | **-2,7** | NM_030950 | ILMN_1730005 |
| 597 | **C1orf93** | **-2,7** | NM_152371 | ILMN_1732347 |
| 598 | **SEC14L4** | **-2,7** | NM_174977 | ILMN_1742947 |
| 599 | **CD44** | **-2,7** | NM_001001390 | ILMN_1803429 |
| 600 | **CDKN1B** | **-2,7** | NM_004064 | ILMN_1722811 |
| 601 | **UBE3C** | **-2,7** | NM_014671 | ILMN_1704342 |
| 602 | **KIF11** | **-2,7** | NM_004523 | ILMN_1794539 |
| 603 | **ANKRD25** | **-2,7** | NM_015493 | ILMN_1733226 |
| 604 | **GAS2** | **-2,7** | NM_177553 | ILMN_1804569 |
| 605 | **PKD2** | **-2,7** | NM_000297 | ILMN_1660236 |
| 606 | **FLJ38663** | **-2,7** | NM_152269 | ILMN_1667510 |
| 607 | **VPS4B** | **-2,7** | NM_004869 | ILMN_1792587 |
| 608 | **PNMA3** | **-2,7** | NM_013364 | ILMN_1783805 |
| 609 | **HPS3** | **-2,7** | NM_032383 | ILMN_1762224 |
| 610 | **ST3GAL5** | **-2,7** | NM_003896 | ILMN_1713496 |
| 611 | **SCD** | **-2,7** | NM_005063 | ILMN_1689329 |
| 612 | **UTP14C** | **-2,7** | NM_021645 | ILMN_1686645 |
| 613 | **KIAA0446** | **-2,7** | NM_014655 | ILMN_1810514 |
| 614 | **ELP3** | **-2,7** | NM_018091 | ILMN_1744068 |
| 615 | **TACC1** | **-2,7** | NM_006283 | ILMN_1770084 |
| 616 | **ECH1** | **-2,7** | NM_001398 | ILMN_1653115 |
| 617 | **ST3GAL2** | **-2,7** | NM_006927 | ILMN_1714165 |
| 618 | **PRMT2** | **-2,7** | NM_206962 | ILMN_1675038 |
| 619 | **LRIG1** | **-2,7** | NM_015541 | ILMN_1707342 |
| 620 | **MLKL** | **-2,7** | NM_152649 | ILMN_1667825 |
| 621 | **PHACTR4** | **-2,7** | NM_023923 | ILMN_1736548 |
| 622 | **SH3BGRL** | **-2,7** | NM_003022 | ILMN_1702835 |
| 623 | **DAZAP2** | **-2,7** | NM_014764 | ILMN_1718988 |
| 624 | **NULP1** | **-2,7** | NM_014972 | ILMN_1814971 |
| 625 | **NUDT21** | **-2,7** | NM_007006 | ILMN_1798886 |
| 626 | **APAF1** | **-2,7** | NM_181869 | ILMN_1659463 |
| 627 | **RARRES1** | **-2,7** | NM_206963 | ILMN_1800091 |
| 628 | **C1orf48** | **-2,7** | NM_015471 | ILMN_1739210 |
| 629 | **IGSF11** | **-2,7** | NM_001015887 | ILMN_1753502 |
| 630 | **LZTFL1** | **-2,7** | NM_020347 | ILMN_1815705 |
| 631 | **FBXO33** | **-2,7** | NM_203301 | ILMN_1664826 |
| 632 | **LEPROTL1** | **-2,7** | NM_015344 | ILMN_1752591 |
| 633 | **KBTBD11** | **-2,7** | NM_014867 | ILMN_1784630 |
| 634 | **SCAP2** | **-2,7** | NM_003930 | ILMN_1657129 |
| 635 | **C4orf13** | **-2,7** | NM_001029998 | ILMN_1732489 |
| 636 | **LARP5** | **-2,7** | NM_015155 | ILMN_1766222 |
| 637 | **LHX2** | **-2,7** | NM_004789 | ILMN_1807016 |
| 638 | **WDR72** | **-2,7** | NM_182758 | ILMN_1763196 |
| 639 | **RAN** | **-2,7** | NM_006325 | ILMN_1757384 |
| 640 | **SNX4** | **-2,7** | NM_003794 | ILMN_1738736 |
| 641 | **PDZK1** | **-2,7** | NM_002614 | ILMN_1694535 |
| 642 | **KLF13** | **-2,7** | NM_015995 | ILMN_1679929 |
| 643 | **OLFML2A** | **-2,7** | NM_182487 | ILMN_1761425 |
| 644 | **SERPINA1** | **-2,7** | NM_001002236 | ILMN_1745961 |
| 645 | **WWC3** | **-2,7** | NM_015691 | ILMN_1661361 |
| 646 | **MKRN1** | **-2,7** | NM_013446 | ILMN_1671583 |
| 647 | **SENP6** | **-2,7** | NM_015571 | ILMN_1761731 |
| 648 | **MC1R** | **-2,7** | NM_002386 | ILMN_1653319 |
| 649 | **CYLN2** | **-2,6** | NM_032421 | ILMN_1798846 |
| 650 | **JOSD1** | **-2,6** | NM_014876 | ILMN_1798536 |
| 651 | **FLJ23191** | **-2,6** | NM_024574 | ILMN_1689176 |
| 652 | **ACACB** | **-2,6** | NM_001093 | ILMN_1763852 |
| 653 | **FLJ46072** | **-2,6** | NM_198488 | ILMN_1751328 |
| 654 | **PCMTD1** | **-2,6** | NM_052937 | ILMN_1737426 |
| 655 | **MOBKL2C** | **-2,6** | NM_201403 | ILMN_1798288 |
| 656 | **PQLC3** | **-2,6** | NM_152391 | ILMN_1814213 |
| 657 | **KRT8** | **-2,6** | NM_002273 | ILMN_1753584 |
| 658 | **PLEKHA1** | **-2,6** | NM_001001974 | ILMN_1662839 |
| 659 | **SDSL** | **-2,6** | NM_138432 | ILMN_1750674 |
| 660 | **MARVELD3** | **-2,6** | NM_001017967 | ILMN_1710644 |
| 661 | **LPIN1** | **-2,6** | NM_145693 | ILMN_1671554 |
| 662 | **ADRA1B** | **-2,6** | NM_000679 | ILMN_1679754 |
| 663 | **SLMAP** | **-2,6** | NM_007159 | ILMN_1783120 |
| 664 | **CAB39** | **-2,6** | NM_016289 | ILMN_1765858 |
| 665 | **UNG** | **-2,6** | NM_080911 | ILMN_1683120 |
| 666 | **PRKAA1** | **-2,6** | NM_206907 | ILMN_1783889 |
| 667 | **ITCH** | **-2,6** | NM_031483 | ILMN_1752283 |
| 668 | **MMD** | **-2,6** | NM_012329 | ILMN_1733937 |
| 669 | **CDH17** | **-2,6** | NM_004063 | ILMN_1814015 |
| 670 | **STXBP3** | **-2,6** | NM_007269 | ILMN_1764168 |
| 671 | **FBXO5** | **-2,6** | NM_012177 | ILMN_1710676 |
| 672 | **ATRN** | **-2,6** | NM_139321 | ILMN_1772124 |
| 673 | **CTH** | **-2,6** | NM_153742 | ILMN_1784112 |
| 674 | **NIPA2** | **-2,6** | NM_030922 | ILMN_1720344 |
| 675 | **ZDHHC7** | **-2,6** | NM_017740 | ILMN_1730568 |
| 676 | **ARL6IP** | **-2,6** | NM_015161 | ILMN_1708416 |
| 677 | **E2F6** | **-2,6** | NM_001952 | ILMN_1656196 |
| 678 | **CDS2** | **-2,6** | NM_003818 | ILMN_1725917 |
| 679 | **PAPOLA** | **-2,6** | NM_032632 | ILMN_1798354 |
| 680 | **FLJ40629** | **-2,6** | NM_152515 | ILMN_1751776 |
| 681 | **TMEM44** | **-2,6** | NM_138399 | ILMN_1688848 |
| 682 | **ZNF17** | **-2,6** | NM_006959 | ILMN_1751692 |
| 683 | **SLC9A3R1** | **-2,6** | NM_004252 | ILMN_1680925 |
| 684 | **CENPF** | **-2,6** | NM_016343 | ILMN_1664516 |
| 685 | **KIF13B** | **-2,6** | NM_015254 | ILMN_1686562 |
| 686 | **CENPA** | **-2,6** | NM_001809 | ILMN_1801257 |
| 687 | **ABCC4** | **-2,6** | NM_005845 | ILMN_1788457 |
| 688 | **FOXO3A** | **-2,6** | NM_001455 | ILMN_1681703 |
| 689 | **H3F3B** | **-2,6** | NM_005324 | ILMN_1695706 |
| 690 | **C1orf60** | **-2,6** | NM_023015 | ILMN_1756086 |
| 691 | **YY1** | **-2,6** | NM_003403 | ILMN_1770892 |
| 692 | **TIGD2** | **-2,6** | NM_145715 | ILMN_1690066 |
| 693 | **BAMBI** | **-2,6** | NM_012342 | ILMN_1691410 |
| 694 | **DEK** | **-2,6** | NM_003472 | ILMN_1663061 |
| 695 | **MNT** | **-2,6** | NM_020310 | ILMN_1792910 |
| 696 | **FLJ11171** | **-2,6** | NM_018348 | ILMN_1814573 |
| 697 | **EDARADD** | **-2,6** | NM_145861 | ILMN_1761820 |
| 698 | **LPGAT1** | **-2,6** | NM_014873 | ILMN_1687998 |
| 699 | **GNG12** | **-2,6** | NM_018841 | ILMN_1673380 |
| 700 | **DIP13B** | **-2,6** | NM_018171 | ILMN_1765076 |
| 701 | **SCARA5** | **-2,6** | NM_173833 | ILMN_1751062 |
| 702 | **AMD1** | **-2,6** | NM_001033059 | ILMN_1788462 |
| 703 | **CRBN** | **-2,6** | NM_016302 | ILMN_1668582 |
| 704 | **TMED7** | **-2,6** | NM_181836 | ILMN_1672405 |
| 705 | **MEIS2** | **-2,6** | NM_020149 | ILMN_1695945 |
| 706 | **C10orf137** | **-2,6** | NM_015608 | ILMN_1791656 |
| 707 | **DEPDC6** | **-2,6** | NM_022783 | ILMN_1756685 |
| 708 | **IFT74** | **-2,6** | NM_025103 | ILMN_1777449 |
| 709 | **MSRB3** | **-2,6** | NM_198080 | ILMN_1676088 |
| 710 | **AGPAT2** | **-2,6** | NM_006412 | ILMN_1681081 |
| 711 | **GCNT3** | **-2,6** | NM_004751 | ILMN_1712082 |
| 712 | **CGGBP1** | **-2,6** | NM_003663 | ILMN_1752631 |
| 713 | **DPP8** | **-2,6** | NM_130434 | ILMN_1759801 |
| 714 | **TBC1D14** | **-2,5** | NM_020773 | ILMN_1779886 |
| 715 | **AKR1C3** | **-2,5** | NM_003739 | ILMN_1713124 |
| 716 | **TUBGCP5** | **-2,5** | NM_052903 | ILMN_1803045 |
| 717 | **FBXO8** | **-2,5** | NM_012180 | ILMN_1672843 |
| 718 | **TXNDC** | **-2,5** | NM_030755 | ILMN_1691181 |
| 719 | **MYO10** | **-2,5** | NM_012334 | ILMN_1703576 |
| 720 | **PARN** | **-2,5** | NM_002582 | ILMN_1724811 |
| 721 | **TLOC1** | **-2,5** | NM_003262 | ILMN_1762003 |
| 722 | **KIAA0319** | **-2,5** | NM_014809 | ILMN_1657497 |
| 723 | **NDFIP2** | **-2,5** | NM_019080 | ILMN_1677396 |
| 724 | **RERG** | **-2,5** | NM_032918 | ILMN_1746359 |
| 725 | **SDPR** | **-2,5** | NM_004657 | ILMN_1715991 |
| 726 | **RAB31** | **-2,5** | NM_006868 | ILMN_1660691 |
| 727 | **NQO1** | **-2,5** | NM_000903 | ILMN_1720282 |
| 728 | **ARID3A** | **-2,5** | NM_005224 | ILMN_1670130 |
| 729 | **MBTPS1** | **-2,5** | NM_201268 | ILMN_1651719 |
| 730 | **MBD4** | **-2,5** | NM_003925 | ILMN_1708764 |
| 731 | **C9orf66** | **-2,5** | NM_152569 | ILMN_1717248 |
| 732 | **PSPH** | **-2,5** | NM_004577 | ILMN_1776105 |
| 733 | **C3orf29** | **-2,5** | NM_022485 | ILMN_1659240 |
| 734 | **BRD3** | **-2,5** | NM_007371 | ILMN_1785635 |
| 735 | **CBX2** | **-2,5** | NM_005189 | ILMN_1770678 |
| 736 | **DACT2** | **-2,5** | NM_214462 | ILMN_1691790 |
| 737 | **ADORA2B** | **-2,5** | NM_000676 | ILMN_1703946 |
| 738 | **ALDH2** | **-2,5** | NM_000690 | ILMN_1793859 |
| 739 | **SLITL2** | **-2,5** | NM_138440 | ILMN_1667295 |
| 740 | **TKT** | **-2,5** | NM_001064 | ILMN_1736597 |
| 741 | **ALPP** | **-2,5** | NM_001632 | ILMN_1693789 |
| 742 | **MLLT7** | **-2,5** | NM_005938 | ILMN_1712095 |
| 743 | **LEPREL1** | **-2,5** | NM_018192 | ILMN_1657373 |
| 744 | **GTF3C2** | **-2,5** | NM_001521 | ILMN_1746457 |
| 745 | **DLG7** | **-2,5** | NM_014750 | ILMN_1749829 |
| 746 | **FLJ20920** | **-2,5** | NM_025149 | ILMN_1711928 |
| 747 | **WEE1** | **-2,5** | NM_003390 | ILMN_1778561 |
| 748 | **INSIG2** | **-2,5** | NM_016133 | ILMN_1797946 |
| 749 | **SH2D4A** | **-2,5** | NM_022071 | ILMN_1679322 |
| 750 | **MATN2** | **-2,5** | NM_030583 | ILMN_1694840 |
| 751 | **SAFB** | **-2,5** | NM_002967 | ILMN_1722059 |
| 752 | **TPM1** | **-2,5** | NM_000366 | ILMN_1716687 |
| 753 | **TMEM41B** | **-2,5** | NM_015012 | ILMN_1678004 |
| 754 | **KBTBD2** | **-2,5** | NM_015483 | ILMN_1784540 |
| 755 | **CPS1** | **-2,5** | NM_001875 | ILMN_1792748 |
| 756 | **PPP1R13B** | **-2,5** | NM_015316 | ILMN_1748831 |
| 757 | **FLJ25476** | **-2,5** | NM_152493 | ILMN_1686968 |
| 758 | **DAG1** | **-2,5** | NM_004393 | ILMN_1658425 |
| 759 | **RCC2** | **-2,5** | NM_018715 | ILMN_1720124 |
| 760 | **SIN3A** | **-2,5** | NM_015477 | ILMN_1805996 |
| 761 | **DDX28** | **-2,5** | NM_018380 | ILMN_1741736 |
| 762 | **ATP7A** | **-2,5** | NM_000052 | ILMN_1808115 |
| 763 | **SLC17A5** | **-2,5** | NM_012434 | ILMN_1771317 |
| 764 | **SFRS1** | **-2,5** | NM_006924 | ILMN_1795341 |
| 765 | **CCDC64** | **-2,5** | NM_207311 | ILMN_1798021 |
| 766 | **CFLAR** | **-2,4** | NM_003879 | ILMN_1789830 |
| 767 | **CA9** | **-2,4** | NM_001216 | ILMN_1725139 |
| 768 | **TPARL** | **-2,4** | NM_018475 | ILMN_1773935 |
| 769 | **G3BP2** | **-2,4** | NM_012297 | ILMN_1720422 |
| 770 | **SERINC3** | **-2,4** | NM_198941 | ILMN_1815656 |
| 771 | **CRIPT** | **-2,4** | NM_014171 | ILMN_1813256 |
| 772 | **UNC84A** | **-2,4** | NM_025154 | ILMN_1772316 |
| 773 | **CALM3** | **-2,4** | NM_005184 | ILMN_1666385 |
| 774 | **DERL1** | **-2,4** | NM_024295 | ILMN_1695726 |
| 775 | **SURF4** | **-2,4** | NM_033161 | ILMN_1799055 |
| 776 | **FN3KRP** | **-2,4** | NM_024619 | ILMN_1652333 |
| 777 | **COG2** | **-2,4** | NM_007357 | ILMN_1776993 |
| 778 | **TSPAN6** | **-2,4** | NM_003270 | ILMN_1730998 |
| 779 | **PTBP1** | **-2,4** | NM_175847 | ILMN_1655154 |
| 780 | **ANKMY1** | **-2,4** | NM_016552 | ILMN_1675483 |
| 781 | **MAPK3** | **-2,4** | NM_002746 | ILMN_1812747 |
| 782 | **BIN1** | **-2,4** | NM_139348 | ILMN_1674160 |
| 783 | **MIDN** | **-2,4** | NM_177401 | ILMN_1746408 |
| 784 | **PHYH2** | **-2,4** | NM_012260 | ILMN_1723414 |
| 785 | **XBP1** | **-2,4** | NM_005080 | ILMN_1710675 |
| 786 | **ROD1** | **-2,4** | NM_005156 | ILMN_1768197 |
| 787 | **FBXO3** | **-2,4** | NM_012175 | ILMN_1749641 |
| 788 | **FLJ11806** | **-2,4** | NM_024824 | ILMN_1785292 |
| 789 | **CTGF** | **-2,4** | NM_001901 | ILMN_1699829 |
| 790 | **HAK** | **-2,4** | NM_052947 | ILMN_1811238 |
| 791 | **TULP4** | **-2,4** | NM_020245 | ILMN_1720965 |
| 792 | **UBE2N** | **-2,4** | NM_003348 | ILMN_1793651 |
| 793 | **DCUN1D1** | **-2,4** | NM_020640 | ILMN_1810719 |
| 794 | **RIN2** | **-2,4** | NM_018993 | ILMN_1769546 |
| 795 | **FOXF2** | **-2,4** | NM_001452 | ILMN_1683960 |
| 796 | **TSNAX** | **-2,4** | NM_005999 | ILMN_1713668 |
| 797 | **VEGFC** | **-2,4** | NM_005429 | ILMN_1701204 |
| 798 | **SLC7A1** | **-2,4** | NM_003045 | ILMN_1683859 |
| 799 | **FOXO1A** | **-2,4** | NM_002015 | ILMN_1738816 |
| 800 | **PPAPDC2** | **-2,4** | NM_203453 | ILMN_1802628 |
| 801 | **LIPA** | **-2,4** | NM_000235 | ILMN_1718063 |
| 802 | **C10orf9** | **-2,4** | NM_145012 | ILMN_1797528 |
| 803 | **KLHL7** | **-2,4** | NM_018846 | ILMN_1760792 |
| 804 | **PARP4** | **-2,4** | NM_006437 | ILMN_1776464 |
| 805 | **GALC** | **-2,4** | NM_000153 | ILMN_1799744 |
| 806 | **NMT1** | **-2,4** | NM_021079 | ILMN_1762678 |
| 807 | **C13orf23** | **-2,4** | NM_025138 | ILMN_1795128 |
| 808 | **DDX54** | **-2,4** | NM_024072 | ILMN_1812976 |
| 809 | **FGG** | **-2,4** | NM_021870 | ILMN_1737683 |
| 810 | **PP2447** | **-2,4** | NM_025204 | ILMN_1755737 |
| 811 | **FYB** | **-2,4** | NM_199335 | ILMN_1796537 |
| 812 | **C6orf211** | **-2,4** | NM_024573 | ILMN_1798108 |
| 813 | **C10orf47** | **-2,4** | NM_153256 | ILMN_1750338 |
| 814 | **HADHSC** | **-2,4** | NM_005327 | ILMN_1719906 |
| 815 | **SNX19** | **-2,4** | NM_014758 | ILMN_1658456 |
| 816 | **GOLPH2** | **-2,4** | NM_016548 | ILMN_1766405 |
| 817 | **PRSS3** | **-2,4** | NM_002771 | ILMN_1685699 |
| 818 | **TANK** | **-2,4** | NM_004180 | ILMN_1715069 |
| 819 | **CD99L2** | **-2,4** | NM_134446 | ILMN_1700681 |
| 820 | **BMP6** | **-2,4** | NM_001718 | ILMN_1747650 |
| 821 | **PPP1R10** | **-2,4** | NM_002714 | ILMN_1659058 |
| 822 | **ANKRA2** | **-2,4** | NM_023039 | ILMN_1710655 |
| 823 | **TOPBP1** | **-2,4** | NM_007027 | ILMN_1684929 |
| 824 | **PHTF1** | **-2,4** | NM_006608 | ILMN_1803464 |
| 825 | **HIST1H4H** | **-2,4** | NM_003543 | ILMN_1695199 |
| 826 | **C14orf135** | **-2,4** | NM_022495 | ILMN_1680781 |
| 827 | **C17orf56** | **-2,4** | NM_144679 | ILMN_1780025 |
| 828 | **PLDN** | **-2,4** | NM_012388 | ILMN_1733666 |
| 829 | **HIP2** | **-2,4** | NM_005339 | ILMN_1782954 |
| 830 | **GMDS** | **-2,4** | NM_001500 | ILMN_1711227 |
| 831 | **ASS** | **-2,4** | NM_000050 | ILMN_1708778 |
| 832 | **NSF** | **-2,4** | NM_006178 | ILMN_1680353 |
| 833 | **KIAA1688** | **-2,4** | NM_025251 | ILMN_1784436 |
| 834 | **TYSND1** | **-2,4** | NM_173555 | ILMN_1775677 |
| 835 | **VTN** | **-2,4** | NM_000638 | ILMN_1691127 |
| 836 | **WDR48** | **-2,4** | NM_020839 | ILMN_1762103 |
| 837 | **ASCIZ** | **-2,4** | NM_015251 | ILMN_1772540 |
| 838 | **DSC2** | **-2,4** | NM_004949 | ILMN_1782125 |
| 839 | **RAB11A** | **-2,4** | NM_004663 | ILMN_1712312 |
| 840 | **PPP1CB** | **-2,4** | NM_002709 | ILMN_1736942 |
| 841 | **DBT** | **-2,4** | NM_001918 | ILMN_1714990 |
| 842 | **C5orf13** | **-2,4** | NM_004772 | ILMN_1680738 |
| 843 | **ELMO1** | **-2,4** | NM_014800 | ILMN_1784320 |
| 844 | **COBL** | **-2,4** | NM_015198 | ILMN_1711888 |
| 845 | **ASB13** | **-2,4** | NM_024701 | ILMN_1654385 |
| 846 | **SFTPD** | **-2,4** | NM_003019 | ILMN_1768575 |
| 847 | **POLR3B** | **-2,4** | NM_018082 | ILMN_1767459 |
| 848 | **SLC44A1** | **-2,4** | NM_080546 | ILMN_1700695 |
| 849 | **NFIB** | **-2,4** | NM_005596 | ILMN_1778991 |
| 850 | **TMEM30B** | **-2,4** | NM_001017970 | ILMN_1752935 |
| 851 | **BTBD2** | **-2,4** | NM_017797 | ILMN_1701660 |
| 852 | **RUTBC1** | **-2,4** | NM_014853 | ILMN_1779171 |
| 853 | **SYNCRIP** | **-2,4** | NM_006372 | ILMN_1727740 |
| 854 | **PPP2R2C** | **-2,4** | NM_181876 | ILMN_1680507 |
| 855 | **BFAR** | **-2,3** | NM_016561 | ILMN_1814808 |
| 856 | **FBXO34** | **-2,3** | NM_017943 | ILMN_1765060 |
| 857 | **C21orf33** | **-2,3** | NM_004649 | ILMN_1737588 |
| 858 | **WDR1** | **-2,3** | NM_017491 | ILMN_1780036 |
| 859 | **BBX** | **-2,3** | NM_020235 | ILMN_1745415 |
| 860 | **PLCL3** | **-2,3** | NM_014996 | ILMN_1668409 |
| 861 | **ORM1** | **-2,3** | NM_000607 | ILMN_1696584 |
| 862 | **PPP4R1** | **-2,3** | NM_005134 | ILMN_1724544 |
| 863 | **RNF13** | **-2,3** | NM_183382 | ILMN_1685576 |
| 864 | **ACTR2** | **-2,3** | NM_001005386 | ILMN_1697774 |
| 865 | **BCLAF1** | **-2,3** | NM_014739 | ILMN_1775192 |
| 866 | **ALDH1A3** | **-2,3** | NM_000693 | ILMN_1807439 |
| 867 | **TGIF** | **-2,3** | NM_170695 | ILMN_1742906 |
| 868 | **ALG14** | **-2,3** | NM_144988 | ILMN_1743635 |
| 869 | **TMEM113** | **-2,3** | NM_025222 | ILMN_1679655 |
| 870 | **FNTB** | **-2,3** | NM_002028 | ILMN_1728517 |
| 871 | **GRN** | **-2,3** | NM_001012479 | ILMN_1811702 |
| 872 | **WDR60** | **-2,3** | NM_018051 | ILMN_1793290 |
| 873 | **C2orf29** | **-2,3** | NM_017546 | ILMN_1708906 |
| 874 | **HYAL1** | **-2,3** | NM_153281 | ILMN_1739813 |
| 875 | **ITGB1** | **-2,3** | NM_002211 | ILMN_1723467 |
| 876 | **ID1** | **-2,3** | NM_181353 | ILMN_1664861 |
| 877 | **DUS2L** | **-2,3** | NM_017803 | ILMN_1811650 |
| 878 | **SSH3** | **-2,3** | NM_017857 | ILMN_1755234 |
| 879 | **UBE2G1** | **-2,3** | NM_182682 | ILMN_1719039 |
| 880 | **ALS2** | **-2,3** | NM_020919 | ILMN_1750256 |
| 881 | **SLC20A2** | **-2,3** | NM_006749 | ILMN_1813763 |
| 882 | **ARPC5** | **-2,3** | NM_005717 | ILMN_1768394 |
| 883 | **PCAF** | **-2,3** | NM_003884 | ILMN_1704654 |
| 884 | **NR1H2** | **-2,3** | NM_007121 | ILMN_1691345 |
| 885 | **SLC24A6** | **-2,3** | NM_024959 | ILMN_1701655 |
| 886 | **OSBPL9** | **-2,3** | NM_024586 | ILMN_1785167 |
| 887 | **PTTG1IP** | **-2,3** | NM_004339 | ILMN_1802251 |
| 888 | **C1orf115** | **-2,3** | NM_024709 | ILMN_1674817 |
| 889 | **40057** | **-2,3** | NM_006640 | ILMN_1769118 |
| 890 | **AFAP** | **-2,3** | NM_198595 | ILMN_1701998 |
| 891 | **KCTD3** | **-2,3** | NM_016121 | ILMN_1800220 |
| 892 | **MYO5C** | **-2,3** | NM_018728 | ILMN_1808789 |
| 893 | **KIAA0528** | **-2,3** | NM_014802 | ILMN_1682572 |
| 894 | **CEP350** | **-2,3** | NM_014810 | ILMN_1742400 |
| 895 | **RGS11** | **-2,3** | NM_183337 | ILMN_1763704 |
| 896 | **ZCCHC11** | **-2,3** | NM_001009881 | ILMN_1655137 |
| 897 | **MGST3** | **-2,3** | NM_004528 | ILMN_1751956 |
| 898 | **C12orf26** | **-2,3** | NM_032230 | ILMN_1745497 |
| 899 | **MID1** | **-2,3** | NM_000381 | ILMN_1761858 |
| 900 | **C6orf85** | **-2,3** | NM_021945 | ILMN_1705116 |
| 901 | **PLXNB1** | **-2,3** | NM_002673 | ILMN_1742330 |
| 902 | **TSC22D4** | **-2,3** | NM_030935 | ILMN_1706609 |
| 903 | **TRUB2** | **-2,3** | NM_015679 | ILMN_1776682 |
| 904 | **ZNF207** | **-2,3** | NM_003457 | ILMN_1778177 |
| 905 | **MATN3** | **-2,3** | NM_002381 | ILMN_1663171 |
| 906 | **ALDH9A1** | **-2,3** | NM_000696 | ILMN_1761804 |
| 907 | **C22orf13** | **-2,3** | NM_031444 | ILMN_1764410 |
| 908 | **SFRS5** | **-2,3** | NM_006925 | ILMN_1761996 |
| 909 | **HSD17B12** | **-2,3** | NM_016142 | ILMN_1702168 |
| 910 | **ACY1** | **-2,3** | NM_000666 | ILMN_1683883 |
| 911 | **CHURC1** | **-2,3** | NM_145165 | ILMN_1732815 |
| 912 | **RAB8A** | **-2,3** | NM_005370 | ILMN_1760858 |
| 913 | **MTMR11** | **-2,3** | NM_181873 | ILMN_1769299 |
| 914 | **CGN** | **-2,3** | NM_020770 | ILMN_1746801 |
| 915 | **S100P** | **-2,3** | NM_005980 | ILMN_1801216 |
| 916 | **SLC36A4** | **-2,3** | NM_152313 | ILMN_1802348 |
| 917 | **BASP1** | **-2,3** | NM_006317 | ILMN_1651826 |
| 918 | **OMA1** | **-2,3** | NM_145243 | ILMN_1670079 |
| 919 | **TRPM4** | **-2,3** | NM_017636 | ILMN_1679401 |
| 920 | **DHCR24** | **-2,3** | NM_014762 | ILMN_1725510 |
| 921 | **TRIM31** | **-2,3** | NM_007028 | ILMN_1748685 |
| 922 | **RBMX** | **-2,3** | NM_002139 | ILMN_1723580 |
| 923 | **MPHOSPH1** | **-2,2** | NM_016195 | ILMN_1712452 |
| 924 | **SCAMP1** | **-2,2** | NM_052822 | ILMN_1728907 |
| 925 | **C10orf38** | **-2,2** | NM_001010924 | ILMN_1749868 |
| 926 | **ARL1** | **-2,2** | NM_001177 | ILMN_1813091 |
| 927 | **ACADM** | **-2,2** | NM_000016 | ILMN_1778104 |
| 928 | **KIAA1838** | **-2,2** | NM_032448 | ILMN_1679641 |
| 929 | **PRNP** | **-2,2** | NM_183079 | ILMN_1737988 |
| 930 | **RXRA** | **-2,2** | NM_002957 | ILMN_1687315 |
| 931 | **DKFZp434I1020** | **-2,2** | NM_194295 | ILMN_1685628 |
| 932 | **MCM8** | **-2,2** | NM_032485 | ILMN_1798581 |
| 933 | **ZMPSTE24** | **-2,2** | NM_005857 | ILMN_1656413 |
| 934 | **STAT1** | **-2,2** | NM_007315 | ILMN_1777325 |
| 935 | **CDA** | **-2,2** | NM_001785 | ILMN_1714592 |
| 936 | **CNOT6** | **-2,2** | NM_015455 | ILMN_1661290 |
| 937 | **C14orf100** | **-2,2** | NM_016475 | ILMN_1700276 |
| 938 | **37500** | **-2,2** | NM_001008491 | ILMN_1740252 |
| 939 | **EVI1** | **-2,2** | NM_005241 | ILMN_1803367 |
| 940 | **RASA1** | **-2,2** | NM_002890 | ILMN_1725312 |
| 941 | **TMEM50A** | **-2,2** | NM_014313 | ILMN_1745368 |
| 942 | **XRCC5** | **-2,2** | NM_021141 | ILMN_1754919 |
| 943 | **TAF1B** | **-2,2** | NM_005680 | ILMN_1667977 |
| 944 | **WDR39** | **-2,2** | NM_004804 | ILMN_1792837 |
| 945 | **EIF4G2** | **-2,2** | NM_001418 | ILMN_1761519 |
| 946 | **TM4SF18** | **-2,2** | NM_138786 | ILMN_1739170 |
| 947 | **MYADM** | **-2,2** | NM_001020820 | ILMN_1658528 |
| 948 | **STK24** | **-2,2** | NM_001032296 | ILMN_1655163 |
| 949 | **UPK1A** | **-2,2** | NM_007000 | ILMN_1655637 |
| 950 | **PSMF1** | **-2,2** | NM_178578 | ILMN_1671696 |
| 951 | **H3F3A** | **-2,2** | NM_002107 | ILMN_1656082 |
| 952 | **CRAT** | **-2,2** | NM_000755 | ILMN_1728671 |
| 953 | **C6orf192** | **-2,2** | NM_052831 | ILMN_1669831 |
| 954 | **TMED2** | **-2,2** | NM_006815 | ILMN_1654939 |
| 955 | **SAPS3** | **-2,2** | NM_018312 | ILMN_1732725 |
| 956 | **TMEM43** | **-2,2** | NM_024334 | ILMN_1698605 |
| 957 | **LYCAT** | **-2,2** | NM_001002257 | ILMN_1708081 |
| 958 | **RPL15** | **-2,2** | NM_002948 | ILMN_1762747 |
| 959 | **NT5DC1** | **-2,2** | NM_152729 | ILMN_1680673 |
| 960 | **TOP2A** | **-2,2** | NM_001067 | ILMN_1686097 |
| 961 | **WDR21A** | **-2,2** | NM_181340 | ILMN_1715563 |
| 962 | **TTC3** | **-2,2** | NM_003316 | ILMN_1728605 |
| 963 | **NCOA6** | **-2,2** | NM_014071 | ILMN_1695797 |
| 964 | **CFI** | **-2,2** | NM_000204 | ILMN_1727815 |
| 965 | **SPP1** | **-2,2** | NM_000582 | ILMN_1651354 |
| 966 | **GLRX** | **-2,2** | NM_002064 | ILMN_1737308 |
| 967 | **FLJ20397** | **-2,2** | NM_017802 | ILMN_1706824 |
| 968 | **BBS2** | **-2,2** | NM_031885 | ILMN_1767612 |
| 969 | **DCBLD2** | **-2,2** | NM_080927 | ILMN_1735499 |
| 970 | **C9orf88** | **-2,2** | NM_022833 | ILMN_1661755 |
| 971 | **LRRC45** | **-2,2** | NM_144999 | ILMN_1754325 |
| 972 | **TFDP1** | **-2,2** | NM_007111 | ILMN_1661717 |
| 973 | **ARHGAP12** | **-2,2** | NM_018287 | ILMN_1753500 |
| 974 | **TEX2** | **-2,2** | NM_018469 | ILMN_1731181 |
| 975 | **SCARB1** | **-2,2** | NM_005505 | ILMN_1668387 |
| 976 | **RAB5A** | **-2,2** | NM_004162 | ILMN_1808875 |
| 977 | **MGC52110** | **-2,2** | NM_001008215 | ILMN_1683065 |
| 978 | **LPHN2** | **-2,2** | NM_012302 | ILMN_1697548 |
| 979 | **F5** | **-2,2** | NM_000130 | ILMN_1709233 |
| 980 | **FLJ31951** | **-2,2** | NM_144726 | ILMN_1719951 |
| 981 | **STOM** | **-2,2** | NM_004099 | ILMN_1766657 |
| 982 | **KLF9** | **-2,2** | NM_001206 | ILMN_1778523 |
| 983 | **STX5A** | **-2,2** | NM_003164 | ILMN_1749345 |
| 984 | **TRAM2** | **-2,2** | NM_012288 | ILMN_1788783 |
| 985 | **RTN3** | **-2,2** | NM_006054 | ILMN_1658643 |
| 986 | **TGFBR3** | **-2,2** | NM_003243 | ILMN_1784287 |
| 987 | **FCGRT** | **-2,2** | NM_004107 | ILMN_1705302 |
| 988 | **PKP4** | **-2,2** | NM_003628 | ILMN_1749410 |
| 989 | **CAPN1** | **-2,2** | NM_005186 | ILMN_1705261 |
| 990 | **CCNDBP1** | **-2,2** | NM_037370 | ILMN_1702247 |
| 991 | **PMM2** | **-2,2** | NM_000303 | ILMN_1785336 |
| 992 | **STARD10** | **-2,2** | NM_006645 | ILMN_1717052 |
| 993 | **KIAA0174** | **-2,1** | NM_014761 | ILMN_1740351 |
| 994 | **ANKRD13C** | **-2,1** | NM_030816 | ILMN_1687264 |
| 995 | **PPHLN1** | **-2,1** | NM_201440 | ILMN_1791093 |
| 996 | **TRIM68** | **-2,1** | NM_018073 | ILMN_1694174 |
| 997 | **GLS** | **-2,1** | NM_014905 | ILMN_1798791 |
| 998 | **FAM45A** | **-2,1** | NM_207009 | ILMN_1691760 |
| 999 | **MORF4L1** | **-2,1** | NM_006791 | ILMN_1760676 |
| 1000 | **WDR59** | **-2,1** | NM_030581 | ILMN_1795428 |
| 1001 | **TMEM5** | **-2,1** | NM_014254 | ILMN_1689704 |
| 1002 | **LANCL2** | **-2,1** | NM_018697 | ILMN_1708009 |
| 1003 | **SLC7A7** | **-2,1** | NM_003982 | ILMN_1810275 |
| 1004 | **PLS3** | **-2,1** | NM_005032 | ILMN_1785265 |
| 1005 | **ZNF467** | **-2,1** | NM_207336 | ILMN_1779015 |
| 1006 | **GGPS1** | **-2,1** | NM_004837 | ILMN_1692276 |
| 1007 | **CERK** | **-2,1** | NM_182661 | ILMN_1767475 |
| 1008 | **NALP2** | **-2,1** | NM_017852 | ILMN_1664894 |
| 1009 | **PMM1** | **-2,1** | NM_002676 | ILMN_1780236 |
| 1010 | **HIST1H2BD** | **-2,1** | NM_138720 | ILMN_1651496 |
| 1011 | **NCOA1** | **-2,1** | NM_147223 | ILMN_1669033 |
| 1012 | **KIAA1287** | **-2,1** | NM_020748 | ILMN_1686553 |
| 1013 | **WDR67** | **-2,1** | NM_145647 | ILMN_1744240 |
| 1014 | **KDELR3** | **-2,1** | NM_006855 | ILMN_1722820 |
| 1015 | **USH1C** | **-2,1** | NM_005709 | ILMN_1668946 |
| 1016 | **LOC129285** | **-2,1** | NM_152994 | ILMN_1667356 |
| 1017 | **EPIM** | **-2,1** | NM_001980 | ILMN_1747775 |
| 1018 | **CHKA** | **-2,1** | NM_001277 | ILMN_1658504 |
| 1019 | **THAP11** | **-2,1** | NM_020457 | ILMN_1780699 |
| 1020 | **EIF2AK4** | **-2,1** | NM_001013703 | ILMN_1755114 |
| 1021 | **LZTR1** | **-2,1** | NM_006767 | ILMN_1805161 |
| 1022 | **ZNF532** | **-2,1** | NM_018181 | ILMN_1731358 |
| 1023 | **VPS41** | **-2,1** | NM_014396 | ILMN_1768486 |
| 1024 | **PRKRIR** | **-2,1** | NM_004705 | ILMN_1655622 |
| 1025 | **GLUD1** | **-2,1** | NM_005271 | ILMN_1713756 |
| 1026 | **C10orf26** | **-2,1** | NM_017787 | ILMN_1658830 |
| 1027 | **TRIM33** | **-2,1** | NM_033020 | ILMN_1682316 |
| 1028 | **TSPAN3** | **-2,1** | NM_198902 | ILMN_1790549 |
| 1029 | **ARHGAP27** | **-2,1** | NM_199282 | ILMN_1734652 |
| 1030 | **CADPS2** | **-2,1** | NM_017954 | ILMN_1684461 |
| 1031 | **CSNK1G3** | **-2,1** | NM_001031812 | ILMN_1652024 |
| 1032 | **DSCR1** | **-2,1** | NM_203417 | ILMN_1814135 |
| 1033 | **SQLE** | **-2,1** | NM_003129 | ILMN_1772241 |
| 1034 | **ZBTB33** | **-2,1** | NM_006777 | ILMN_1673138 |
| 1035 | **COBLL1** | **-2,1** | NM_014900 | ILMN_1761260 |
| 1036 | **CALM2** | **-2,1** | NM_001743 | ILMN_1687858 |
| 1037 | **SGCE** | **-2,1** | NM_003919 | ILMN_1674620 |
| 1038 | **SMARCA3** | **-2,1** | NM_003071 | ILMN_1798588 |
| 1039 | **TMEM123** | **-2,1** | NM_052932 | ILMN_1724139 |
| 1040 | **C14orf129** | **-2,1** | NM_016472 | ILMN_1712748 |
| 1041 | **TMED5** | **-2,1** | NM_016040 | ILMN_1803279 |
| 1042 | **SLC12A9** | **-2,1** | NM_020246 | ILMN_1695962 |
| 1043 | **FUK** | **-2,1** | NM_145059 | ILMN_1704195 |
| 1044 | **MAT2B** | **-2,1** | NM_013283 | ILMN_1688437 |
| 1045 | **CLN5** | **-2,1** | NM_006493 | ILMN_1778203 |
| 1046 | **RAD21** | **-2,1** | NM_006265 | ILMN_1748578 |
| 1047 | **KLHDC5** | **-2,1** | NM_020782 | ILMN_1727134 |
| 1048 | **UBE2D3** | **-2,1** | NM_181890 | ILMN_1682443 |
| 1049 | **SFRS7** | **-2,1** | NM_001031684 | ILMN_1778836 |
| 1050 | **C13orf8** | **-2,1** | NM_032436 | ILMN_1729976 |
| 1051 | **PACSIN2** | **-2,1** | NM_007229 | ILMN_1702396 |
| 1052 | **ABLIM1** | **-2,1** | NM_006720 | ILMN_1785424 |
| 1053 | **AHR** | **-2,1** | NM_001621 | ILMN_1812640 |
| 1054 | **RRAGB** | **-2,1** | NM_006064 | ILMN_1741219 |
| 1055 | **BET1** | **-2,1** | NM_005868 | ILMN_1684042 |
| 1056 | **STAU2** | **-2,1** | NM_014393 | ILMN_1769720 |
| 1057 | **EXT1** | **-2,1** | NM_000127 | ILMN_1794343 |
| 1058 | **XPO1** | **-2,1** | NM_003400 | ILMN_1725121 |
| 1059 | **POP4** | **-2,0** | NM_006627 | ILMN_1652686 |
| 1060 | **IL18** | **-2,0** | NM_001562 | ILMN_1778457 |
| 1061 | **AXL** | **-2,0** | NM_001699 | ILMN_1701877 |
| 1062 | **RXRB** | **-2,0** | NM_021976 | ILMN_1774074 |
| 1063 | **SH3BGRL2** | **-2,0** | NM_031469 | ILMN_1762764 |
| 1064 | **LTB4DH** | **-2,0** | NM_012212 | ILMN_1704531 |
| 1065 | **MAP4K4** | **-2,0** | NM_145686 | ILMN_1797745 |
| 1066 | **TMEM60** | **-2,0** | NM_032936 | ILMN_1752213 |
| 1067 | **WIPI1** | **-2,0** | NM_017983 | ILMN_1781386 |
| 1068 | **LTBR** | **-2,0** | NM_002342 | ILMN_1667476 |
| 1069 | **ZNF42** | **-2,0** | NM_198055 | ILMN_1749838 |
| 1070 | **PAIP2** | **-2,0** | NM_001033112 | ILMN_1784753 |
| 1071 | **PTPN4** | **-2,0** | NM_002830 | ILMN_1793549 |
| 1072 | **PRODH2** | **-2,0** | NM_021232 | ILMN_1686259 |
| 1073 | **C6orf55** | **-2,0** | NM_016485 | ILMN_1690233 |
| 1074 | **MBNL1** | **-2,0** | NM_207293 | ILMN_1807304 |
| 1075 | **LEMD2** | **-2,0** | NM_181336 | ILMN_1680860 |
| 1076 | **YIPF1** | **-2,0** | NM_018982 | ILMN_1803564 |
| 1077 | **BCL11B** | **-2,0** | NM_022898 | ILMN_1665761 |
| 1078 | **TP53INP1** | **-2,0** | NM_033285 | ILMN_1714108 |
| 1079 | **RPUSD2** | **-2,0** | NM_152260 | ILMN_1730077 |
| 1080 | **SNX6** | **-2,0** | NM_152233 | ILMN_1807873 |
| 1081 | **MAN2A1** | **-2,0** | NM_002372 | ILMN_1809402 |
| 1082 | **MARCH7** | **-2,0** | NM_022826 | ILMN_1717337 |
| 1083 | **RDH14** | **-2,0** | NM_020905 | ILMN_1705469 |
| 1084 | **TNRC6B** | **-2,0** | NM_001024843 | ILMN_1726786 |
| 1085 | **ARHGAP1** | **-2,0** | NM_004308 | ILMN_1733068 |
| 1086 | **UBXD2** | **-2,0** | NM_014607 | ILMN_1781097 |
| 1087 | **CIRBP** | **-2,0** | NM_001280 | ILMN_1674661 |
| 1088 | **MFGE8** | **-2,0** | NM_005928 | ILMN_1756071 |
| 1089 | **DYNC1LI2** | **-2,0** | NM_006141 | ILMN_1783448 |
| 1090 | **DNAJB9** | **-2,0** | NM_012328 | ILMN_1773742 |
| 1091 | **MKLN1** | **-2,0** | NM_013255 | ILMN_1742578 |
| 1092 | **PRKAR1A** | **-2,0** | NM_002734 | ILMN_1738632 |
| 1093 | **HNRPH1** | **-2,0** | NM_005520 | ILMN_1702273 |
| 1094 | **SF3A1** | **-2,0** | NM_001005409 | ILMN_1697286 |
| 1095 | **TMEM98** | **-2,0** | NM_015544 | ILMN_1779182 |
| 1096 | **UBE3A** | **-2,0** | NM_130839 | ILMN_1764549 |
| 1097 | **KIFAP3** | **-2,0** | NM_014970 | ILMN_1697884 |
| 1098 | **SH3KBP1** | **-2,0** | NM_001024666 | ILMN_1808501 |
| 1099 | **ACLY** | **-2,0** | NM_198830 | ILMN_1749014 |
| 1100 | **MAL2** | **-2,0** | NM_052886 | ILMN_1770653 |
| 1101 | **C19orf2** | **-2,0** | NM_134447 | ILMN_1798728 |
| 1102 | **TSPAN17** | **-2,0** | NM_012171 | ILMN_1777881 |
| 1103 | **MDFIC** | **-2,0** | NM_199072 | ILMN_1717366 |
| 1104 | **SCARB2** | **-2,0** | NM_005506 | ILMN_1814726 |
| 1105 | **MED6** | **-2,0** | NM_005466 | ILMN_1654543 |
| 1106 | **PGM1** | **-2,0** | NM_002633 | ILMN_1800659 |
| 1107 | **KIAA0376** | **-2,0** | NM_015330 | ILMN_1779185 |
| 1108 | **FAM46A** | **-2,0** | NM_017633 | ILMN_1740466 |
| 1109 | **CCNI** | **-2,0** | NM_006835 | ILMN_1691942 |
| 1110 | **RBL2** | **-2,0** | NM_005611 | ILMN_1756999 |
| 1111 | **CAPZA2** | **-2,0** | NM_006136 | ILMN_1768870 |
| 1112 | **LYN** | **-2,0** | NM_002350 | ILMN_1781155 |
| 1113 | **LRRC1** | **-2,0** | NM_018214 | ILMN_1755383 |
| 1114 | **BSG** | **-2,0** | NM_198589 | ILMN_1778374 |
| 1115 | **SHMT1** | **-2,0** | NM_148918 | ILMN_1811933 |
| 1116 | **IMP3** | **-2,0** | NM_018285 | ILMN_1733696 |
| 1117 | **GALNT11** | **-2,0** | NM_022087 | ILMN_1651819 |
| 1118 | **CYP2S1** | **-1,9** | NM_030622 | ILMN_1705403 |
| 1119 | **M6PRBP1** | **-1,9** | NM_005817 | ILMN_1660021 |
| 1120 | **CEBPD** | **-1,9** | NM_005195 | ILMN_1782050 |
| 1121 | **C10orf86** | **-1,9** | NM_017615 | ILMN_1679134 |
| 1122 | **TMEM51** | **-1,9** | NM_018022 | ILMN_1674985 |
| 1123 | **C10orf57** | **-1,9** | NM_025125 | ILMN_1672717 |
| 1124 | **NFYB** | **-1,9** | NM_006166 | ILMN_1750005 |
| 1125 | **IARS2** | **-1,9** | NM_018060 | ILMN_1671207 |
| 1126 | **HSBP1** | **-1,9** | NM_001537 | ILMN_1667030 |
| 1127 | **DNAJA1** | **-1,9** | NM_001539 | ILMN_1672496 |
| 1128 | **C9orf10** | **-1,9** | NM_014612 | ILMN_1721089 |
| 1129 | **CLDN1** | **-1,9** | NM_021101 | ILMN_1724686 |
| 1130 | **HOXB5** | **-1,9** | NM_002147 | ILMN_1674908 |
| 1131 | **QARS** | **-1,9** | NM_005051 | ILMN_1763080 |
| 1132 | **ATXN2** | **-1,9** | NM_002973 | ILMN_1743829 |
| 1133 | **SFT2D3** | **-1,9** | NM_032740 | ILMN_1765746 |
| 1134 | **MAPK6** | **-1,9** | NM_002748 | ILMN_1757287 |
| 1135 | **CAP2** | **-1,9** | NM_006366 | ILMN_1691237 |
| 1136 | **RAPGEF1** | **-1,9** | NM_005312 | ILMN_1769412 |
| 1137 | **FLNB** | **-1,9** | NM_001457 | ILMN_1752635 |
| 1138 | **RNF38** | **-1,9** | NM_194331 | ILMN_1793616 |
| 1139 | **SSR1** | **-1,9** | NM_003144 | ILMN_1750693 |
| 1140 | **C1orf106** | **-1,9** | NM_018265 | ILMN_1713952 |
| 1141 | **ARL2BP** | **-1,9** | NM_012106 | ILMN_1755391 |
| 1142 | **RSPRY1** | **-1,9** | NM_133368 | ILMN_1763694 |
| 1143 | **CNOT1** | **-1,9** | NM_016284 | ILMN_1669206 |
| 1144 | **AASDH** | **-1,9** | NM_181806 | ILMN_1784269 |
| 1145 | **ARHGEF18** | **-1,9** | NM_015318 | ILMN_1664016 |
| 1146 | **RALGDS** | **-1,9** | NM_006266 | ILMN_1699856 |
| 1147 | **SLC23A2** | **-1,9** | NM_005116 | ILMN_1746578 |
| 1148 | **ARPC1A** | **-1,9** | NM_006409 | ILMN_1759915 |
| 1149 | **H2AFZ** | **-1,9** | NM_002106 | ILMN_1707858 |
| 1150 | **C11orf2** | **-1,9** | NM_013265 | ILMN_1794828 |
| 1151 | **ATP2A2** | **-1,9** | NM_170665 | ILMN_1815666 |
| 1152 | **ADCY3** | **-1,9** | NM_004036 | ILMN_1676893 |
| 1153 | **CFD** | **-1,9** | NM_001928 | ILMN_1777190 |
| 1154 | **LAMC1** | **-1,9** | NM_002293 | ILMN_1810852 |
| 1155 | **TMEM97** | **-1,9** | NM_014573 | ILMN_1710962 |
| 1156 | **VCL** | **-1,9** | NM_003373 | ILMN_1795429 |
| 1157 | **COASY** | **-1,9** | NM_025233 | ILMN_1753498 |
| 1158 | **FLJ10081** | **-1,9** | NM_017991 | ILMN_1663571 |
| 1159 | **OSBP** | **-1,9** | NM_002556 | ILMN_1706376 |
| 1160 | **OTUD5** | **-1,9** | NM_017602 | ILMN_1726470 |
| 1161 | **NCKAP1** | **-1,9** | NM_013436 | ILMN_1747392 |
| 1162 | **SIDT2** | **-1,9** | NM_015996 | ILMN_1791912 |
| 1163 | **TXNDC5** | **-1,9** | NM_030810 | ILMN_1788108 |
| 1164 | **PGRMC1** | **-1,9** | NM_006667 | ILMN_1684771 |
| 1165 | **KLHL5** | **-1,9** | NM_001007075 | ILMN_1706687 |
| 1166 | **ATP1B1** | **-1,9** | NM_001677 | ILMN_1736862 |
| 1167 | **ALDH1A1** | **-1,9** | NM_000689 | ILMN_1709348 |
| 1168 | **PHLDB1** | **-1,9** | NM_015157 | ILMN_1666819 |
| 1169 | **BSDC1** | **-1,9** | NM_018045 | ILMN_1734483 |
| 1170 | **CTSD** | **-1,9** | NM_001909 | ILMN_1714546 |
| 1171 | **RAB5B** | **-1,9** | NM_002868 | ILMN_1752582 |
| 1172 | **TGOLN2** | **-1,9** | NM_006464 | ILMN_1651735 |
| 1173 | **IDH1** | **-1,9** | NM_005896 | ILMN_1696432 |
| 1174 | **C7orf27** | **-1,9** | NM_152743 | ILMN_1804498 |
| 1175 | **PLA2G10** | **-1,8** | NM_003561 | ILMN_1762561 |
| 1176 | **MORF4L2** | **-1,8** | NM_012286 | ILMN_1782875 |
| 1177 | **RCN1** | **-1,8** | NM_002901 | ILMN_1800276 |
| 1178 | **C16orf63** | **-1,8** | NM_144600 | ILMN_1790650 |
| 1179 | **GULP1** | **-1,8** | NM_016315 | ILMN_1802690 |
| 1180 | **CNOT2** | **-1,8** | NM_014515 | ILMN_1772651 |
| 1181 | **YWHAB** | **-1,8** | NM_139323 | ILMN_1694385 |
| 1182 | **FNTA** | **-1,8** | NM_002027 | ILMN_1721704 |
| 1183 | **EXOC8** | **-1,8** | NM_175876 | ILMN_1721648 |
| 1184 | **LARP1** | **-1,8** | NM_033551 | ILMN_1692770 |
| 1185 | **EIF4B** | **-1,8** | NM_001417 | ILMN_1655497 |
| 1186 | **GLDC** | **-1,8** | NM_000170 | ILMN_1806754 |
| 1187 | **MAP7** | **-1,8** | NM_003980 | ILMN_1712719 |
| 1188 | **PLOD2** | **-1,8** | NM_182943 | ILMN_1771599 |
| 1189 | **PPP3R1** | **-1,8** | NM_000945 | ILMN_1796962 |
| 1190 | **DSTN** | **-1,8** | NM_006870 | ILMN_1706426 |
| 1191 | **ADIPOR2** | **-1,8** | NM_024551 | ILMN_1750651 |
| 1192 | **SLC37A4** | **-1,8** | NM_001467 | ILMN_1678678 |
| 1193 | **GRAMD1A** | **-1,8** | NM_020895 | ILMN_1737157 |
| 1194 | **PRDX3** | **-1,8** | NM_014098 | ILMN_1737800 |
| 1195 | **MYNN** | **-1,8** | NM_018657 | ILMN_1672287 |
| 1196 | **LITAF** | **-1,8** | NM_004862 | ILMN_1713934 |
| 1197 | **DEDD2** | **-1,8** | NM_133328 | ILMN_1768031 |
| 1198 | **ACTB** | **-1,8** | NM_001101 | ILMN_2038777 |
| 1199 | **GRSF1** | **-1,8** | NM_002092 | ILMN_1806601 |
| 1200 | **TNFRSF21** | **-1,8** | NM_014452 | ILMN_1738006 |
| 1201 | **BET1L** | **-1,8** | NM_016526 | ILMN_1692773 |
| 1202 | **WASL** | **-1,8** | NM_003941 | ILMN_1666004 |
| 1203 | **DENR** | **-1,8** | NM_003677 | ILMN_1760954 |
| 1204 | **COPB** | **-1,8** | NM_016451 | ILMN_1699112 |
| 1205 | **ECHDC2** | **-1,7** | NM_018281 | ILMN_1671568 |
| 1206 | **QPCT** | **-1,7** | NM_012413 | ILMN_1741727 |
| 1207 | **KIAA0152** | **-1,7** | NM_014730 | ILMN_1657495 |
| 1208 | **CAT** | **-1,7** | NM_001752 | ILMN_1805905 |
| 1209 | **LHFPL2** | **-1,7** | NM_005779 | ILMN_1811077 |
| 1210 | **SUMO3** | **-1,7** | NM_006936 | ILMN_1725642 |
| 1211 | **NCLN** | **-1,7** | NM_020170 | ILMN_1689959 |
| 1212 | **KRT7** | **-1,7** | NM_005556 | ILMN_1801661 |
| 1213 | **SERPINB6** | **-1,7** | NM_004568 | ILMN_1712400 |
| 1214 | **HNRPD** | **-1,7** | NM_001003810 | ILMN_1751368 |
| 1215 | **CCAR1** | **-1,7** | NM_018237 | ILMN_1746208 |
| 1216 | **HSU79303** | **-1,7** | NM_013301 | ILMN_1682567 |
| 1217 | **DBNL** | **-1,7** | NM_014063 | ILMN_1698307 |
| 1218 | **GTPBP6** | **-1,7** | NM_012227 | ILMN_1776080 |
| 1219 | **AUH** | **-1,7** | NM_001698 | ILMN_1740349 |
| 1220 | **LOC339123** | **-1,7** | NM_001005920 | ILMN_1687921 |
| 1221 | **ADM** | **-1,7** | NM_001124 | ILMN_1708934 |
| 1222 | **PTPN1** | **-1,7** | NM_002827 | ILMN_1681591 |
| 1223 | **PTPRM** | **-1,7** | NM_002845 | ILMN_1744937 |
| 1224 | **BRD9** | **-1,7** | NM_023924 | ILMN_1651405 |
| 1225 | **C1orf181** | **-1,7** | NM_017953 | ILMN_1668540 |
| 1226 | **EIF5** | **-1,7** | NM_001969 | ILMN_1815733 |
| 1227 | **DEFB1** | **-1,7** | NM_005218 | ILMN_1686573 |
| 1228 | **ANXA7** | **-1,7** | NM_004034 | ILMN_1703791 |
| 1229 | **GOLGA7** | **-1,7** | NM_001002296 | ILMN_1778673 |
| 1230 | **DERA** | **-1,6** | NM_015954 | ILMN_1811551 |
| 1231 | **TBCE** | **-1,6** | NM_003193 | ILMN_1725183 |
| 1232 | **PTPLAD1** | **-1,6** | NM_016395 | ILMN_1743065 |
| 1233 | **TMBIM4** | **-1,6** | NM_016056 | ILMN_1664750 |
| 1234 | **CUEDC1** | **-1,6** | NM_017949 | ILMN_1676665 |
| 1235 | **TXN** | **1,5** | NM_003329 | ILMN_1343293 |
| 1236 | **CIRH1A** | **1,5** | NM_032830 | ILMN_1796235 |
| 1237 | **SSRP1** | **1,5** | NM_003146 | ILMN_1689642 |
| 1238 | **STK4** | **1,6** | NM_006282 | ILMN_1711383 |
| 1239 | **RFC5** | **1,6** | NM_007370 | ILMN_1659364 |
| 1240 | **RBM15** | **1,6** | NM_022768 | ILMN_1666739 |
| 1241 | **MRPS11** | **1,6** | NM_176805 | ILMN_1722905 |
| 1242 | **TBL3** | **1,6** | NM_006453 | ILMN_1657640 |
| 1243 | **CPNE1** | **1,6** | NM_152931 | ILMN_1670841 |
| 1244 | **UBE2E3** | **1,6** | NM_006357 | ILMN_1809652 |
| 1245 | **GNL2** | **1,6** | NM_013285 | ILMN_1761113 |
| 1246 | **LOC124446** | **1,6** | NM_194280 | ILMN_1737644 |
| 1247 | **ZNF668** | **1,7** | NM_024706 | ILMN_1739236 |
| 1248 | **CDK7** | **1,7** | NM_001799 | ILMN_1778917 |
| 1249 | **SNX5** | **1,7** | NM_152227 | ILMN_1673676 |
| 1250 | **CDK4** | **1,7** | NM_000075 | ILMN_1689001 |
| 1251 | **FAM82C** | **1,7** | NM_018145 | ILMN_1727309 |
| 1252 | **ZNF34** | **1,7** | NM_030580 | ILMN_1687981 |
| 1253 | **AGPS** | **1,7** | NM_003659 | ILMN_1750465 |
| 1254 | **GNPDA1** | **1,7** | NM_005471 | ILMN_1784709 |
| 1255 | **AATF** | **1,7** | NM_012138 | ILMN_1703743 |
| 1256 | **ZNHIT1** | **1,7** | NM_006349 | ILMN_1741491 |
| 1257 | **U2AF2** | **1,7** | NM_007279 | ILMN_1768930 |
| 1258 | **LRRC28** | **1,7** | NM_144598 | ILMN_1794968 |
| 1259 | **COMMD3** | **1,7** | NM_012071 | ILMN_1690392 |
| 1260 | **HDGF** | **1,7** | NM_004494 | ILMN_1765621 |
| 1261 | **COX5B** | **1,7** | NM_001862 | ILMN_1737236 |
| 1262 | **RAP1GDS1** | **1,7** | NM_021159 | ILMN_1806266 |
| 1263 | **GSTO2** | **1,7** | NM_183239 | ILMN_1740234 |
| 1264 | **CRIP2** | **1,7** | NM_001312 | ILMN_1694432 |
| 1265 | **C3orf31** | **1,7** | NM_138807 | ILMN_1793724 |
| 1266 | **ZNF695** | **1,7** | NM_020394 | ILMN_1747943 |
| 1267 | **THEM2** | **1,7** | NM_018473 | ILMN_1797336 |
| 1268 | **B2M** | **1,7** | NM_004048 | ILMN_1725427 |
| 1269 | **RCC1** | **1,7** | NM_001269 | ILMN_1707493 |
| 1270 | **SLC25A26** | **1,7** | NM_001009937 | ILMN_1777976 |
| 1271 | **ATG3** | **1,7** | NM_022488 | ILMN_1769566 |
| 1272 | **C10orf12** | **1,7** | NM_015652 | ILMN_1665508 |
| 1273 | **TOMM34** | **1,7** | NM_006809 | ILMN_1721128 |
| 1274 | **QDPR** | **1,7** | NM_000320 | ILMN_1672443 |
| 1275 | **KCNJ16** | **1,7** | NM_018658 | ILMN_1736045 |
| 1276 | **C6orf75** | **1,7** | NM_001031712 | ILMN_1805481 |
| 1277 | **SUCLG1** | **1,7** | NM_003849 | ILMN_1779616 |
| 1278 | **PTTG1** | **1,7** | NM_004219 | ILMN_1753196 |
| 1279 | **COX6B1** | **1,7** | NM_001863 | ILMN_1781094 |
| 1280 | **KARS** | **1,7** | NM_005548 | ILMN_1777584 |
| 1281 | **VHL** | **1,7** | NM_000551 | ILMN_1801984 |
| 1282 | **IMPDH2** | **1,7** | NM_000884 | ILMN_1705737 |
| 1283 | **MGC15523** | **1,7** | NM_138570 | ILMN_1759743 |
| 1284 | **ATP5H** | **1,7** | NM_006356 | ILMN_1666372 |
| 1285 | **LRRFIP2** | **1,7** | NM_006309 | ILMN_1772329 |
| 1286 | **PAK1** | **1,7** | NM_002576 | ILMN_1767365 |
| 1287 | **SNAPC2** | **1,7** | NM_003083 | ILMN_1698478 |
| 1288 | **ARHGEF2** | **1,7** | NM_004723 | ILMN_1703477 |
| 1289 | **SYP** | **1,7** | NM_003179 | ILMN_1701483 |
| 1290 | **CCDC53** | **1,7** | NM_016053 | ILMN_1715569 |
| 1291 | **MORC4** | **1,7** | NM_024657 | ILMN_1721526 |
| 1292 | **SIPA1L1** | **1,7** | NM_015556 | ILMN_1692023 |
| 1293 | **GIT1** | **1,7** | NM_014030 | ILMN_1733155 |
| 1294 | **ATP6V0B** | **1,7** | NM_004047 | ILMN_1721391 |
| 1295 | **C10orf11** | **1,7** | NM_032024 | ILMN_1783247 |
| 1296 | **GBL** | **1,7** | NM_022372 | ILMN_1789240 |
| 1297 | **UBE2M** | **1,7** | NM_003969 | ILMN_1701331 |
| 1298 | **HSC20** | **1,7** | NM_172002 | ILMN_1771921 |
| 1299 | **HSPD1** | **1,7** | NM_002156 | ILMN_1784367 |
| 1300 | **C16orf45** | **1,7** | NM_033201 | ILMN_1687821 |
| 1301 | **NRAS** | **1,7** | NM_002524 | ILMN_1775759 |
| 1302 | **NFE2L1** | **1,7** | NM_003204 | ILMN_1739450 |
| 1303 | **EIF2B2** | **1,7** | NM_014239 | ILMN_1713380 |
| 1304 | **C1orf35** | **1,7** | NM_024319 | ILMN_1790202 |
| 1305 | **NR2C2** | **1,7** | NM_003298 | ILMN_1724479 |
| 1306 | **GRPEL1** | **1,7** | NM_025196 | ILMN_1670817 |
| 1307 | **CPSF1** | **1,8** | NM_013291 | ILMN_1654545 |
| 1308 | **KIAA1622** | **1,8** | NM_020958 | ILMN_1660356 |
| 1309 | **VARSL** | **1,8** | NM_020442 | ILMN_1737585 |
| 1310 | **KIAA0913** | **1,8** | NM_015037 | ILMN_1669433 |
| 1311 | **SDF2L1** | **1,8** | NM_022044 | ILMN_1749213 |
| 1312 | **MRPL39** | **1,8** | NM_017446 | ILMN_1726391 |
| 1313 | **SUPT5H** | **1,8** | NM_003169 | ILMN_1703866 |
| 1314 | **BARD1** | **1,8** | NM_000465 | ILMN_1785340 |
| 1315 | **FLJ36070** | **1,8** | NM_182574 | ILMN_1718770 |
| 1316 | **RBMX2** | **1,8** | NM_016024 | ILMN_1678203 |
| 1317 | **VPS28** | **1,8** | NM_016208 | ILMN_1790797 |
| 1318 | **SYPL1** | **1,8** | NM_182715 | ILMN_1691458 |
| 1319 | **C1orf24** | **1,8** | NM_022083 | ILMN_1810725 |
| 1320 | **C16orf44** | **1,8** | NM_024731 | ILMN_1703314 |
| 1321 | **RPL7A** | **1,8** | NM_000972 | ILMN_1740749 |
| 1322 | **ZGPAT** | **1,8** | NM_032527 | ILMN_1794643 |
| 1323 | **THUMPD2** | **1,8** | NM_025264 | ILMN_1787511 |
| 1324 | **SFRP1** | **1,8** | NM_003012 | ILMN_1775728 |
| 1325 | **FKBP8** | **1,8** | NM_012181 | ILMN_1733947 |
| 1326 | **CACYBP** | **1,8** | NM_014412 | ILMN_1726574 |
| 1327 | **ProSAPiP1** | **1,8** | NM_014731 | ILMN_1736154 |
| 1328 | **PSMB3** | **1,8** | NM_002795 | ILMN_1748651 |
| 1329 | **PTPLA** | **1,8** | NM_014241 | ILMN_1725791 |
| 1330 | **ATR** | **1,8** | NM_001184 | ILMN_1716460 |
| 1331 | **SURF1** | **1,8** | NM_003172 | ILMN_1663407 |
| 1332 | **LGALS1** | **1,8** | NM_002305 | ILMN_1723978 |
| 1333 | **TRAPPC1** | **1,8** | NM_021210 | ILMN_1716913 |
| 1334 | **CRB3** | **1,8** | NM_139161 | ILMN_1754635 |
| 1335 | **ZNF297B** | **1,8** | NM_014007 | ILMN_1731113 |
| 1336 | **UBTD1** | **1,8** | NM_024954 | ILMN_1794914 |
| 1337 | **DHX30** | **1,8** | NM_014966 | ILMN_1795218 |
| 1338 | **RUFY3** | **1,8** | NM_014961 | ILMN_1730931 |
| 1339 | **PSMB2** | **1,8** | NM_002794 | ILMN_1764794 |
| 1340 | **CCDC12** | **1,8** | NM_144716 | ILMN_1725071 |
| 1341 | **TBPL1** | **1,8** | NM_004865 | ILMN_1708147 |
| 1342 | **TUBB** | **1,8** | NM_178014 | ILMN_1665583 |
| 1343 | **PSME2** | **1,8** | NM_002818 | ILMN_1786612 |
| 1344 | **LMTK2** | **1,8** | NM_014916 | ILMN_1724315 |
| 1345 | **TUBG1** | **1,8** | NM_001070 | ILMN_1695731 |
| 1346 | **TAP1** | **1,8** | NM_000593 | ILMN_1751079 |
| 1347 | **HIST1H4C** | **1,8** | NM_003542 | ILMN_1698511 |
| 1348 | **GCS1** | **1,8** | NM_006302 | ILMN_1727642 |
| 1349 | **STX16** | **1,8** | NM_003763 | ILMN_1741942 |
| 1350 | **LOC440093** | **1,8** | NM_001013699 | ILMN_1769705 |
| 1351 | **C12orf44** | **1,8** | NM_021934 | ILMN_1772527 |
| 1352 | **CCDC5** | **1,8** | NM_138443 | ILMN_1745946 |
| 1353 | **LAS1L** | **1,8** | NM_031206 | ILMN_1774890 |
| 1354 | **IQGAP3** | **1,8** | NM_178229 | ILMN_1813638 |
| 1355 | **DYRK3** | **1,8** | NM_003582 | ILMN_1758912 |
| 1356 | **NDUFB11** | **1,8** | NM_019056 | ILMN_1749709 |
| 1357 | **SEC61G** | **1,8** | NM_001012456 | ILMN_1787026 |
| 1358 | **RPL27** | **1,8** | NM_000988 | ILMN_1656807 |
| 1359 | **MRPS33** | **1,8** | NM_016071 | ILMN_1772722 |
| 1360 | **SDCCAG8** | **1,8** | NM_006642 | ILMN_1728802 |
| 1361 | **C7orf21** | **1,8** | NM_031434 | ILMN_1666050 |
| 1362 | **CHPF** | **1,8** | NM_024536 | ILMN_1731353 |
| 1363 | **GOT1** | **1,8** | NM_002079 | ILMN_1656145 |
| 1364 | **KCNJ4** | **1,8** | NM_152868 | ILMN_1697620 |
| 1365 | **VAC14** | **1,8** | NM_018052 | ILMN_1750792 |
| 1366 | **NAT9** | **1,8** | NM_015654 | ILMN_1776088 |
| 1367 | **ZFP36** | **1,8** | NM_003407 | ILMN_1720829 |
| 1368 | **CXorf44** | **1,8** | NM_138362 | ILMN_1705848 |
| 1369 | **TTLL4** | **1,8** | NM_014640 | ILMN_1746846 |
| 1370 | **TMEM68** | **1,8** | NM_152417 | ILMN_1702244 |
| 1371 | **SF3A2** | **1,8** | NM_007165 | ILMN_1754220 |
| 1372 | **ARG2** | **1,8** | NM_001172 | ILMN_1800898 |
| 1373 | **HSPC148** | **1,8** | NM_016403 | ILMN_1713482 |
| 1374 | **PTD008** | **1,8** | NM_016145 | ILMN_1757914 |
| 1375 | **FAM14A** | **1,8** | NM_032036 | ILMN_1740319 |
| 1376 | **CCT7** | **1,8** | NM_006429 | ILMN_1703718 |
| 1377 | **HLA-A** | **1,8** | NM_002116 | ILMN_1671054 |
| 1378 | **SLC35A2** | **1,8** | NM_001032289 | ILMN_1742731 |
| 1379 | **ACAA1** | **1,8** | NM_001607 | ILMN_1738921 |
| 1380 | **HSPA4L** | **1,8** | NM_014278 | ILMN_1732468 |
| 1381 | **SH3RF2** | **1,8** | NM_152550 | ILMN_1661137 |
| 1382 | **DNAJA5** | **1,8** | NM_194283 | ILMN_1714563 |
| 1383 | **C14orf150** | **1,8** | NM_080666 | ILMN_1659517 |
| 1384 | **CHKB** | **1,8** | NM_005198 | ILMN_1659054 |
| 1385 | **ID2** | **1,8** | NM_002166 | ILMN_1793990 |
| 1386 | **MTHFD1** | **1,8** | NM_005956 | ILMN_1785324 |
| 1387 | **RAG1AP1** | **1,8** | NM_018845 | ILMN_1776522 |
| 1388 | **NAPB** | **1,8** | NM_022080 | ILMN_1698741 |
| 1389 | **WDR5** | **1,8** | NM_017588 | ILMN_1737087 |
| 1390 | **CDCA4** | **1,8** | NM_017955 | ILMN_1753183 |
| 1391 | **GUSBL2** | **1,8** | NM_206910 | ILMN_1673660 |
| 1392 | **MGC22793** | **1,8** | NM_145030 | ILMN_1738514 |
| 1393 | **CCNB1IP1** | **1,8** | NM_021178 | ILMN_1752394 |
| 1394 | **UTP14A** | **1,8** | NM_006649 | ILMN_1688725 |
| 1395 | **HIST1H3J** | **1,8** | NM_003535 | ILMN_1707257 |
| 1396 | **C14orf124** | **1,8** | NM_020195 | ILMN_1683779 |
| 1397 | **CLIC1** | **1,8** | NM_001288 | ILMN_1716360 |
| 1398 | **TNKS** | **1,8** | NM_003747 | ILMN_1657891 |
| 1399 | **FAM62A** | **1,8** | NM_015292 | ILMN_1761159 |
| 1400 | **RNPS1** | **1,8** | NM_006711 | ILMN_1691843 |
| 1401 | **SCAMP3** | **1,8** | NM_052837 | ILMN_1793712 |
| 1402 | **NUP50** | **1,8** | NM_153645 | ILMN_1725612 |
| 1403 | **C22orf19** | **1,8** | NM_001002878 | ILMN_1773496 |
| 1404 | **NSMCE1** | **1,8** | NM_145080 | ILMN_1697962 |
| 1405 | **AGPAT3** | **1,8** | NM_020132 | ILMN_1678548 |
| 1406 | **PXMP2** | **1,8** | NM_018663 | ILMN_1799015 |
| 1407 | **NUDC** | **1,8** | NM_006600 | ILMN_1774079 |
| 1408 | **MRPL9** | **1,8** | NM_031420 | ILMN_1795346 |
| 1409 | **STYXL1** | **1,8** | NM_016086 | ILMN_1718822 |
| 1410 | **TUFM** | **1,8** | NM_003321 | ILMN_1738369 |
| 1411 | **DEDD** | **1,9** | NM_004216 | ILMN_1699711 |
| 1412 | **PUS1** | **1,9** | NM_025215 | ILMN_1672157 |
| 1413 | **ARL4C** | **1,9** | NM_005737 | ILMN_1768391 |
| 1414 | **PIN1** | **1,9** | NM_006221 | ILMN_1776375 |
| 1415 | **OSBPL6** | **1,9** | NM_145739 | ILMN_1756935 |
| 1416 | **CTSL** | **1,9** | NM_001912 | ILMN_1694757 |
| 1417 | **C16orf51** | **1,9** | NM_015421 | ILMN_1799804 |
| 1418 | **LENG8** | **1,9** | NM_052925 | ILMN_1654890 |
| 1419 | **LOC388272** | **1,9** | NM_001001436 | ILMN_1690911 |
| 1420 | **SLC25A15** | **1,9** | NM_014252 | ILMN_1667670 |
| 1421 | **TIMELESS** | **1,9** | NM_003920 | ILMN_1735093 |
| 1422 | **RAB4B** | **1,9** | NM_016154 | ILMN_1803136 |
| 1423 | **TOMM22** | **1,9** | NM_020243 | ILMN_1714623 |
| 1424 | **FBXO11** | **1,9** | NM_012167 | ILMN_1746302 |
| 1425 | **C9orf46** | **1,9** | NM_018465 | ILMN_1709043 |
| 1426 | **EIF2C3** | **1,9** | NM_024852 | ILMN_1809751 |
| 1427 | **DKFZp547C195** | **1,9** | NM_207343 | ILMN_1800420 |
| 1428 | **GTF2H4** | **1,9** | NM_001517 | ILMN_1712307 |
| 1429 | **MRPL24** | **1,9** | NM_024540 | ILMN_1695576 |
| 1430 | **ARIH1** | **1,9** | NM_005744 | ILMN_1694219 |
| 1431 | **NDUFS3** | **1,9** | NM_004551 | ILMN_1756355 |
| 1432 | **RAB38** | **1,9** | NM_022337 | ILMN_1687293 |
| 1433 | **SBSN** | **1,9** | NM_198538 | ILMN_1712759 |
| 1434 | **C9orf123** | **1,9** | NM_033428 | ILMN_1769409 |
| 1435 | **TRIM16** | **1,9** | NM_006470 | ILMN_1786331 |
| 1436 | **HIST2H4** | **1,9** | NM_003548 | ILMN_1797905 |
| 1437 | **G10** | **1,9** | NM_003910 | ILMN_1710697 |
| 1438 | **GJA5** | **1,9** | NM_005266 | ILMN_1703381 |
| 1439 | **UBE2G2** | **1,9** | NM_182688 | ILMN_1785179 |
| 1440 | **C14orf122** | **1,9** | NM_016049 | ILMN_1755677 |
| 1441 | **TDG** | **1,9** | NM_003211 | ILMN_1782331 |
| 1442 | **SEC63D1** | **1,9** | NM_198550 | ILMN_1796572 |
| 1443 | **ZC3H8** | **1,9** | NM_032494 | ILMN_1666530 |
| 1444 | **STMN3** | **1,9** | NM_015894 | ILMN_1693425 |
| 1445 | **BOP1** | **1,9** | NM_015201 | ILMN_1715583 |
| 1446 | **RNASEH2A** | **1,9** | NM_006397 | ILMN_1810901 |
| 1447 | **CNKSR3** | **1,9** | NM_173515 | ILMN_1748844 |
| 1448 | **UBIAD1** | **1,9** | NM_013319 | ILMN_1651872 |
| 1449 | **KIAA0907** | **1,9** | NM_014949 | ILMN_1670752 |
| 1450 | **IL27RA** | **1,9** | NM_004843 | ILMN_1688152 |
| 1451 | **CD163** | **1,9** | NM_203416 | ILMN_1722622 |
| 1452 | **B3GAT3** | **1,9** | NM_012200 | ILMN_1721432 |
| 1453 | **RSRC1** | **1,9** | NM_016625 | ILMN_1682494 |
| 1454 | **SEC22L3** | **1,9** | NM_004206 | ILMN_1664051 |
| 1455 | **STOML2** | **1,9** | NM_013442 | ILMN_1663002 |
| 1456 | **RPL10A** | **1,9** | NM_007104 | ILMN_1808041 |
| 1457 | **RAB5C** | **1,9** | NM_201434 | ILMN_1769665 |
| 1458 | **SIGIRR** | **1,9** | NM_021805 | ILMN_1807981 |
| 1459 | **DHRS10** | **1,9** | NM_016246 | ILMN_1809483 |
| 1460 | **DDA1** | **1,9** | NM_024050 | ILMN_1694530 |
| 1461 | **B4GALT3** | **1,9** | NM_003779 | ILMN_1692267 |
| 1462 | **C7orf11** | **1,9** | NM_138701 | ILMN_1745119 |
| 1463 | **MICAL-L1** | **1,9** | NM_033386 | ILMN_1671242 |
| 1464 | **NEBL** | **1,9** | NM_213569 | ILMN_1675927 |
| 1465 | **STRN4** | **1,9** | NM_013403 | ILMN_1696190 |
| 1466 | **TMEM1** | **1,9** | NM_001001723 | ILMN_1717973 |
| 1467 | **ILKAP** | **1,9** | NM_030768 | ILMN_1684647 |
| 1468 | **FLJ22318** | **1,9** | NM_022762 | ILMN_1737847 |
| 1469 | **ACTA2** | **1,9** | NM_001613 | ILMN_1671703 |
| 1470 | **S100A9** | **1,9** | NM_002965 | ILMN_1714991 |
| 1471 | **RGS12** | **1,9** | NM_198432 | ILMN_1686594 |
| 1472 | **DOM3Z** | **1,9** | NM_005510 | ILMN_1726990 |
| 1473 | **SEC61A2** | **1,9** | NM_018144 | ILMN_1779381 |
| 1474 | **COX7A2** | **1,9** | NM_001865 | ILMN_1701293 |
| 1475 | **IFP38** | **1,9** | NM_031943 | ILMN_1703005 |
| 1476 | **MGC9850** | **1,9** | NM_152705 | ILMN_1742427 |
| 1477 | **SITPEC** | **1,9** | NM_016581 | ILMN_1687769 |
| 1478 | **MRPL17** | **1,9** | NM_022061 | ILMN_1797933 |
| 1479 | **HINT3** | **1,9** | NM_138571 | ILMN_1779076 |
| 1480 | **RCE1** | **1,9** | NM_001032279 | ILMN_1685002 |
| 1481 | **BCCIP** | **1,9** | NM_016567 | ILMN_1786433 |
| 1482 | **LOC129531** | **1,9** | NM_138798 | ILMN_1677133 |
| 1483 | **C16orf52** | **1,9** | NM_173501 | ILMN_1717324 |
| 1484 | **TSPYL2** | **1,9** | NM_022117 | ILMN_1657554 |
| 1485 | **PX19** | **1,9** | NM_013237 | ILMN_1680022 |
| 1486 | **PNKP** | **1,9** | NM_007254 | ILMN_1694111 |
| 1487 | **RPIA** | **1,9** | NM_144563 | ILMN_1714809 |
| 1488 | **ATP5E** | **1,9** | NM_006886 | ILMN_1756674 |
| 1489 | **TBCA** | **1,9** | NM_004607 | ILMN_1726239 |
| 1490 | **PRAF1** | **1,9** | NM_022490 | ILMN_1678934 |
| 1491 | **TMC6** | **1,9** | NM_007267 | ILMN_1794677 |
| 1492 | **MGC4825** | **1,9** | NM_024122 | ILMN_1721623 |
| 1493 | **DGUOK** | **1,9** | NM_080916 | ILMN_1743432 |
| 1494 | **SLC6A15** | **1,9** | NM_182767 | ILMN_1659166 |
| 1495 | **COPE** | **1,9** | NM_199444 | ILMN_1738103 |
| 1496 | **ERCC2** | **1,9** | NM_000400 | ILMN_1815859 |
| 1497 | **PFN1** | **1,9** | NM_005022 | ILMN_1763012 |
| 1498 | **C21orf59** | **1,9** | NM_017835 | ILMN_1776147 |
| 1499 | **HNRPAB** | **1,9** | NM_031266 | ILMN_1696485 |
| 1500 | **FAH** | **1,9** | NM_000137 | ILMN_1781536 |
| 1501 | **CATSPER2** | **1,9** | NM_172097 | ILMN_1673077 |
| 1502 | **PHF5A** | **1,9** | NM_032758 | ILMN_1775901 |
| 1503 | **ZMAT5** | **1,9** | NM_019103 | ILMN_1745421 |
| 1504 | **C20orf27** | **1,9** | NM_017874 | ILMN_1697363 |
| 1505 | **SVH** | **1,9** | NM_031905 | ILMN_1689296 |
| 1506 | **COX7C** | **1,9** | NM_001867 | ILMN_1798189 |
| 1507 | **IPO7** | **1,9** | NM_006391 | ILMN_1682647 |
| 1508 | **C6orf134** | **1,9** | NM_001031722 | ILMN_1760037 |
| 1509 | **COG5** | **1,9** | NM_006348 | ILMN_1721535 |
| 1510 | **DYNLT1** | **1,9** | NM_006519 | ILMN_1678766 |
| 1511 | **CPA4** | **1,9** | NM_016352 | ILMN_1784294 |
| 1512 | **ARHGDIA** | **1,9** | NM_004309 | ILMN_1734742 |
| 1513 | **LDOC1** | **1,9** | NM_012317 | ILMN_1788250 |
| 1514 | **ACD** | **1,9** | NM_022914 | ILMN_1716480 |
| 1515 | **FLJ20489** | **1,9** | NM_017842 | ILMN_1758279 |
| 1516 | **SLC30A1** | **1,9** | NM_021194 | ILMN_1745021 |
| 1517 | **RPL35A** | **1,9** | NM_000996 | ILMN_1756360 |
| 1518 | **ZD52F10** | **1,9** | NM_033317 | ILMN_1778319 |
| 1519 | **ACN9** | **1,9** | NM_020186 | ILMN_1771348 |
| 1520 | **PREI3** | **1,9** | NM_015387 | ILMN_1664795 |
| 1521 | **ZNF330** | **1,9** | NM_014487 | ILMN_1710873 |
| 1522 | **FHL2** | **1,9** | NM_001450 | ILMN_1668411 |
| 1523 | **BSPRY** | **1,9** | NM_017688 | ILMN_1667077 |
| 1524 | **ACVR1B** | **1,9** | NM_020328 | ILMN_1763916 |
| 1525 | **NDUFA2** | **1,9** | NM_002488 | ILMN_1767123 |
| 1526 | **MAFG** | **1,9** | NM_002359 | ILMN_1692260 |
| 1527 | **HADH2** | **1,9** | NM_004493 | ILMN_1758275 |
| 1528 | **MRPS9** | **1,9** | NM_182640 | ILMN_1734973 |
| 1529 | **GAL** | **1,9** | NM_015973 | ILMN_1682015 |
| 1530 | **CYorf15B** | **1,9** | NM_032576 | ILMN_1756506 |
| 1531 | **RDBP** | **1,9** | NM_002904 | ILMN_1765532 |
| 1532 | **HSF2BP** | **1,9** | NM_007031 | ILMN_1706088 |
| 1533 | **LIMA1** | **2,0** | NM_016357 | ILMN_1704369 |
| 1534 | **PLEK2** | **2,0** | NM_016445 | ILMN_1811470 |
| 1535 | **MGC72104** | **2,0** | NM_207350 | ILMN_1688318 |
| 1536 | **MRPL22** | **2,0** | NM_014180 | ILMN_1663220 |
| 1537 | **RPL37** | **2,0** | NM_000997 | ILMN_1800796 |
| 1538 | **KLP1** | **2,0** | NM_020378 | ILMN_1784113 |
| 1539 | **UBE2C** | **2,0** | NM_181803 | ILMN_1714730 |
| 1540 | **HIST1H1C** | **2,0** | NM_005319 | ILMN_1757406 |
| 1541 | **NDUFS4** | **2,0** | NM_002495 | ILMN_1812312 |
| 1542 | **PLEKHB2** | **2,0** | NM_001031706 | ILMN_1698323 |
| 1543 | **NDUFA1** | **2,0** | NM_004541 | ILMN_1784286 |
| 1544 | **SCO2** | **2,0** | NM_005138 | ILMN_1811522 |
| 1545 | **MID2** | **2,0** | NM_052817 | ILMN_1760180 |
| 1546 | **DDX24** | **2,0** | NM_020414 | ILMN_1700628 |
| 1547 | **PIGF** | **2,0** | NM_173074 | ILMN_1808938 |
| 1548 | **ZFAND2B** | **2,0** | NM_138802 | ILMN_1653718 |
| 1549 | **SEC8L1** | **2,0** | NM_021807 | ILMN_1742827 |
| 1550 | **UBE2D4** | **2,0** | NM_015983 | ILMN_1707084 |
| 1551 | **TMEM126B** | **2,0** | NM_018480 | ILMN_1766851 |
| 1552 | **LOC349196** | **2,0** | NM_001025473 | ILMN_1764768 |
| 1553 | **TES** | **2,0** | NM_015641 | ILMN_1679546 |
| 1554 | **KIAA1467** | **2,0** | NM_020853 | ILMN_1668619 |
| 1555 | **NUP155** | **2,0** | NM_004298 | ILMN_1768293 |
| 1556 | **HEBP1** | **2,0** | NM_015987 | ILMN_1802557 |
| 1557 | **SNRPG** | **2,0** | NM_003096 | ILMN_1683562 |
| 1558 | **CLUAP1** | **2,0** | NM_015041 | ILMN_1774974 |
| 1559 | **LSM2** | **2,0** | NM_021177 | ILMN_1791544 |
| 1560 | **SPTLC1** | **2,0** | NM_178324 | ILMN_1665094 |
| 1561 | **SLC35F2** | **2,0** | NM_017515 | ILMN_1790577 |
| 1562 | **TAGLN** | **2,0** | NM_003186 | ILMN_1778668 |
| 1563 | **CHTF18** | **2,0** | NM_022092 | ILMN_1756705 |
| 1564 | **RABL3** | **2,0** | NM_173825 | ILMN_1662306 |
| 1565 | **GCN1L1** | **2,0** | NM_006836 | ILMN_1734312 |
| 1566 | **LONRF2** | **2,0** | NM_198461 | ILMN_1751016 |
| 1567 | **C3orf52** | **2,0** | NM_024616 | ILMN_1672969 |
| 1568 | **C4orf9** | **2,0** | NM_003703 | ILMN_1750052 |
| 1569 | **CALB2** | **2,0** | NM_001740 | ILMN_1748840 |
| 1570 | **VPREB3** | **2,0** | NM_013378 | ILMN_1700147 |
| 1571 | **MDS028** | **2,0** | NM_018463 | ILMN_1701244 |
| 1572 | **NCL** | **2,0** | NM_005381 | ILMN_1695422 |
| 1573 | **C21orf127** | **2,0** | NM_182749 | ILMN_1701077 |
| 1574 | **CLN6** | **2,0** | NM_017882 | ILMN_1776267 |
| 1575 | **SH3GLB1** | **2,0** | NM_016009 | ILMN_1764779 |
| 1576 | **FBXW9** | **2,0** | NM_032301 | ILMN_1750661 |
| 1577 | **NOC3L** | **2,0** | NM_022451 | ILMN_1771586 |
| 1578 | **KHDRBS3** | **2,0** | NM_006558 | ILMN_1691747 |
| 1579 | **LRRC8C** | **2,0** | NM_032270 | ILMN_1765855 |
| 1580 | **CUTA** | **2,0** | NM_001014433 | ILMN_1712390 |
| 1581 | **S100A6** | **2,0** | NM_014624 | ILMN_1713636 |
| 1582 | **MRPS7** | **2,0** | NM_015971 | ILMN_1813389 |
| 1583 | **PGAM5** | **2,0** | NM_138575 | ILMN_1748027 |
| 1584 | **MTX1** | **2,0** | NM_002455 | ILMN_1667222 |
| 1585 | **ASAH1** | **2,0** | NM_177924 | ILMN_1684054 |
| 1586 | **SRrp35** | **2,0** | NM_080743 | ILMN_1775345 |
| 1587 | **RHBDD1** | **2,0** | NM_032276 | ILMN_1681543 |
| 1588 | **FGF6** | **2,0** | NM_020996 | ILMN_1764605 |
| 1589 | **MED8** | **2,0** | NM_001001653 | ILMN_1736847 |
| 1590 | **IRAK1** | **2,0** | NM_001025242 | ILMN_1738397 |
| 1591 | **NRP2** | **2,0** | NM_201264 | ILMN_1682309 |
| 1592 | **ANGEL1** | **2,0** | NM_015305 | ILMN_1665871 |
| 1593 | **DNASE1** | **2,0** | NM_005223 | ILMN_1719616 |
| 1594 | **C1orf162** | **2,0** | NM_174896 | ILMN_1754894 |
| 1595 | **ASTN2** | **2,0** | NM_014010 | ILMN_1744118 |
| 1596 | **MICB** | **2,0** | NM_005931 | ILMN_1708006 |
| 1597 | **POMZP3** | **2,0** | NM_012230 | ILMN_1662970 |
| 1598 | **SHKBP1** | **2,0** | NM_138392 | ILMN_1765493 |
| 1599 | **COPS8** | **2,0** | NM_198189 | ILMN_1756065 |
| 1600 | **PABPN1** | **2,0** | NM_004643 | ILMN_1759154 |
| 1601 | **PPIL5** | **2,0** | NM_152329 | ILMN_1715616 |
| 1602 | **VTI1B** | **2,0** | NM_006370 | ILMN_1693136 |
| 1603 | **OGFRL1** | **2,0** | NM_024576 | ILMN_1715809 |
| 1604 | **EIF1B** | **2,0** | NM_005875 | ILMN_1679324 |
| 1605 | **C1orf33** | **2,0** | NM_016183 | ILMN_1689800 |
| 1606 | **MDP-1** | **2,0** | NM_138476 | ILMN_1662263 |
| 1607 | **LRP5L** | **2,0** | NM_182492 | ILMN_1718633 |
| 1608 | **MRPL28** | **2,0** | NM_006428 | ILMN_1694950 |
| 1609 | **FKBP10** | **2,0** | NM_021939 | ILMN_1659905 |
| 1610 | **APOA1BP** | **2,0** | NM_144772 | ILMN_1704368 |
| 1611 | **SF3B5** | **2,0** | NM_031287 | ILMN_1689389 |
| 1612 | **SRFBP1** | **2,0** | NM_152546 | ILMN_1801097 |
| 1613 | **OBFC1** | **2,0** | NM_024928 | ILMN_1789186 |
| 1614 | **FBXL15** | **2,0** | NM_024326 | ILMN_1719452 |
| 1615 | **SSBP2** | **2,0** | NM_012446 | ILMN_1711608 |
| 1616 | **MLLT3** | **2,0** | NM_004529 | ILMN_1812473 |
| 1617 | **MLF2** | **2,0** | NM_005439 | ILMN_1671885 |
| 1618 | **XRCC1** | **2,0** | NM_006297 | ILMN_1751743 |
| 1619 | **RASSF1** | **2,0** | NM_007182 | ILMN_1734205 |
| 1620 | **TMEM63B** | **2,0** | NM_018426 | ILMN_1728349 |
| 1621 | **BXDC1** | **2,0** | NM_032194 | ILMN_1664167 |
| 1622 | **ATF2** | **2,0** | NM_001880 | ILMN_1748271 |
| 1623 | **TBX1** | **2,0** | NM_080646 | ILMN_1680741 |
| 1624 | **NDUFAB1** | **2,0** | NM_005003 | ILMN_1755521 |
| 1625 | **GAB2** | **2,0** | NM_080491 | ILMN_1665964 |
| 1626 | **CSTF1** | **2,0** | NM_001033521 | ILMN_1758339 |
| 1627 | **MTRF1** | **2,0** | NM_004294 | ILMN_1654918 |
| 1628 | **PHF6** | **2,0** | NM_032335 | ILMN_1766110 |
| 1629 | **BRI3BP** | **2,0** | NM_080626 | ILMN_1800619 |
| 1630 | **KCNK12** | **2,0** | NM_022055 | ILMN_1711988 |
| 1631 | **SNTA1** | **2,0** | NM_003098 | ILMN_1753241 |
| 1632 | **SLC7A6OS** | **2,0** | NM_032178 | ILMN_1690607 |
| 1633 | **TATDN1** | **2,0** | NM_032026 | ILMN_1701058 |
| 1634 | **MSL3L1** | **2,0** | NM_078628 | ILMN_1670723 |
| 1635 | **NOB1P** | **2,0** | NM_014062 | ILMN_1689110 |
| 1636 | **CKMT1B** | **2,0** | NM_020990 | ILMN_1763491 |
| 1637 | **KLC2** | **2,0** | NM_022822 | ILMN_1779064 |
| 1638 | **RPL37A** | **2,0** | NM_000998 | ILMN_1711222 |
| 1639 | **NUCKS1** | **2,0** | NM_022731 | ILMN_1680692 |
| 1640 | **PAK1IP1** | **2,0** | NM_017906 | ILMN_1726064 |
| 1641 | **HARS2** | **2,0** | NM_080820 | ILMN_1774432 |
| 1642 | **NBR1** | **2,0** | NM_031862 | ILMN_1799628 |
| 1643 | **SERPINE2** | **2,0** | NM_006216 | ILMN_1655595 |
| 1644 | **C20orf7** | **2,0** | NM_024120 | ILMN_1813344 |
| 1645 | **C11orf51** | **2,0** | NM_014042 | ILMN_1779596 |
| 1646 | **KLF10** | **2,1** | NM_005655 | ILMN_1659122 |
| 1647 | **PPIE** | **2,1** | NM_006112 | ILMN_1680341 |
| 1648 | **HDLBP** | **2,1** | NM_005336 | ILMN_1678252 |
| 1649 | **JAGN1** | **2,1** | NM_032492 | ILMN_1748399 |
| 1650 | **PPM1G** | **2,1** | NM_002707 | ILMN_1771637 |
| 1651 | **KIAA1274** | **2,1** | NM_014431 | ILMN_1796751 |
| 1652 | **EBPL** | **2,1** | NM_032565 | ILMN_1805922 |
| 1653 | **SLC30A3** | **2,1** | NM_003459 | ILMN_1805807 |
| 1654 | **RSAD1** | **2,1** | NM_018346 | ILMN_1809963 |
| 1655 | **MAP2** | **2,1** | NM_031845 | ILMN_1764201 |
| 1656 | **STAMBPL1** | **2,1** | NM_020799 | ILMN_1682799 |
| 1657 | **SEC11L3** | **2,1** | NM_033280 | ILMN_1701681 |
| 1658 | **EXOSC3** | **2,1** | NM_016042 | ILMN_1734194 |
| 1659 | **C10orf35** | **2,1** | NM_145306 | ILMN_1662470 |
| 1660 | **HSPC138** | **2,1** | NM_016401 | ILMN_1781419 |
| 1661 | **SLC1A5** | **2,1** | NM_005628 | ILMN_1707720 |
| 1662 | **PTRH2** | **2,1** | NM_001015509 | ILMN_1683052 |
| 1663 | **LOC130355** | **2,1** | NM_001017927 | ILMN_1726729 |
| 1664 | **ITGA5** | **2,1** | NM_002205 | ILMN_1792679 |
| 1665 | **KIAA0020** | **2,1** | NM_014878 | ILMN_1665483 |
| 1666 | **C13orf12** | **2,1** | NM_015932 | ILMN_1693287 |
| 1667 | **PRDX5** | **2,1** | NM_012094 | ILMN_1815024 |
| 1668 | **EMD** | **2,1** | NM_000117 | ILMN_1801421 |
| 1669 | **MLC1SA** | **2,1** | NM_002475 | ILMN_1713450 |
| 1670 | **CCNH** | **2,1** | NM_001239 | ILMN_1742250 |
| 1671 | **PFKM** | **2,1** | NM_000289 | ILMN_1708180 |
| 1672 | **WHSC1L1** | **2,1** | NM_023034 | ILMN_1807379 |
| 1673 | **BTG3** | **2,1** | NM_006806 | ILMN_1707339 |
| 1674 | **AZIN1** | **2,1** | NM_015878 | ILMN_1704550 |
| 1675 | **MGC39633** | **2,1** | NM_152549 | ILMN_1761101 |
| 1676 | **YRDC** | **2,1** | NM_024640 | ILMN_1736008 |
| 1677 | **SLC4A2** | **2,1** | NM_003040 | ILMN_1799652 |
| 1678 | **NY-REN-7** | **2,1** | NM_173663 | ILMN_1745669 |
| 1679 | **TIPRL** | **2,1** | NM_001031800 | ILMN_1781457 |
| 1680 | **POLR2E** | **2,1** | NM_002695 | ILMN_1746679 |
| 1681 | **TBXAS1** | **2,1** | NM_001061 | ILMN_1713036 |
| 1682 | **VASP** | **2,1** | NM_003370 | ILMN_1702863 |
| 1683 | **GCNT2** | **2,1** | NM_145649 | ILMN_1655651 |
| 1684 | **POP7** | **2,1** | NM_005837 | ILMN_1680091 |
| 1685 | **C10orf70** | **2,1** | NM_018464 | ILMN_1761781 |
| 1686 | **GCDH** | **2,1** | NM_000159 | ILMN_1753796 |
| 1687 | **FN5** | **2,1** | NM_020179 | ILMN_1798270 |
| 1688 | **VAT1** | **2,1** | NM_006373 | ILMN_1700690 |
| 1689 | **ERGIC1** | **2,1** | NM_001031711 | ILMN_1778377 |
| 1690 | **FOXD4L4** | **2,1** | NM_199244 | ILMN_1727674 |
| 1691 | **NEU1** | **2,1** | NM_000434 | ILMN_1763144 |
| 1692 | **ADAM9** | **2,1** | NM_003816 | ILMN_1727524 |
| 1693 | **MGC2408** | **2,1** | NM_032331 | ILMN_1762883 |
| 1694 | **ABHD14A** | **2,1** | NM_015407 | ILMN_1794213 |
| 1695 | **DNCL2A** | **2,1** | NM_177953 | ILMN_1766762 |
| 1696 | **PDRG1** | **2,1** | NM_030815 | ILMN_1731720 |
| 1697 | **BSCL2** | **2,1** | NM_032667 | ILMN_1774596 |
| 1698 | **THOC4** | **2,1** | NM_005782 | ILMN_1698987 |
| 1699 | **FIP1L1** | **2,1** | NM_030917 | ILMN_1768743 |
| 1700 | **POLE4** | **2,1** | NM_019896 | ILMN_1660063 |
| 1701 | **UHRF2** | **2,1** | NM_152306 | ILMN_1691444 |
| 1702 | **C14orf138** | **2,1** | NM_024558 | ILMN_1781102 |
| 1703 | **GUK1** | **2,1** | NM_000858 | ILMN_1758398 |
| 1704 | **ARHGAP11A** | **2,1** | NM_199357 | ILMN_1738830 |
| 1705 | **IDH3G** | **2,1** | NM_004135 | ILMN_1802706 |
| 1706 | **UBQLN1** | **2,1** | NM_053067 | ILMN_1798380 |
| 1707 | **SSR4** | **2,1** | NM_006280 | ILMN_1680403 |
| 1708 | **PURG** | **2,1** | NM_001015508 | ILMN_1709815 |
| 1709 | **SLC25A35** | **2,1** | NM_201520 | ILMN_1724612 |
| 1710 | **ETS1** | **2,1** | NM_005238 | ILMN_1687538 |
| 1711 | **TFIP11** | **2,1** | NM_001008697 | ILMN_1695000 |
| 1712 | **NAPG** | **2,1** | NM_003826 | ILMN_1788268 |
| 1713 | **UNQ3045** | **2,1** | NM_207409 | ILMN_1803151 |
| 1714 | **PPP3CC** | **2,1** | NM_005605 | ILMN_1690546 |
| 1715 | **CCDC25** | **2,1** | NM_001031708 | ILMN_1793146 |
| 1716 | **TAF1A** | **2,1** | NM_005681 | ILMN_1689266 |
| 1717 | **DDX11** | **2,1** | NM_030655 | ILMN_1679413 |
| 1718 | **AVEN** | **2,1** | NM_020371 | ILMN_1710216 |
| 1719 | **FLJ23436** | **2,1** | NM_024671 | ILMN_1791820 |
| 1720 | **HIGD2A** | **2,1** | NM_138820 | ILMN_1774334 |
| 1721 | **EPB41L2** | **2,1** | NM_001431 | ILMN_1805448 |
| 1722 | **DIP2A** | **2,1** | NM_206890 | ILMN_1695074 |
| 1723 | **MLLT10** | **2,1** | NM_004641 | ILMN_1770119 |
| 1724 | **BEX1** | **2,1** | NM_018476 | ILMN_1702637 |
| 1725 | **ZDHHC24** | **2,1** | NM_207340 | ILMN_1687626 |
| 1726 | **COX11** | **2,1** | NM_004375 | ILMN_1666280 |
| 1727 | **BRI3** | **2,1** | NM_015379 | ILMN_1781580 |
| 1728 | **TPRKB** | **2,1** | NM_016058 | ILMN_1690307 |
| 1729 | **HEY1** | **2,1** | NM_012258 | ILMN_1788203 |
| 1730 | **XAB2** | **2,1** | NM_020196 | ILMN_1771057 |
| 1731 | **DUS1L** | **2,1** | NM_022156 | ILMN_1653342 |
| 1732 | **TRIAP1** | **2,1** | NM_016399 | ILMN_1774083 |
| 1733 | **CHMP4A** | **2,1** | NM_014169 | ILMN_1702828 |
| 1734 | **XKR6** | **2,1** | NM_173683 | ILMN_1731175 |
| 1735 | **C14orf130** | **2,1** | NM_175748 | ILMN_1758293 |
| 1736 | **MAP1B** | **2,1** | NM_032010 | ILMN_1751136 |
| 1737 | **C14orf48** | **2,1** | NM_152777 | ILMN_1662800 |
| 1738 | **HABP4** | **2,1** | NM_014282 | ILMN_1792384 |
| 1739 | **MATR3** | **2,1** | NM_199189 | ILMN_1661673 |
| 1740 | **FBXO44** | **2,1** | NM_183412 | ILMN_1732182 |
| 1741 | **CNTNAP1** | **2,1** | NM_003632 | ILMN_1692398 |
| 1742 | **C16orf33** | **2,1** | NM_024571 | ILMN_1801118 |
| 1743 | **SIRT7** | **2,1** | NM_016538 | ILMN_1800418 |
| 1744 | **COX8A** | **2,1** | NM_004074 | ILMN_1809495 |
| 1745 | **RABEPK** | **2,1** | NM_005833 | ILMN_1741957 |
| 1746 | **COX6C** | **2,1** | NM_004374 | ILMN_1654151 |
| 1747 | **SORD** | **2,1** | NM_003104 | ILMN_1734559 |
| 1748 | **ZFAND2A** | **2,1** | NM_182491 | ILMN_1694671 |
| 1749 | **EIF5B** | **2,1** | NM_015904 | ILMN_1756767 |
| 1750 | **GTF2IRD2** | **2,1** | NM_173537 | ILMN_1680501 |
| 1751 | **FHOD1** | **2,1** | NM_013241 | ILMN_1651776 |
| 1752 | **C6orf106** | **2,1** | NM_024294 | ILMN_1757723 |
| 1753 | **HIST1H3F** | **2,1** | NM_021018 | ILMN_1788489 |
| 1754 | **EAF1** | **2,1** | NM_033083 | ILMN_1685012 |
| 1755 | **ABHD14B** | **2,2** | NM_032750 | ILMN_1801260 |
| 1756 | **CSNK1G1** | **2,2** | NM_001011664 | ILMN_1723398 |
| 1757 | **COMMD6** | **2,2** | NM_203495 | ILMN_1777378 |
| 1758 | **IMMP2L** | **2,2** | NM_032549 | ILMN_1809292 |
| 1759 | **PVR** | **2,2** | NM_006505 | ILMN_1677305 |
| 1760 | **ZNF337** | **2,2** | NM_015655 | ILMN_1663160 |
| 1761 | **FLJ31438** | **2,2** | NM_152385 | ILMN_1805148 |
| 1762 | **PHC1** | **2,2** | NM_004426 | ILMN_1796710 |
| 1763 | **ZNF133** | **2,2** | NM_003434 | ILMN_1759375 |
| 1764 | **SETD4** | **2,2** | NM_001007260 | ILMN_1751075 |
| 1765 | **WDR58** | **2,2** | NM_024339 | ILMN_1679880 |
| 1766 | **FAM103A1** | **2,2** | NM_031452 | ILMN_1763365 |
| 1767 | **S100A16** | **2,2** | NM_080388 | ILMN_1728049 |
| 1768 | **RPL17** | **2,2** | NM_000985 | ILMN_1701689 |
| 1769 | **C12orf62** | **2,2** | NM_032901 | ILMN_1804656 |
| 1770 | **MPHOSPH6** | **2,2** | NM_005792 | ILMN_1746682 |
| 1771 | **BCS1L** | **2,2** | NM_004328 | ILMN_1738529 |
| 1772 | **CCK** | **2,2** | NM_000729 | ILMN_1712943 |
| 1773 | **RASSF3** | **2,2** | NM_178169 | ILMN_1761026 |
| 1774 | **CMTM7** | **2,2** | NM_138410 | ILMN_1740487 |
| 1775 | **OBFC2B** | **2,2** | NM_024068 | ILMN_1704672 |
| 1776 | **ORC2L** | **2,2** | NM_006190 | ILMN_1701440 |
| 1777 | **SLC39A7** | **2,2** | NM_006979 | ILMN_1770855 |
| 1778 | **BRP44L** | **2,2** | NM_016098 | ILMN_1666967 |
| 1779 | **SUPT3H** | **2,2** | NM_003599 | ILMN_1813277 |
| 1780 | **FES** | **2,2** | NM_002005 | ILMN_1693650 |
| 1781 | **CGI-69** | **2,2** | NM_016016 | ILMN_1721723 |
| 1782 | **COX6A1** | **2,2** | NM_004373 | ILMN_1783636 |
| 1783 | **CAPZB** | **2,2** | NM_004930 | ILMN_1798980 |
| 1784 | **DLNB14** | **2,2** | NM_198489 | ILMN_1709227 |
| 1785 | **CD79B** | **2,2** | NM_021602 | ILMN_1789636 |
| 1786 | **ADRA1A** | **2,2** | NM_033302 | ILMN_1799019 |
| 1787 | **RAB24** | **2,2** | NM_130781 | ILMN_1677843 |
| 1788 | **SAC** | **2,2** | NM_018417 | ILMN_1808797 |
| 1789 | **FOSL2** | **2,2** | NM_005253 | ILMN_1725175 |
| 1790 | **CECR1** | **2,2** | NM_177405 | ILMN_1666174 |
| 1791 | **C1orf144** | **2,2** | NM_015609 | ILMN_1707503 |
| 1792 | **PQBP1** | **2,2** | NM_001032385 | ILMN_1711596 |
| 1793 | **DXYS155E** | **2,2** | NM_005088 | ILMN_1807737 |
| 1794 | **FLJ20323** | **2,2** | NM_019005 | ILMN_1680644 |
| 1795 | **SENP5** | **2,2** | NM_152699 | ILMN_1675501 |
| 1796 | **COPS6** | **2,2** | NM_006833 | ILMN_1764431 |
| 1797 | **GRINA** | **2,2** | NM_000837 | ILMN_1796490 |
| 1798 | **ZNF581** | **2,2** | NM_016535 | ILMN_1679093 |
| 1799 | **DSCR5** | **2,2** | NM_153682 | ILMN_1774949 |
| 1800 | **SETD3** | **2,2** | NM_199123 | ILMN_1756328 |
| 1801 | **YWHAE** | **2,2** | NM_006761 | ILMN_1807535 |
| 1802 | **BCAS4** | **2,2** | NM_017843 | ILMN_1808059 |
| 1803 | **SNF8** | **2,2** | NM_007241 | ILMN_1766171 |
| 1804 | **CMIP** | **2,2** | NM_198390 | ILMN_1767182 |
| 1805 | **ZNF638** | **2,2** | NM_001014972 | ILMN_1791379 |
| 1806 | **NSUN5** | **2,2** | NM_148956 | ILMN_1751958 |
| 1807 | **ZBTB7A** | **2,2** | NM_015898 | ILMN_1656118 |
| 1808 | **RPL26** | **2,2** | NM_000987 | ILMN_1731546 |
| 1809 | **PLK3** | **2,2** | NM_004073 | ILMN_1679979 |
| 1810 | **HIST1H2AM** | **2,2** | NM_003514 | ILMN_1756022 |
| 1811 | **BBC3** | **2,2** | NM_014417 | ILMN_1729645 |
| 1812 | **LMO6** | **2,2** | NM_006150 | ILMN_1656942 |
| 1813 | **COQ4** | **2,2** | NM_016035 | ILMN_1745677 |
| 1814 | **CKMT1A** | **2,2** | NM_001015001 | ILMN_1732066 |
| 1815 | **NELF** | **2,2** | NM_015537 | ILMN_1665095 |
| 1816 | **HINT2** | **2,2** | NM_032593 | ILMN_1697820 |
| 1817 | **SYNGR4** | **2,2** | NM_012451 | ILMN_1726924 |
| 1818 | **HRAS** | **2,2** | NM_176795 | ILMN_1773751 |
| 1819 | **SOX30** | **2,2** | NM_007017 | ILMN_1702430 |
| 1820 | **RHOG** | **2,2** | NM_001665 | ILMN_1739792 |
| 1821 | **ASB4** | **2,2** | NM_145872 | ILMN_1813668 |
| 1822 | **DHDH** | **2,2** | NM_014475 | ILMN_1689200 |
| 1823 | **NDUFB8** | **2,2** | NM_005004 | ILMN_1661170 |
| 1824 | **CAPN6** | **2,2** | NM_014289 | ILMN_1782654 |
| 1825 | **NUDT14** | **2,2** | NM_177533 | ILMN_1669788 |
| 1826 | **CCDC34** | **2,2** | NM_030771 | ILMN_1657547 |
| 1827 | **ZNF524** | **2,2** | NM_153219 | ILMN_1810147 |
| 1828 | **SMUG1** | **2,2** | NM_014311 | ILMN_1804642 |
| 1829 | **SCAMP2** | **2,2** | NM_005697 | ILMN_1654893 |
| 1830 | **POLR2J3** | **2,2** | NM_001015884 | ILMN_1677138 |
| 1831 | **TFE3** | **2,2** | NM_006521 | ILMN_1764826 |
| 1832 | **ATP5D** | **2,2** | NM_001687 | ILMN_1679178 |
| 1833 | **NOLA1** | **2,2** | NM_032993 | ILMN_1681925 |
| 1834 | **HNRPUL1** | **2,2** | NM_144732 | ILMN_1719728 |
| 1835 | **COX10** | **2,2** | NM_001303 | ILMN_1699200 |
| 1836 | **FKBP4** | **2,2** | NM_002014 | ILMN_1782045 |
| 1837 | **LOC51255** | **2,2** | NM_016494 | ILMN_1655340 |
| 1838 | **RAD52** | **2,2** | NM_134422 | ILMN_1781345 |
| 1839 | **C1orf61** | **2,2** | NM_006365 | ILMN_1759652 |
| 1840 | **MRPS34** | **2,2** | NM_023936 | ILMN_1780127 |
| 1841 | **IPO4** | **2,2** | NM_024658 | ILMN_1772988 |
| 1842 | **BTF3** | **2,2** | NM_001207 | ILMN_1676221 |
| 1843 | **ACIN1** | **2,2** | NM_014977 | ILMN_1699636 |
| 1844 | **FIS1** | **2,2** | NM_016068 | ILMN_1658351 |
| 1845 | **PDCD2** | **2,2** | NM_144781 | ILMN_1758915 |
| 1846 | **GSK3B** | **2,2** | NM_002093 | ILMN_1779376 |
| 1847 | **TIMM9** | **2,2** | NM_012460 | ILMN_1653709 |
| 1848 | **CNIH4** | **2,2** | NM_014184 | ILMN_1714759 |
| 1849 | **UBE2S** | **2,2** | NM_014501 | ILMN_1808591 |
| 1850 | **MOS** | **2,2** | NM_005372 | ILMN_1760479 |
| 1851 | **MYCL1** | **2,2** | NM_005376 | ILMN_1764443 |
| 1852 | **MRPL34** | **2,2** | NM_023937 | ILMN_1783681 |
| 1853 | **ABCC3** | **2,2** | NM_020038 | ILMN_1717466 |
| 1854 | **GPR103** | **2,2** | NM_198179 | ILMN_1692456 |
| 1855 | **RPL36A** | **2,2** | NM_021029 | ILMN_1781137 |
| 1856 | **SERPINE1** | **2,2** | NM_000602 | ILMN_1744381 |
| 1857 | **SLC26A2** | **2,2** | NM_000112 | ILMN_1801491 |
| 1858 | **NDUFS5** | **2,2** | NM_004552 | ILMN_1776104 |
| 1859 | **RANBP1** | **2,2** | NM_002882 | ILMN_1721457 |
| 1860 | **PHF17** | **2,2** | NM_199320 | ILMN_1655194 |
| 1861 | **FAU** | **2,2** | NM_001997 | ILMN_1664614 |
| 1862 | **FSD1NL** | **2,2** | NM_031919 | ILMN_1679678 |
| 1863 | **FLJ37478** | **2,2** | NM_178557 | ILMN_1672437 |
| 1864 | **VARS** | **2,2** | NM_006295 | ILMN_1696601 |
| 1865 | **NRIP3** | **2,2** | NM_020645 | ILMN_1759563 |
| 1866 | **C16orf24** | **2,2** | NM_023933 | ILMN_1773780 |
| 1867 | **PSCD2** | **2,2** | NM_017457 | ILMN_1721241 |
| 1868 | **POFUT1** | **2,2** | NM_172236 | ILMN_1802242 |
| 1869 | **VTI1A** | **2,2** | NM_145206 | ILMN_1810848 |
| 1870 | **KIAA0690** | **2,2** | NM_015179 | ILMN_1767253 |
| 1871 | **C21orf6** | **2,2** | NM_016940 | ILMN_1698233 |
| 1872 | **JTB** | **2,3** | NM_006694 | ILMN_1746990 |
| 1873 | **HIST1H1E** | **2,3** | NM_005321 | ILMN_1746435 |
| 1874 | **HNRPL** | **2,3** | NM_001533 | ILMN_1679378 |
| 1875 | **KRT18** | **2,3** | NM_199187 | ILMN_1730848 |
| 1876 | **C2orf26** | **2,3** | NM_023016 | ILMN_1724040 |
| 1877 | **ZMAT4** | **2,3** | NM_024645 | ILMN_1720732 |
| 1878 | **FLI1** | **2,3** | NM_002017 | ILMN_1665738 |
| 1879 | **CT45-3** | **2,3** | NM_001017435 | ILMN_1757689 |
| 1880 | **ADMR** | **2,3** | NM_007264 | ILMN_1696810 |
| 1881 | **PRPF4B** | **2,3** | NM_176800 | ILMN_1760281 |
| 1882 | **RPUSD3** | **2,3** | NM_173659 | ILMN_1795383 |
| 1883 | **GDI1** | **2,3** | NM_001493 | ILMN_1734153 |
| 1884 | **SUPT6H** | **2,3** | NM_003170 | ILMN_1747335 |
| 1885 | **STMN1** | **2,3** | NM_005563 | ILMN_1745593 |
| 1886 | **MRPS15** | **2,3** | NM_031280 | ILMN_1680703 |
| 1887 | **UXT** | **2,3** | NM_004182 | ILMN_1693687 |
| 1888 | **TSPYL6** | **2,3** | NM_001003937 | ILMN_1701264 |
| 1889 | **DGKD** | **2,3** | NM_152879 | ILMN_1735301 |
| 1890 | **GP1BA** | **2,3** | NM_000173 | ILMN_1753575 |
| 1891 | **CCDC19** | **2,3** | NM_012337 | ILMN_1807976 |
| 1892 | **CKLF** | **2,3** | NM_016326 | ILMN_1712389 |
| 1893 | **PKN1** | **2,3** | NM_213560 | ILMN_1776917 |
| 1894 | **FAM86A** | **2,3** | NM_201400 | ILMN_1780298 |
| 1895 | **LOC126208** | **2,3** | NM_001002836 | ILMN_1791388 |
| 1896 | **C14orf102** | **2,3** | NM_017970 | ILMN_1740165 |
| 1897 | **NDUFB2** | **2,3** | NM_004546 | ILMN_1714495 |
| 1898 | **VRK2** | **2,3** | NM_006296 | ILMN_1750088 |
| 1899 | **PCDH10** | **2,3** | NM_020815 | ILMN_1786745 |
| 1900 | **ZNF180** | **2,3** | NM_013256 | ILMN_1696319 |
| 1901 | **PNKD** | **2,3** | NM_015488 | ILMN_1711810 |
| 1902 | **NFKBIA** | **2,3** | NM_020529 | ILMN_1773154 |
| 1903 | **RALY** | **2,3** | NM_016732 | ILMN_1690610 |
| 1904 | **SRI** | **2,3** | NM_003130 | ILMN_1682054 |
| 1905 | **C21orf124** | **2,3** | NM_032920 | ILMN_1764503 |
| 1906 | **ZC3HAV1** | **2,3** | NM_024625 | ILMN_1729973 |
| 1907 | **ODZ1** | **2,3** | NM_014253 | ILMN_1803375 |
| 1908 | **BAZ1A** | **2,3** | NM_013448 | ILMN_1658327 |
| 1909 | **PEX16** | **2,3** | NM_004813 | ILMN_1745655 |
| 1910 | **ELOVL1** | **2,3** | NM_022821 | ILMN_1798123 |
| 1911 | **FLJ43093** | **2,3** | NM_207498 | ILMN_1746235 |
| 1912 | **SPAG7** | **2,3** | NM_004890 | ILMN_1684446 |
| 1913 | **GRINL1A** | **2,3** | NM_001018102 | ILMN_1809511 |
| 1914 | **CLPP** | **2,3** | NM_006012 | ILMN_1725705 |
| 1915 | **FANCD2** | **2,3** | NM_001018115 | ILMN_1810703 |
| 1916 | **THOC2** | **2,3** | NM_020449 | ILMN_1696883 |
| 1917 | **PHF22** | **2,3** | NM_020395 | ILMN_1725169 |
| 1918 | **FLJ90652** | **2,3** | NM_173618 | ILMN_1724406 |
| 1919 | **C9orf37** | **2,3** | NM_032937 | ILMN_1799320 |
| 1920 | **RPS18** | **2,3** | NM_022551 | ILMN_1753534 |
| 1921 | **MRPS12** | **2,3** | NM_033363 | ILMN_1714515 |
| 1922 | **TOMM7** | **2,3** | NM_019059 | ILMN_1674069 |
| 1923 | **FLJ35767** | **2,3** | NM_207459 | ILMN_1775067 |
| 1924 | **NUDT2** | **2,3** | NM_147173 | ILMN_1778347 |
| 1925 | **ALG2** | **2,3** | NM_197973 | ILMN_1722688 |
| 1926 | **APBA3** | **2,3** | NM_004886 | ILMN_1686610 |
| 1927 | **CHCHD1** | **2,3** | NM_203298 | ILMN_1672149 |
| 1928 | **DGKB** | **2,3** | NM_145695 | ILMN_1699693 |
| 1929 | **RFX3** | **2,3** | NM_002919 | ILMN_1685591 |
| 1930 | **MAGED2** | **2,3** | NM_201222 | ILMN_1661417 |
| 1931 | **JOSD2** | **2,3** | NM_138334 | ILMN_1720964 |
| 1932 | **LOC201175** | **2,3** | NM_174919 | ILMN_1656361 |
| 1933 | **EIF5A** | **2,3** | NM_001970 | ILMN_1794522 |
| 1934 | **LONRF3** | **2,3** | NM_024778 | ILMN_1720452 |
| 1935 | **SCAND1** | **2,3** | NM_033630 | ILMN_1794230 |
| 1936 | **ZNF2** | **2,3** | NM_021088 | ILMN_1770650 |
| 1937 | **MGC23909** | **2,3** | NM_174909 | ILMN_1742813 |
| 1938 | **MCL1** | **2,3** | NM_021960 | ILMN_1683375 |
| 1939 | **RABGGTB** | **2,3** | NM_004582 | ILMN_1790354 |
| 1940 | **PAFAH1B3** | **2,3** | NM_002573 | ILMN_1762654 |
| 1941 | **MGC15407** | **2,3** | NM_080667 | ILMN_1792456 |
| 1942 | **PRR6** | **2,3** | NM_181716 | ILMN_1729142 |
| 1943 | **MGC19604** | **2,3** | NM_001031734 | ILMN_1744628 |
| 1944 | **NEIL2** | **2,3** | NM_145043 | ILMN_1715680 |
| 1945 | **C1orf128** | **2,3** | NM_020362 | ILMN_1784207 |
| 1946 | **ZNF557** | **2,3** | NM_024341 | ILMN_1719163 |
| 1947 | **CBX1** | **2,3** | NM_006807 | ILMN_1770244 |
| 1948 | **PDE6C** | **2,3** | NM_006204 | ILMN_1730851 |
| 1949 | **TAAR2** | **2,3** | NM_001033080 | ILMN_1761170 |
| 1950 | **TRIM43** | **2,3** | NM_138800 | ILMN_1784230 |
| 1951 | **EXOSC1** | **2,3** | NM_016046 | ILMN_1768150 |
| 1952 | **PIP5K2A** | **2,3** | NM_005028 | ILMN_1751981 |
| 1953 | **HSPC152** | **2,3** | NM_016404 | ILMN_1690802 |
| 1954 | **PRSS36** | **2,3** | NM_173502 | ILMN_1758240 |
| 1955 | **NOLA3** | **2,3** | NM_018648 | ILMN_1815479 |
| 1956 | **C1orf57** | **2,3** | NM_032324 | ILMN_1657446 |
| 1957 | **C8orf48** | **2,3** | NM_001007090 | ILMN_1784408 |
| 1958 | **POLA** | **2,3** | NM_016937 | ILMN_1674315 |
| 1959 | **KIAA0409** | **2,3** | NM_015324 | ILMN_1765272 |
| 1960 | **RIP** | **2,3** | NM_001033002 | ILMN_1770339 |
| 1961 | **SNX8** | **2,3** | NM_013321 | ILMN_1804051 |
| 1962 | **TULP3** | **2,3** | NM_003324 | ILMN_1719199 |
| 1963 | **CHCHD3** | **2,3** | NM_017812 | ILMN_1673026 |
| 1964 | **IL11** | **2,3** | NM_000641 | ILMN_1788107 |
| 1965 | **RPS19BP1** | **2,3** | NM_194326 | ILMN_1702059 |
| 1966 | **C14orf168** | **2,3** | NM_031427 | ILMN_1730464 |
| 1967 | **ROM1** | **2,3** | NM_000327 | ILMN_1723743 |
| 1968 | **INHBE** | **2,4** | NM_031479 | ILMN_1811767 |
| 1969 | **TEAD4** | **2,4** | NM_201441 | ILMN_1705301 |
| 1970 | **KIAA0329** | **2,4** | NM_014844 | ILMN_1777790 |
| 1971 | **PLP1** | **2,4** | NM_000533 | ILMN_1790106 |
| 1972 | **C1orf53** | **2,4** | NM_001024594 | ILMN_1661595 |
| 1973 | **CDK5RAP2** | **2,4** | NM_001011649 | ILMN_1655990 |
| 1974 | **MRPL2** | **2,4** | NM_015950 | ILMN_1763264 |
| 1975 | **PEBP1** | **2,4** | NM_002567 | ILMN_1688089 |
| 1976 | **FAM83F** | **2,4** | NM_138435 | ILMN_1683231 |
| 1977 | **TMCC1** | **2,4** | NM_015008 | ILMN_1750539 |
| 1978 | **LIF** | **2,4** | NM_002309 | ILMN_1780599 |
| 1979 | **IRX1** | **2,4** | NM_024337 | ILMN_1735353 |
| 1980 | **RNF138** | **2,4** | NM_016271 | ILMN_1742806 |
| 1981 | **CRY2** | **2,4** | NM_021117 | ILMN_1796180 |
| 1982 | **SERF1B** | **2,4** | NM_022978 | ILMN_1655011 |
| 1983 | **FAF1** | **2,4** | NM_131917 | ILMN_1667016 |
| 1984 | **GAMT** | **2,4** | NM_000156 | ILMN_1794595 |
| 1985 | **RAB32** | **2,4** | NM_006834 | ILMN_1768771 |
| 1986 | **PTGES2** | **2,4** | NM_198938 | ILMN_1763824 |
| 1987 | **PLEC1** | **2,4** | NM_201384 | ILMN_1744268 |
| 1988 | **ZNF297** | **2,4** | NM_005453 | ILMN_1671536 |
| 1989 | **CEP170** | **2,4** | NM_014812 | ILMN_1755290 |
| 1990 | **TAGLN3** | **2,4** | NM_001008273 | ILMN_1698179 |
| 1991 | **INPP4B** | **2,4** | NM_003866 | ILMN_1767480 |
| 1992 | **AMHR2** | **2,4** | NM_020547 | ILMN_1736412 |
| 1993 | **RANGNRF** | **2,4** | NM_016492 | ILMN_1745760 |
| 1994 | **MGC61571** | **2,4** | NM_182523 | ILMN_1707634 |
| 1995 | **TSPAN7** | **2,4** | NM_004615 | ILMN_1809291 |
| 1996 | **NFIL3** | **2,4** | NM_005384 | ILMN_1707312 |
| 1997 | **CDC26** | **2,4** | NM_139286 | ILMN_1800487 |
| 1998 | **BCDO2** | **2,4** | NM_031938 | ILMN_1711509 |
| 1999 | **ZNF226** | **2,4** | NM_001032372 | ILMN_1715418 |
| 2000 | **NUDCD2** | **2,4** | NM_145266 | ILMN_1751589 |
| 2001 | **HSPB8** | **2,4** | NM_014365 | ILMN_1791280 |
| 2002 | **LTB** | **2,4** | NM_002341 | ILMN_1737043 |
| 2003 | **C11orf10** | **2,4** | NM_014206 | ILMN_1786759 |
| 2004 | **DNAJC17** | **2,4** | NM_018163 | ILMN_1703573 |
| 2005 | **CLTB** | **2,4** | NM_001834 | ILMN_1674609 |
| 2006 | **CALR** | **2,4** | NM_004343 | ILMN_1736256 |
| 2007 | **FBXL19** | **2,4** | NM_019085 | ILMN_1671215 |
| 2008 | **KCNJ10** | **2,4** | NM_002241 | ILMN_1808272 |
| 2009 | **NMB** | **2,4** | NM_021077 | ILMN_1683940 |
| 2010 | **TXNL5** | **2,4** | NM_032731 | ILMN_1659437 |
| 2011 | **HOXB9** | **2,4** | NM_024017 | ILMN_1716708 |
| 2012 | **RPL7** | **2,4** | NM_000971 | ILMN_1815292 |
| 2013 | **ICT1** | **2,4** | NM_001545 | ILMN_1734508 |
| 2014 | **VAMP4** | **2,4** | NM_003762 | ILMN_1761363 |
| 2015 | **GTF2IRD2B** | **2,4** | NM_001003795 | ILMN_1760011 |
| 2016 | **FLJ20674** | **2,4** | NM_019086 | ILMN_1778318 |
| 2017 | **SSTR2** | **2,4** | NM_001050 | ILMN_1803487 |
| 2018 | **SPG21** | **2,4** | NM_016630 | ILMN_1652474 |
| 2019 | **VSIG9** | **2,4** | NM_173799 | ILMN_1770693 |
| 2020 | **TRPT1** | **2,4** | NM_031472 | ILMN_1735909 |
| 2021 | **ALKBH2** | **2,4** | NM_001001655 | ILMN_1690252 |
| 2022 | **THAP4** | **2,4** | NM_015963 | ILMN_1677530 |
| 2023 | **ADA** | **2,4** | NM_000022 | ILMN_1803686 |
| 2024 | **PSENEN** | **2,4** | NM_172341 | ILMN_1669718 |
| 2025 | **SNCB** | **2,4** | NM_001001502 | ILMN_1704570 |
| 2026 | **KIAA1143** | **2,4** | NM_020696 | ILMN_1752273 |
| 2027 | **AUP1** | **2,4** | NM_012103 | ILMN_1651378 |
| 2028 | **KIAA1189** | **2,4** | NM_020711 | ILMN_1659763 |
| 2029 | **CCDC59** | **2,4** | NM_014167 | ILMN_1662318 |
| 2030 | **SAT2** | **2,4** | NM_133491 | ILMN_1746883 |
| 2031 | **CLEC2D** | **2,4** | NM_013269 | ILMN_1711702 |
| 2032 | **C9orf16** | **2,4** | NM_024112 | ILMN_1782618 |
| 2033 | **UTS2** | **2,4** | NM_021995 | ILMN_1671818 |
| 2034 | **EXOSC7** | **2,4** | NM_015004 | ILMN_1726354 |
| 2035 | **EPPB9** | **2,4** | NM_015681 | ILMN_1781281 |
| 2036 | **ACCN4** | **2,4** | NM_018674 | ILMN_1758005 |
| 2037 | **NIFIE14** | **2,4** | NM_032635 | ILMN_1803624 |
| 2038 | **ING1** | **2,4** | NM_198219 | ILMN_1655537 |
| 2039 | **FARSLA** | **2,4** | NM_004461 | ILMN_1778255 |
| 2040 | **LOC283377** | **2,4** | NM_207344 | ILMN_1729868 |
| 2041 | **C1orf41** | **2,4** | NM_016126 | ILMN_1655834 |
| 2042 | **BRWD1** | **2,4** | NM_001007246 | ILMN_1805111 |
| 2043 | **C12orf5** | **2,4** | NM_020375 | ILMN_1791792 |
| 2044 | **CBX3** | **2,4** | NM_016587 | ILMN_1790625 |
| 2045 | **RAD23A** | **2,4** | NM_005053 | ILMN_1751571 |
| 2046 | **TMEPAI** | **2,4** | NM_020182 | ILMN_1774717 |
| 2047 | **VKORC1L1** | **2,4** | NM_173517 | ILMN_1802894 |
| 2048 | **D15Wsu75e** | **2,4** | NM_015704 | ILMN_1737580 |
| 2049 | **RNF6** | **2,4** | NM_183045 | ILMN_1687267 |
| 2050 | **SLC20A1** | **2,4** | NM_005415 | ILMN_1672662 |
| 2051 | **ADAM18** | **2,5** | NM_014237 | ILMN_1694562 |
| 2052 | **CYB561D1** | **2,5** | NM_182580 | ILMN_1803209 |
| 2053 | **CSNK1E** | **2,5** | NM_152221 | ILMN_1724363 |
| 2054 | **MGC24381** | **2,5** | NM_001001410 | ILMN_1713884 |
| 2055 | **CAD** | **2,5** | NM_004341 | ILMN_1810992 |
| 2056 | **STX8** | **2,5** | NM_004853 | ILMN_1752895 |
| 2057 | **WTAP** | **2,5** | NM_152858 | ILMN_1748904 |
| 2058 | **METTL5** | **2,5** | NM_014168 | ILMN_1691570 |
| 2059 | **MIF** | **2,5** | NM_002415 | ILMN_1716169 |
| 2060 | **UNQ846** | **2,5** | NM_207316 | ILMN_1704022 |
| 2061 | **WWP2** | **2,5** | NM_199423 | ILMN_1659703 |
| 2062 | **GIT2** | **2,5** | NM_139201 | ILMN_1760922 |
| 2063 | **FBXL6** | **2,5** | NM_012162 | ILMN_1799389 |
| 2064 | **PTS** | **2,5** | NM_000317 | ILMN_1720322 |
| 2065 | **DCPS** | **2,5** | NM_014026 | ILMN_1740737 |
| 2066 | **METRN** | **2,5** | NM_024042 | ILMN_1712583 |
| 2067 | **C9orf89** | **2,5** | NM_032310 | ILMN_1659189 |
| 2068 | **TMEM42** | **2,5** | NM_144638 | ILMN_1760245 |
| 2069 | **SFTPA2** | **2,5** | NM_006926 | ILMN_1689812 |
| 2070 | **NUP188** | **2,5** | NM_015354 | ILMN_1715416 |
| 2071 | **FLJ37970** | **2,5** | NM_032251 | ILMN_1772208 |
| 2072 | **GART** | **2,5** | NM_175085 | ILMN_1800020 |
| 2073 | **BXDC2** | **2,5** | NM_018321 | ILMN_1679800 |
| 2074 | **BCL3** | **2,5** | NM_005178 | ILMN_1662437 |
| 2075 | **PNPO** | **2,5** | NM_018129 | ILMN_1684289 |
| 2076 | **SPRR1A** | **2,5** | NM_005987 | ILMN_1716591 |
| 2077 | **BLOC1S1** | **2,5** | NM_001487 | ILMN_1767549 |
| 2078 | **EXOSC4** | **2,5** | NM_019037 | ILMN_1745271 |
| 2079 | **NOC2L** | **2,5** | NM_015658 | ILMN_1716428 |
| 2080 | **ENO2** | **2,5** | NM_001975 | ILMN_1765796 |
| 2081 | **TLCD1** | **2,5** | NM_138463 | ILMN_1784655 |
| 2082 | **TULP2** | **2,5** | NM_003323 | ILMN_1654618 |
| 2083 | **RIT1** | **2,5** | NM_006912 | ILMN_1656335 |
| 2084 | **BAK1** | **2,5** | NM_001188 | ILMN_1805990 |
| 2085 | **CTXN1** | **2,5** | NM_206833 | ILMN_1759766 |
| 2086 | **GSS** | **2,5** | NM_000178 | ILMN_1683462 |
| 2087 | **C6orf49** | **2,5** | NM_013397 | ILMN_1695868 |
| 2088 | **PRAF2** | **2,5** | NM_007213 | ILMN_1720578 |
| 2089 | **FLJ20516** | **2,5** | NM_017858 | ILMN_1761939 |
| 2090 | **C17orf79** | **2,5** | NM_018405 | ILMN_1752947 |
| 2091 | **NOLA2** | **2,5** | NM_017838 | ILMN_1718672 |
| 2092 | **DMRTC1** | **2,5** | NM_033053 | ILMN_1661486 |
| 2093 | **POLR2H** | **2,5** | NM_006232 | ILMN_1689445 |
| 2094 | **ANP32A** | **2,5** | NM_006305 | ILMN_1803500 |
| 2095 | **FCRL6** | **2,5** | NM_001004310 | ILMN_1661591 |
| 2096 | **STX7** | **2,5** | NM_003569 | ILMN_1792518 |
| 2097 | **DUSP23** | **2,5** | NM_017823 | ILMN_1659462 |
| 2098 | **ELSPBP1** | **2,5** | NM_022142 | ILMN_1719183 |
| 2099 | **ITGB3** | **2,5** | NM_000212 | ILMN_1661614 |
| 2100 | **SMOX** | **2,5** | NM_019025 | ILMN_1775380 |
| 2101 | **15E1.2** | **2,5** | NM_176818 | ILMN_1809034 |
| 2102 | **MRPL23** | **2,5** | NM_021134 | ILMN_1806123 |
| 2103 | **RUNX1** | **2,5** | NM_001001890 | ILMN_1654941 |
| 2104 | **KCNG1** | **2,5** | NM_002237 | ILMN_1673769 |
| 2105 | **NKAP** | **2,5** | NM_024528 | ILMN_1675359 |
| 2106 | **BZRP** | **2,5** | NM_000714 | ILMN_1754663 |
| 2107 | **FGFR1** | **2,5** | NM_023110 | ILMN_1796229 |
| 2108 | **MANBAL** | **2,5** | NM_022077 | ILMN_1661958 |
| 2109 | **C12orf47** | **2,5** | NM_016534 | ILMN_1798957 |
| 2110 | **GPC2** | **2,5** | NM_152742 | ILMN_1651642 |
| 2111 | **SNRPD2** | **2,5** | NM_004597 | ILMN_1679088 |
| 2112 | **PHF19** | **2,5** | NM_001009936 | ILMN_1745420 |
| 2113 | **LOC221143** | **2,5** | NM_174928 | ILMN_1730260 |
| 2114 | **POLR3D** | **2,5** | NM_001722 | ILMN_1685109 |
| 2115 | **CD63** | **2,5** | NM_001780 | ILMN_1753468 |
| 2116 | **C11orf17** | **2,5** | NM_020642 | ILMN_1788356 |
| 2117 | **NUDT3** | **2,5** | NM_006703 | ILMN_1724907 |
| 2118 | **CTNNAL1** | **2,5** | NM_003798 | ILMN_1721901 |
| 2119 | **NUDT8** | **2,5** | NM_181843 | ILMN_1768712 |
| 2120 | **QIL1** | **2,5** | NM_205767 | ILMN_1765684 |
| 2121 | **FLJ39575** | **2,5** | NM_182597 | ILMN_1804895 |
| 2122 | **PIP3-E** | **2,5** | NM_015553 | ILMN_1796497 |
| 2123 | **LSM5** | **2,5** | NM_012322 | ILMN_1737947 |
| 2124 | **RGS16** | **2,6** | NM_002928 | ILMN_1800834 |
| 2125 | **MTCP1** | **2,6** | NM_001018025 | ILMN_1814230 |
| 2126 | **ARFGEF2** | **2,6** | NM_006420 | ILMN_1789990 |
| 2127 | **EIF3S8** | **2,6** | NM_003752 | ILMN_1725984 |
| 2128 | **GAJ** | **2,6** | NM_032117 | ILMN_1671906 |
| 2129 | **MAGOH** | **2,6** | NM_002370 | ILMN_1814396 |
| 2130 | **LOC283951** | **2,6** | NM_001010878 | ILMN_1759325 |
| 2131 | **TMEM58** | **2,6** | NM_198149 | ILMN_1677942 |
| 2132 | **NFS1** | **2,6** | NM_181679 | ILMN_1761314 |
| 2133 | **C6orf173** | **2,6** | NM_001012507 | ILMN_1763907 |
| 2134 | **CDC42** | **2,6** | NM_044472 | ILMN_1675156 |
| 2135 | **FDXR** | **2,6** | NM_024417 | ILMN_1799319 |
| 2136 | **RPL32** | **2,6** | NM_001007074 | ILMN_1769741 |
| 2137 | **HES6** | **2,6** | NM_018645 | ILMN_1694268 |
| 2138 | **TNFRSF6B** | **2,6** | NM_032945 | ILMN_1656151 |
| 2139 | **JPH2** | **2,6** | NM_175913 | ILMN_1802434 |
| 2140 | **C14orf2** | **2,6** | NM_004894 | ILMN_1652722 |
| 2141 | **HIST1H3H** | **2,6** | NM_003536 | ILMN_1749368 |
| 2142 | **PPAN** | **2,6** | NM_020230 | ILMN_1803613 |
| 2143 | **C12orf24** | **2,6** | NM_013300 | ILMN_1753781 |
| 2144 | **MGC13114** | **2,6** | NM_032366 | ILMN_1774990 |
| 2145 | **HSD17B8** | **2,6** | NM_014234 | ILMN_1715324 |
| 2146 | **SLC6A6** | **2,6** | NM_003043 | ILMN_1673586 |
| 2147 | **CLK3** | **2,6** | NM_003992 | ILMN_1695961 |
| 2148 | **SH2D2A** | **2,6** | NM_003975 | ILMN_1766319 |
| 2149 | **TIMP1** | **2,6** | NM_003254 | ILMN_1711566 |
| 2150 | **FKBP11** | **2,6** | NM_016594 | ILMN_1787345 |
| 2151 | **RAC3** | **2,6** | NM_005052 | ILMN_1693340 |
| 2152 | **CDH9** | **2,6** | NM_016279 | ILMN_1736518 |
| 2153 | **USH3A** | **2,6** | NM_052995 | ILMN_1698745 |
| 2154 | **C1orf97** | **2,6** | NM_032705 | ILMN_1713411 |
| 2155 | **DMRT1** | **2,6** | NM_021951 | ILMN_1803113 |
| 2156 | **PPIAL4** | **2,6** | NM_178230 | ILMN_1776260 |
| 2157 | **SCG2** | **2,6** | NM_003469 | ILMN_1703178 |
| 2158 | **NTNG2** | **2,6** | NM_032536 | ILMN_1667985 |
| 2159 | **ZA20D1** | **2,6** | NM_020205 | ILMN_1661689 |
| 2160 | **CT45-4** | **2,6** | NM_001017436 | ILMN_1672783 |
| 2161 | **PRKAR1B** | **2,6** | NM_002735 | ILMN_1674390 |
| 2162 | **OR2T35** | **2,6** | NM_001001827 | ILMN_1700439 |
| 2163 | **MRPS30** | **2,6** | NM_016640 | ILMN_1726743 |
| 2164 | **WBSCR27** | **2,6** | NM_152559 | ILMN_1719170 |
| 2165 | **EDA2R** | **2,6** | NM_021783 | ILMN_1767233 |
| 2166 | **SLC25A22** | **2,6** | NM_024698 | ILMN_1807014 |
| 2167 | **UCK2** | **2,6** | NM_012474 | ILMN_1768662 |
| 2168 | **LOC440686** | **2,6** | NM_001025303 | ILMN_1664706 |
| 2169 | **DPP7** | **2,6** | NM_013379 | ILMN_1811328 |
| 2170 | **MRPL54** | **2,6** | NM_172251 | ILMN_1658486 |
| 2171 | **SCFD2** | **2,6** | NM_152540 | ILMN_1708891 |
| 2172 | **ATP6V0C** | **2,6** | NM_001694 | ILMN_1773849 |
| 2173 | **GCNT4** | **2,6** | NM_016591 | ILMN_1742065 |
| 2174 | **CBX5** | **2,6** | NM_012117 | ILMN_1726134 |
| 2175 | **S100A14** | **2,6** | NM_020672 | ILMN_1783287 |
| 2176 | **UIP1** | **2,6** | NM_207107 | ILMN_1694890 |
| 2177 | **NEDD8** | **2,6** | NM_006156 | ILMN_1785711 |
| 2178 | **POLB** | **2,6** | NM_002690 | ILMN_1767894 |
| 2179 | **C16orf60** | **2,6** | NM_018455 | ILMN_1720526 |
| 2180 | **H2-ALPHA** | **2,6** | NM_080386 | ILMN_1712147 |
| 2181 | **K6HF** | **2,6** | NM_004693 | ILMN_1721247 |
| 2182 | **FLAD1** | **2,6** | NM_201398 | ILMN_1663667 |
| 2183 | **RELB** | **2,6** | NM_006509 | ILMN_1811258 |
| 2184 | **ATPBD3** | **2,6** | NM_145232 | ILMN_1682375 |
| 2185 | **C18orf22** | **2,6** | NM_024805 | ILMN_1736130 |
| 2186 | **CHCHD8** | **2,6** | NM_016565 | ILMN_1732750 |
| 2187 | **CX3CL1** | **2,6** | NM_002996 | ILMN_1654072 |
| 2188 | **SFRS14** | **2,6** | NM_014884 | ILMN_1772487 |
| 2189 | **C22orf18** | **2,6** | NM_024053 | ILMN_1668814 |
| 2190 | **CXorf38** | **2,6** | NM_144970 | ILMN_1697864 |
| 2191 | **GOLGA2** | **2,6** | NM_004486 | ILMN_1738821 |
| 2192 | **NENF** | **2,6** | NM_013349 | ILMN_1796438 |
| 2193 | **CDKN3** | **2,6** | NM_005192 | ILMN_1666305 |
| 2194 | **ABHD2** | **2,6** | NM_007011 | ILMN_1723662 |
| 2195 | **FLJ22639** | **2,6** | NM_024796 | ILMN_1702866 |
| 2196 | **MRPL52** | **2,6** | NM_180981 | ILMN_1758578 |
| 2197 | **SMG5** | **2,6** | NM_015327 | ILMN_1721735 |
| 2198 | **CALN1** | **2,6** | NM_031468 | ILMN_1778121 |
| 2199 | **LOC201164** | **2,6** | NM_178836 | ILMN_1731518 |
| 2200 | **UBE2T** | **2,7** | NM_014176 | ILMN_1711470 |
| 2201 | **LOC389289** | **2,7** | NM_001014279 | ILMN_1675465 |
| 2202 | **ZNF419** | **2,7** | NM_024691 | ILMN_1680693 |
| 2203 | **POLR2J** | **2,7** | NM_006234 | ILMN_1657317 |
| 2204 | **RPRC1** | **2,7** | NM_018067 | ILMN_1733348 |
| 2205 | **CDV3** | **2,7** | NM_017548 | ILMN_1691106 |
| 2206 | **NXT1** | **2,7** | NM_013248 | ILMN_1760280 |
| 2207 | **NDUFS6** | **2,7** | NM_004553 | ILMN_1706765 |
| 2208 | **MT1F** | **2,7** | NM_005949 | ILMN_1718766 |
| 2209 | **RRAGD** | **2,7** | NM_021244 | ILMN_1699772 |
| 2210 | **MAPBPIP** | **2,7** | NM_014017 | ILMN_1756352 |
| 2211 | **TNFAIP3** | **2,7** | NM_006290 | ILMN_1702691 |
| 2212 | **TUFT1** | **2,7** | NM_020127 | ILMN_1781374 |
| 2213 | **FAM14B** | **2,7** | NM_145249 | ILMN_1709626 |
| 2214 | **NKD1** | **2,7** | NM_033119 | ILMN_1692674 |
| 2215 | **PARVB** | **2,7** | NM_013327 | ILMN_1787919 |
| 2216 | **C6orf129** | **2,7** | NM_138493 | ILMN_1651987 |
| 2217 | **EEF1D** | **2,7** | NM_032378 | ILMN_1782543 |
| 2218 | **C1orf63** | **2,7** | NM_020317 | ILMN_1749915 |
| 2219 | **SELPLG** | **2,7** | NM_003006 | ILMN_1760560 |
| 2220 | **NR4A1** | **2,7** | NM_173158 | ILMN_1772351 |
| 2221 | **MAD2L2** | **2,7** | NM_006341 | ILMN_1669550 |
| 2222 | **FAM98C** | **2,7** | NM_174905 | ILMN_1760609 |
| 2223 | **PELP1** | **2,7** | NM_014389 | ILMN_1728684 |
| 2224 | **C21orf119** | **2,7** | NM_032910 | ILMN_1728403 |
| 2225 | **C18orf56** | **2,7** | NM_001012716 | ILMN_1796074 |
| 2226 | **PTK2** | **2,7** | NM_153831 | ILMN_1676305 |
| 2227 | **KREMEN2** | **2,7** | NM_024507 | ILMN_1795710 |
| 2228 | **RPL26L1** | **2,7** | NM_016093 | ILMN_1776586 |
| 2229 | **FLNA** | **2,7** | NM_001456 | ILMN_1687335 |
| 2230 | **MEA1** | **2,7** | NM_014623 | ILMN_1727073 |
| 2231 | **ARPC5L** | **2,7** | NM_030978 | ILMN_1800844 |
| 2232 | **TRIM9** | **2,7** | NM_052978 | ILMN_1786697 |
| 2233 | **ABCA12** | **2,7** | NM_015657 | ILMN_1701642 |
| 2234 | **GPS2** | **2,7** | NM_004489 | ILMN_1815158 |
| 2235 | **EXOSC8** | **2,7** | NM_181503 | ILMN_1756162 |
| 2236 | **ZNF655** | **2,7** | NM_001009957 | ILMN_1765254 |
| 2237 | **RBM19** | **2,7** | NM_016196 | ILMN_1774839 |
| 2238 | **SLC2A8** | **2,7** | NM_014580 | ILMN_1724609 |
| 2239 | **TMEM126A** | **2,7** | NM_032273 | ILMN_1679542 |
| 2240 | **LIN7B** | **2,7** | NM_022165 | ILMN_1663444 |
| 2241 | **MAPK1** | **2,7** | NM_138957 | ILMN_1665276 |
| 2242 | **FAM18B2** | **2,7** | NM_145301 | ILMN_1731972 |
| 2243 | **ZNF265** | **2,7** | NM_005455 | ILMN_1662383 |
| 2244 | **TSTA3** | **2,7** | NM_003313 | ILMN_1697777 |
| 2245 | **KALRN** | **2,7** | NM_003947 | ILMN_1707798 |
| 2246 | **MRPS24** | **2,7** | NM_032014 | ILMN_1802553 |
| 2247 | **MRPL40** | **2,7** | NM_003776 | ILMN_1687403 |
| 2248 | **NDUFA12** | **2,7** | NM_018838 | ILMN_1803632 |
| 2249 | **OPRS1** | **2,7** | NM_147160 | ILMN_1717925 |
| 2250 | **SLC29A1** | **2,7** | NM_004955 | ILMN_1723971 |
| 2251 | **CSRP2** | **2,7** | NM_001321 | ILMN_1660806 |
| 2252 | **NDUFB10** | **2,7** | NM_004548 | ILMN_1811754 |
| 2253 | **ANAPC10** | **2,7** | NM_014885 | ILMN_1770378 |
| 2254 | **TNPO2** | **2,7** | NM_013433 | ILMN_1656066 |
| 2255 | **FBXO6** | **2,7** | NM_018438 | ILMN_1701455 |
| 2256 | **MRPS21** | **2,7** | NM_018997 | ILMN_1660292 |
| 2257 | **ANAPC11** | **2,8** | NM_001002247 | ILMN_1722102 |
| 2258 | **HAGHL** | **2,8** | NM_032304 | ILMN_1793201 |
| 2259 | **PCK2** | **2,8** | NM_001018073 | ILMN_1802699 |
| 2260 | **UBE2V1** | **2,8** | NM_001032288 | ILMN_1726107 |
| 2261 | **ID3** | **2,8** | NM_002167 | ILMN_1732296 |
| 2262 | **LOC388799** | **2,8** | NM_001013646 | ILMN_1714667 |
| 2263 | **POLR2F** | **2,8** | NM_021974 | ILMN_1745885 |
| 2264 | **SLC19A2** | **2,8** | NM_006996 | ILMN_1729594 |
| 2265 | **LOC391356** | **2,8** | NM_001013663 | ILMN_1692707 |
| 2266 | **ERCC1** | **2,8** | NM_202001 | ILMN_1676748 |
| 2267 | **GEM** | **2,8** | NM_005261 | ILMN_1677092 |
| 2268 | **S100A3** | **2,8** | NM_002960 | ILMN_1712545 |
| 2269 | **C6orf136** | **2,8** | NM_145029 | ILMN_1813236 |
| 2270 | **MTMR1** | **2,8** | NM_176789 | ILMN_1741556 |
| 2271 | **FAM86B1** | **2,8** | NM_032916 | ILMN_1655051 |
| 2272 | **EYA3** | **2,8** | NM_172098 | ILMN_1790562 |
| 2273 | **MAPRE3** | **2,8** | NM_012326 | ILMN_1734290 |
| 2274 | **BIRC3** | **2,8** | NM_001165 | ILMN_1696549 |
| 2275 | **MRPL11** | **2,8** | NM_016050 | ILMN_1690371 |
| 2276 | **LYAR** | **2,8** | NM_017816 | ILMN_1764362 |
| 2277 | **LOC144501** | **2,8** | NM_182507 | ILMN_1705814 |
| 2278 | **KRTCAP2** | **2,8** | NM_173852 | ILMN_1658802 |
| 2279 | **SLC35F3** | **2,8** | NM_173508 | ILMN_1794959 |
| 2280 | **C19orf33** | **2,8** | NM_033520 | ILMN_1717793 |
| 2281 | **DKFZP564J0863** | **2,8** | NM_015459 | ILMN_1751086 |
| 2282 | **C16orf61** | **2,8** | NM_020188 | ILMN_1783333 |
| 2283 | **LAMC2** | **2,8** | NM_005562 | ILMN_1701424 |
| 2284 | **NHLRC2** | **2,8** | NM_198514 | ILMN_1698715 |
| 2285 | **SAT** | **2,8** | NM_002970 | ILMN_1753342 |
| 2286 | **SCNM1** | **2,8** | NM_024041 | ILMN_1746598 |
| 2287 | **PINX1** | **2,8** | NM_017884 | ILMN_1790309 |
| 2288 | **PA2G4** | **2,8** | NM_006191 | ILMN_1728984 |
| 2289 | **C3orf26** | **2,8** | NM_032359 | ILMN_1705753 |
| 2290 | **TMLHE** | **2,8** | NM_018196 | ILMN_1683575 |
| 2291 | **C17orf61** | **2,8** | NM_152766 | ILMN_1737358 |
| 2292 | **TCEB1** | **2,8** | NM_005648 | ILMN_1704873 |
| 2293 | **CA5B** | **2,8** | NM_007220 | ILMN_1672807 |
| 2294 | **RBM18** | **2,8** | NM_033117 | ILMN_1802355 |
| 2295 | **HSPC111** | **2,8** | NM_016391 | ILMN_1704055 |
| 2296 | **ATP5J** | **2,8** | NM_001003703 | ILMN_1661574 |
| 2297 | **AARSD1** | **2,8** | NM_025267 | ILMN_1700461 |
| 2298 | **WWOX** | **2,8** | NM_130844 | ILMN_1730570 |
| 2299 | **NUTF2** | **2,8** | NM_005796 | ILMN_1655046 |
| 2300 | **SLC6A9** | **2,8** | NM_001024845 | ILMN_1754262 |
| 2301 | **ELOF1** | **2,8** | NM_032377 | ILMN_1723185 |
| 2302 | **CHMP2A** | **2,8** | NM_014453 | ILMN_1656621 |
| 2303 | **JOSD3** | **2,8** | NM_024116 | ILMN_1682038 |
| 2304 | **LSM12** | **2,8** | NM_152344 | ILMN_1734428 |
| 2305 | **RP9** | **2,8** | NM_203288 | ILMN_1687922 |
| 2306 | **PLP2** | **2,9** | NM_002668 | ILMN_1738767 |
| 2307 | **VKORC1** | **2,9** | NM_024006 | ILMN_1786139 |
| 2308 | **SHFM1** | **2,9** | NM_006304 | ILMN_1794505 |
| 2309 | **RHCG** | **2,9** | NM_016321 | ILMN_1778687 |
| 2310 | **SUHW4** | **2,9** | NM_001002844 | ILMN_1767142 |
| 2311 | **ACSS2** | **2,9** | NM_018677 | ILMN_1697510 |
| 2312 | **KHSRP** | **2,9** | NM_003685 | ILMN_1673936 |
| 2313 | **ACAD11** | **2,9** | NM_032169 | ILMN_1761058 |
| 2314 | **SHB** | **2,9** | NM_003028 | ILMN_1732612 |
| 2315 | **HSF1** | **2,9** | NM_005526 | ILMN_1667060 |
| 2316 | **C1orf31** | **2,9** | NM_001012985 | ILMN_1681741 |
| 2317 | **MGC13096** | **2,9** | NM_032346 | ILMN_1706149 |
| 2318 | **NFKBIZ** | **2,9** | NM_001005474 | ILMN_1719695 |
| 2319 | **MESP1** | **2,9** | NM_018670 | ILMN_1806603 |
| 2320 | **LSM10** | **2,9** | NM_032881 | ILMN_1751803 |
| 2321 | **C6orf108** | **2,9** | NM_199184 | ILMN_1769343 |
| 2322 | **SOCS7** | **2,9** | NM_014598 | ILMN_1753683 |
| 2323 | **RP11-529I10.4** | **2,9** | NM_015448 | ILMN_1744584 |
| 2324 | **SELM** | **2,9** | NM_080430 | ILMN_1651429 |
| 2325 | **PSMD13** | **2,9** | NM_002817 | ILMN_1704404 |
| 2326 | **HIST1H2BJ** | **2,9** | NM_021058 | ILMN_1658702 |
| 2327 | **ODF2** | **2,9** | NM_153437 | ILMN_1698653 |
| 2328 | **CPT1C** | **2,9** | NM_152359 | ILMN_1773855 |
| 2329 | **C1orf122** | **2,9** | NM_198446 | ILMN_1786273 |
| 2330 | **FLJ14466** | **2,9** | NM_032790 | ILMN_1724148 |
| 2331 | **NDUFA11** | **2,9** | NM_175614 | ILMN_1682299 |
| 2332 | **MRPL36** | **2,9** | NM_032479 | ILMN_1800197 |
| 2333 | **SURF2** | **2,9** | NM_017503 | ILMN_1781628 |
| 2334 | **SERPINB8** | **2,9** | NM_001031848 | ILMN_1718960 |
| 2335 | **C17orf48** | **2,9** | NM_020233 | ILMN_1702526 |
| 2336 | **C6orf66** | **2,9** | NM_014165 | ILMN_1659524 |
| 2337 | **GADD45GIP1** | **2,9** | NM_052850 | ILMN_1751530 |
| 2338 | **IHPK2** | **2,9** | NM_001005911 | ILMN_1683328 |
| 2339 | **EREG** | **2,9** | NM_001432 | ILMN_1657248 |
| 2340 | **COMMD1** | **3,0** | NM_152516 | ILMN_1761242 |
| 2341 | **MRPL33** | **3,0** | NM_004891 | ILMN_1731599 |
| 2342 | **RPL6** | **3,0** | NM_001024662 | ILMN_1717490 |
| 2343 | **MCTS1** | **3,0** | NM_014060 | ILMN_1751816 |
| 2344 | **MPV17** | **3,0** | NM_002437 | ILMN_1691090 |
| 2345 | **RPL35** | **3,0** | NM_007209 | ILMN_1775243 |
| 2346 | **SRRM2** | **3,0** | NM_016333 | ILMN_1764460 |
| 2347 | **RPL39L** | **3,0** | NM_052969 | ILMN_1712413 |
| 2348 | **TIMM10** | **3,0** | NM_012456 | ILMN_1765332 |
| 2349 | **ABI2** | **3,0** | NM_005759 | ILMN_1724497 |
| 2350 | **HAX1** | **3,0** | NM_001018837 | ILMN_1750658 |
| 2351 | **LOC115098** | **3,0** | NM_138442 | ILMN_1811775 |
| 2352 | **PBX1** | **3,0** | NM_002585 | ILMN_1784678 |
| 2353 | **TNFRSF9** | **3,0** | NM_001561 | ILMN_1813379 |
| 2354 | **UBE2I** | **3,0** | NM_194259 | ILMN_1664542 |
| 2355 | **IRF1** | **3,0** | NM_002198 | ILMN_1708375 |
| 2356 | **QP-C** | **3,0** | NM_014402 | ILMN_1666471 |
| 2357 | **IFI30** | **3,0** | NM_006332 | ILMN_1682846 |
| 2358 | **SERF2** | **3,0** | NM_001018108 | ILMN_1789136 |
| 2359 | **NARG2** | **3,0** | NM_024611 | ILMN_1718830 |
| 2360 | **ORF1-FL49** | **3,0** | NM_032412 | ILMN_1761566 |
| 2361 | **GOSR2** | **3,0** | NM_054022 | ILMN_1656293 |
| 2362 | **NDUFB7** | **3,0** | NM_004146 | ILMN_1813604 |
| 2363 | **HOMER2** | **3,0** | NM_004839 | ILMN_1671486 |
| 2364 | **GIP** | **3,0** | NM_004123 | ILMN_1673805 |
| 2365 | **DYNLL2** | **3,0** | NM_080677 | ILMN_1772796 |
| 2366 | **NRP1** | **3,0** | NM_001024629 | ILMN_1742547 |
| 2367 | **MORN2** | **3,0** | NM_194270 | ILMN_1756826 |
| 2368 | **LOC400506** | **3,0** | NM_001012991 | ILMN_1712664 |
| 2369 | **MYOHD1** | **3,0** | NM_001033580 | ILMN_1750711 |
| 2370 | **PPP1R14A** | **3,0** | NM_033256 | ILMN_1761968 |
| 2371 | **HCFC1R1** | **3,0** | NM_001002017 | ILMN_1757877 |
| 2372 | **RGS10** | **3,1** | NM_001005339 | ILMN_1668559 |
| 2373 | **LOC441150** | **3,1** | NM_001008739 | ILMN_1743755 |
| 2374 | **EIF2B3** | **3,1** | NM_020365 | ILMN_1689233 |
| 2375 | **MGC15416** | **3,1** | NM_138418 | ILMN_1730523 |
| 2376 | **TP53AP1** | **3,1** | NM_007233 | ILMN_1658469 |
| 2377 | **NSUN5C** | **3,1** | NM_149379 | ILMN_1688070 |
| 2378 | **C9orf30** | **3,1** | NM_080655 | ILMN_1714278 |
| 2379 | **SEC61B** | **3,1** | NM_006808 | ILMN_1736389 |
| 2380 | **HSPC171** | **3,1** | NM_014187 | ILMN_1700419 |
| 2381 | **AURKAIP1** | **3,1** | NM_017900 | ILMN_1700793 |
| 2382 | **PTMS** | **3,1** | NM_002824 | ILMN_1721046 |
| 2383 | **NDUFB3** | **3,1** | NM_002491 | ILMN_1718428 |
| 2384 | **EDF1** | **3,1** | NM_003792 | ILMN_1696544 |
| 2385 | **PTGS2** | **3,1** | NM_000963 | ILMN_1677511 |
| 2386 | **PROCR** | **3,1** | NM_006404 | ILMN_1717262 |
| 2387 | **RNF7** | **3,1** | NM_183063 | ILMN_1731123 |
| 2388 | **CHCHD5** | **3,1** | NM_032309 | ILMN_1797530 |
| 2389 | **TMEM11** | **3,1** | NM_003876 | ILMN_1712050 |
| 2390 | **LIX1L** | **3,1** | NM_153713 | ILMN_1708098 |
| 2391 | **PEMT** | **3,1** | NM_148172 | ILMN_1727855 |
| 2392 | **POLR2I** | **3,1** | NM_006233 | ILMN_1720542 |
| 2393 | **ING5** | **3,1** | NM_032329 | ILMN_1724578 |
| 2394 | **BLVRB** | **3,1** | NM_000713 | ILMN_1797793 |
| 2395 | **ZNF580** | **3,1** | NM_207115 | ILMN_1688565 |
| 2396 | **EBI3** | **3,1** | NM_005755 | ILMN_1802653 |
| 2397 | **U2AF1L3** | **3,1** | NM_144987 | ILMN_1779177 |
| 2398 | **DDT** | **3,1** | NM_001355 | ILMN_1690982 |
| 2399 | **MRPL27** | **3,1** | NM_016504 | ILMN_1811327 |
| 2400 | **RBAF600** | **3,1** | NM_020765 | ILMN_1699226 |
| 2401 | **STRA13** | **3,1** | NM_144998 | ILMN_1769634 |
| 2402 | **UQCRB** | **3,1** | NM_006294 | ILMN_1759453 |
| 2403 | **UBPH** | **3,1** | NM_019116 | ILMN_1700811 |
| 2404 | **SEPW1** | **3,2** | NM_003009 | ILMN_1769226 |
| 2405 | **COX7B** | **3,2** | NM_001866 | ILMN_1794223 |
| 2406 | **CGI-96** | **3,2** | NM_015703 | ILMN_1688178 |
| 2407 | **NDUFS7** | **3,2** | NM_024407 | ILMN_1669966 |
| 2408 | **MRPL12** | **3,2** | NM_002949 | ILMN_1699603 |
| 2409 | **ADK** | **3,2** | NM_006721 | ILMN_1768062 |
| 2410 | **UBE2H** | **3,2** | NM_003344 | ILMN_1674633 |
| 2411 | **GPR175** | **3,2** | NM_016372 | ILMN_1804938 |
| 2412 | **APOC1** | **3,2** | NM_001645 | ILMN_1789007 |
| 2413 | **C7orf24** | **3,2** | NM_024051 | ILMN_1703858 |
| 2414 | **C6orf141** | **3,2** | NM_153344 | ILMN_1761762 |
| 2415 | **RPL23** | **3,2** | NM_000978 | ILMN_1755115 |
| 2416 | **LETM2** | **3,2** | NM_144652 | ILMN_1714223 |
| 2417 | **C14orf118** | **3,2** | NM_017926 | ILMN_1731269 |
| 2418 | **ZNF585A** | **3,2** | NM_152655 | ILMN_1764415 |
| 2419 | **GNG5** | **3,2** | NM_005274 | ILMN_1701854 |
| 2420 | **C18orf21** | **3,2** | NM_031446 | ILMN_1805998 |
| 2421 | **LOC389541** | **3,2** | NM_001008395 | ILMN_1779735 |
| 2422 | **C17orf49** | **3,2** | NM_174893 | ILMN_1763688 |
| 2423 | **PMS2L3** | **3,2** | NM_001003686 | ILMN_1699049 |
| 2424 | **ARL6IP4** | **3,2** | NM_018694 | ILMN_1791149 |
| 2425 | **TAF13** | **3,2** | NM_005645 | ILMN_1712561 |
| 2426 | **PNN** | **3,2** | NM_002687 | ILMN_1721703 |
| 2427 | **TRMT1** | **3,2** | NM_017722 | ILMN_1812940 |
| 2428 | **CAPN10** | **3,2** | NM_023089 | ILMN_1677357 |
| 2429 | **NUDT1** | **3,3** | NM_198954 | ILMN_1735692 |
| 2430 | **TIMM17B** | **3,3** | NM_005834 | ILMN_1813260 |
| 2431 | **PUSL1** | **3,3** | NM_153339 | ILMN_1780315 |
| 2432 | **IER3** | **3,3** | NM_052815 | ILMN_1682717 |
| 2433 | **38231** | **3,3** | NM_080417 | ILMN_1776157 |
| 2434 | **C19orf24** | **3,3** | NM_017914 | ILMN_1678052 |
| 2435 | **IMP4** | **3,3** | NM_033416 | ILMN_1661347 |
| 2436 | **MRPL14** | **3,3** | NM_032111 | ILMN_1727004 |
| 2437 | **HSPC176** | **3,3** | NM_016209 | ILMN_1747058 |
| 2438 | **HSPC159** | **3,3** | NM_014181 | ILMN_1673548 |
| 2439 | **DLL3** | **3,3** | NM_016941 | ILMN_1736096 |
| 2440 | **NDUFB6** | **3,3** | NM_002493 | ILMN_1763147 |
| 2441 | **WDR33** | **3,3** | NM_001006622 | ILMN_1716086 |
| 2442 | **OCIAD2** | **3,3** | NM_152398 | ILMN_1772286 |
| 2443 | **BNIP1** | **3,3** | NM_013978 | ILMN_1734410 |
| 2444 | **LARP6** | **3,3** | NM_197958 | ILMN_1663401 |
| 2445 | **HIST1H4K** | **3,3** | NM_003541 | ILMN_1662359 |
| 2446 | **NDUFA7** | **3,4** | NM_005001 | ILMN_1675239 |
| 2447 | **MRPL30** | **3,4** | NM_145213 | ILMN_1766154 |
| 2448 | **KRT10** | **3,4** | NM_000421 | ILMN_1716093 |
| 2449 | **YIF1B** | **3,4** | NM_033557 | ILMN_1740386 |
| 2450 | **ZNF259** | **3,4** | NM_003904 | ILMN_1753790 |
| 2451 | **ATP5G2** | **3,4** | NM_005176 | ILMN_1669102 |
| 2452 | **MGC4172** | **3,4** | NM_024308 | ILMN_1756701 |
| 2453 | **KIAA1754** | **3,4** | NM_033397 | ILMN_1805192 |
| 2454 | **BLOC1S2** | **3,4** | NM_001001342 | ILMN_1679782 |
| 2455 | **ZNF342** | **3,4** | NM_145288 | ILMN_1693242 |
| 2456 | **EPHA2** | **3,4** | NM_004431 | ILMN_1700527 |
| 2457 | **PFDN6** | **3,4** | NM_014260 | ILMN_1661490 |
| 2458 | **UCHL3** | **3,4** | NM_006002 | ILMN_1660111 |
| 2459 | **EIF4EBP1** | **3,4** | NM_004095 | ILMN_1767324 |
| 2460 | **CCDC72** | **3,4** | NM_015933 | ILMN_1707783 |
| 2461 | **DXS9879E** | **3,4** | NM_006014 | ILMN_1708151 |
| 2462 | **RPS28** | **3,4** | NM_001031 | ILMN_1651228 |
| 2463 | **PBEF1** | **3,4** | NM_182790 | ILMN_1753111 |
| 2464 | **C14orf151** | **3,4** | NM_032714 | ILMN_1729596 |
| 2465 | **DPM2** | **3,5** | NM_003863 | ILMN_1732049 |
| 2466 | **TCEB2** | **3,5** | NM_007108 | ILMN_1733927 |
| 2467 | **ITGAE** | **3,5** | NM_002208 | ILMN_1683927 |
| 2468 | **TAF10** | **3,5** | NM_006284 | ILMN_1721093 |
| 2469 | **HK2** | **3,5** | NM_000189 | ILMN_1723486 |
| 2470 | **HPS1** | **3,5** | NM_182639 | ILMN_1700207 |
| 2471 | **MRPS17** | **3,5** | NM_015969 | ILMN_1804851 |
| 2472 | **SLC25A25** | **3,5** | NM_052901 | ILMN_1791728 |
| 2473 | **MGC71993** | **3,5** | NM_001004333 | ILMN_1715698 |
| 2474 | **MKKS** | **3,5** | NM_170784 | ILMN_1728276 |
| 2475 | **ATP5L** | **3,5** | NM_006476 | ILMN_1812638 |
| 2476 | **SH3BGRL3** | **3,5** | NM_031286 | ILMN_1737163 |
| 2477 | **EMG1** | **3,5** | NM_006331 | ILMN_1797074 |
| 2478 | **C9orf142** | **3,5** | NM_183241 | ILMN_1761138 |
| 2479 | **NDUFA13** | **3,5** | NM_015965 | ILMN_1767139 |
| 2480 | **HES4** | **3,5** | NM_021170 | ILMN_1653466 |
| 2481 | **ATP5J2** | **3,5** | NM_001003714 | ILMN_1750143 |
| 2482 | **C14orf156** | **3,6** | NM_031210 | ILMN_1661945 |
| 2483 | **FABP5** | **3,6** | NM_001444 | ILMN_1696302 |
| 2484 | **LSMD1** | **3,6** | NM_032356 | ILMN_1733960 |
| 2485 | **PDCD5** | **3,6** | NM_004708 | ILMN_1668425 |
| 2486 | **KLF6** | **3,6** | NM_001008490 | ILMN_1702995 |
| 2487 | **FAM96B** | **3,6** | NM_016062 | ILMN_1779813 |
| 2488 | **RNU3IP2** | **3,6** | NM_004704 | ILMN_1795758 |
| 2489 | **NDUFB9** | **3,6** | NM_005005 | ILMN_1666326 |
| 2490 | **MTA2** | **3,6** | NM_004739 | ILMN_1773763 |
| 2491 | **CHCHD6** | **3,6** | NM_032343 | ILMN_1785161 |
| 2492 | **ASPHD1** | **3,6** | NM_181718 | ILMN_1662390 |
| 2493 | **C6orf57** | **3,6** | NM_145267 | ILMN_1728057 |
| 2494 | **USP2** | **3,6** | NM_004205 | ILMN_1653940 |
| 2495 | **GLRX2** | **3,6** | NM_197962 | ILMN_1734903 |
| 2496 | **MRPL53** | **3,6** | NM_053050 | ILMN_1813682 |
| 2497 | **PYCR1** | **3,6** | NM_153824 | ILMN_1796013 |
| 2498 | **MAP2K3** | **3,6** | NM_145110 | ILMN_1815238 |
| 2499 | **CALML4** | **3,6** | NM_001031733 | ILMN_1652389 |
| 2500 | **C22orf16** | **3,6** | NM_213720 | ILMN_1740170 |
| 2501 | **DENND1A** | **3,7** | NM_024820 | ILMN_1727315 |
| 2502 | **FKBP2** | **3,7** | NM_004470 | ILMN_1807563 |
| 2503 | **RAD51C** | **3,7** | NM_002876 | ILMN_1695386 |
| 2504 | **APRT** | **3,7** | NM_001030018 | ILMN_1726410 |
| 2505 | **MGC17839** | **3,7** | NM_174926 | ILMN_1815346 |
| 2506 | **TFPI2** | **3,7** | NM_006528 | ILMN_1667630 |
| 2507 | **TIGA1** | **3,7** | NM_053000 | ILMN_1654609 |
| 2508 | **CCL2** | **3,7** | NM_002982 | ILMN_1720048 |
| 2509 | **C20orf52** | **3,7** | NM_080748 | ILMN_1664429 |
| 2510 | **ATF3** | **3,7** | NM_001030287 | ILMN_1799748 |
| 2511 | **C9orf23** | **3,7** | NM_148179 | ILMN_1722589 |
| 2512 | **SNRPB2** | **3,7** | NM_003092 | ILMN_1771620 |
| 2513 | **HNRPDL** | **3,7** | NM_005463 | ILMN_1653432 |
| 2514 | **C3orf40** | **3,7** | NM_144635 | ILMN_1729217 |
| 2515 | **IL12A** | **3,7** | NM_000882 | ILMN_1671353 |
| 2516 | **MPP1** | **3,7** | NM_002436 | ILMN_1733675 |
| 2517 | **COL7A1** | **3,7** | NM_000094 | ILMN_1751161 |
| 2518 | **BCL2L12** | **3,7** | NM_138639 | ILMN_1731193 |
| 2519 | **SNRPF** | **3,8** | NM_003095 | ILMN_1678966 |
| 2520 | **ITPKA** | **3,8** | NM_002220 | ILMN_1776516 |
| 2521 | **TUBB3** | **3,8** | NM_006086 | ILMN_1791726 |
| 2522 | **PEA15** | **3,8** | NM_003768 | ILMN_1771376 |
| 2523 | **SLC25A3** | **3,8** | NM_213612 | ILMN_1704396 |
| 2524 | **C15orf48** | **3,8** | NM_032413 | ILMN_1805410 |
| 2525 | **RPL8** | **3,8** | NM_033301 | ILMN_1811433 |
| 2526 | **NIFUN** | **3,8** | NM_014301 | ILMN_1735432 |
| 2527 | **IRAK2** | **3,8** | NM_001570 | ILMN_1745964 |
| 2528 | **DCXR** | **3,8** | NM_016286 | ILMN_1681437 |
| 2529 | **C21orf34** | **3,8** | NM_001005732 | ILMN_1690703 |
| 2530 | **HSPC023** | **3,8** | NM_014047 | ILMN_1671374 |
| 2531 | **DKFZp564J157** | **3,8** | NM_001005354 | ILMN_1795944 |
| 2532 | **C1orf50** | **3,8** | NM_024097 | ILMN_1801941 |
| 2533 | **C3orf28** | **3,8** | NM_014367 | ILMN_1803647 |
| 2534 | **PIM3** | **3,9** | NM_001001852 | ILMN_1707748 |
| 2535 | **BOLA3** | **3,9** | NM_212552 | ILMN_1786658 |
| 2536 | **ATPIF1** | **3,9** | NM_016311 | ILMN_1727332 |
| 2537 | **CCDC58** | **3,9** | NM_001017928 | ILMN_1686920 |
| 2538 | **CKS2** | **3,9** | NM_001827 | ILMN_1756326 |
| 2539 | **FLJ25801** | **3,9** | NM_173553 | ILMN_1666893 |
| 2540 | **C6orf48** | **3,9** | NM_016947 | ILMN_1766446 |
| 2541 | **LOC317671** | **3,9** | NM_173362 | ILMN_1802162 |
| 2542 | **C20orf149** | **3,9** | NM_024299 | ILMN_1720430 |
| 2543 | **DDX10** | **3,9** | NM_004398 | ILMN_1753249 |
| 2544 | **ZSCAN2** | **3,9** | NM_001007072 | ILMN_1653163 |
| 2545 | **PPIH** | **3,9** | NM_006347 | ILMN_1801913 |
| 2546 | **FAM91A1** | **3,9** | NM_144963 | ILMN_1777322 |
| 2547 | **QTRT1** | **3,9** | NM_031209 | ILMN_1780153 |
| 2548 | **NOLC1** | **3,9** | NM_004741 | ILMN_1800224 |
| 2549 | **PRR7** | **4,0** | NM_030567 | ILMN_1677509 |
| 2550 | **NEDD4** | **4,0** | NM_006154 | ILMN_1807881 |
| 2551 | **RPL29** | **4,0** | NM_000992 | ILMN_1737517 |
| 2552 | **HIATL2** | **4,0** | NM_032318 | ILMN_1777058 |
| 2553 | **SDC4** | **4,0** | NM_002999 | ILMN_1666181 |
| 2554 | **SLC39A4** | **4,0** | NM_017767 | ILMN_1706386 |
| 2555 | **DFFA** | **4,0** | NM_004401 | ILMN_1667213 |
| 2556 | **ANKRD1** | **4,0** | NM_014391 | ILMN_1716264 |
| 2557 | **PAWR** | **4,0** | NM_002583 | ILMN_1806907 |
| 2558 | **LOC348262** | **4,0** | NM_207368 | ILMN_1733799 |
| 2559 | **MRPS18C** | **4,0** | NM_016067 | ILMN_1658416 |
| 2560 | **PMS2L5** | **4,0** | NM_174930 | ILMN_1759901 |
| 2561 | **SRM** | **4,0** | NM_003132 | ILMN_1661337 |
| 2562 | **MRPL41** | **4,1** | NM_032477 | ILMN_1705464 |
| 2563 | **TRA16** | **4,1** | NM_176880 | ILMN_1784512 |
| 2564 | **S100A11** | **4,1** | NM_005620 | ILMN_1750101 |
| 2565 | **ATP6V1F** | **4,1** | NM_004231 | ILMN_1678308 |
| 2566 | **PMS2** | **4,1** | NM_001018040 | ILMN_1677887 |
| 2567 | **KCTD13** | **4,2** | NM_178863 | ILMN_1786843 |
| 2568 | **mimitin** | **4,2** | NM_174889 | ILMN_1682857 |
| 2569 | **PRPH** | **4,2** | NM_006262 | ILMN_1723678 |
| 2570 | **NFKB2** | **4,2** | NM_002502 | ILMN_1799062 |
| 2571 | **CCL20** | **4,2** | NM_004591 | ILMN_1657234 |
| 2572 | **C21orf70** | **4,2** | NM_058190 | ILMN_1751301 |
| 2573 | **COX17** | **4,3** | NM_005694 | ILMN_1770885 |
| 2574 | **UPF2** | **4,3** | NM_015542 | ILMN_1739283 |
| 2575 | **RPL27A** | **4,3** | NM_000990 | ILMN_1716740 |
| 2576 | **PPFIA1** | **4,3** | NM_177423 | ILMN_1727050 |
| 2577 | **MICA** | **4,3** | NM_000247 | ILMN_1655675 |
| 2578 | **ACYP1** | **4,3** | NM_203488 | ILMN_1694233 |
| 2579 | **GABPB2** | **4,3** | NM_181427 | ILMN_1761147 |
| 2580 | **HSPC268** | **4,4** | NM_197964 | ILMN_1779751 |
| 2581 | **H2AFJ** | **4,4** | NM_177925 | ILMN_1708728 |
| 2582 | **NDUFA3** | **4,4** | NM_004542 | ILMN_1784641 |
| 2583 | **GPX1** | **4,4** | NM_201397 | ILMN_1749662 |
| 2584 | **FKBP1A** | **4,4** | NM_054014 | ILMN_1702237 |
| 2585 | **MSI2** | **4,4** | NM_170721 | ILMN_1804448 |
| 2586 | **GLIPR1** | **4,4** | NM_006851 | ILMN_1769245 |
| 2587 | **TNFRSF10B** | **4,4** | NM_003842 | ILMN_1775196 |
| 2588 | **POLR3K** | **4,4** | NM_016310 | ILMN_1801664 |
| 2589 | **ZCSL2** | **4,4** | NM_206831 | ILMN_1679912 |
| 2590 | **LOC285989** | **4,4** | NM_001013258 | ILMN_1810127 |
| 2591 | **UBL5** | **4,4** | NM_024292 | ILMN_1691379 |
| 2592 | **FBXO22** | **4,4** | NM_012170 | ILMN_1786469 |
| 2593 | **CLEC11A** | **4,4** | NM_002975 | ILMN_1807359 |
| 2594 | **MGC5509** | **4,4** | NM_024093 | ILMN_1701131 |
| 2595 | **FLCN** | **4,4** | NM_144606 | ILMN_1814952 |
| 2596 | **MGC13170** | **4,5** | NM_199249 | ILMN_1681124 |
| 2597 | **ANKRD39** | **4,5** | NM_016466 | ILMN_1710979 |
| 2598 | **NME1** | **4,5** | NM_198175 | ILMN_1713875 |
| 2599 | **CNFN** | **4,5** | NM_032488 | ILMN_1803838 |
| 2600 | **DIPA** | **4,5** | NM_006848 | ILMN_1657332 |
| 2601 | **CSTF3** | **4,5** | NM_001033505 | ILMN_1736161 |
| 2602 | **PRIM2A** | **4,5** | NM_000947 | ILMN_1694100 |
| 2603 | **TFAP2A** | **4,5** | NM_003220 | ILMN_1733135 |
| 2604 | **COTL1** | **4,5** | NM_021149 | ILMN_1788283 |
| 2605 | **SAMD4A** | **4,6** | NM_015589 | ILMN_1690466 |
| 2606 | **PHLDA2** | **4,6** | NM_003311 | ILMN_1671557 |
| 2607 | **RPL9** | **4,6** | NM_001024921 | ILMN_1729033 |
| 2608 | **NDUFC1** | **4,6** | NM_002494 | ILMN_1733603 |
| 2609 | **LSM7** | **4,6** | NM_016199 | ILMN_1678165 |
| 2610 | **XAGE1** | **4,7** | NM_020411 | ILMN_1691494 |
| 2611 | **HBQ1** | **4,7** | NM_005331 | ILMN_1696183 |
| 2612 | **AK2** | **4,7** | NM_001625 | ILMN_1670542 |
| 2613 | **CXCL1** | **4,7** | NM_001511 | ILMN_1787897 |
| 2614 | **ZNRD1** | **4,7** | NM_170783 | ILMN_1692486 |
| 2615 | **NFKBIB** | **4,7** | NM_002503 | ILMN_1690473 |
| 2616 | **UCRC** | **4,7** | NM_001003684 | ILMN_1781986 |
| 2617 | **Bles03** | **4,8** | NM_031450 | ILMN_1757847 |
| 2618 | **FLJ20186** | **4,8** | NM_017702 | ILMN_1656185 |
| 2619 | **FLJ20512** | **4,8** | NM_017854 | ILMN_1704024 |
| 2620 | **CREB5** | **4,8** | NM_001011666 | ILMN_1728677 |
| 2621 | **RPP21** | **4,9** | NM_024839 | ILMN_1717681 |
| 2622 | **UQCRH** | **4,9** | NM_006004 | ILMN_1792138 |
| 2623 | **CXCL2** | **4,9** | NM_002089 | ILMN_1682636 |
| 2624 | **FHIT** | **5,0** | NM_002012 | ILMN_1766123 |
| 2625 | **MAX** | **5,0** | NM_145113 | ILMN_1706546 |
| 2626 | **FAM24B** | **5,0** | NM_152644 | ILMN_1659536 |
| 2627 | **ATOX1** | **5,0** | NM_004045 | ILMN_1670609 |
| 2628 | **TGM2** | **5,1** | NM_198951 | ILMN_1679267 |
| 2629 | **SSSCA1** | **5,1** | NM_006396 | ILMN_1728714 |
| 2630 | **MGC33839** | **5,1** | NM_152353 | ILMN_1759545 |
| 2631 | **DRAP1** | **5,1** | NM_006442 | ILMN_1733048 |
| 2632 | **C6orf125** | **5,1** | NM_032340 | ILMN_1790461 |
| 2633 | **EMP3** | **5,1** | NM_001425 | ILMN_1765446 |
| 2634 | **HRK** | **5,2** | NM_003806 | ILMN_1668830 |
| 2635 | **C6orf1** | **5,2** | NM_001008703 | ILMN_1744196 |
| 2636 | **RPL41** | **5,2** | NM_021104 | ILMN_1710001 |
| 2637 | **SFN** | **5,3** | NM_006142 | ILMN_1806607 |
| 2638 | **MRPL21** | **5,3** | NM_181512 | ILMN_1654250 |
| 2639 | **RPS29** | **5,3** | NM_001030001 | ILMN_1719476 |
| 2640 | **USMG5** | **5,3** | NM_032747 | ILMN_1773313 |
| 2641 | **ARD1A** | **5,3** | NM_003491 | ILMN_1721977 |
| 2642 | **SLC27A5** | **5,4** | NM_012254 | ILMN_1725366 |
| 2643 | **C11orf48** | **5,4** | NM_024099 | ILMN_1739345 |
| 2644 | **RIS1** | **5,4** | NM_015444 | ILMN_1792455 |
| 2645 | **GAD1** | **5,5** | NM_013445 | ILMN_1660973 |
| 2646 | **Magmas** | **5,5** | NM_016069 | ILMN_1763884 |
| 2647 | **GDF15** | **5,5** | NM_004864 | ILMN_1763658 |
| 2648 | **FLJ43870** | **5,6** | NM_001001686 | ILMN_1792814 |
| 2649 | **PCBD1** | **5,6** | NM_001001939 | ILMN_1708813 |
| 2650 | **CUGBP1** | **5,7** | NM_198700 | ILMN_1686516 |
| 2651 | **DPM3** | **5,7** | NM_018973 | ILMN_1673323 |
| 2652 | **RPLP1** | **5,7** | NM_001003 | ILMN_1689725 |
| 2653 | **PFDN2** | **5,8** | NM_012394 | ILMN_1678754 |
| 2654 | **RPS7** | **5,9** | NM_001011 | ILMN_1750722 |
| 2655 | **ABL2** | **5,9** | NM_005158 | ILMN_1657870 |
| 2656 | **BTBD14A** | **5,9** | NM_144653 | ILMN_1809522 |
| 2657 | **RPL13** | **5,9** | NM_033251 | ILMN_1709039 |
| 2658 | **LOC205251** | **5,9** | NM_174925 | ILMN_1693685 |
| 2659 | **PTPN14** | **6,0** | NM_005401 | ILMN_1754830 |
| 2660 | **PCGF1** | **6,1** | NM_032673 | ILMN_1757956 |
| 2661 | **RPL14** | **6,1** | NM_003973 | ILMN_1726460 |
| 2662 | **BAX** | **6,1** | NM_004324 | ILMN_1683300 |
| 2663 | **PPP1R15A** | **6,1** | NM_014330 | ILMN_1659936 |
| 2664 | **PTRH1** | **6,1** | NM_001002913 | ILMN_1685240 |
| 2665 | **HIST2H2AC** | **6,1** | NM_003517 | ILMN_1768973 |
| 2666 | **LOC352909** | **6,2** | NM_001031802 | ILMN_1772074 |
| 2667 | **BOLA2** | **6,2** | NM_001031827 | ILMN_1659343 |
| 2668 | **RPL34** | **6,3** | NM_033625 | ILMN_1774823 |
| 2669 | **G1P2** | **6,3** | NM_005101 | ILMN_1813289 |
| 2670 | **ZNF593** | **6,3** | NM_015871 | ILMN_1703441 |
| 2671 | **PHLDA1** | **6,3** | NM_007350 | ILMN_1687978 |
| 2672 | **PSMB10** | **6,5** | NM_002801 | ILMN_1683026 |
| 2673 | **IGFL2** | **6,5** | NM_001002915 | ILMN_1745238 |
| 2674 | **IL32** | **6,7** | NM_001012632 | ILMN_1778010 |
| 2675 | **NHP2L1** | **6,8** | NM_001003796 | ILMN_1697614 |
| 2676 | **CXorf33** | **6,8** | NM_198450 | ILMN_1777483 |
| 2677 | **RPS21** | **6,9** | NM_001024 | ILMN_1800573 |
| 2678 | **NME1-NME2** | **7,3** | NM_001018136 | ILMN_1693430 |
| 2679 | **C12orf45** | **7,5** | NM_152318 | ILMN_1728435 |
| 2680 | **TMEM93** | **7,5** | NM_001014764 | ILMN_1794560 |
| 2681 | **HIST2H2AA** | **7,6** | NM_003516 | ILMN_1659047 |
| 2682 | **FOSL1** | **7,7** | NM_005438 | ILMN_1771841 |
| 2683 | **ATP5I** | **7,9** | NM_007100 | ILMN_1803509 |
| 2684 | **RPL36** | **7,9** | NM_015414 | ILMN_1662169 |
| 2685 | **DHRS9** | **8,2** | NM_005771 | ILMN_1733998 |
| 2686 | **ATP5G1** | **8,2** | NM_005175 | ILMN_1712430 |
| 2687 | **NRG1** | **8,4** | NM_013961 | ILMN_1737252 |
| 2688 | **ERN1** | **8,7** | NM_152461 | ILMN_1698404 |
| 2689 | **CALCB** | **8,7** | NM_000728 | ILMN_1694427 |
| 2690 | **CST1** | **8,8** | NM_001898 | ILMN_1753449 |
| 2691 | **GADD45A** | **9,6** | NM_001924 | ILMN_1694075 |
| 2692 | **DDIT3** | **9,8** | NM_004083 | ILMN_1676984 |
| 2693 | **RBP1** | **9,8** | NM_002899 | ILMN_1656837 |
| 2694 | **FLT4** | **10,0** | NM_002020 | ILMN_1651296 |
| 2695 | **MT1X** | **10,1** | NM_005952 | ILMN_1775170 |
| 2696 | **MDM2** | **10,4** | NM_002392 | ILMN_1736829 |
| 2697 | **RPS15** | **11,2** | NM_001018 | ILMN_1691807 |
| 2698 | **TNFSF7** | **11,3** | NM_001252 | ILMN_1760247 |
| 2699 | **TNFRSF12A** | **11,3** | NM_016639 | ILMN_1689004 |
| 2700 | **TM4SF19** | **11,6** | NM_138461 | ILMN_1808325 |
| 2701 | **SOD2** | **11,6** | NM_001024465 | ILMN_1775672 |
| 2702 | **EGR1** | **12,3** | NM_001964 | ILMN_1762899 |
| 2703 | **CDCP1** | **12,6** | NM_178181 | ILMN_1708167 |
| 2704 | **PDLIM7** | **12,7** | NM_213636 | ILMN_1690125 |
| 2705 | **MT1A** | **15,2** | NM_005946 | ILMN_1691156 |
| 2706 | **CCRN4L** | **16,6** | NM_012118 | ILMN_1689378 |
| 2707 | **UPP1** | **16,8** | NM_181597 | ILMN_1798256 |
| 2708 | **VGF** | **21,7** | NM_003378 | ILMN_1757497 |
| 2709 | **MT2A** | **23,2** | NM_005953 | ILMN_1690957 |
| 2710 | **IL8** | **40,3** | NM_000584 | ILMN_1666733 |
| 2711 | **CDKN1A** | **57,0** | NM_078467 | ILMN_1787212 |
