# Supplementary material for: Enzastaurin inhibits invasion and metastasis in lung cancer by diverse molecules
Source: Br J Cancer. 2010 Aug 24;103(6):802–11. doi: 10.1038/sj.bjc.6605818 (PMC2966618; doi:10.1038/sj.bjc.6605818)
Supplement: Supplementary Figure Legends [file 6605818x8.doc]

**Supplementary figure 1.**

**Enzastaurin inhibits PKC, AKT, p42/22 and GSK3β activation**

a,b,c,d) p-PKC, p-AKT, p-p42/44 and p-GSK3β bands was quantified using densitometry method. The bands were normalised either with the respective endogenous expression of the molecule or with the β–Actin.

**Supplementary figure 2.**

**Enzastaurin inhibits Sp1, Sp3 and c-Jun gene expression**

a,b,c) Quantification of Sp1, Sp3 and c-Jun mRNA by Real-time-sybr green-PCR from A549 and H1299 cells after 24 or 48 hours of DMSO or Enz (IC_50_) treatment. Gene expression was normalized against GAPDH mRNA. *:p<0.05. d) Under similar conditions as Real-time-PCR, Western blot analysis was performed with A549 and H1299 cells after 48h of Enz treatment with specific antibodies against the indicated molecules were used, together with anti-beta-Actin as a loading control.

**Supplementary figure 3.**

Enzastaurin inhibits Sp1, Sp3 and c-Jun binding to u-PAR promoter either with. or without, EGF stimulatiuon

a) EMSA performed with labelled u-PAR-Sp1 (-152/-135) or with u-PAR-AP1 (-190/-171) oligonucleotides in the presence, or absence, of a 100-fold excess of unlabeled competitor oligonucleotides, and following EGF stimulation. 5µg of nuclear extract from A549 cells from the different treatments (described in Material and Methods) was used and complexes were analyzed by gel electrophoresis. Specific complexes are shown with brackets. Free probe is shown at the bottom of the gel. b,c) Supershift assay performed with 5µg of nuclear extract from A549 cells (treated with DMSO or Enz for 24h) for the oligos explained above, with antibodies against the respective transcription factor family members. Specific bands are shown with arrows and labelled with the respective transcription factor. d) Schematic representation of the u-PAR promoter with the transcription factor binding motifs investigated and the primer binding positions used for ChIP.

**Supplementary figure 4.**

u-PAR knockdown cells were more sensitive to Enz treatment.

a,b) Confirmation of si-RNA/sh-RNA knockdown of u-PAR by ELISA and u-PAR protein amounts after Enz treatment.
